# Adverse childhood experiences and severity levels of inflammation and depression from childhood to young adulthood: a longitudinal cohort study

**DOI:** 10.1038/s41380-022-01478-x

**Published:** 2022-03-03

**Authors:** Eleonora Iob, Rebecca Lacey, Valentina Giunchiglia, Andrew Steptoe

**Affiliations:** 1 Department of Behavioural Science and Health, University College London, London, UK; 2 Department of Epidemiology and Public Health, University College London, London, UK; 3 Department of Brain Sciences, Faculty of Medicine, Imperial College London, London, UK

**Keywords:** Depression, Predictive markers

## Abstract

Adverse childhood experiences (ACEs) are associated with depression and systemic inflammation in adults. However, limited longitudinal research has tested these relationships in children and young people, and it is unclear whether inflammation is an underlying mechanism through which ACEs influence depression. We examined the longitudinal associations of several ACEs across different early-life periods with longitudinal patterns of early-life inflammation and depression in young adulthood and assessed the mediating role of inflammation. The data came from the Avon Longitudinal Study of Parents and Children (*N* = 3931). ACEs from the prenatal period through to adolescence were operationalised using cumulative scores, single adversities, and dimensions derived through factor analysis. Inflammation (C-reactive protein) was measured on three occasions (9–18 years) and depressive symptoms were ascertained on four occasions (18–23 years). Latent class growth analysis was employed to delineate group-based trajectories of inflammation and depression. The associations between ACEs and the inflammation/depression trajectories were tested using multinomial logistic regression analysis. Most types of ACEs across all early-life periods were associated with elevated depression trajectories, with larger associations for threat-related adversities compared with other ACEs. Bullying victimisation and sexual abuse in late childhood/adolescence were associated with elevated CRP trajectories, while other ACEs were unrelated to inflammation. Inflammation was also unrelated to depression and did not mediate the associations with ACEs. These results suggest that ACEs are consistently associated with depression, whereas the associations of inflammation with ACEs and depression are weak in young people. Interventions targeting inflammation in this population might not offer protection against depression.

## Introduction

Adverse childhood experiences (ACEs) (e.g., abuse, family dysfunction, bullying) are associated with an increased risk of depression in both children and adults [1, 2]. Elevated systemic inflammation is a plausible mechanism through which ACEs might be translated into biological risk for depression [3]. Several observational studies have demonstrated that adults exposed to ACEs often exhibit elevated levels of inflammatory markers, such as C-reactive protein (CRP) and interleukins (ILs) [4, 5]. Meta-analyses of clinical and population-based studies have also found that depression is associated with chronic systemic inflammation in adults [6, 7]. However, most research to date has focused on adults, while there is a paucity of studies that have examined the associations of systemic inflammation with ACEs and depression in children and adolescents. Furthermore, the majority of these studies were cross-sectional, only included single assessments of inflammation and depression, and did not formally test the plausible role of inflammation as a mediating mechanism through which ACEs may become biologically embedded and contribute to the pathogenesis of depression.

Another important limitation of the existing literature concerns the role of the timing and patterning of ACEs in their associations with depression and systemic inflammation. Various conceptual models have been proposed in an attempt to explain the biological embedding of ACEs, including the accumulation, recency, and sensitive period models [8, 9]. However, studies to date have predominantly used retrospective assessments of ACEs and have neglected the timing of ACEs. The few studies explicitly testing the impact of the timing of ACEs on mental health have revealed mixed findings. Some research suggests that early childhood and adolescence are periods of heightened vulnerability to adversity [10–12], while other studies found that the effects of ACEs on psychopathology symptoms were primarily explained by recency and/or accumulation effects [9, 13]. Furthermore, studies examining the impact of the timing of ACEs on inflammation are lacking. It is also currently unknown whether certain ACEs or combinations of ACEs might be more strongly related to depression and inflammation than others [14]. Some studies have shown that childhood adversities involving threat (e.g., psychological or physical abuse) have larger associations with unfavourable biological and mental health outcomes than other ACEs [15, 16]. However, this work has concentrated on specific adversities in isolation without considering their interrelated nature. Recent research using factor analysis (FA; variable-centred method) and latent class analysis (LCA; person-centred method) has demonstrated that ACEs clusters related to abuse, household dysfunction, parental absence, and poor parent–child relationships were all associated with increased inflammation in mid and later life [5, 17]. In contrast, another study found that neither LCA-derived clusters nor cumulative ACEs scores were related to inflammation at age 9 years [18].

To address these gaps, we examined the associations between several types of ACEs from the prenatal period through to adolescence with longitudinal patterns of early-life inflammation (9–18 years) and depressive symptoms in young adulthood (18–23 years) in a large population cohort, considering both the timing and patterning of ACEs. Furthermore, we tested the mediating role of inflammation in the associations between ACEs and later depressive symptoms (Supplemental Information (SI), sIntroduction for the specific hypotheses that were tested).

## Materials and methods

### Sample

The participants were drawn from the Avon Longitudinal Study of Parents and Children (ALSPAC), a prospective observational cohort study investigating influences on health and development across the life course [19, 20] (SI, sMethods for further details). The present analysis uses data from the prenatal period through to 23 years of age. We focused on adversities experienced throughout the entire childhood period (prenatal to 18 years), as well as during specific early-life periods, namely: prenatal period (18 weeks gestation to birth), early childhood (0–3 years), middle childhood (>3–7 years), late childhood (>7–12 years), and adolescence (>12–18 years). The analytical sample was defined as children with at least 10% of ACEs data across all early-life periods, one measure of inflammation, and one measure of depressive symptoms (*N* = 3931). Supplementary Fig. S1a provides an overview of the study design and assessment timepoints. Written informed consent was obtained from the parents of the study children. Ethical approval was granted by the ALSPAC Ethics and Law Committee and the Local Research Ethics Committees.

### Measures

#### Adverse childhood experiences (ACEs)

ACEs from the prenatal period up to adolescence were assessed repeatedly using both prospectively (~90%) and retrospectively collected information, reported by the parents and/or the study children. These included physical abuse, emotional abuse/neglect, sexual abuse, bullying, household violence, parental substance use problems, parental mental health problems, parental convictions, parental separation, and low parent–child bonding (SI, sMethods for further information). Supplementary Fig. S1b shows the definitions, respondents, and time periods of the ACEs measures. The exact variables, timepoints, methods of data collection, and dichotomisation criteria for each ACE construct are reported in Supplementary Tables S1–S5.

First, for each early-life period considered in the analysis (i.e., prenatal, 0–3, 3–7, 7–12, and 12–18 years), binary ACEs constructs were derived representing any exposure to the relevant adversity in the specified period, as described elsewhere [21] (SI, sMethods). Second, cumulative risk scores were calculated representing the total number of ACEs experienced by the participant throughout the entire childhood period and during each specific early-life period. Third, FA was applied to the individual ACEs constructs to identify distinct dimensions of ACEs, namely clusters of ACEs that tend to co-occur in the sample. Of note, the use of these different yet complementary methods for operationalising ACEs allowed us to conduct a more comprehensive and rigorous assessment of ACEs, and to understand the role of distinct aspects of ACEs (i.e., cumulative exposure, type of adversity, and underlying dimensions) in their relationships with inflammation and depression. In addition, since earlier research in ALSPAC has investigated the clustering of ACEs using LCA [18], the application of FA enabled us to assess how the results compare across different data reduction techniques.

#### Early-life inflammation

Early-life inflammation was indexed by CRP. This biomarker was chosen because the ALSPAC resource includes repeated measures of CRP, whereas other inflammatory markers (e.g., IL-6) were assessed only once. Non-fasting blood samples for the analysis of CRP were taken from the study children at 9, 15 and 18 years of age (SI, sMethods for further information). The CRP measures were used as continuous variables and were log-transformed to make their distribution closer to normality and reduce potential skewness in the regression residuals. These data were then used to delineate group-based trajectories of CRP representing different longitudinal patterns of inflammation from childhood up to adolescence (see ‘Statistical analyses’).

#### Depressive symptoms

Self-reported depressive symptoms in young adulthood were assessed using the Short Mood and Feelings Questionnaire (SMFQ) [22] when the study children were aged 18, 21, 22 and 23 years (SI, sMethods for further information). The total SMFQ scores at the four timepoints were then used to identify distinct group-based trajectories representing different longitudinal patterns of depression during young adulthood.

#### Covariates

Possible confounders of the associations between ACEs, inflammation, and depressive symptoms were selected based on previous studies in the field. These were sex—also analysed as a possible effect modifier; ethnicity; the mother’s marital status at the time of birth of the study child; the mother’s highest educational qualification at the time of birth; the parents’ social class at the time of birth; and whether the mother had smoked during pregnancy since this is linked to maternal stress during pregnancy [23] and can also have adverse effects on the child’s neurodevelopment [24] (SI, sMethods for the specific coding of the covariates).

### Statistical analyses

**Fig. 1:**
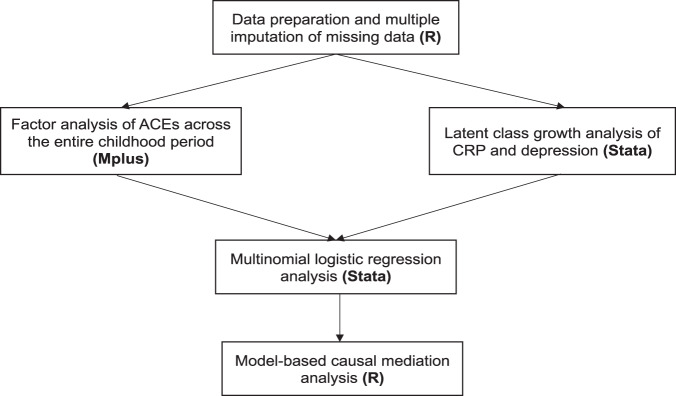
Flowchart of the statistical analyses. (i) Data preparation and multiple imputation of missing data were conducted in R; (ii) Factor analysis of ACEs was performed in Mplus, and Latent class growth analysis of CRP and depression was performed in Stata; (iii) Multinomial logistic regression analysis was conducted in Stata; (iv) Model-based causal mediation analysis was conducted in R.

Explorative factor analysis (EFA) and confirmatory factor analysis (CFA) were employed to identify distinct dimensions of adversity underlying the ACEs constructs, as described elsewhere [25]. EFA and CFA were conducted on the ACE variables covering the entire childhood period (prenatal to 18 years) (SI, sMethods for further details). In the regression analysis, the resulting FA-derived ACEs dimensions were indexed by binary indicators representing the presence of any exposure to the relevant ACEs. Latent class growth analysis [26] was used to delineate group-based longitudinal trajectories of CRP and depressive symptoms. This method enabled us to group the study participants into distinct groups representing different levels of depressive symptoms and CRP over time (SI, sMethods for further details). The resulting group-based trajectories were then used as outcome variables in the regression analysis. The associations between ACEs, CRP trajectories, and depression trajectories were tested using multinomial logistic regression analysis. For the associations of ACEs with CRP and depression, the following models were tested: Model 1—associations of ACEs (i.e., cumulative scores, dimensions, individual adversities) during specific early-life periods and throughout childhood with the CRP/depression trajectories, adjusted for all covariates; Model 2—Model 1 + adjustment for ACEs experienced during previous early-life periods to disentangle possible sensitive period versus accumulation effects; Model 3—Model 2 + interaction effect between the ACE variable and the child’s sex. For the associations involving ACEs throughout the entire childhood period, an additional regression model (Model 4) tested the associations of single-exposure (i.e., once) and multiple-exposure (i.e., twice or more) to ACEs with the CRP/depression trajectories, adjusted for all covariates, to examine possible accumulation effects over time. Furthermore, Model 5 tested the mutually adjusted associations of the ACEs dimensions to assess the independence of their effects. Regarding the associations between CRP and depression, the following models were tested: Model 1—associations between CRP trajectories and depression trajectories, adjusted for all covariates; Model 2—Model 1 + interaction effect between CRP and the child’s sex. Lastly, model-based causal mediation analysis [27] was performed to examine the mediating role of early-life inflammation in the relationship between ACEs across the entire childhood period and patterns of depressive symptoms during young adulthood (SI, sMethods for further details). Missing data on all variables were accounted for using multiple imputation by chained equations (MICE) under the Missing at Random assumption. All study variables were used as predictors of the missing data generation mechanism. Twenty imputed datasets were created, and the estimates from all statistical analyses were pooled using Rubin’s rules [28]. Data management, MICE, and mediation analysis were performed using R version 4.0.2. EFA and CFA were conducted using Mplus version 7. Latent class growth analysis and regression analysis were conducted in Stata version 16. The flowchart of the statistical analyses is illustrated in Fig. 1. In sensitivity analyses, (i) we calculated false discovery rate (FDR)-corrected *p* values for the associations of ACEs with CRP and depressive symptoms tested in the main imputed data analysis in order to account for multiple testing; (ii) we restricted the analyses to participants with complete data on all variables; (iii) we investigated differences in the baseline characteristics of the analytical sample versus participants excluded due to missing data on the exposure or outcome variables; (iv) we tested the associations of ACEs with individual CRP measures and with the rate of change in CRP levels across the timepoints using growth curve mixed-effects modelling, as well as the associations of the average CRP score across the timepoints with depressive symptoms; (v) we adjusted the associations of CRP with ACEs and depressive symptoms for body mass index at age 15 years; and lastly (vi) we compared the associations of child-reported versus parent-reported emotional neglect (16 years) with depressive symptoms in order to explore potential differences in the strength of the associations between reporting sources.

## Results

### Descriptive statistics

The characteristics of the study participants in the observed and imputed data are presented in Supplementary Table S6a. The distribution of the observed and imputed data was similar, suggesting that the MICE analysis achieved its goals. Prevalence statistics of ACEs across specific early-life periods are illustrated in Supplementary Fig. S2 and reported in Supplementary Table S6b. Further details on the descriptive analyses are provided in the SI (sResults).

### ACEs dimensions and group-based trajectories of CRP and depression

**Fig. 2:**
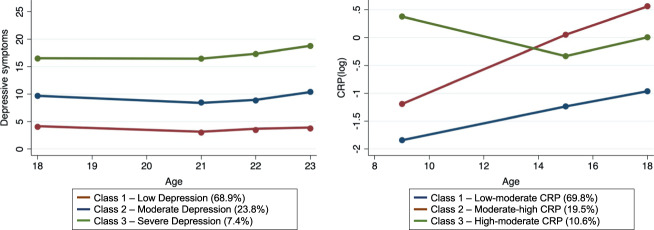
Group-based trajectories of depressive symptoms and CRP. Sample: ALSPAC (*N* = 3931). The latent class growth analysis resulted in the following trajectories: (1) Depression: Class 1—low depressive symptoms (68.9%), representing participants with low depressive symptoms at all assessments; Class 2—moderate depressive symptoms (23.8%), representing participants with moderate depressive symptoms at all assessments; Class 3—severe depressive symptoms (7.4%), representing participants with high levels of depressive symptoms at all assessments; (2) CRP: Class 1—low-moderate CRP (69.8%), representing participants with low levels in late childhood and moderate levels in adolescence; Class 2—moderate-high CRP (19.5%), representing participants with moderate levels in late childhood and high levels in adolescence; Class 3—high-moderate CRP (10.6%), representing participants with high levels in late childhood but lower levels in adolescence. The ‘low depression’ and ‘low-moderate CRP’ trajectories were used as reference categories in multinomial logistic regression analysis.

Using FA, two distinct dimensions of ACEs were identified, namely Physical/Emotional Threat (including physical abuse, emotional abuse/neglect, and low parent–child bonding) and Household Dysfunction (including household violence, parental substance use problems, parental mental health problems, parental convictions, and parental separation). Of note, these two dimensions do not encompass sexual abuse and bullying; these ACEs did not correlate well with any of the latent factors in EFA and were therefore included in the final CFA model as standalone factors (SI, sResults for further information). Latent class growth analysis identified the following group-based trajectories (Supplementary Table S8 for the fit indices of the 2- to 4-class models): Depression: Class 1—low depressive symptoms (68.9%), representing participants with low depressive symptoms at all assessments; Class 2—moderate depressive symptoms (23.8%), representing participants with moderate depressive symptoms at all assessments; Class 3—severe depressive symptoms (7.4%), representing participants with persistently high levels of depressive symptoms; CRP: Class 1—low-moderate CRP (69.8%), representing participants with low CRP levels in late childhood and moderate levels in adolescence; Class 2—moderate-high CRP (19.5%), representing participants with moderate CRP levels in late childhood and high levels in adolescence; Class 3—high-moderate CRP (10.6%), representing participants with high CRP levels in late childhood but lower levels in adolescence (Fig. 2).

### Associations of ACEs with inflammation and depressive symptoms

#### ACEs prenatal period

**Fig. 3:**
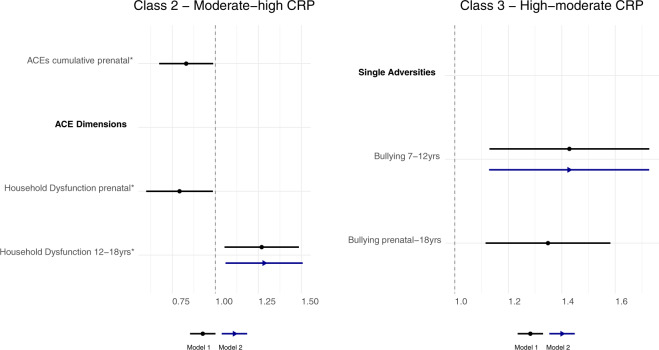
Associations between ACEs (prenatal to 18 years) and CRP trajectories (9–18 years). Sample: ALSPAC (*N* = 3931). Pooled estimates and 95% confidence intervals from multinomial logistic regression models across 20 imputed datasets. Model 1—adjusted for sex, ethnicity, maternal smoking during pregnancy, mother’s marital status, mother’s education, and household’s social class; Model 2—Model 1 + ACEs experienced during previous early-life periods. The associations are statistically significant at the 95% confidence level if the confidence interval does not cross 1 (dotted line of graph), and those marked with an asterisk symbol (*) were no longer significant following the correction for multiple comparisons. Reference trajectory: low-moderate CRP levels.

**Fig. 4:**
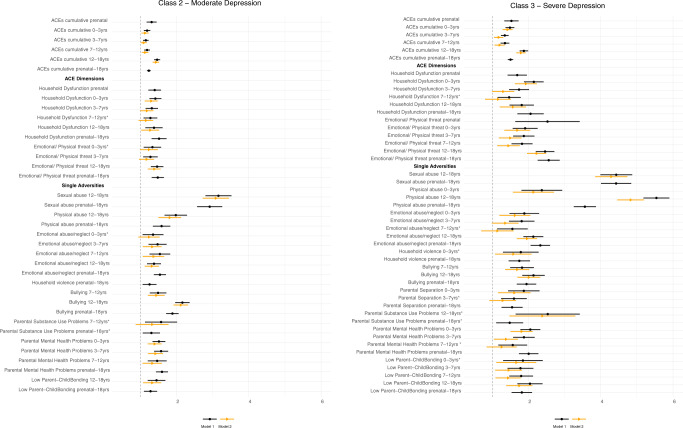
Associations between ACEs (prenatal to 18 years) and depression trajectories (18–23 years). Sample: ALSPAC (*N* = 3931). Pooled estimates and 95% confidence intervals from multinomial logistic regression models across 20 imputed datasets. Model 1—adjusted for sex, ethnicity, maternal smoking during pregnancy, mother’s marital status, mother’s education, and household’s social class; Model 2—Model 1 + ACEs experienced during previous early-life periods. The associations are statistically significant at the 95% confidence level if the confidence interval does not cross 1, and those marked with an asterisk symbol (*) were no longer significant following the correction for multiple comparisons. Reference trajectory: low depressive symptoms.

Cumulative exposure to ACEs during the prenatal period was related to a lower risk of moderate-high CRP levels, compared with low-moderate levels (OR = 0.83 [95% CI: 0.67; 0.99] *p* = 0.020). This association was driven by the dimension representing household dysfunction (OR = 0.79 [95% CI: 0.60; 0.99] *p* = 0.018) (Fig. 3, Model 1), while the emotional/physical threat dimension was unrelated to patterns of inflammation (Supplementary Table S9). The ACEs cumulative score was related to a higher risk of both moderate (OR = 1.31 [95% CI: 1.17; 1.45] *p* < 0.001) and severe (OR = 1.53 [95% CI: 1.33; 1.73] *p* < 0.001) depression trajectories (versus low), and both ACEs dimensions were related to severe depression trajectories (household dysfunction: OR = 1.69 [95% CI: 1.43; 1.96] *p* < 0.001; emotional/physical threat: OR = 2.53 [95% CI: 1.64; 3.42] *p* = 0.041) (Fig. 4 and Supplementary Table S10, Model 1). Since several individual adversities had a low prevalence in the prenatal period (Supplementary Table S6b), their associations with the outcomes were not tested in regression analysis.

#### ACEs 0–3 years (early childhood)

All types of ACEs (i.e., cumulative scores, dimensions, and single adversities) between 0 and 3 years were unrelated to inflammation (Supplementary Table S11). Several associations were found for depressive symptoms (Supplementary Table S12). The ACEs cumulative score was positively associated with both moderate (OR = 1.19 [95% CI: 1.10; 1.28] *p* < 0.001) and severe (OR = 1.49 [95% CI: 1.37; 1.61] *p* < 0.001) depression trajectories. The two ACEs dimensions were also positively associated with both depression trajectories (household dysfunction: OR_Moderate_ = 1.42 [95% CI: 1.25; 1.58] *p* < 0.001; OR_Severe_ = 2.15 [95% CI: 1.88; 2.42] *p* < 0.001; emotional/physical threat: OR_Moderate_ = 1.34 [95% CI: 1.10; 1.58] *p* = 0.018; OR_Severe_ = 1.91 [95% CI: 1.57; 2.25] *p* = 0.001). Among the single adversities, parental mental health problems and emotional abuse/neglect were related to moderate depressive symptoms. In addition, except for parental convictions and substance use problems, all other adversities were associated with severe depressive symptoms (Fig. 4, Model 1).

#### ACEs 3–7 years (middle childhood)

ACEs between 3 and 7 years were associated with depressive symptoms (Supplementary Table S14), but not with inflammation (Supplementary Table S13). The ACEs cumulative score (OR_Moderate_ = 1.15 [95% CI: 1.08; 1.23] *p* < 0.001; OR_Severe_ = 1.35 [95% CI: 1.24; 1.45] *p* < 0.001) and both ACEs dimensions (emotional/physical threat: OR_Moderate_ = 1.28 [95% CI: 1.08; 1.48] *p* = 0.015; OR_Severe_ = 1.87 [95% CI: 1.58; 2.17] *p* < 0.001; household dysfunction: OR_Moderate_ = 1.32 [95% CI: 1.15; 1.49] *p* = 0.001; OR_Severe_ = 1.74 [95% CI: 1.47; 2.02] *p* < 0.001) were positively associated with moderate and severe depression trajectories. Among the single adversities, parental mental health problems and emotional abuse/neglect were related to both moderate and severe depression trajectories. In addition, parental separation and low parent–child bonding were associated with severe depressive symptoms (Fig. 4, Model 1).

#### ACEs 7–12 years (late childhood)

Exposure to bullying victimisation between 7 and 12 years was associated with a higher risk of high-moderate CRP trajectories (OR = 1.43 [95% CI: 1.13; 1.73] *p* = 0.019) (Fig. 3, Model 1). Other ACEs were not associated with CRP (Supplementary Table S15). Regarding depressive symptoms (Supplementary Table S16), the cumulative ACEs score was associated with a higher risk of moderate (OR = 1.19 [95% CI: 1.11; 1.27] *p* < 0.001) and severe (OR = 1.35 [95% CI: 1.23; 1.47] *p* < 0.001) depression trajectories. The household dysfunction (OR = 1.47 [95% CI: 1.15; 1.79] *p* = 0.018) and emotional/physical threat (OR = 1.82 [95% CI: 1.53; 2.11] *p* < 0.001) dimensions were significantly associated with severe depressive symptoms, and household dysfunction was also related to moderate depressive symptoms (OR = 1.28 [95% CI: 1.09; 1.47] *p* = 0.011). Among the single adversities, emotional abuse/neglect, bullying, and parental mental health problems were related to both moderate and severe depressive symptoms. In addition, low parent–child bonding was associated with the severe depression trajectory, while parental substance use problems were only related to the moderate trajectory (Fig. 4, Model 1).

#### ACEs 12–18 years (adolescence)

The household dysfunction dimension (OR = 1.27 [95% CI: 1.05; 1.49] *p* = 0.030) was associated with a higher risk of moderate-high CRP trajectories (Fig. 3, Model 1). Other ACEs were not associated with inflammation (Supplementary Table S17). In relation to depressive symptoms (Supplementary Table S18), the ACEs cumulative score (OR_Moderate_ = 1.47 [95% CI: 1.38; 1.55] *p* < 0.001; OR_Severe_ = 1.87 [95% CI: 1.76; 1.99] *p* < 0.001) and the two ACEs dimensions (emotional/physical threat: OR_Moderate_ = 1.47 [95% CI: 1.29; 1.64] *p* < 0.001; OR_Severe_ = 2.46 [95% CI: 2.21; 2.72] *p* < 0.001; household dysfunction: OR_Moderate_ = 1.38 [95% CI: 1.14; 1.62] *p* = 0.008; OR_Severe_ = 1.82 [95% CI: 1.48; 2.15] *p* = 0.001) were all related to both moderate and severe depressive symptoms. Among the single adversities, sexual abuse, physical abuse, emotional abuse/neglect, bullying, and low parent–child bonding were related to both moderate and severe depressive symptoms. Parental substance use problems were only associated with severe depressive symptoms (Fig. 4, Model 1).

#### ACEs prenatal-18 years (entire childhood period)

The associations of ACEs with the CRP and depression trajectories mirrored those found across specific early-life periods (Figs. 3 and 4 and Supplementary Tables S19 and S20, Model 1).

### Timing and patterning of ACEs

Most time-specific associations between ACEs and the outcomes remained after accounting for earlier ACEs, but their magnitude was smaller than in the previous models (Supplementary Tables S11–S18 and Figs. 3 and 4, Model 2). Of note, the associations of ACEs between 12 and 18 years with depressive symptoms were somewhat larger compared with those of earlier periods, even after adjustment for previous ACEs (see confidence intervals in Fig. 4). Sexual abuse and physical abuse at 12–18 years had the largest associations with depressive symptoms.

**Fig. 5:**
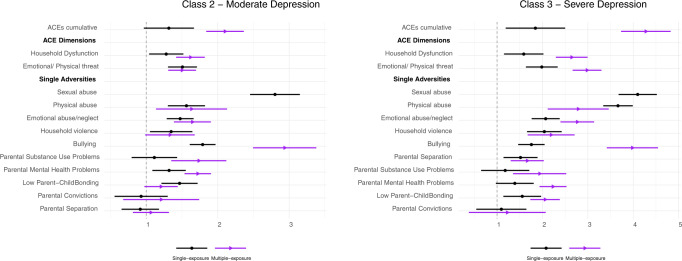
Associations of single and multiple exposures to ACEs (prenatal to 18 years) with depression trajectories (18–23 years). Sample: ALSPAC (*N* = 3931). Pooled estimates and 95% confidence intervals from multinomial logistic regression models across 20 imputed datasets. Model 4—adjusted for sex, ethnicity, maternal smoking during pregnancy, mother’s marital status, mother’s education, and household’s social class. The associations are statistically significant at the 95% confidence level if the confidence interval does not cross 1 (dotted line of graph). The reference group for both single and multiple exposures to ACEs is ‘no ACE exposure’. The multiple-exposure group was not tested for sexual abuse as only a few participants reported experiences of sexual abuse twice during childhood. Reference trajectory: low depressive symptoms.

Regarding the accumulation effects of ACEs (Model 4), both single and multiple exposure to most ACEs, except bullying, were unrelated to CRP levels (Supplementary Table S21). The associations of multiple exposure to ACEs with moderate and severe levels of depressive symptoms were generally larger than those involving single ACEs exposure, across most ACEs variables (Fig. 5 and Supplementary Table S22).

The mutually adjusted associations of the ACEs dimensions with the CRP and depression trajectories were almost identical to those found in the analysis testing each dimension individually (Supplementary Tables S23 and S24, Model 5). Based on this model, the emotional/physical threat dimension during adolescence had larger associations with severe levels of depression than the household dysfunction dimension, which is in agreement with the results involving single adversities. The associations of the ACEs dimensions with depression in other early-life periods did not differ significantly (Supplementary Table S24).

### Sex differences

The most notable sex difference was found for the association of sexual abuse 12–18 years with moderate-high CRP trajectories, suggesting that this adversity was associated with higher CRP levels in boys but not in girls (OR interaction effect = 0.13 [95% CI: 0.04; 0.44] *p* = 0.001) (Supplementary Fig. S4). Other sex differences in the relationship between ACEs and CRP were only small and inconsistent across different early-life periods (Supplementary Fig. S4 and Supplementary Table S25). Furthermore, most interaction effects between ACEs and the child’s sex were not related to depressive symptoms (Supplementary Fig. S5 and Supplementary Table S26).

### Associations between inflammation and depressive symptoms and mediation analysis

Neither the CRP trajectories nor the individual CRP measures (age 9, 15, and 18 years) were associated with moderate or severe trajectories of depressive symptoms (Supplementary Table S27). Likewise, no associations were found between the CRP trajectories/individual CRP measures and the risk of clinically significant depressive symptoms (i.e., SMFQ total score ≥10^29^) at 18, 21, or 23 years of age (Supplementary Table S28). As expected, all mediation effects of CRP in the associations between ACEs and depression were almost null and nonsignificant (Supplementary Table S29).

### Sensitivity analyses

Given the large number of tests conducted, we calculated FDR corrected *p* values for the associations between ACEs and the CRP/depression trajectories. Furthermore, the regression and mediation models were retested in a restricted sample with complete data on all variables. The associations of the household dysfunction dimension (prenatal period and adolescence) with CRP were not robust to the adjustment for multiple comparisons, while the other results were broadly consistent with those of the main analysis (SI, sResults for further information). Of note, the association of depression at age 18 years with child-reported emotional neglect (OR = 1.40 [95% CI: 1.04; 1.89] *p* = 0.027) was larger than that with parent-reported emotional neglect (OR = 1.11 [95% CI: 0.93; 1.31] *p* = 0.247) (Supplementary Table S50).

## Discussion

### Summary of main findings

Using data from a large population cohort, we investigated the associations of ACEs with longitudinal patterns of early-life inflammation and depressive symptoms in young adulthood, considering both the patterning and timing of ACEs from the prenatal period through to adolescence. The associations of the cumulative ACEs scores and the FA-derived dimensions with the CRP trajectories were weak in all early-life periods. Bullying victimisation between 7 and 18 years was the only individual adversity associated with elevated CRP levels, and exposure to sexual abuse in adolescence was related to elevated CRP levels in boys but not in girls, independently of previous ACEs and other covariates. The cumulative ACEs scores, FA-derived ACEs dimensions, and most individual adversities across all early-life periods were related to moderate and severe levels of depressive symptoms in young adulthood. The associations of ACEs during late childhood/adolescence with depressive symptoms were larger than those of earlier ACEs, also when accounting for previous ACEs exposure. The largest associations were found for sexual abuse and adversities involving physical/emotional threat in adolescence. Furthermore, multiple exposure to the same ACEs throughout childhood was more strongly related to depression than single ACEs exposure. In contrast, longitudinal patterns as well as single measures of inflammation during childhood/adolescence were unrelated to depressive symptoms in young adulthood, and early-life CRP levels did not mediate the associations between ACEs and depressive symptoms.

### Findings regarding depressive symptoms

Our results expand the existing literature by showing that most ACEs across all early-life periods were related to persistently elevated levels of depressive symptoms in young adulthood. This suggests that ACEs experienced at any stage of childhood might increase the individual’s vulnerability to poor mental health in adulthood. Furthermore, the results provide some evidence for a later sensitive period (or recency) and accumulation effects of ACEs. As previously suggested [8], sensitive period, accumulation, and recency effects might all contribute to the widespread associations between ACEs and depression. More precisely, all early-life periods considered in the present analysis may be particularly sensitive to the effects of ACEs on mental health; there may also be a dose-response relationship between ACEs and mental health, involving the cumulative experience of similar or different types of ACEs throughout childhood; and the impact of ACEs on mental health may be stronger when the adversity occurred close to the assessment of mental health.

Sexual abuse and adversities involving physical and emotional threats, particularly during adolescence, had larger associations with depression in young adulthood than other ACEs. This finding is consistent with previous work suggesting that childhood adversities involving threat are more strongly related to unfavourable biological and mental health outcomes than other ACEs [15, 16]. ACEs of a less severe and more common nature, such as parental separation/divorce, were not consistently associated with depressive symptoms. Accordingly, it has been proposed that children’s adaptation to parental separation is affected by the quality of the interparental relationship and the parent–child interaction [29].

### Findings regarding inflammation

In line with the results of earlier studies [18], we found weak evidence for the associations between ACEs and early-life inflammation. However, our analysis makes a significant contribution to the current body of evidence by showing that some associations started to emerge with adversities experienced in late childhood and adolescence (i.e., bullying and sexual abuse). This finding suggests that late childhood and adolescence might be particularly sensitive periods for the effects of ACEs on inflammation. Furthermore, it corroborates the idea that threat-related ACEs might have stronger effects on stress-related systems than other ACEs. Notably, recency effects could also be at play, but our analysis was not able to disentangle late sensitive period versus recency effects. We also found that sexual abuse was associated with higher CRP levels in boys but not in girls. This result could be explained by the fact that males are less likely to disclose sexual abuse than females and may also face additional challenges because of social attitudes and stereotypes surrounding men and masculinity [30].

Previous studies have provided evidence both for and against the association between inflammation and depression in young people [31, 32]. Recent research in ALSPAC found that CRP levels at age 9 years were not associated with depressive symptoms at age 18 [33]. In contrast, another study showed that an increasing pattern of inflammation during adolescence was associated with moderate/severe levels of depression in young adulthood [34], although this analysis was based on a small subsample of ALSPAC participants with complete data on all variables (*N*~1460). In the present study, neither the individual CRP measures nor longitudinal patterns of CRP from childhood to adolescence were related to depressive symptoms in young adulthood, both in the imputed and complete data analyses. Furthermore, in contrast with recent evidence in older adults [35], CRP levels did not mediate the associations between ACEs and depressive symptoms in young adulthood. These results suggest that the contribution of CRP levels to depression might be more prominent in adults than in young people. It is also important to note that the biological relevance of inflammation in childhood and adolescence might be different from inflammation in adulthood and later life. Developing bodies are known to be masterful at compensatory mechanisms/adaptive regulation, but little is currently known regarding developmental trajectories of immune system functioning and their relationship with sub-clinical measures of peripheral inflammation and depressive symptoms.

### Strengths and limitations

This study has several strengths, including the use of a large population sample of young people, repeated measures of various types of ACEs from the prenatal period up to adolescence in order to explore both the patterning and timing of ACEs, and repeated measures of CRP levels and depressive symptoms in childhood and young adulthood to study their longitudinal patterns over time. Most ACEs measures were prospectively collected and were complemented by retrospective information. Nevertheless, a number of limitations should be noted. First, the regional basis of the ALSPAC cohort posits some limitations in external validity when generalising findings to the national population of children, and the analytical sample was not fully representative of the entire ALSPAC cohort (Supplementary Table S45). Second, the parents of the study children reported both on events they themselves experienced and on those experienced by their child. This could introduce issues related to shared method variance, as well as bias in understanding the child’s actual experience. For instance, it has been shown that self-reported measures of childhood maltreatment have stronger associations with psychopathology compared with measures obtained through different sources (e.g., court reports), suggesting that the subjective experience of ACEs is particularly important in predicting future mental health outcomes [36]. In agreement with this finding, our sensitivity analysis shows that the association of depression with child-reported emotional neglect is larger than that with parent-reported emotional neglect. Therefore, the fact that the study children began self-reporting ACEs from age 8 onwards might explain why ACEs in late childhood/adolescence were more strongly related to depressive symptoms and inflammation than earlier ACEs. Third, this analysis only focused on a single biomarker of inflammation. However, other inflammatory markers such as IL-6 might be particularly important in understanding stress-related inflammatory responses in children and adolescents [33, 37]. In addition, recent work suggests that novel inflammatory markers, including soluble urokinase plasminogen activator receptor (suPAR) and glycoprotein acetyls (GlycA), are associated with ACEs in individuals where CRP is not [38, 39]. Future research should therefore examine alternative markers of systemic inflammation in children (e.g., IL-6, suPAR, GlycA).

## Conclusion

This study shows that ACEs from the prenatal period until adolescence are robustly associated with moderate and severe levels of depressive symptoms in young adulthood. Early interventions to prevent ACEs and ACE-related trauma might help to reduce the risk of depression across the life course. CRP levels are not consistently associated with ACEs and depression in children and young people. Therefore, interventions targeting inflammation in this population might not offer protection against depression. Future studies should consider other inflammatory markers and different biological mechanisms.

## Supplementary information

Supplementary information (SI)

## Data Availability

The code of the statistical analyses is available from the corresponding author (EI) upon request.
