# Supplementary material for: Adverse childhood experiences and severity levels of inflammation and depression from childhood to young adulthood: a longitudinal cohort study
Source: Mol Psychiatry. 2022 Mar 3;27(4):2255–63. doi: 10.1038/s41380-022-01478-x (PMC9126802; doi:10.1038/s41380-022-01478-x)
Supplement: Supplementary file 1 — Supplementary information (SI) [file 41380_2022_1478_MOESM1_ESM.docx]

## Supplemental Information (SI)

**sIntroduction**

*Hypotheses*

1. ACEs are associated with increased levels of inflammation during childhood and depressive symptoms in young adulthood, with differential associations across different individual adversities, cumulative ACEs scores, and FA-derived ACEs dimensions.
2. The associations of ACEs with inflammation and depressive symptoms differ across different early-life periods due to possible sensitive period, accumulation, or recency effects.
3. Increased levels of inflammation during childhood are associated with elevated levels of depressive symptoms in young adulthood.
4. The relationships between ACEs, inflammation, and depressive symptoms differ between girls and boys.
5. Inflammation partially mediates the associations between ACEs and depressive symptoms.

**sMethods**

**Sample**

The sample consisted of participants from the Avon Longitudinal Study of Parents and Children (ALSPAC). ALSPAC is a prospective observational cohort study investigating influences on health and development across the life course (1,2). The study recruited 14,541 pregnant women living in the Bristol area (United Kingdom) with expected delivery dates between 1^st^ April 1991 and 31^st^ December 1992. ﻿This resulted in 14,062 live-born children of whom 13,988 were alive at 1 year of age. When the oldest study children were approximately 7 years of age, the initial sample was boosted with eligible participants who had failed to join the study when it first began. As a result, ﻿the total sample, including later enrolment phases, includes 14,775 live births and 14,701 children alive at 1 year of age. Data collection took place at several time points from pregnancy up to young adulthood. The present analysis uses data from the prenatal period through to 23 years of age. We focused on adversities experienced throughout the entire childhood period (-1 to 18 years), as well as during specific early-life periods: prenatal period, 0-3 years, >3-7 years, >7-12 years, and >12-18 years. The analytical sample was defined as children with at least 10% of ACEs data across all early-life periods, one measure of inflammation, and one measure of depressive symptoms (N=3,931). A comparison of the baseline characteristics of the ALSPAC participants included in the analytical sample versus those excluded from the analysis is presented in Table S45 –Supplemental Information (SI). Study data were collected and managed using REDCap electronic data capture tools hosted at the University of Bristol (3). REDCap (Research Electronic Data Capture) is a secure, web-based software platform designed to support data capture for research studies. Written informed consent was obtained from the parents of the study children. Ethical approval was granted by the ALSPAC Ethics and Law Committee and the Local Research Ethics Committees. Consent for the collection of biological samples was obtained in accordance with the Human Tissue Act (2004). Further information regarding the study design, variables, and methods of data collection can be found on the study website. Please note that the study website contains details of all the data that is available through a fully searchable data dictionary and variable search tool (<http://www.bristol.ac.uk/alspac/researchers/our-data/>).

**Measures**

*Adverse childhood experiences (ACEs)*

ACEs from the prenatal period up to adolescence (prenatal to 18 years) were assessed repeatedly using both prospectively and retrospectively collected information, reported by the parents and/or the study children. The following adversities were included in the analysis: physical abuse, emotional abuse/neglect, sexual abuse, bullying, household violence, parental substance use problems, parental mental health problems, parental convictions, parental separation, and low parent-child bonding. We adopted an ‘extended’ ACEs approach considering not only the classic ACEs included in the first seminal ACEs investigations (i.e. abuse, neglect, and family dysfunction) (4,5), but also additional psychosocial adversities that have been shown to predict long-term mental health and well-being outcomes (6), such as poor parent-child relationships and bullying (7–9). The aforementioned ACEs also align closely with the definition proposed by McLaughlin (2016), which describes ACEs as those early-life experiences that are likely to require considerable psychological, social, or neurobiological adaptation by an average child, and that represent a deviation from the expectable environment (10). In order to improve the operationalisation of ACEs across different ALSPAC studies, each adversity was defined and measured using the approach for deriving ACEs constructs developed by ﻿Houtepen et al. (2018) (7). Overall, around 90% of all ACEs variables were measured through prospective assessments. In addition, retrospective self-report measures were included in the analysis to complement the prospective data, particularly on sensitive topics such as sexual abuse. All ACEs data measured between 0-8 years were parent-reported, and from age 8 years onwards the study children began self-reporting ACEs as well. All variables were recoded to binary (yes/no) based on the criteria described in the SI (Tables S1-S5). Table 1 shows the definitions, respondents, and time periods of the ACEs data. A more detailed overview of the exact variables, time points, methods of data collection, and dichotomisation criteria for each ACE construct can be found in Tables S1-S5.

First, for each early-life period considered in the analysis (i.e. prenatal, 0-3yrs, 3-7yrs, 7-12yrs, and 12-18yrs), a binary construct was derived for each ACE representing any exposure to the relevant adversity in the specified period. For participants with less than 50% of the responses available for one type of ACE, the binary ACE construct was coded as missing. Each early-life period included all variables representing ACEs that started and ended within the specified time frame, and also those that started a few months before or ended a few months after the specified time frame. Second, a cumulative risk score was calculated representing the total number of ACEs experienced by the participant throughout the entire childhood period and during specific early-life periods. Third, factor analysis was applied to the individual ACEs constructs to identify distinct dimensions of ACEs, namely clusters of ACEs that tend to co-occur in the sample.

*Early-life inflammation*

Non-fasting blood samples for the analysis of CRP were taken from the study children at 9, 15, and 18 years of age. After collection, the blood samples were immediately spun, frozen, and stored at -80℃. High-sensitivity CRP concentrations were measured using an automated particle-enhanced immunoturbidimetric assay (Roche, Welwyn Garden City, UK). The minimum detection limit was 0.01 mg/L (11). CRP measurements were available for 5,081 children at age 9 years, 3,488 children at age 15 years, and 3,286 children at age 18 years. Participants with CRP values > 10 mg/L (N_age9_ = 60, N_age15_ = 61, N_age18_ = 79) were excluded from the analysis, as this could reflect current infection rather than chronic inflammation (12).

*Depressive symptoms*

Self-reported depressive symptoms in young adulthood were measured using the Short Mood and Feelings Questionnaire (SMFQ) (13) when the study children were aged 18, 21, 22, and 23 years. The SMFQ includes 13 items assessing the presence of various depressive symptoms in the last two weeks (e.g. respondent felt miserable or unhappy, didn't enjoy anything at all, felt so tired he/she just sat around and did nothing). Each item was scored from 0 to 2 (0 = “Not True”, 1 = “Sometimes”, 2 = “True”), resulting in a total score ranging between 0-26. Previous work has shown that the SMFQ total score correlates highly with measures of clinical depression (14,15). Additionally, the SMFQ has been used to delineate trajectories of depressive symptoms in other studies of young people (16,17), and it has been shown to have good internal validity in the ALSPAC cohort (Cronbach’s *α* ~ 0.85) (18). The total SMFQ scores at the four time points were then used to identify distinct group-based trajectories representing longitudinal patterns of depressive symptoms during young adulthood.

*Covariates*

Possible confounders of the associations between ACEs, inflammation, and depressive symptoms were selected based on previous studies in the field. These were: sex (male, female) – also analysed as a possible effect modifier; ethnicity (white/other); whether the mother had smoked during pregnancy (no, yes); the mother’s marital status at the time of birth of the study child (never married, 1^st^ marriage, 2^nd^/3^rd^ marriage, divorced/separated, widowed); the mother’s highest educational qualification at the time of birth [certificate of secondary education (CSE), vocational qualification, ordinary (O)-level, advanced (A)-level, degree or higher qualification]; and the parents’ social class at the time of birth, based on the highest occupational level of the mother and the partner (professional, managerial, and technical, skilled non-manual, skilled manual, partly skilled, unskilled).

**Statistical analyses**

*ACEs dimensions*

Explorative factor analysis (EFA) and confirmatory factor analysis (CFA) were employed to identify distinct dimensions of adversity underlying the ACEs constructs, as described elsewhere (19). The dimensional model was validated using 5-fold cross-validation to improve its generalisability and reduce the risk of overfitting. After randomly splitting the sample into five equally sized groups, EFA with geomin rotation was conducted in the four training datasets to identify the most stable and consistent factor structure. This process was informed by inspection of scree plots and eigenvalues. CFA was then performed on the test dataset, using the EFA results to specify the number of latent factors and item loadings. Since the ACEs items were categorical, all models were fitted using the diagonally weighted least squares (WLSMV) estimator, which is specifically designed for latent variable modelling with categorical data (20). Model fit was evaluated using RMSEA (< 0.08), CFI (> 0.90), and TLI (> 0.90). The factor analysis was conducted on the overall sample of ALSPAC children with at least 10% of ACEs data available across all early-life periods (N = 12,830). In the regression analysis, the resulting ACEs dimensions were indexed by binary indicators representing the presence of any exposure to the relevant ACEs.

*Group-based trajectories of CRP and depressive symptoms*

Latent class growth analysis (LCGA) was used to identify group-based longitudinal trajectories of CRP and depressive symptoms. This method is characterised by the combination of latent growth modelling with latent class analysis (21), and it enabled us to group the study participants into distinct groups representing different levels of depressive symptoms and CRP over time. The optimal number of trajectories was identified using a stepwise approach. First, one LCGA model was fitted including a single trajectory. Second, an additional trajectory was included at each following step until the optimal number of trajectories was reached. The optimal class solution was determined using the following criteria: lower Akaike information criterion (AIC), lower Bayesian Information Criterion (BIC), every class containing more than 5% of participants, entropy value (i.e. quality of the classification model) closest to 1, and good interpretability of the latent trajectory groups, as recommended elsewhere (21). The resulting group-based trajectories were then used as outcome variables in the regression analysis.

*Causal mediation analysis*

Model-based causal mediation analysis was performed using the *Mediation* R package (22) in order to examine whether the exposure (ACEs) was indirectly associated with the outcome (depressive symptoms) through the mediator (CRP). This mediation analysis approach is embedded within the counterfactual framework of causal inference and does not make reference to any specific statistical model. Consequently, it can be applied to a wide range of data settings including logistic and mixed-effects regression models. The average causal mediation effect (ACME) (or indirect effect) is defined as follows:

*δi (e) = Yi(e,Mi(1)) −Yi(e,Mi(0)),*

for each unit *i* and each exposure status *e* = 0, 1. This equation represents the change in the outcome that would occur by changing the mediator *(M)* from the value that would result under the unexposed condition *Mi(0)* to the value that would be realised under the exposed condition *Mi(1)*, while holding exposure status constant (23). Model-based ACMEs are estimated under the sequential ignorability assumption (i.e. no unmeasured confounders affecting the relationship of the exposure with the mediator and the outcome, and the relationship between the mediator and the outcome), which can be satisfied by including the relevant confounding variables in the mediation models. Causal mediation analysis also allows for the assessment of possible variations in the ACMEs according to the baseline exposure status. This can be tested by adding an interaction term between the exposure and the mediator to the outcome model, which provides the ACMEs under the exposed and unexposed conditions.

To compute the ACMEs of inflammation in the present study, two logistic regression models were fitted for the probability of moderate-high and high-moderate CRP trajectories (vs low) (see ‘Results’) including the ACE variable and all covariates as independent variables (mediator models), and two logistic regression models were fitted for the probability of moderate and severe depression trajectories (vs low) (see ‘Results’) including the ACE variable, CRP trajectories, and all covariates as independent variables (outcome models). The mediation models also accounted for possible exposure-mediator interactions in order to assess whether the ACMEs of inflammation may take different values depending on the exposure status (i.e. no exposure vs exposure to ACEs).

**sResults**

*Descriptive statistics*

The characteristics of the study participants in the observed and imputed data are presented in Table S6a (SI file). The distribution of the observed and imputed data was similar, suggesting that the MICE analysis achieved its goals. The sample was 60% female, and 96% of the participants had a white ethnic background. Average levels of CRP were low at age 9 years (0.56 mg/L), but they increased over the following assessments. The average total score of depressive symptoms across the time points was around 6. The lowest depression score was reported at age 21 (mean 5.5), while the highest score was observed at age 23 (mean 6.7). Throughout childhood, the most commonly reported ACE was parental mental health problems (50.7%), followed by emotional abuse/neglect (37.2%), and parental separation (30.8%). The least commonly reported ACE was sexual abuse (4.8%), followed by parental convictions (7.6%). Around 87% of the sample reported at least one ACE throughout childhood, and 23.5% experienced four or more ACEs (Table S6a). Prevalence statistics of ACEs across specific early-life periods are illustrated in Figure 2 and reported in Table S6b (SI file). Parental mental health problems were one of the most commonly reported ACEs across most periods, along with emotional abuse/neglect (12-18 years) and low parent-child bonding (7-12 years). Sexual abuse and parental convictions were the least commonly reported ACEs in all early-life periods. Regarding the cumulative risk scores, the number of participants reporting 1 or more ACEs tended to increase over time. In the prenatal period, around 70% of the study children had not experienced any ACE. By contrast, only 45% of the sample did not report any ACEs during adolescence. The prevalence of physical abuse, emotional abuse/neglect, and household violence in the prenatal period, sexual abuse from 0 to 12 years, and parental convictions at 12-18 years was very low (i.e. less than 30 participants in the exposed groups). As such, the statistical significance of the associations involving these time-specific adversities could not be reliably tested. In addition, information on sexual abuse and parent-child bonding was not available in the prenatal period, data on bullying was only available from age 7 onwards, and data on household violence was not available at 12-18 years. For the prenatal period, only the effects of the ACEs cumulative score and the ACEs dimensions were tested in regression analysis, as several individual adversities had a low prevalence before birth.

*ACEs dimensions*

The results of the EFA of ACEs in the four training datasets are reported in Table S7 and the final CFA model fitted on the test dataset is illustrated in Figure S3 (SI file). The CFA model fit the test dataset very well, RMSEA=0.022, CFI=0.981, TLI=0.970. Standardised factor loadings ranged from medium (0.42) to high (0.73). All factors had good discriminant validity since their correlations were considerably lower than 0.85 (24). This model revealed two distinct dimensions of ACEs, namely *Physical/Emotional Threat* (including physical abuse, emotional abuse/neglect, and low parent-child bonding) and *Household Dysfunction* (including household violence, parental substance use problems, parental mental health problems, parental convictions, and parental separation). Sexual abuse and bullying were included in the final CFA model as standalone dimensions since in EFA they did not correlate well with any of the latent factors ­­– i.e., factor loadings in all EFA models were less than 0.3 (25) (see Table S7).

*Sensitivity analyses*

First, given the large number of tests conducted, False Discovery Rate (FDR) corrected p-values are provided for the associations of ACEs with CRP and depressive symptoms tested in the main imputed data analysis in order to account for multiple testing. Most associations between ACEs and depressive symptoms remained after FDR correction (Tables S10, S12, S14, S16, S18, S20, S22). All associations between ACEs and CRP were instead nonsignificant (Tables S9, S11, S13, S15, S17, S19, S21), except for the association of bullying (prenatal-18yrs) with high-moderate CRP levels (Table S19) and the interaction effect between sexual abuse 12-18yrs and the child’s sex on moderate-high CRP trajectories (Table S25) which were robust to adjustment for multiple testing.

Second, the associations between ACEs, CRP, and depressive symptoms and the mediation effects of CRP were retested in a restricted sample with complete data on all variables. The associations between ACEs and depression trajectories mirrored those found in the main imputed data analysis (Tables S31, S33, S35, S37, S39, S41). The cumulative ACEs score and the household dysfunction and emotional/physical threat dimensions were consistently associated with moderate and severe depressive symptoms in most early-life periods. Fewer associations were found between the individual adversities in specific early-life periods and depressive symptoms, possibly due to the reduced statistical power of the complete data analysis. As for the imputed data analysis, most associations between ACEs and the CRP trajectories were nonsignificant (Table S30, S32, S34, S36, S38, S40). The positive association between bullying and high-moderate CRP trajectories was also present in the complete data analysis, at both 7-12yrs and 12-18yrs (Table S36 and S38). A few additional associations between ACEs and CRP emerged in the complete data analysis which were not found in the imputed data analysis. However, some of these associations were positive (e.g. parental separation 3-7yrs and moderate-high CRP), while others were negative (e.g. physical abuse 3-7yrs and moderate-high CRP). The reliability of these associations is questionable owing to the unrepresentativeness and small size of the samples used in the complete data analysis. Further, as for the analysis with the imputed data, all associations between CRP and depressive symptoms were nonsignificant (Tables S42-S43), and there was no evidence for the mediating role of CRP in the associations between ACEs and depressive symptoms (Table S44).

Third, significant differences were found between the baseline characteristics of the ALSPAC participants included in the analytical sample versus those excluded from the analysis owing to missing data on the exposure or outcome variables (Table S45). Compared with non-included participants, the children included in the analytical sample were more likely to be female, they had higher family socioeconomic status, and they were slightly more likely to have a white ethnic background.

Fourth, the entropy value of the 3-class trajectory model for CRP values was relatively low (0.584). We therefore considered alternative approaches to operationalise CRP levels, including individual log-transformed CRP measures, the average CRP trajectory across the time points, and the average CRP value across the time points. In linear regression analysis, all ACEs variables were unrelated to individual measures of CRP at age 9, 15, and 18 years (Table S46). We then used growth curve mixed-effects modelling to test the associations of ACEs with the rate of change in the average CRP trajectory over time. The results (Table S47) showed that log CRP levels increased by 0.094 (95%CI: 0.088; 0.100) points at each consecutive time point, on average. In line with the results for the individual CRP measures, all ACEs variables were unrelated to both baseline CRP levels (i.e. age 9 years) and the rate of change in CRP levels over time (Table S47). Further, we tested the associations of the average CRP value across the time points with the depression trajectories (Table S27) and the individual binary depression scores at age 18, 21, and 23 years (Table S28). As for the individual CRP measures and the CRP trajectories, the average CRP value was unrelated to both the depression trajectories and the individual depression measures.

Fifth, the associations of ACEs (entire childhood period) with the CRP trajectories and those between the CRP and depression trajectories were further adjusted for BMI at age 15 years. The adjustment for BMI did not substantially change the strength of the associations of CRP with ACEs and depressive symptoms (Tables S48 and S49).

Lastly, we tested the associations of child-reported versus parent-reported emotional neglect (16 years) with depression (18 years) in order to explore potential differences in the strength of the associations between reporting sources. We found that the association with child-reported neglect (OR=1.40 [95%CI: 1.04;1.89] p=0.027) was larger than that with parent-reported neglect (OR=1.11 [95%CI: 0.93; 1.31] p=0.247) (Table S50).

**References**

1. Boyd A, Golding J, Macleod J, Lawlor DA, Fraser A, Henderson J, et al. Cohort profile: The ’Children of the 90s’-The index offspring of the avon longitudinal study of parents and children. Int J Epidemiol. 2013;42(1):111–27.

2. Fraser A, Macdonald-Wallis C, Tilling K, Boyd A, Golding J, Davey Smith G, et al. Cohort Profile: The Avon Longitudinal Study of Parents and Children: ALSPAC mothers cohort. Int J Epidemiol. 2013;42(1):97–110.

3. Harris PA, Taylor R, Thielke R, Payne J, Gonzalez N, Conde JG. Research electronic data capture (REDCap)—A metadata-driven methodology and workflow process for providing translational research informatics support. J Biomed Inform. 2009;42(2):377–81.

4. Dong M, Anda RF, Felitti VJ, Dube SR, Williamson DF, Thompson TJ, et al. The interrelatedness of multiple forms of childhood abuse, neglect, and household dysfunction. Child Abuse Negl. 2004;28(7):771–84.

5. Felitti VJ, Anda RF, Nordenberg D, Williamson DF, Spitz AM, Edwards V, et al. Relationship of Childhood Abuse and Household Dysfunction to Many of the Leading Causes of Death in Adults. Am J Prev Med. 1998;14(4):245–58.

6. Finkelhor D, Shattuck A, Turner H, Hamby S. A revised inventory of Adverse Childhood Experiences. Child Abus Negl. 2015;48:13–21.

7. Houtepen LC, Heron J, Suderman MJ, Tilling K, Howe LD. Adverse childhood experiences in the children of the Avon Longitudinal Study of Parents and Children (ALSPAC). Wellcome Open Res. 2018;3:106.

8. Yap MBH, Pilkington PD, Ryan SM, Jorm AF. Parental factors associated with depression and anxiety in young people: A systematic review and meta-analysis. J Affect Disord. 2014;156:8–23.

9. Schoeler T, Duncan L, Cecil CM, Ploubidis GB, Pingault JB. Quasi-experimental evidence on short- and long-term consequences of bullying victimization: A meta-analysis. Psychol Bull. 2018;144(12):1229–46.

10. McLaughlin KA, Sheridan MA. Beyond Cumulative Risk: A Dimensional Approach to Childhood Adversity. Curr Dir Psychol Sci. 2016;25(4):239–45.

11. Chu AL, Stochl J, Lewis G, Zammit S, Jones PB, Khandaker GM. Longitudinal association between inflammatory markers and specific symptoms of depression in a prospective birth cohort. Brain Behav Immun. 2019;76:74–81.

12. Pearson TA, Mensah GA, Alexander RW, Anderson JL, Cannon RO, Criqui M, et al. Markers of Inflammation and Cardiovascular Disease. Circulation. 2003;107(3):499–511.

13. Angold A, Costello J, Van Kämmen W, Stouthamer-Loeber M. Development of a short questionnaire for use in epidemiological studies of depression in children and adolescents: factor composition and structure across development. Int J Methods Psychiatr Res. 1996;5(4):251–62.

14. Thapar A, McGuffin P. Validity of the shortened Mood and Feelings Questionnaire in a community sample of children and adolescents: a preliminary research note. Psychiatry Res. 1998;81(2):259–68.

15. Turner N, Joinson C, Peters TJ, Wiles N, Lewis G. Validity of the Short Mood and Feelings Questionnaire in late adolescence. Psychol Assess. 2014;26(3):752–62.

16. Kingsbury M, Weeks M, MacKinnon N, Evans J, Mahedy L, Dykxhoorn J, et al. Stressful Life Events During Pregnancy and Offspring Depression: Evidence From a Prospective Cohort Study. J Am Acad Child Adolesc Psychiatry. 2016;55(8):709-716.e2.

17. Kwong ASF, López-López JA, Hammerton G, Manley D, Timpson NJ, Leckie G, et al. Genetic and Environmental Risk Factors Associated With Trajectories of Depression Symptoms From Adolescence to Young Adulthood. JAMA Netw Open. 2019;2(6):e196587.

18. Kwong ASF. Examining the longitudinal nature of depressive symptoms in the Avon Longitudinal Study of Parents and Children (ALSPAC). Wellcome Open Res. 2019;4:126.

19. Iob E, Lacey R, Steptoe A. The long-term association of adverse childhood experiences with C-reactive protein and hair cortisol: Cumulative risk versus dimensions of adversity. Brain Behav Immun. 2020;87:318–28.

20. Li C-H. Confirmatory factor analysis with ordinal data: Comparing robust maximum likelihood and diagonally weighted least squares. Behav Res Methods. 2016;48(3):936–49.

21. Herle M, Micali N, Abdulkadir M, Loos R, Bryant-Waugh R, Hübel C, et al. Identifying typical trajectories in longitudinal data: modelling strategies and interpretations. Eur J Epidemiol. 2020;

22. Tingley D, Yamamoto T, Hirose K, Keele L, Imai K. Mediation: R package for causal mediation analysis. J Stat Softw. 2014;59(5):1–38.

23. Imai K, Keele L, Tingley D. A General Approach to Causal Mediation Analysis. Psychol Methods. 2010;15(4):309–34.

24. Kenny DA. Multiple Latent Variable Models: Confirmatory Factor Analysis [Internet]. 2016.

25. Field A. Discovering statistics using SPSS. London: SAGE Publications Limited; 2013.

| Legend of Tables S1­­-S5. | |
| --- | --- |
| Legend column name: | **Explanation:** |
| *variable name* | ALSPAC variable id |
| *description* | Brief variable description |
| *datasource* | Who reported and when |
| *retrospective* | Whether the time period reported on (see column time_period) is in the past |
| *ACE* | Adverse childhood experience category |
| *time_alspac* | Time of data collection as denoted in the alspac files |
| *time_period* | Time period (in years) covered by the question, for retrospective questions can be different from time_alspac |
| *time_yrs_start* | Time in years of start reported period |
| *time_yrs_end* | Time in years of end reported period |
| *reverse_scale* | When calculating adversity, the adverse condition needs to be the highest level in the factor. For most variable this is either true (yes) or false (no), but for some variables the order of the factor must be changed. This is denoted by yes_special or no_special. |
| *recode_ACE* | If the current coding needs to be adapted, this column contains the way this should be done. `;` separates variables and `]` indicates which categories should be merged.  Thus *Always feel;Often feel;Sometimes feel];Never feel*, indicates *Never feel* remains one category while the three separate categories *Always feel, Often feel, Sometimes feel* will be merged into one category. |
| *factor_level_after_recode* | After applying recode_ACE, non-exposed on left and exposed on right of the ];. Thus *Never feel];Always feel_Often feel_Sometimes feel* indicates that if answer was *never feel* it was classified as non-exposed while answers always,often,sometimes were classified as exposed. |
| Note. Tables F1-F5 have been adapted from: *Houtepen et al. (2018), Adverse childhood experiences in the children of the Avon Longitudinal Study of Parents and Children (ALSPAC), Wellcome Open Research, 3:106.* | |

| **Table S1. ACE variables in the prenatal period.** | | | | | | | | | | | |
| --- | --- | --- | --- | --- | --- | --- | --- | --- | --- | --- | --- |
| **variable name** | **description** | **datasource** | **retrospective** | **ACE** | **time_alspac** | **time_period** | **time_yrs_start** | **time_yrs_end** | **reverse_scale** | **recode_ACE** | **factor_level_after_recode** |
| b608 | PTNR was EMOT cruel to CH since PREG | Quest_Mother | no | emotional_abuse | 18w gestation | 2nd trimester | -1 | -1 | yes | affected a lot;fairly affected;mildly affected;N effect at all];didnt happen | didnt happen];affected a lot_fairly affected_mildly affected_N effect at all |
| b370 | Edinburgh Post-natal Depression Score | Quest_Mother | no | mental_health_problems_or_suicide | 18w gestation | 2nd trimester | -1 | -1 | no | 0;1;2;3;4;5;6;7;8;9;10;11;12];13;14;15;16;17;18;19;20;21;22;23;24;25;26;27;28;30 | 0_1_2_3_4_5_6_7_8_9_10_11_12];13_14_15_16_17_18_19_20_21_22_23_24_25_26_27_28_30 |
| c600 | EPDS in YP | Quest_Mother | no | mental_health_problems_or_suicide | 32w gestation | 3rd trimester | -1 | -1 | no | 0;1;2;3;4;5;6;7;8;9;10;11;12];13;14;15;16;17;18;19;20;21;22;23;24;25;26;27;28;29 | 0_1_2_3_4_5_6_7_8_9_10_11_12];13_14_15_16_17_18_19_20_21_22_23_24_25_26_27_28_29 |
| pb260 | EPDS Score I | Quest_Partner | no | mental_health_problems_or_suicide | 18w gestation | 2nd trimester | -1 | -1 | no | 0;1;2;3;4;5;6;7;8;9;10;11;12];13;14;15;16;17;18;19;20;21;22;23;24;25;26;27 | 0_1_2_3_4_5_6_7_8_9_10_11_12];13_14_15_16_17_18_19_20_21_22_23_24_25_26_27 |
| b106 | Medication for anxiety this PREG | Quest_Mother | no | mental_health_problems_or_suicide | 18w gestation | 2nd trimester | -1 | -1 | yes | Y in 1-3 MTHS;Y 4 MTHS to now;Y both time periods];not at all | not at all];Y in 1-3 MTHS_Y 4 MTHS to now_Y both time periods |
| b107 | Medication for anxiety in 1st 3 months | Quest_Mother | no | mental_health_problems_or_suicide | 18w gestation | 2nd trimester | -1 | -1 | yes | Yes];No | No];Yes |
| b122 | Medication for depression this PREG | Quest_Mother | no | mental_health_problems_or_suicide | 18w gestation | 2nd trimester | -1 | -1 | yes | Y in 1-3 MTHS;Y 4 MTHS to now;Y both time periods];not at all | not at all];Y in 1-3 MTHS_Y 4 MTHS to now_Y both time periods |
| b123 | Medication for depression in 1st 3 months | Quest_Mother | no | mental_health_problems_or_suicide | 18w gestation | 2nd trimester | -1 | -1 | yes | Yes];No | No];Yes |
| b597 | Attempted suicide since PREG | Quest_Mother | no | mental_health_problems_or_suicide | 18w gestation | 2nd trimester | -1 | -1 | yes | affected a lot;mildly affected;N effect at all];didnt happen | didnt happen];affected a lot_mildly affected_N effect at all |
| c093 | MEDTN for anxiety in last 3MTHS | Quest_Mother | no | mental_health_problems_or_suicide | 32w gestation | 3rd trimester | -1 | -1 | yes | Y];N | N];Y |
| c101 | MEDTN for depression in last 3MTHS | Quest_Mother | no | mental_health_problems_or_suicide | 32w gestation | 3rd trimester | -1 | -1 | yes | Y];N | N];Y |
| d152 | Had bulimia | Quest_Mother | no | mental_health_problems_or_suicide | 15w gestation | 2nd trimester | -1 | -1 | yes | Yes recently];Yes in past;No never | No never_Yes in past];Yes recently |
| d169 | Had schizophrenia | Quest_Mother | no | mental_health_problems_or_suicide | 15w gestation | 2nd trimester | -1 | -1 | yes | Yes recently;Yes in past];No never | No never_Yes in past];Yes recently |
| d170 | Had anorexia nervosa | Quest_Mother | no | mental_health_problems_or_suicide | 15w gestation | 2nd trimester | -1 | -1 | yes | Yes recently];Yes in past;No never | No never_Yes in past];Yes recently |
| pa172 | Had bulimia | Quest_Partner | no | mental_health_problems_or_suicide | 12w gestation | 1st trimester | -1 | -1 | yes | Y recently];Y in past;N never | N never_Y in past];Y recently |
| pa189 | Had schizophrenia | Quest_Partner | no | mental_health_problems_or_suicide | 12w gestation | 1st trimester | -1 | -1 | yes | Y recently;Y in past];N never | N never];Y recently_Y in past |
| pa190 | Had anorexia nervosa | Quest_Partner | no | mental_health_problems_or_suicide | 12w gestation | 1st trimester | -1 | -1 | yes | Y recently];Y in past;N never | N never_Y in past];Y recently |
| pb187 | Attempted suicide since PTNR PREG | Quest_Partner | no | mental_health_problems_or_suicide | 18w gestation | 2nd trimester | -1 | -1 | yes | affected a lot;fairly affected;mildly affected;N affect at all];didnt happen | didnt happen];affected a lot_fairly affected_mildly affected_N affect at all |
| e427 | Attempted suicide since MID PREG | Quest_Mother | no | mental_health_problems_or_suicide | 8w | 2nd trimester-0.16yrs | -1 | 0.16 | yes | Affected a lot;MOD affected;Mildly affected;No effect];Did not happen | Did not happen];Affected a lot_MOD affected_Mildly affected_No effect |
| pc227 | Attempted suicide since MID PREG | Quest_Partner | no | mental_health_problems_or_suicide | 8w | 2nd trimester-0.16yrs | -1 | 0.16 | yes | affected a lot;MOD affected;Mildly affected;No effect];Did not happen | Did not happen];affected a lot_MOD affected_Mildly affected_No effect |
| b598 | Convicted of an offence since PREG | Quest_Mother | no | parent_convicted_offence | 18w gestation | 2nd trimester | -1 | -1 | yes | affected a lot;fairly affected;mildly affected;N effect at all];didnt happen | didnt happen];affected a lot_fairly affected_mildly affected_N effect at all |
| pb188a | Convicted of offence since PTNR PREG,Y/N | Quest_Partner | no | parent_convicted_offence | 18w gestation | 2nd trimester | -1 | -1 | yes | Yes];No | No];Yes |
| e428 | Convicted since MID PREG | Quest_Mother | no | parent_convicted_offence | 8w | 0.16yrs | -1 | 0.16 | yes | Affected a lot;MOD affected;Mildly affected;No effect];Did not happen | Did not happen];Affected a lot_MOD affected_Mildly affected_No effect |
| pc228a | Convicted since MID PREG, Y/N | Quest_Partner | no | parent_convicted_offence | 8w | 0.16yrs | -1 | 0.16 | yes | Yes];No | No];Yes |
| b578 | Divorced since PREG | Quest_Mother | no | parental_separation | 18w gestation | 2nd trimester | -1 | -1 | yes | affected a lot;fairly affected;mildly affected;N effect at all];didnt happen | didnt happen];affected a lot_fairly affected_mildly affected_N effect at all |
| b587 | Separated since PREG | Quest_Mother | no | parental_separation | 18w gestation | 2nd trimester | -1 | -1 | yes | affected a lot;fairly affected;mildly affected;N effect at all];didnt happen | didnt happen];affected a lot_fairly affected_mildly affected_N effect at all |
| pb168 | Divorced since PTNR PREG | Quest_Partner | no | parental_separation | 18w gestation | 2nd trimester | -1 | -1 | yes | affected a lot;fairly affected;mildly affected;N affect at all];didnt happen | didnt happen];affected a lot_fairly affected_mildly affected_N affect at all |
| pb177 | Separated since PTNR PREG | Quest_Partner | no | parental_separation | 18w gestation | 2nd trimester | -1 | -1 | yes | affected a lot;fairly affected;mildly affected;N affect at all];didnt happen | didnt happen];affected a lot_fairly affected_mildly affected_N affect at all |
| e408 | Divorced since MID PREG | Quest_Mother | no | parental_separation | 8w | 2nd trimester-0.16yrs | -1 | 0.16 | yes | Affected a lot;MOD affected;Mildly affected;No effect];Did not happen | Did not happen];Affected a lot_MOD affected_Mildly affected_No effect |
| e417 | Separated since MID PREG | Quest_Mother | no | parental_separation | 8w | 2nd trimester-0.16yrs | -1 | 0.16 | yes | Affected a lot;MOD affected;Mildly affected;No effect];Did not happen | Did not happen];Affected a lot_MOD affected_Mildly affected_No effect |
| pc208 | Divorced since MID PREG | Quest_Partner | no | parental_separation | 8w | 2nd trimester-0.16yrs | -1 | 0.16 | yes | affected a lot;MOD affected;Mildly affected;No effect];Did not happen | Did not happen];affected a lot_MOD affected_Mildly affected_No effect |
| pc217 | You & PTNR separated since MID PREG | Quest_Partner | no | parental_separation | 8w | 2nd trimester-0.16yrs | -1 | 0.16 | yes | affected a lot;MOD affected;Mildly affected;No effect];Did not happen | Did not happen];affected a lot_MOD affected_Mildly affected_No effect |
| pc226 | PTNR physical-cruel to CH since MID PREG | Quest_Partner | no | physical_abuse | 8w | 2nd trimester-0.16yrs | -1 | 0.16 | yes | affected a lot;MOD affected;Mildly affected;No effect];Did not happen | Did not happen];affected a lot_MOD affected_Mildly affected_No effect |
| b701 | Smoked cannabis in 1-3MTHS of PREG | Quest_Mother | yes | substance_household | 18w gestation | 2nd trimester | -1 | -1 | yes | everyday];2-4 times PWK;once PWK;<once PWK;not at all | 2-4 times PWK_once PWK_<once PWK_not at all];everyday |
| b702 | Smoked cannabis >3MTHS PREG | Quest_Mother | no | substance_household | 18w gestation | 2nd trimester | -1 | -1 | yes | everyday];2-4 times PWK;once PWK;<once PWK;not at all | 2-4 times PWK_once PWK_<once PWK_not at all];everyday |
| b714 | Hard drugs | Quest_Mother | no | substance_household | 18w gestation | 2nd trimester | -1 | -1 | yes | yes];no | no];yes |
| d167 | Had drug addiction | Quest_Mother | no | substance_household | 15w gestation | 2nd trimester | -1 | -1 | yes | Yes recently];Yes in past;No never | Yes in past_No never];Yes recently |
| d168 | Had alcoholism | Quest_Mother | no | substance_household | 15w gestation | 2nd trimester | -1 | -1 | yes | Yes recently];Yes in past;No never | Yes in past_No never];Yes recently |
| pa187 | Had drug addiction | Quest_Partner | no | substance_household | 12w gestation | 1st trimester | -1 | -1 | yes | Y recently];Y in past;N never | Y in past_N never];Y recently |
| pa188 | Had alcoholism | Quest_Partner | no | substance_household | 12w gestation | 1st trimester | -1 | -1 | yes | Y recently];Y in past;N never | Y in past_N never];Y recently |
| pb098 | Hard drugs | Quest_Partner | no | substance_household | 18w gestation | 2nd trimester | -1 | -1 | yes | Yes];No | No];Yes |
| e190 | FREQ of ganja use in last 2MTHS of PREG | Quest_Mother | yes | substance_household | 8w | 3rd trimester-0yrs | -1 | 0 | yes | Daily];2-4 PWK;Once PWK;<once PWK;Not at all | 2-4 PWK_Once PWK_<once PWK_Not at all];Daily |
| e203 | Hard drug in last 2 months | Quest_Mother | no | substance_household | 8w | 3rd trimester-0yrs | -1 | 0 | yes | Yes];No | No];Yes |
| pc222 | PTNR physically hurt you since MID PREG | Quest_Partner | no | violence_between_parents | 8w | 2nd trimester-0.16yrs | -1 | 0.16 | yes | affected a lot;MOD affected;Mildly affected];No effect;Did not happen | Did not happen];affected a lot_MOD affected_Mildly affected_No effect |

| **Table S2. ACE variables in early childhood (0-3 yrs).** | | | | | | | | | | | |
| --- | --- | --- | --- | --- | --- | --- | --- | --- | --- | --- | --- |
| **variable name** | **description** | **datasource** | **retrospective** | **ACE** | **time_alspac** | **time_period** | **time_yrs_start** | **time_yrs_end** | **reverse_scale** | **recode_ACE** | **factor_level_after_recode** |
| f257 | PTNR EMOT cruel to CHDR >CH born | Quest_Mother | no | emotional_abuse | 8m | 0-0.67yrs | 0 | 0.67 | yes | Y much affected;Y MOD affected;Y mildly affected;Y but N effect];N did not happen | N did not happen];Y much affected_Y MOD affected_Y mildly affected_Y but N effect |
| f258 | MUM EMOT cruel to CHDR >CH born | Quest_Mother | no | emotional_abuse | 8m | 0-0.67yrs | 0 | 0.67 | yes | Y much affected;Y MOD affected;Y mildly affected;Y but N effect];N did not happen | N did not happen];Y much affected_Y MOD affected_Y mildly affected_Y but N effect |
| pd257 | Ptnr Emotionally Cruel To Children | Quest_Partner | no | emotional_abuse | 8m | 0-0.67yrs | 0 | 0.67 | yes | Yes Affected A Lot;Yes Mod Affected;Yes Mildly Affected;Yes Did Not Affect];No Did Not Happen | No Did Not Happen];Yes Affected A Lot_Yes Mod Affected_Yes Mildly Affected_Yes Did Not Affect |
| pd258 | Self Emotionally Cruel To Children | Quest_Partner | no | emotional_abuse | 8m | 0-0.67yrs | 0 | 0.67 | yes | Yes Affected A Lot;Yes Mod Affected;Yes Mildly Affected;Yes Did Not Affect];No Did Not Happen | No Did Not Happen];Yes Affected A Lot_Yes Mod Affected_Yes Mildly Affected_Yes Did Not Affect |
| pc236 | PTNR EMOT cruel to CH | Quest_Partner | no | emotional_abuse | 8w | 0.16yrs | 0.16 | 0.16 | yes | affected a lot;MOD affected;Mildly affected;No effect];Did not happen | Did not happen];affected a lot_MOD affected_Mildly affected_No effect |
| g337 | Partner emotionally cruel to children >CH8MTHs | Quest_Mother | no | emotional_abuse | 1yrs9m | 0.67-2yrs | 0.67 | 2 | yes | Yes Big Effect;Yes Some Effect;Yes Mild Effect;Yes No Effect];Did Not Happen | Did Not Happen];Yes Big Effect_Yes Some Effect_Yes Mild Effect_Yes No Effect |
| g338 | Mum emotionally cruel to children >CH18MTHs | Quest_Mother | no | emotional_abuse | 1yrs9m | 0.67-2yrs | 0.67 | 2 | yes | Yes Big Effect;Yes Some Effect;Yes Mild Effect;Yes No Effect];Did Not Happen | Did Not Happen];Yes Big Effect_Yes Some Effect_Yes Mild Effect_Yes No Effect |
| pe337 | Partner Emotionally Cruel To Child | Quest_Partner | no | emotional_abuse | 1yrs9m | 0.67-2yrs | 0.67 | 2 | yes | Yes & Affected Lots;Yes Mod Affected;Yes Mildly Affected;Yes Did Not Affect];No Did Not Happen | No Did Not Happen];Yes & Affected Lots_Yes Mod Affected_Yes Mildly Affected_Yes Did Not Affect |
| pe338 | Self Emotionally Cruel To Child | Quest_Partner | no | emotional_abuse | 1yrs9m | 0.67-2yrs | 0.67 | 2 | yes | Yes & Affected Lots;Yes Mod Affected;Yes Mildly Affected;Yes Did Not Affect];No Did Not Happen | No Did Not Happen];Yes & Affected Lots_Yes Mod Affected_Yes Mildly Affected_Yes Did Not Affect |
| h247 | E38: Whether partner was emotionally cruel to children since study child was 18 months old and effect this had | Quest_Mother | no | emotional_abuse | 2yrs9m | 1.5-3yrs | 1.5 | 3 | yes | yes had big effect;yes medium effect;yes mild effect;yes but no effect];didnt happen | didnt happen];yes had big effect_yes medium effect_yes mild effect_yes but no effect |
| h248 | E39: Whether mum was emotionally cruel to children since study child was 18 months old and effect this had | Quest_Mother | no | emotional_abuse | 2yrs9m | 1.5-3yrs | 1.5 | 3 | yes | yes had big effect;yes medium effect;yes mild effect;yes but no effect];didnt happen | didnt happen];yes had big effect_yes medium effect_yes mild effect_yes but no effect |
| pf5036 | E37: Partner's partner was emotionally cruel to their children since study child was 18 months old | Quest_Partner | no | emotional_abuse | 2yrs9m | 1.5-3yrs | 1.5 | 3 | yes | Yes & affected me a lot;Yes, moderately affected;Yes, mildly affected;Yes, but did not affect me at all];No did not happen | No did not happen];Yes & affected me a lot_Yes, moderately affected_Yes, mildly affected_Yes, but did not affect me at all |
| pf5037 | E38: Partner was emotionally cruel to their children since study child was 18 months old | Quest_Partner | no | emotional_abuse | 2yrs9m | 1.5-3yrs | 1.5 | 3 | yes | Yes & affected me a lot;Yes, moderately affected;Yes, mildly affected;Yes, but did not affect me at all];No did not happen | No did not happen];Yes & affected me a lot_Yes, moderately affected_Yes, mildly affected_Yes, but did not affect me at all |
| e326 | FREQ of anti-depressant use since birth | Quest_Mother | no | mental_health_problems_or_suicide | 8w | 0-0.16yrs | 0 | 0.16 | yes | Almost daily;SMTS];Not at all | Not at all];Almost daily_SMTS |
| f063 | Anti-depressant use since CH born | Quest_Mother | no | mental_health_problems_or_suicide | 8m | 0-0.67yrs | 0 | 0.67 | yes | Daily;Often;SMTS];Not at all | Not at all];Daily_Often_SMTS |
| f248 | Attempted suicide > CH born | Quest_Mother | no | mental_health_problems_or_suicide | 8m | 0-0.67yrs | 0 | 0.67 | yes | Y much affected;Y MOD affected;Y mildly affected;Y but N effect];N did not happen | N did not happen];Y much affected_Y MOD affected_Y mildly affected_Y but N effect |
| f526 | PTNR had schizophrenia >CH born | Quest_Mother | no | mental_health_problems_or_suicide | 8m | 0-0.67yrs | 0 | 0.67 | yes | Y saw DR;Y didnt see DR];Never | Never];Y saw DR_Y didnt see DR |
| pd063 | Used Pills for Depression Since Baby Born | Quest_Partner | no | mental_health_problems_or_suicide | 8m | 0-0.67yrs | 0 | 0.67 | yes | Every Day;Often;Sometimes];Not At All | Not At All];Every Day_Often_Sometimes |
| pd248 | Attempted Suicide Since Baby Born | Quest_Partner | no | mental_health_problems_or_suicide | 8m | 0-0.67yrs | 0 | 0.67 | yes | Yes Affected A Lot;Yes Mildly Affected;Yes Did Not Affect];No Did Not Happen | No Did Not Happen];Yes Affected A Lot_Yes Mildly Affected_Yes Did Not Affect |
| e391 | EPDS | Quest_Mother | no | mental_health_problems_or_suicide | 8w | 0.16yrs | 0.16 | 0.16 | no | 0;1;2;3;4;5;6;7;8;9;10;11;12];13;14;15;16;17;18;19;20;21;22;23;24;25;26;27;28 | 0_1_2_3_4_5_6_7_8_9_10_11_12];13_14_15_16_17_18_19_20_21_22_23_24_25_26_27_28 |
| pc102 | EPDS Score I | Quest_Partner | no | mental_health_problems_or_suicide | 8w | 0.16yrs | 0.16 | 0.16 | no | 0;1;2;3;4;5;6;7;8;9;10;11;12];13;14;15;16;17;18;19;20;21;22;23;26;27 | 0_1_2_3_4_5_6_7_8_9_10_11_12];13_14_15_16_17_18_19_20_21_22_23_26_27 |
| f200 | Edinburgh Post-natal Depression Score | Quest_Mother | no | mental_health_problems_or_suicide | 8m | 0.67yrs | 0.67 | 0.67 | no | 0;1;2;3;4;5;6;7;8;9;10;11;12];13;14;15;16;17;18;19;20;21;22;23;24;25;26;27;28;29 | 0_1_2_3_4_5_6_7_8_9_10_11_12];13_14_15_16_17_18_19_20_21_22_23_24_25_26_27_28_29 |
| pd200 | Edinburgh Post-natal Depression Score | Quest_Partner | no | mental_health_problems_or_suicide | 8m | 0.67yrs | 0.67 | 0.67 | no | 0;1;2;3;4;5;6;7;8;9;10;11;12];13;14;15;16;17;18;19;20;21;22;24 | 0_1_2_3_4_5_6_7_8_9_10_11_12];13_14_15_16_17_18_19_20_21_22_24 |
| g049 | Mum had depression pills >CH8MTHs | Quest_Mother | no | mental_health_problems_or_suicide | 1yrs9m | 0.67-2yrs | 0.67 | 2 | yes | Every day;Often;Sometimes];Not at all | Not at all];Every day_Often_Sometimes |
| g328 | Mum attempted suicide >CH8MTHs | Quest_Mother | no | mental_health_problems_or_suicide | 1yrs9m | 0.67-2yrs | 0.67 | 2 | yes | Yes Big Effect;Yes Some Effect;Yes Mild Effect;Yes No Effect];Did Not Happen | Did Not Happen];Yes Big Effect_Yes Some Effect_Yes Mild Effect_Yes No Effect |
| g612 | Partner had schizophrenia >CH8MTHs | Quest_Mother | no | mental_health_problems_or_suicide | 1yrs9m | 0.67-2yrs | 0.67 | 2 | yes | Yes saw Doctor;yes did not see Dr];No Not at all | No Not at all];Yes saw Doctor_yes did not see Dr |
| pe020 | Anxiety Since Child > 8 Months | Quest_Partner | no | mental_health_problems_or_suicide | 1yrs9m | 0.67-2yrs | 0.67 | 2 | yes | Yes Consulted Dr];Yes Not Consult Dr;No | Yes Not Consult Dr_No];Yes Consulted Dr |
| pe064 | Taken Antidepressants CH > 8 Months | Quest_Partner | no | mental_health_problems_or_suicide | 1yrs9m | 0.67-2yrs | 0.67 | 2 | yes | Every Day;Often;Sometimes;Rarely];Never | Never];Every Day_Often_Sometimes_Rarely |
| h039 | A3e: Frequency Mum has taken pills for depression since study child was 18 months old | Quest_Mother | no | mental_health_problems_or_suicide | 2yrs9m | 1.5-3yrs | 1.5 | 3 | yes | Every Day;often;smts];not at all | not at all];Every Day_often_smts |
| h238 | E29: Whether mum attempted suicide since study child was 18 months old and effect this had | Quest_Mother | no | mental_health_problems_or_suicide | 2yrs9m | 1.5-3yrs | 1.5 | 3 | yes | yes had big effect;yes medium effect;yes mild effect;yes but no effect];didnt happen | didnt happen];yes had big effect_yes medium effect_yes mild effect_yes but no effect |
| h497 | H3l: Partner had schizophrenia since study child was 18 months old | Quest_Mother | no | mental_health_problems_or_suicide | 2yrs9m | 1.5-3yrs | 1.5 | 3 | yes | Yes, saw doctor;Yes, did not see doctor];No, not at all | No, not at all];Yes, saw doctor_Yes, did not see doctor |
| pf5028 | E29: Partner attempted suicide since study child was 18 months old | Quest_Partner | no | mental_health_problems_or_suicide | 2yrs9m | 1.5-3yrs | 1.5 | 3 | yes | Yes & affected me a lot;Yes, but did not affect me at all];No did not happen | No did not happen];Yes & affected me a lot_Yes, but did not affect me at all |
| g290 | Edinburgh Post-natal Depression Score | Quest_Mother | no | mental_health_problems_or_suicide | 1yrs9m | 2yrs | 2 | 2 | no | 0;1;2;3;4;5;6;7;8;9;10;11;12];13;14;15;16;17;18;19;20;21;22;23;24;25;26;27;28;29;30 | 0_1_2_3_4_5_6_7_8_9_10_11_12];13_14_15_16_17_18_19_20_21_22_23_24_25_26_27_28_29_30 |
| pe290 | EPDS Score I | Quest_Partner | no | mental_health_problems_or_suicide | 1yrs9m | 2yrs | 2 | 2 | no | 0;1;2;3;4;5;6;7;8;9;10;11;12];13;14;15;16;17;18;19;20;21;22;23;24;25;26;27 | 0_1_2_3_4_5_6_7_8_9_10_11_12];13_14_15_16_17_18_19_20_21_22_23_24_25_26_27 |
| pe328 | Attempted Suicide | Quest_Partner | no | mental_health_problems_or_suicide | 1yrs9m | 2yrs | 2 | 2 | yes | Yes & Affected Lots;Yes Mod Affected;Yes Mildly Affected;Yes Did Not Affect];No Did Not Happen | No Did Not Happen];Yes & Affected Lots_Yes Mod Affected_Yes Mildly Affected_Yes Did Not Affect |
| h200a | DV: Edinburgh postnatal depression scale score (complete cases) | Quest_Mother | no | mental_health_problems_or_suicide | 2yrs9m | 3yrs | 3 | 3 | no | 0;1;2;3;4;5;6;7;8;9;10;11;12];13;14;15;16;17;18;19;20;21;22;23;24;25;26;27;28;30 | 0_1_2_3_4_5_6_7_8_9_10_11_12];13_14_15_16_17_18_19_20_21_22_23_24_25_26_27_28_30 |
| g634 | Partner seems very close to child | Quest_Mother | no | parent_child_bond | 1yrs9m | 2yrs | 2 | 2 | no | Always feel;Sometimes feel];Never feel | Always feel_Sometimes feel];Never feel |
| h514 | H4e: Mum feels partner is close to child | Quest_Mother | no | parent_child_bond | 2yrs9m | 3yrs | 3 | 3 | no | Feel always;Feel sometimes];Never feel | Feel always_Feel sometimes];Never feel |
| f249a | Court conviction | Quest_Mother | no | parent_convicted_offence | 8m | 0.67yrs | 0.67 | 0.67 | yes | Yes];No | No];Yes |
| pd249a | Convicted of Offence Since Baby Born | Quest_Partner | no | parent_convicted_offence | 8m | 0.67yrs | 0.67 | 0.67 | yes | Yes];No | No];Yes |
| g329 | Mum convicted of offence >CH8MTHs | Quest_Mother | no | parent_convicted_offence | 1yrs9m | 0.67-2yrs | 0.67 | 2 | yes | Yes Big Effect;Yes Some Effect;Yes Mild Effect;Yes No Effect];Did Not Happen | Did Not Happen];Yes Big Effect_Yes Some Effect_Yes Mild Effect_Yes No Effect |
| g329a | Mum convicted of offence >CH8MTHs | Quest_Mother | no | parent_convicted_offence | 1yrs9m | 0.67-2yrs | 0.67 | 2 | yes | Yes];No | No];Yes |
| pe329a | Convicted of Offence Y/N | Quest_Partner | no | parent_convicted_offence | 1yrs9m | 0.67-2yrs | 0.67 | 2 | yes | Yes];No | No];Yes |
| h239 | E30: Whether mum was convicted of an offence since study child was 18 months old and effect this had | Quest_Mother | no | parent_convicted_offence | 2yrs9m | 1.5-3yrs | 1.5 | 3 | yes | yes had big effect;yes medium effect;yes mild effect;yes but no effect];didnt happen | didnt happen];yes had big effect_yes medium effect_yes mild effect_yes but no effect |
| pf5029 | E30: Partner was convicted of an offence since study child was 18 months old | Quest_Partner | no | parent_convicted_offence | 2yrs9m | 1.5-3yrs | 1.5 | 3 | yes | Yes & affected me a lot;Yes, moderately affected;Yes, mildly affected;Yes, but did not affect me at all];No did not happen | No did not happen];Yes & affected me a lot_Yes, moderately affected_Yes, mildly affected_Yes, but did not affect me at all |
| f228 | Divorce >CH born | Quest_Mother | no | parental_separation | 8m | 0-0.67yrs | 0 | 0.67 | yes | Y much affected;Y MOD affected;Y mildly affected;Y but N effect];N did not happen | N did not happen];Y much affected_Y MOD affected_Y mildly affected_Y but N effect |
| f237 | Separation from PTNR >CH born | Quest_Mother | no | parental_separation | 8m | 0-0.67yrs | 0 | 0.67 | yes | Y much affected;Y MOD affected;Y mildly affected;Y but N effect];N did not happen | N did not happen];Y much affected_Y MOD affected_Y mildly affected_Y but N effect |
| pd228 | Divorced Since Baby Born | Quest_Partner | no | parental_separation | 8m | 0-0.67yrs | 0 | 0.67 | yes | Yes Affected A Lot;Yes Mod Affected;Yes Mildly Affected;Yes Did Not Affect];No Did Not Happen | No Did Not Happen];Yes Affected A Lot_Yes Mod Affected_Yes Mildly Affected_Yes Did Not Affect |
| pd237 | Separated Since Baby Born | Quest_Partner | no | parental_separation | 8m | 0-0.67yrs | 0 | 0.67 | yes | Yes Affected A Lot;Yes Mod Affected;Yes Mildly Affected;Yes Did Not Affect];No Did Not Happen | No Did Not Happen];Yes Affected A Lot_Yes Mod Affected_Yes Mildly Affected_Yes Did Not Affect |
| g308 | Mum divorced >CH8MTHs | Quest_Mother | no | parental_separation | 1yrs9m | 0.67-2yrs | 0.67 | 2 | yes | Yes Big Effect;Yes Some Effect;Yes Mild Effect;Yes No Effect];Did Not Happen | Did Not Happen];Yes Big Effect_Yes Some Effect_Yes Mild Effect_Yes No Effect |
| g317 | Mum and partner separated >CH8MTHs | Quest_Mother | no | parental_separation | 1yrs9m | 0.67-2yrs | 0.67 | 2 | yes | Yes Big Effect;Yes Some Effect;Yes Mild Effect;Yes No Effect];Did Not Happen | Did Not Happen];Yes Big Effect_Yes Some Effect_Yes Mild Effect_Yes No Effect |
| pe308 | Divorced | Quest_Partner | no | parental_separation | 1yrs9m | 0.67-2yrs | 0.67 | 2 | yes | Yes & Affected Lots;Yes Mildly Affected;Yes Did Not Affect];No Did Not Happen | No Did Not Happen];Yes & Affected Lots_Yes Mildly Affected_Yes Did Not Affect |
| pe317 | Separated From Partner | Quest_Partner | no | parental_separation | 1yrs9m | 0.67-2yrs | 0.67 | 2 | yes | Yes & Affected Lots;Yes Mod Affected;Yes Mildly Affected;Yes Did Not Affect];No Did Not Happen | No Did Not Happen];Yes & Affected Lots_Yes Mod Affected_Yes Mildly Affected_Yes Did Not Affect |
| h218 | E9: Whether mum got divorced since study child was 18 months old and effect this had | Quest_Mother | no | parental_separation | 2yrs9m | 1.5-3yrs | 1.5 | 3 | yes | yes had big effect;yes medium effect;yes mild effect;yes but no effect];didnt happen | didnt happen];yes had big effect_yes medium effect_yes mild effect_yes but no effect |
| h227 | E18: Whether mum and partner separated since study child was 18 months old and effect this had | Quest_Mother | no | parental_separation | 2yrs9m | 1.5-3yrs | 1.5 | 3 | yes | yes had big effect;yes medium effect;yes mild effect;yes but no effect];didnt happen | didnt happen];yes had big effect_yes medium effect_yes mild effect_yes but no effect |
| pf5008 | E9: Partner was divorced since study child was 18 months old | Quest_Partner | no | parental_separation | 2yrs9m | 1.5-3yrs | 1.5 | 3 | yes | Yes & affected me a lot;Yes, moderately affected;Yes, mildly affected;Yes, but did not affect me at all];No did not happen | No did not happen];Yes & affected me a lot_Yes, moderately affected_Yes, mildly affected_Yes, but did not affect me at all |
| pf5017 | E18: Partner and partner's partner have separated since study child was 18 months old | Quest_Partner | no | parental_separation | 2yrs9m | 1.5-3yrs | 1.5 | 3 | yes | Yes & affected me a lot;Yes, moderately affected;Yes, mildly affected;Yes, but did not affect me at all];No did not happen | No did not happen];Yes & affected me a lot_Yes, moderately affected_Yes, mildly affected_Yes, but did not affect me at all |
| f246 | PTNR physically cruel to CHDR >CH born | Quest_Mother | no | physical_abuse | 8m | 0-0.67yrs | 0 | 0.67 | yes | Y much affected;Y MOD affected;Y mildly affected;Y but N effect];N did not happen | N did not happen];Y much affected_Y MOD affected_Y mildly affected_Y but N effect |
| f247 | MUM physically cruel to CHDR >CH born | Quest_Mother | no | physical_abuse | 8m | 0-0.67yrs | 0 | 0.67 | yes | Y much affected;Y MOD affected;Y mildly affected;Y but N effect];N did not happen | N did not happen];Y much affected_Y MOD affected_Y mildly affected_Y but N effect |
| pd246 | Ptnr Physically Cruel To Children | Quest_Partner | no | physical_abuse | 8m | 0.67yrs | 0.67 | 0.67 | yes | Yes Affected A Lot;Yes Mod Affected;Yes Mildly Affected;Yes Did Not Affect];No Did Not Happen | No Did Not Happen];Yes Affected A Lot_Yes Mod Affected_Yes Mildly Affected_Yes Did Not Affect |
| pd247 | Self Physically Cruel To Children | Quest_Partner | no | physical_abuse | 8m | 0.67yrs | 0.67 | 0.67 | yes | Yes Affected A Lot;Yes Mod Affected;Yes Mildly Affected;Yes Did Not Affect];No Did Not Happen | No Did Not Happen];Yes Affected A Lot_Yes Mod Affected_Yes Mildly Affected_Yes Did Not Affect |
| g326 | Partner physically cruel to children >CH8MTHs | Quest_Mother | no | physical_abuse | 1yrs9m | 0.67-2yrs | 0.67 | 2 | yes | Yes Big Effect;Yes Some Effect;Yes Mild Effect;Yes No Effect];Did Not Happen | Did Not Happen];Yes Big Effect_Yes Some Effect_Yes Mild Effect_Yes No Effect |
| g327 | Mum physically cruel to children >CH8MTHs | Quest_Mother | no | physical_abuse | 1yrs9m | 0.67-2yrs | 0.67 | 2 | yes | Yes Big Effect;Yes Some Effect;Yes Mild Effect;Yes No Effect];Did Not Happen | Did Not Happen];Yes Big Effect_Yes Some Effect_Yes Mild Effect_Yes No Effect |
| pe326 | Partner Physically Cruel to Child | Quest_Partner | no | physical_abuse | 1yrs9m | 0.67-2yrs | 0.67 | 2 | yes | Yes & Affected Lots;Yes Mod Affected;Yes Mildly Affected;Yes Did Not Affect];No Did Not Happen | No Did Not Happen];Yes & Affected Lots_Yes Mod Affected_Yes Mildly Affected_Yes Did Not Affect |
| pe327 | Self Physically Cruel to Child | Quest_Partner | no | physical_abuse | 1yrs9m | 0.67-2yrs | 0.67 | 2 | yes | Yes & Affected Lots;Yes Mod Affected;Yes Mildly Affected;Yes Did Not Affect];No Did Not Happen | No Did Not Happen];Yes & Affected Lots_Yes Mod Affected_Yes Mildly Affected_Yes Did Not Affect |
| h236 | E27: Whether partner was physically cruel to children since study child was 18 months old and effect this had | Quest_Mother | no | physical_abuse | 2yrs9m | 1.5-3yrs | 1.5 | 3 | yes | yes had big effect;yes medium effect;yes mild effect;yes but no effect];didnt happen | didnt happen];yes had big effect_yes medium effect_yes mild effect_yes but no effect |
| h237 | E28: Whether mum was physically cruel to children since study child was 18 months old and effect this had | Quest_Mother | no | physical_abuse | 2yrs9m | 1.5-3yrs | 1.5 | 3 | yes | yes had big effect;yes medium effect;yes mild effect;yes but no effect];didnt happen | didnt happen];yes had big effect_yes medium effect_yes mild effect_yes but no effect |
| pf5026 | E27: Partner's partner was physically cruel to their children since study child was 18 months old | Quest_Partner | no | physical_abuse | 2yrs9m | 1.5-3yrs | 1.5 | 3 | yes | Yes & affected me a lot;Yes, moderately affected;Yes, mildly affected;Yes, but did not affect me at all];No did not happen | No did not happen];Yes & affected me a lot_Yes, moderately affected_Yes, mildly affected_Yes, but did not affect me at all |
| pf5027 | E28: Partner was physically cruel to their children since study child was 18 months old | Quest_Partner | no | physical_abuse | 2yrs9m | 1.5-3yrs | 1.5 | 3 | yes | Yes & affected me a lot;Yes, moderately affected;Yes, mildly affected;Yes, but did not affect me at all];No did not happen | No did not happen];Yes & affected me a lot_Yes, moderately affected_Yes, mildly affected_Yes, but did not affect me at all |
| kd505a | CH Sexually Abused > 6 MTHS | Quest_Child Based | no | sexual_abuse | 18m | 0.5-1.5yrs | 0.5 | 1.5 | yes | Yes & CH Very Upset;Yes & CH Quite Upset;Yes & CH Bit Upset];Did Not Happen | Did Not Happen];Yes & CH Very Upset_Yes & CH Quite Upset_Yes & CH Bit Upset |
| kf455a | Child sexually abused > 18 months, Y/N | Quest_Child Based | no | sexual_abuse | 30m | 1.5-2.5yrs | 1.5 | 2.5 | yes | Yes];No | No];Yes |
| e192 | FREQ of ganja use since birth | Quest_Mother | no | substance_household | 8w | 0-0.16yrs | 0 | 0.16 | yes | Daily];2-4 PWK;Once PWK;<once PWK;Not at all | 2-4 PWK_Once PWK_<once PWK_Not at all];Daily |
| e213 | Hard drug use since delivery | Quest_Mother | no | substance_household | 8w | 0-0.16yrs | 0 | 0.16 | yes | Yes];No | No];Yes |
| pc266 | FREQ cannabis smoked since birth | Quest_Partner | no | substance_household | 8w | 0-0.16yrs | 0 | 0.16 | yes | Daily];2-4 times PWK;Once PWK;<once PWK;Not at all | 2-4 times PWK_Once PWK_<once PWK_Not at all];Daily |
| f061 | Cannabis use since CH born | Quest_Mother | no | substance_household | 8m | 0-0.67yrs | 0 | 0.67 | yes | Daily];Often;SMTS;Not at all | Often_SMTS_Not at all];Daily |
| f067 | Amphetamine use since CH born | Quest_Mother | no | substance_household | 8m | 0-0.67yrs | 0 | 0.67 | yes | Daily;Often;SMTS];Not at all | Not at all];Daily_Often_SMTS |
| f069 | Opiate or cocaine use since CH born | Quest_Mother | no | substance_household | 8m | 0-0.67yrs | 0 | 0.67 | yes | Daily;Often;SMTS];Not at all | Not at all];Daily_Often_SMTS |
| f527 | PTNR had alcoholism >CH born | Quest_Mother | no | substance_household | 8m | 0-0.67yrs | 0 | 0.67 | yes | Y saw DR];Y didnt see DR;Never | Y didnt see DR_Never];Y saw DR |
| pd061 | Used Cannabis/Marijuana Since Baby Born | Quest_Partner | no | substance_household | 8m | 0-0.67yrs | 0 | 0.67 | yes | Every Day];Often;Sometimes;Not At All | Often_Sometimes_Not At All];Every Day |
| pd066 | Used Amphetamines Since Baby Born | Quest_Partner | no | substance_household | 8m | 0-0.67yrs | 0 | 0.67 | yes | Often;Sometimes];Not At All | Not At All];Often_Sometimes |
| pd067 | Used Heroin, Cocaine Since Baby Born | Quest_Partner | no | substance_household | 8m | 0-0.67yrs | 0 | 0.67 | yes | Every Day;Often;Sometimes];Not At All | Not At All];Every Day_Often_Sometimes |
| pc276 | Hard drugs | Quest_Partner | no | substance_household | 8w | 0.16yrs | 0.16 | 0.16 | yes | Yes];No | No];Yes |
| g047 | Mum had cannabis >CH8MTHs | Quest_Mother | no | substance_household | 1yrs9m | 0.67-2yrs | 0.67 | 2 | yes | Every day];Often;Sometimes;Not at all | Often_Sometimes_Not at all];Every day |
| g053 | Mum had amphetamines >CH8MTHs | Quest_Mother | no | substance_household | 1yrs9m | 0.67-2yrs | 0.67 | 2 | yes | Every day;Often;Sometimes];Not at all | Not at all];Every day_Often_Sometimes |
| g056 | Mum had heroin meth coc >CH8MTHs | Quest_Mother | no | substance_household | 1yrs9m | 0.67-2yrs | 0.67 | 2 | yes | Every day;Sometimes];Not at all | Not at all];Every day_Sometimes |
| g613 | Partner alcoholic >CH8MTHs | Quest_Mother | no | substance_household | 1yrs9m | 0.67-2yrs | 0.67 | 2 | yes | Yes saw Doctor];yes did not see Dr;No Not at all | yes did not see Dr_No Not at all];Yes saw Doctor |
| pe062 | Taken Cannabis Since CH > 8 Months | Quest_Partner | no | substance_household | 1yrs9m | 0.67-2yrs | 0.67 | 2 | yes | Every Day];Often;Sometimes;Rarely;Never | Often_Sometimes_Rarely_Never];Every Day |
| pe067 | Taken Amphetamines Since CH > 8 Months | Quest_Partner | no | substance_household | 1yrs9m | 0.67-2yrs | 0.67 | 2 | yes | Every Day;Often;Sometimes;Rarely];Never | Never];Every Day_Often_Sometimes_Rarely |
| pe069 | Taken Heroin/Cocaine CH > 8 Months | Quest_Partner | no | substance_household | 1yrs9m | 0.67-2yrs | 0.67 | 2 | yes | Every Day;Often;Sometimes;Rarely];Never | Never];Every Day_Often_Sometimes_Rarely |
| h037 | A3c: Frequency Mum has taken cannabis since study child was 18 months old | Quest_Mother | no | substance_household | 2yrs9m | 1.5-3yrs | 1.5 | 3 | yes | Every Day];often;smts;not at all | often_smts_not at all];Every Day |
| h043 | A3i: Frequency Mum has taken amphetamines since study child was 18 months old | Quest_Mother | no | substance_household | 2yrs9m | 1.5-3yrs | 1.5 | 3 | yes | Every Day;often;smts];not at all | not at all];Every Day_often_smts |
| h046 | A3l: Frequency Mum has taken heroin, methadone, crack or cocaine since study child was 18 months old | Quest_Mother | no | substance_household | 2yrs9m | 1.5-3yrs | 1.5 | 3 | yes | Every Day;often;smts];not at all | not at all];Every Day_often_smts |
| h498 | H3m: Partner had an alcohol problem since study child was 18 months old | Quest_Mother | no | substance_household | 2yrs9m | 1.5-3yrs | 1.5 | 3 | yes | Yes, saw doctor];Yes, did not see doctor;No, not at all | Yes, did not see doctor_No, not at all];Yes, saw doctor |
| pf1032 | A3c: Frequency partner has taken cannabis/marihuana since the study child was 18 months old | Quest_Partner | no | substance_household | 2yrs9m | 1.5-3yrs | 1.5 | 3 | yes | Every day];Often;Sometimes;Not at all | Often_Sometimes_Not at all];Every day |
| pf1037 | A3h: Frequency partner has taken amphetamines or other stimulants since the study child was 18 months old | Quest_Partner | no | substance_household | 2yrs9m | 1.5-3yrs | 1.5 | 3 | yes | Every day;Often;Sometimes];Not at all | Not at all];Every day_Often_Sometimes |
| pf1039 | A3j: Frequency partner has taken heroin/methadone/crack/cocaine since the study child was 18 months old | Quest_Partner | no | substance_household | 2yrs9m | 1.5-3yrs | 1.5 | 3 | yes | Every day;Often;Sometimes];Not at all | Not at all];Every day_Often_Sometimes |
| f242 | Physically hurt by PTNR >CH born | Quest_Mother | no | violence_between_parents | 8m | 0-0.67yrs | 0 | 0.67 | yes | Y much affected;Y MOD affected;Y mildly affected];Y but N effect;N did not happen | N did not happen];Y much affected_Y MOD affected_Y mildly affected_Y but N effect |
| pd242 | Ptnr Physically Cruel Since Baby Born | Quest_Partner | no | violence_between_parents | 8m | 0-0.67yrs | 0 | 0.67 | yes | Yes Affected A Lot;Yes Mod Affected;Yes Mildly Affected];Yes Did Not Affect;No Did Not Happen | No Did Not Happen];Yes Affected A Lot_Yes Mod Affected_Yes Mildly Affected_Yes Did Not Affect |
| g322 | Partner physically cruel to mum >CH8MTHs | Quest_Mother | no | violence_between_parents | 1yrs9m | 0.67-2yrs | 0.67 | 2 | yes | Yes Big Effect;Yes Some Effect;Yes Mild Effect];Yes No Effect;Did Not Happen | Did Not Happen];Yes Big Effect_Yes Some Effect_Yes Mild Effect_Yes No Effect |
| pe322 | Partner Physically Cruel | Quest_Partner | no | violence_between_parents | 1yrs9m | 0.67-2yrs | 0.67 | 2 | yes | Yes & Affected Lots;Yes Mod Affected;Yes Mildly Affected];Yes Did Not Affect;No Did Not Happen | No Did Not Happen];Yes & Affected Lots_Yes Mod Affected_Yes Mildly Affected_Yes Did Not Affect |
| h232 | E23: Whether partner was physically cruel to mum since study child was 18 months old and effect this had | Quest_Mother | no | violence_between_parents | 2yrs9m | 1.5-3yrs | 1.5 | 3 | yes | yes had big effect;yes medium effect;yes mild effect];yes but no effect;didnt happen | didnt happen];yes had big effect_yes medium effect_yes mild effect_yes but no effect |
| pf5022 | E23: Partner's partner was physically cruel to them since study child was 18 months old | Quest_Partner | no | violence_between_parents | 2yrs9m | 1.5-3yrs | 1.5 | 3 | yes | Yes & affected me a lot;Yes, moderately affected;Yes, mildly affected];Yes, but did not affect me at all;No did not happen | No did not happen];Yes & affected me a lot_Yes, moderately affected_Yes, mildly affected_Yes, but did not affect me at all |

| **Table S3. ACE variables in middle childhood (3-7 yrs).** | | | | | | | | | | | |
| --- | --- | --- | --- | --- | --- | --- | --- | --- | --- | --- | --- |
| **variable name** | **description** | **datasource** | **retrospective** | **ACE** | **time_alspac** | **time_period** | **time_yrs_start** | **time_yrs_end** | **reverse_scale** | **recode_ACE** | **factor_level_after_recode** |
| k4037 | D38: Mother’s partner was emotionally cruel to children in past year | Quest_Mother | no | emotional_abuse | 5yrs1m | 4-5yrs | 4 | 5 | yes | Yes, affected a lot;Yes, moderately affected;Yes, mildly affected;Yes, did not affect at all];No, did not happen | No, did not happen];Yes, affected a lot_Yes, moderately affected_Yes, mildly affected_Yes, did not affect at all |
| k4038 | D39: Mother was emotionally cruel to children in past year | Quest_Mother | no | emotional_abuse | 5yrs1m | 4-5yrs | 4 | 5 | yes | Yes, affected a lot;Yes, moderately affected;Yes, mildly affected;Yes, did not affect at all];No, did not happen | No, did not happen];Yes, affected a lot_Yes, moderately affected_Yes, mildly affected_Yes, did not affect at all |
| ph4037 | D37: Respondent's assessment of how much their partner being emotionally cruel to the children in the last year has affected them | Quest_Partner | no | emotional_abuse | 5yrs1m | 4-5yrs | 4 | 5 | yes | Yes, affected me a lot;Yes, moderately affected;Yes, mildly affected;Yes, but did not affect me at all];No, did not happen | No, did not happen];Yes, affected me a lot_Yes, moderately affected_Yes, mildly affected_Yes, but did not affect me at all |
| ph4038 | D38: Respondent's assessment of how much being emotionally cruel to their children in the last year has affected them | Quest_Partner | no | emotional_abuse | 5yrs1m | 4-5yrs | 4 | 5 | yes | Yes, affected me a lot;Yes, moderately affected;Yes, mildly affected;Yes, but did not affect me at all];No, did not happen | No, did not happen];Yes, affected me a lot_Yes, moderately affected_Yes, mildly affected_Yes, but did not affect me at all |
| l4037 | D38: Respondent's partner was emotionally cruel to respondent's children since study child's 5th birthday | Quest_Mother | no | emotional_abuse | 6yrs1m | 5-6yrs | 5 | 6 | yes | Yes & affected respondent a lot;Yes, moderately affected;Yes, mildly affected;Yes, did not affect respondent at all];No, did not happen | No, did not happen];Yes & affected respondent a lot_Yes, moderately affected_Yes, mildly affected_Yes, did not affect respondent at all |
| l4038 | D39: Respondent was emotionally cruel to their children since study child's 5th birthday | Quest_Mother | no | emotional_abuse | 6yrs1m | 5-6yrs | 5 | 6 | yes | Yes & affected respondent a lot;Yes, moderately affected;Yes, mildly affected;Yes, did not affect respondent at all];No, did not happen | No, did not happen];Yes & affected respondent a lot_Yes, moderately affected_Yes, mildly affected_Yes, did not affect respondent at all |
| pj4037 | D37: Respondent's assessment of how much partner's emotional cruelty to children since study child's 5th birthday has affected them | Quest_Partner | no | emotional_abuse | 6yrs1m | 5-6yrs | 5 | 6 | yes | Yes and affected me a lot;Yes and moderately affected;Yes, mildly affected;Yes, but did not affect me at all];No, did not happen | No, did not happen];Yes and affected me a lot_Yes and moderately affected_Yes, mildly affected_Yes, but did not affect me at all |
| pj4038 | D38: Respondent's assessment of how much being emotionally cruel to children since study child's 5th birthday has affected them | Quest_Partner | no | emotional_abuse | 6yrs1m | 5-6yrs | 5 | 6 | yes | Yes and affected me a lot;Yes and moderately affected;Yes, mildly affected;Yes, but did not affect me at all];No, did not happen | No, did not happen];Yes and affected me a lot_Yes and moderately affected_Yes, mildly affected_Yes, but did not affect me at all |
| p2037 | B38: Mother's husband/partner was emotionally cruel to her children since the study child's 6th birthday | Quest_Mother | yes | emotional_abuse | 9yrs2m | 6-7yrs | 6 | 7 | special_yes | Yes, when the study child was 6 or 7];Yes, since the study child's 8th birthday];Yes, both when the study child was 6/7 and 8+];No, did not happen in past 3 years | No, did not happen in past 3 years_Yes, since the study child's 8th birthday];Yes, when the study child was 6 or 7_Yes, both when the study child was 6/7 and 8+ |
| p2038 | B39: Mother was emotionally cruel to her children since the study child's 6th birthday | Quest_Mother | yes | emotional_abuse | 9yrs2m | 6-7yrs | 6 | 7 | special_yes | Yes, when the study child was 6 or 7];Yes, since the study child's 8th birthday];Yes, both when the study child was 6/7 and 8+];No, did not happen in past 3 years | No, did not happen in past 3 years_Yes, since the study child's 8th birthday];Yes, when the study child was 6 or 7_Yes, both when the study child was 6/7 and 8+ |
| pm2037 | B38: Father's wife/partner was emotionally cruel to his children since the study child's 6th birthday | Quest_Partner | yes | emotional_abuse | 9yrs2m | 6-7yrs | 6 | 7 | special_yes | Yes, when the study child was 6 or 7];Yes, since the study child's 8th birthday];Yes, both when the study child was 6/7 and 8+];No, did not happen in past 3 years | No, did not happen in past 3 years_Yes, since the study child's 8th birthday];Yes, when the study child was 6 or 7_Yes, both when the study child was 6/7 and 8+ |
| pm2038 | B39: Father was emotionally cruel to his children since the study child's 6th birthday | Quest_Partner | yes | emotional_abuse | 9yrs2m | 6-7yrs | 6 | 7 | special_yes | Yes, when the study child was 6 or 7];Yes, since the study child's 8th birthday];Yes, both when the study child was 6/7 and 8+];No, did not happen in past 3 years | No, did not happen in past 3 years_Yes, since the study child's 8th birthday];Yes, when the study child was 6 or 7_Yes, both when the study child was 6/7 and 8+ |
| j044 | MUM took Depression Pills >1 YR | Quest_Mother | no | mental_health_problems_or_suicide | 3yrs11m | 3-4yrs | 3 | 4 | yes | Every Day;Often;Sometimes];Not at All | Not at All];Every Day_Often_Sometimes |
| j615 | Partner had Schizophrenia> 1 YR | Quest_Mother | no | mental_health_problems_or_suicide | 3yrs11m | 3-4yrs | 3 | 4 | yes | Yes & saw DR;Yes & didnt see DR];No Not at All | No Not at All];Yes & saw DR_Yes & didnt see DR |
| pg1034 | A3e: Frequency partner has taken pills for depression in the past year | Quest_Partner | no | mental_health_problems_or_suicide | 3yrs11m | 3-4yrs | 3 | 4 | yes | Once;Every day;Often;Sometimes];Not at all | Not at all];Once_Every day_Often_Sometimes |
| k1020 | A2k: Mother had schizophrenia in past year | Quest_Mother | no | mental_health_problems_or_suicide | 5yrs1m | 4-5yrs | 4 | 5 | yes | Yes, consulted doctor;Yes, did not consult doctor];No | No];Yes, consulted doctor_Yes, did not consult doctor |
| k1044 | A3e: Frequency mother had pills for depression in past year | Quest_Mother | no | mental_health_problems_or_suicide | 5yrs1m | 4-5yrs | 4 | 5 | yes | Every day;Often;Sometimes];Not at all | Not at all];Every day_Often_Sometimes |
| k4028 | D29: Mother attempted suicide in past year | Quest_Mother | no | mental_health_problems_or_suicide | 5yrs1m | 4-5yrs | 4 | 5 | yes | Yes, affected a lot;Yes, moderately affected;Yes, mildly affected;Yes, did not affect at all];No, did not happen | No, did not happen];Yes, affected a lot_Yes, moderately affected_Yes, mildly affected_Yes, did not affect at all |
| ph1020 | A2k: Respondent had schizophrenia in the past year | Quest_Partner | no | mental_health_problems_or_suicide | 5yrs1m | 4-5yrs | 4 | 5 | yes | Yes, consulted a doctor;Yes, did not consult a doctor];No | No];Yes, consulted a doctor_Yes, did not consult a doctor |
| ph1044 | A3e: Frequency in the past year respondent has taken pills for depression | Quest_Partner | no | mental_health_problems_or_suicide | 5yrs1m | 4-5yrs | 4 | 5 | yes | Every day;Often;Sometimes];Not at all | Not at all];Every day_Often_Sometimes |
| ph4028 | D29: Respondent's assessment of how much attempting suicide in the last year has affected them | Quest_Partner | no | mental_health_problems_or_suicide | 5yrs1m | 4-5yrs | 4 | 5 | yes | Yes, affected me a lot;Yes, mildly affected;Yes, but did not affect me at all];No, did not happen | No, did not happen];Yes, affected me a lot_Yes, mildly affected_Yes, but did not affect me at all |
| l3020 | C2k: Respondent has had/continued to have schizophrenia since study child's 5th birthday | Quest_Mother | no | mental_health_problems_or_suicide | 6yrs1m | 5-6yrs | 5 | 6 | yes | Yes & consulted doctor;Yes but did not consult doctor];No | No];Yes & consulted doctor_Yes but did not consult doctor |
| l3044 | C3e: Frequency respondent has taken pills for depression since study child's 5th birthday | Quest_Mother | no | mental_health_problems_or_suicide | 6yrs1m | 5-6yrs | 5 | 6 | yes | Every day;Often;Sometimes];Not at all | Not at all];Every day_Often_Sometimes |
| l4028 | D29: Respondent attempted suicide since study child's 5th birthday | Quest_Mother | no | mental_health_problems_or_suicide | 6yrs1m | 5-6yrs | 5 | 6 | yes | Yes & affected respondent a lot;Yes, moderately affected;Yes, mildly affected;Yes, did not affect respondent at all];No, did not happen | No, did not happen];Yes & affected respondent a lot_Yes, moderately affected_Yes, mildly affected_Yes, did not affect respondent at all |
| l6031 | F3l: Respondent's partner has had schizophrenia since study child was 5 years old | Quest_Mother | no | mental_health_problems_or_suicide | 6yrs1m | 5-6yrs | 5 | 6 | yes | Yes & saw a doctor;Yes, but did not see a doctor];No, not at all;Do not know | No, not at all_Do not know];Yes & saw a doctor_Yes, but did not see a doctor |
| pj3020 | C2k: Respondent has suffered from schizophrenia since child's 5th birthday | Quest_Partner | no | mental_health_problems_or_suicide | 6yrs1m | 5-6yrs | 5 | 6 | yes | Yes and consulted a doctor;Yes but did not consult a doctor];No | No];Yes and consulted a doctor_Yes but did not consult a doctor |
| pj3044 | C3e: Respondent has taken pills for depression since child's 5th birthday | Quest_Partner | no | mental_health_problems_or_suicide | 6yrs1m | 5-6yrs | 5 | 6 | yes | Every day;Often;Sometimes];Not at all | Not at all];Every day_Often_Sometimes |
| pj4028 | D29: Respondent's assessment of how much attempting suicide since study child's 5th birthday has affected them | Quest_Partner | no | mental_health_problems_or_suicide | 6yrs1m | 5-6yrs | 5 | 6 | yes | Yes and affected me a lot;Yes, mildly affected;Yes, but did not affect me at all];No, did not happen | No, did not happen];Yes and affected me a lot_Yes, mildly affected_Yes, but did not affect me at all |
| pj6031 | F3l: Respondent's partner has had schizophrenia since study child was 5 | Quest_Partner | no | mental_health_problems_or_suicide | 6yrs1m | 5-6yrs | 5 | 6 | yes | Yes and saw a doctor];No, not at all;Do not know | No, not at all_Do not know];Yes and saw a doctor |
| p2028 | B29: Mother attempted suicide since the study child's 6th birthday | Quest_Mother | yes | mental_health_problems_or_suicide | 9yrs2m | 6-7yrs | 6 | 7 | special_yes | Yes, when the study child was 6 or 7];Yes, since the study child's 8th birthday];Yes, both when the study child was 6/7 and 8+];No, did not happen in past 3 years | No, did not happen in past 3 years_Yes, since the study child's 8th birthday;]Yes, when the study child was 6 or 7_Yes, both when the study child was 6/7 and 8+ |
| pm2028 | B29: Father attempted suicide since the study child's 6th birthday | Quest_Partner | yes | mental_health_problems_or_suicide | 9yrs2m | 6-7yrs | 6 | 7 | yes | Yes, when the study child was 6 or 7];Yes, since the study child's 8th birthday;No, did not happen in past 3 years | No, did not happen in past 3 years_Yes, since the study child's 8th birthday];Yes, when the study child was 6 or 7 |
| j328 | MUM Attempted Suicide> CH 30 MTHs | Quest_Mother | no | mental_health_problems_or_suicide | 3yrs11m | 2.5-4yrs | 2.5 | 4 | yes | Yes but Not Affected;Yes Bit affected;Yes MOD Affected;Yes & Affected Lot];No | No];Yes but Not Affected_Yes Bit affected_Yes MOD Affected_Yes & Affected Lot |
| pg3028 | C29: Degree to which attempted suicide affected partner since child was 2.5 years old | Quest_Partner | no | mental_health_problems_or_suicide | 3yrs11m | 2.5-4yrs | 2.5 | 4 | yes | A lot;Mildly;Not at all];Did not happen | Did not happen];A lot_Mildly_Not at all |
| j567 | MUM Feels Close to CH | Quest_Mother | no | parent_child_bond | 3yrs11m | 4yrs | 4 | 4 | special_no | Yes];No];Yes & No | Yes & No_Yes];No |
| j577 | Partner Feels Close to CH | Quest_Mother | no | parent_child_bond | 3yrs11m | 4yrs | 4 | 4 | special_no | Yes];No];Sometimes | Sometimes_Yes];No |
| pg4167 | D10h: Partner feels very close to child | Quest_Partner | no | parent_child_bond | 3yrs11m | 4yrs | 4 | 4 | special_no | Yes];No];Sometimes | Sometimes_Yes];No |
| pg4177 | D11h: Partner's partner feels very close to child | Quest_Partner | no | parent_child_bond | 3yrs11m | 4yrs | 4 | 4 | special_no | Yes];No];Sometimes | Sometimes_Yes];No |
| l6054 | F4e: Frequency respondent's partner seems to feel very close to study child | Quest_Mother | no | parent_child_bond | 6yrs1m | 6yrs | 6 | 6 | no | Always how respondent feels;Sometimes how respondent feels];Respondent never feels this way | Always how respondent feels_Sometimes how respondent feels];Respondent never feels this way |
| pj6054 | F4e: Respondent's partner feels very close to study child | Quest_Partner | no | parent_child_bond | 6yrs1m | 6yrs | 6 | 6 | no | This is how respondent always feels;This is sometimes how respondent feels];Respondent never feels this way | This is how respondent always feels_This is sometimes how respondent feels];Respondent never feels this way |
| m3357 | C18h: Mother seems to feel close to study child | Quest_Mother | no | parent_child_bond | 7yrs1m | 7yrs | 7 | 7 | special_no | Yes];No];Sometimes | Yes];No_Sometimes |
| m3367 | C19h: Partner seems to feel close to study child | Quest_Mother | no | parent_child_bond | 7yrs1m | 7yrs | 7 | 7 | special_no | Yes];No];Sometimes | Yes];No_Sometimes |
| pk3357 | C18h: Partner feels very close to study child | Quest_Partner | no | parent_child_bond | 7yrs1m | 7yrs | 7 | 7 | special_no | Yes];No];Sometimes/occasionally | Sometimes/occasionally_Yes];No |
| pk3367 | C19h: Partner's partner seems to feel very close to study child | Quest_Partner | no | parent_child_bond | 7yrs1m | 7yrs | 7 | 7 | special_no | Yes];No];Sometimes/occasionally | Sometimes/occasionally_Yes];No |
| k4029 | D30: Mother was convicted of an offence in past year | Quest_Mother | no | parent_convicted_offence | 5yrs1m | 4-5yrs | 4 | 5 | yes | Yes, affected a lot;Yes, moderately affected;Yes, mildly affected;Yes, did not affect at all];No, did not happen | No, did not happen];Yes, affected a lot_Yes, moderately affected_Yes, mildly affected_Yes, did not affect at all |
| ph4029 | D30: Respondent's assessment of how much being convicted of an offence in the last year has affected them | Quest_Partner | no | parent_convicted_offence | 5yrs1m | 4-5yrs | 4 | 5 | yes | Yes, affected me a lot;Yes, moderately affected;Yes, mildly affected;Yes, but did not affect me at all];No, did not happen | No, did not happen];Yes, affected me a lot_Yes, moderately affected_Yes, mildly affected_Yes, but did not affect me at all |
| l4029 | D30: Respondent convicted of an offence since study child's 5th birthday | Quest_Mother | no | parent_convicted_offence | 6yrs1m | 5-6yrs | 5 | 6 | yes | Yes & affected respondent a lot;Yes, moderately affected;Yes, mildly affected;Yes, did not affect respondent at all];No, did not happen | No, did not happen];Yes & affected respondent a lot_Yes, moderately affected_Yes, mildly affected_Yes, did not affect respondent at all |
| pj4029 | D30: Respondent's assessment of how much being convicted of an offence since study child's 5th birthday has affected them | Quest_Partner | no | parent_convicted_offence | 6yrs1m | 5-6yrs | 5 | 6 | yes | Yes and affected me a lot;Yes and moderately affected;Yes, mildly affected;Yes, but did not affect me at all];No, did not happen | No, did not happen];Yes and affected me a lot_Yes and moderately affected_Yes, mildly affected_Yes, but did not affect me at all |
| p2029 | B30: Mother was convicted of an offence since the study child's 6th birthday | Quest_Mother | yes | parent_convicted_offence | 9yrs2m | 6-7yrs | 6 | 7 | yes | Yes, when the study child was 6 or 7];Yes, since the study child's 8th birthday;No, did not happen in past 3 years | No, did not happen in past 3 years_Yes, since the study child's 8th birthday];Yes, when the study child was 6 or 7 |
| pm2029 | B30: Father was convicted of an offence since the study child's 6th birthday | Quest_Partner | yes | parent_convicted_offence | 9yrs2m | 6-7yrs | 6 | 7 | yes | Yes, when the study child was 6 or 7];Yes, since the study child's 8th birthday;No, did not happen in past 3 years | No, did not happen in past 3 years_Yes, since the study child's 8th birthday];Yes, when the study child was 6 or 7 |
| j329 | MUM Convicted of Offence> CH 30 MTHs | Quest_Mother | no | parent_convicted_offence | 3yrs11m | 2.5-4yrs | 2.5 | 4 | yes | Yes but Not Affected;Yes Bit affected;Yes MOD Affected;Yes & Affected Lot];No | No];Yes but Not Affected_Yes Bit affected_Yes MOD Affected_Yes & Affected Lot |
| pg3029 | C30: Degree to which a criminal conviction affected partner since child was 2.5 years old | Quest_Partner | no | parent_convicted_offence | 3yrs11m | 2.5-4yrs | 2.5 | 4 | yes | A lot;Moderately;Mildly;Not at all];Did not happen | Did not happen];A lot_Moderately_Mildly_Not at all |
| k4008 | D9: Mother was divorced in past year | Quest_Mother | no | parental_separation | 5yrs1m | 4-5yrs | 4 | 5 | yes | Yes, affected a lot;Yes, moderately affected;Yes, mildly affected;Yes, did not affect at all];No, did not happen | No, did not happen];Yes, affected a lot_Yes, moderately affected_Yes, mildly affected_Yes, did not affect at all |
| k4017 | D18: Mother and partner separated in past year | Quest_Mother | no | parental_separation | 5yrs1m | 4-5yrs | 4 | 5 | yes | Yes, affected a lot;Yes, moderately affected;Yes, mildly affected;Yes, did not affect at all];No, did not happen | No, did not happen];Yes, affected a lot_Yes, moderately affected_Yes, mildly affected_Yes, did not affect at all |
| ph4017 | D18: Respondent's assessment of how much separating from their partner in the last year has affected them | Quest_Partner | no | parental_separation | 5yrs1m | 4-5yrs | 4 | 5 | yes | Yes, affected me a lot;Yes, moderately affected;Yes, mildly affected;Yes, but did not affect me at all];No, did not happen | No, did not happen];Yes, affected me a lot_Yes, moderately affected_Yes, mildly affected_Yes, but did not affect me at all |
| l4008 | D9: Respondent was divorced since study child's 5th birthday | Quest_Mother | no | parental_separation | 6yrs1m | 5-6yrs | 5 | 6 | yes | Yes & affected respondent a lot;Yes, moderately affected;Yes, mildly affected;Yes, did not affect respondent at all];No, did not happen | No, did not happen];Yes & affected respondent a lot_Yes, moderately affected_Yes, mildly affected_Yes, did not affect respondent at all |
| l4017 | D18: Respondent separated from partner since study child's 5th birthday | Quest_Mother | no | parental_separation | 6yrs1m | 5-6yrs | 5 | 6 | yes | Yes & affected respondent a lot;Yes, moderately affected;Yes, mildly affected;Yes, did not affect respondent at all];No, did not happen | No, did not happen];Yes & affected respondent a lot_Yes, moderately affected_Yes, mildly affected_Yes, did not affect respondent at all |
| pj4008 | D9: Respondent's assessment of how much divorce since study child's 5th birthday has affected them | Quest_Partner | no | parental_separation | 6yrs1m | 5-6yrs | 5 | 6 | yes | Yes and affected me a lot;Yes and moderately affected;Yes, mildly affected;Yes, but did not affect me at all];No, did not happen | No, did not happen];Yes and affected me a lot_Yes and moderately affected_Yes, mildly affected_Yes, but did not affect me at all |
| pj4017 | D18: Respondent's assessment of how much separating from partner since study child's 5th birthday has affected them | Quest_Partner | no | parental_separation | 6yrs1m | 5-6yrs | 5 | 6 | yes | Yes and affected me a lot;Yes and moderately affected;Yes, mildly affected];No, did not happen | No, did not happen];Yes and affected me a lot_Yes and moderately affected_Yes, mildly affected |
| p2008 | B9: Mother was divorced since the study child's 6th birthday | Quest_Mother | yes | parental_separation | 9yrs2m | 6-7yrs | 6 | 7 | special_yes | Yes, when the study child was 6 or 7];Yes, since the study child's 8th birthday];Yes, both when the study child was 6/7 and 8+];No, did not happen in past 3 years | No, did not happen in past 3 years_Yes, since the study child's 8th birthday];Yes, when the study child was 6 or 7_Yes, both when the study child was 6/7 and 8+ |
| p2017 | B18: Mother and husband/partner separated since the study child's 6th birthday | Quest_Mother | yes | parental_separation | 9yrs2m | 6-7yrs | 6 | 7 | special_yes | Yes, when the study child was 6 or 7];Yes, since the study child's 8th birthday];Yes, both when the study child was 6/7 and 8+];No, did not happen in past 3 years | No, did not happen in past 3 years_Yes, since the study child's 8th birthday];Yes, when the study child was 6 or 7_Yes, both when the study child was 6/7 and 8+ |
| pm2008 | B9: Father was divorced since the study child's 6th birthday | Quest_Partner | yes | parental_separation | 9yrs2m | 6-7yrs | 6 | 7 | special_yes | Yes, when the study child was 6 or 7];Yes, since the study child's 8th birthday];Yes, both when the study child was 6/7 and 8+];No, did not happen in past 3 years | No, did not happen in past 3 years_Yes, since the study child's 8th birthday];Yes, when the study child was 6 or 7_Yes, both when the study child was 6/7 and 8+ |
| pm2017 | B18: Father and wife/partner separated since the study child's 6th birthday | Quest_Partner | yes | parental_separation | 9yrs2m | 6-7yrs | 6 | 7 | special_yes | Yes, when the study child was 6 or 7];Yes, since the study child's 8th birthday];Yes, both when the study child was 6/7 and 8+];No, did not happen in past 3 years | No, did not happen in past 3 years_Yes, since the study child's 8th birthday];Yes, when the study child was 6 or 7_Yes, both when the study child was 6/7 and 8+ |
| j308 | MUM Divorced> CH 30 MTHs | Quest_Mother | no | parental_separation | 3yrs11m | 2.5-4yrs | 2.5 | 4 | yes | Yes but Not Affected;Yes Bit affected;Yes MOD Affected;Yes & Affected Lot];No | No];Yes but Not Affected_Yes Bit affected_Yes MOD Affected_Yes & Affected Lot |
| j317 | MUM & PTR Separated> CH 30 MTHs | Quest_Mother | no | parental_separation | 3yrs11m | 2.5-4yrs | 2.5 | 4 | yes | Yes but Not Affected;Yes Bit affected;Yes MOD Affected;Yes & Affected Lot];No | No];Yes but Not Affected_Yes Bit affected_Yes MOD Affected_Yes & Affected Lot |
| pg3008 | C9: Degree to which divorce affected partner since child was 2.5 years old | Quest_Partner | no | parental_separation | 3yrs11m | 2.5-4yrs | 2.5 | 4 | yes | A lot;Moderately;Mildly;Not at all];Did not happen | Did not happen];A lot_Moderately_Mildly_Not at all |
| pg3017 | C18: Degree to which separation affected partner since child was 2.5 years old | Quest_Partner | no | parental_separation | 3yrs11m | 2.5-4yrs | 2.5 | 4 | yes | A lot;Moderately;Mildly;Not at all];Did not happen | Did not happen];A lot_Moderately_Mildly_Not at all |
| k4026 | D27: Mother’s partner was physically cruel to children in past year | Quest_Mother | no | physical_abuse | 5yrs1m | 4-5yrs | 4 | 5 | yes | Yes, affected a lot;Yes, moderately affected;Yes, mildly affected;Yes, did not affect at all];No, did not happen | No, did not happen];Yes, affected a lot_Yes, moderately affected_Yes, mildly affected_Yes, did not affect at all |
| k4027 | D28: Mother was physically cruel to children in past year | Quest_Mother | no | physical_abuse | 5yrs1m | 4-5yrs | 4 | 5 | yes | Yes, affected a lot;Yes, moderately affected;Yes, mildly affected;Yes, did not affect at all];No, did not happen | No, did not happen];Yes, affected a lot_Yes, moderately affected_Yes, mildly affected_Yes, did not affect at all |
| ph4026 | D27: Respondent's assessment how much their partner being physically cruel to the children in the last year has affected them | Quest_Partner | no | physical_abuse | 5yrs1m | 4-5yrs | 4 | 5 | yes | Yes, affected me a lot;Yes, moderately affected;Yes, mildly affected;Yes, but did not affect me at all];No, did not happen | No, did not happen];Yes, affected me a lot_Yes, moderately affected_Yes, mildly affected_Yes, but did not affect me at all |
| ph4027 | D28: Respondent's assessment of how much being physically cruel to the children in the last year has affected them | Quest_Partner | no | physical_abuse | 5yrs1m | 4-5yrs | 4 | 5 | yes | Yes, affected me a lot;Yes, moderately affected;Yes, mildly affected;Yes, but did not affect me at all];No, did not happen | No, did not happen];Yes, affected me a lot_Yes, moderately affected_Yes, mildly affected_Yes, but did not affect me at all |
| l4026 | D27: Respondent's partner physically cruel to respondent's children since study child's 5th birthday | Quest_Mother | no | physical_abuse | 6yrs1m | 5-6yrs | 5 | 6 | yes | Yes & affected respondent a lot;Yes, moderately affected;Yes, mildly affected;Yes, did not affect respondent at all];No, did not happen | No, did not happen];Yes & affected respondent a lot_Yes, moderately affected_Yes, mildly affected_Yes, did not affect respondent at all |
| l4027 | D28: Respondent physically cruel to own children since study child's 5th birthday | Quest_Mother | no | physical_abuse | 6yrs1m | 5-6yrs | 5 | 6 | yes | Yes & affected respondent a lot;Yes, moderately affected;Yes, mildly affected;Yes, did not affect respondent at all];No, did not happen | No, did not happen];Yes & affected respondent a lot_Yes, moderately affected_Yes, mildly affected_Yes, did not affect respondent at all |
| pj4026 | D27: Respondent's assessment of how much partner's physical cruelty to children since study child's 5th birthday has affected them | Quest_Partner | no | physical_abuse | 6yrs1m | 5-6yrs | 5 | 6 | yes | Yes and affected me a lot;Yes and moderately affected;Yes, mildly affected;Yes, but did not affect me at all];No, did not happen | No, did not happen];Yes and affected me a lot_Yes and moderately affected_Yes, mildly affected_Yes, but did not affect me at all |
| pj4027 | D28: Respondent's assessment of how much being physically cruel to children since study child's 5th birthday has affected them | Quest_Partner | no | physical_abuse | 6yrs1m | 5-6yrs | 5 | 6 | yes | Yes and affected me a lot;Yes and moderately affected;Yes, mildly affected;Yes, but did not affect me at all];No, did not happen | No, did not happen];Yes and affected me a lot_Yes and moderately affected_Yes, mildly affected_Yes, but did not affect me at all |
| pm2026 | B27: Father's wife/partner was physically cruel to his children since the study child's 6th birthday | Quest_Partner | yes | physical_abuse | 9yrs2m | 6-7yrs | 6 | 7 | special_no | Yes, since the study child's 8th birthday];Yes, both when the study child was 6/7 and 8+];No, did not happen in past 3 years | No, did not happen in past 3 years_Yes, since the study child's 8th birthday];Yes, both when the study child was 6/7 and 8+ |
| pm2027 | B28: Father was physically cruel to his children since the study child's 6th birthday | Quest_Partner | yes | physical_abuse | 9yrs2m | 6-7yrs | 6 | 7 | special_no | Yes, since the study child's 8th birthday];Yes, both when the study child was 6/7 and 8+];No, did not happen in past 3 years | No, did not happen in past 3 years_Yes, since the study child's 8th birthday];Yes, both when the study child was 6/7 and 8+ |
| p2026 | B27: Mother's husband/partner was physically cruel to her children since the study child's 6th birthday | Quest_Mother | yes | physical_abuse | 9yrs2m | 6-7yrs | 6 | 7 | special_yes | Yes, when the study child was 6 or 7];Yes, since the study child's 8th birthday];Yes, both when the study child was 6/7 and 8+];No, did not happen in past 3 years | No, did not happen in past 3 years_Yes, since the study child's 8th birthday];Yes, when the study child was 6 or 7_Yes, both when the study child was 6/7 and 8+ |
| p2027 | B28: Mother was physically cruel to her children since the study child's 6th birthday | Quest_Mother | yes | physical_abuse | 9yrs2m | 6-7yrs | 6 | 7 | special_yes | Yes, when the study child was 6 or 7];Yes, since the study child's 8th birthday];Yes, both when the study child was 6/7 and 8+];No, did not happen in past 3 years | No, did not happen in past 3 years_Yes, since the study child's 8th birthday];Yes, when the study child was 6 or 7_Yes, both when the study child was 6/7 and 8+ |
| j326 | PTR PHYS Cruel to CDRN> CH 30 MTHs | Quest_Mother | no | physical_abuse | 3yrs11m | 2.5-4yrs | 2.5 | 4 | yes | Yes but Not Affected;Yes Bit affected;Yes MOD Affected;Yes & Affected Lot];No | No];Yes but Not Affected_Yes Bit affected_Yes MOD Affected_Yes & Affected Lot |
| j327 | MUM PHYS Cruel to CDRN> CH 30 MTHs | Quest_Mother | no | physical_abuse | 3yrs11m | 2.5-4yrs | 2.5 | 4 | yes | Yes but Not Affected;Yes Bit affected;Yes MOD Affected;Yes & Affected Lot];No | No];Yes but Not Affected_Yes Bit affected_Yes MOD Affected_Yes & Affected Lot |
| pg3026 | C27: Degree to which physical cruelty from a partner to children affected partner since study child was 2.5 years old | Quest_Partner | no | physical_abuse | 3yrs11m | 2.5-4yrs | 2.5 | 4 | yes | A lot;Moderately;Mildly;Not at all];Did not happen | Did not happen];A lot_Moderately_Mildly_Not at all |
| pg3027 | C28: Degree to which partner being physically cruel to children affected partner since study child was 2.5 years old | Quest_Partner | no | physical_abuse | 3yrs11m | 2.5-4yrs | 2.5 | 4 | yes | A lot;Moderately;Mildly;Not at all];Did not happen | Did not happen];A lot_Moderately_Mildly_Not at all |
| kl475 | D6: Child was sexually abused since age 3 | Quest_Child Based | no | sexual_abuse | 57m | 3-5yrs | 3 | 5 | yes | Yes, child was very upset;Yes, child was quite upset;Yes, child was a bit upset];No, did not happen | No, did not happen];Yes, child was very upset_Yes, child was quite upset_Yes, child was a bit upset |
| kn4005 | D6: Child sexually abused in past 15 months | Quest_Child Based | no | sexual_abuse | 69m | 4.5-6yrs | 4.5 | 6 | yes | Yes And Was Very Upset;Yes And Was Quite Upset;Yes And Was A Bit Upset;Yes But Was Not Upset];No Did Not Happen | No Did Not Happen];Yes And Was Very Upset_Yes And Was Quite Upset_Yes And Was A Bit Upset_Yes But Was Not Upset |
| kq365 | D6: Child was sexually abused since his/her 5th birthday | Quest_Child Based | no | sexual_abuse | 81m | 5-7yrs | 5 | 7 | yes | Yes, very upset;Yes, quite upset;Yes, a bit upset;Yes, not upset];No, did not happen | No, did not happen];Yes, very upset_Yes, quite upset_Yes, a bit upset_Yes, not upset |
| pg1032 | A3c: Frequency partner has used cannabis/marihuana in the past year | Quest_Partner | no | substance_household | 3yrs11m | 3-4yrs | 3 | 4 | special_no | Once];Every day];Often;Sometimes;Not at all | Often_Sometimes_Once_Not at all];Every day |
| j042 | MUM took Cannabis >1 YR | Quest_Mother | no | substance_household | 3yrs11m | 3-4yrs | 3 | 4 | yes | Every Day];Often;Sometimes;Not at All | Often_Sometimes_Not at All];Every Day |
| j048 | MUM took Amphetamines >1 YR | Quest_Mother | no | substance_household | 3yrs11m | 3-4yrs | 3 | 4 | yes | Every Day;Often;Sometimes];Not at All | Not at All];Every Day_Often_Sometimes |
| j051 | MUM took Heroin >1 YR | Quest_Mother | no | substance_household | 3yrs11m | 3-4yrs | 3 | 4 | yes | Every Day;Often;Sometimes];Not at All | Not at All];Every Day_Often_Sometimes |
| j616 | Partner had Alcohol Problem> 1 YR | Quest_Mother | no | substance_household | 3yrs11m | 3-4yrs | 3 | 4 | yes | Yes & saw DR];Yes & didnt see DR;No Not at All | Yes & didnt see DR_No Not at All];Yes & saw DR |
| pg1037 | A3h: Frequency partner has taken amphetamines or other stimulants in the past year | Quest_Partner | no | substance_household | 3yrs11m | 3-4yrs | 3 | 4 | yes | Every day;Often;Sometimes];Not at all | Not at all];Every day_Often_Sometimes |
| pg1039 | A3j: Frequency partner has taken heroin, methadone, crack or cocaine in the past year | Quest_Partner | no | substance_household | 3yrs11m | 3-4yrs | 3 | 4 | yes | Every day;Sometimes];Not at all | Not at all];Every day_Sometimes |
| k1022 | A2m: Mother had alcohol problem in past year | Quest_Mother | no | substance_household | 5yrs1m | 4-5yrs | 4 | 5 | yes | Yes, consulted doctor];Yes, did not consult doctor;No | Yes, did not consult doctor_No];Yes, consulted doctor |
| k1042 | A3c: Frequency mother had cannabis/marijuana in past year | Quest_Mother | no | substance_household | 5yrs1m | 4-5yrs | 4 | 5 | yes | Every day];Often;Sometimes;Not at all;Once | Often_Sometimes_Not at all_Once];Every day |
| k1050 | A3k: Frequency mother had amphetamines in past year | Quest_Mother | no | substance_household | 5yrs1m | 4-5yrs | 4 | 5 | yes | Every day;Often;Sometimes];Not at all;Once | Not at all_Once];Every day_Often_Sometimes |
| k1053 | A3n: Frequency mother had heroin/methadone/cocaine in past year | Quest_Mother | no | substance_household | 5yrs1m | 4-5yrs | 4 | 5 | yes | Every day;Often;Sometimes];Not at all | Not at all];Every day_Often_Sometimes |
| ph1022 | A2m: Respondent had alcohol problems in the past year | Quest_Partner | no | substance_household | 5yrs1m | 4-5yrs | 4 | 5 | yes | Yes, consulted a doctor];Yes, did not consult a doctor;No | Yes, did not consult a doctor_No];Yes, consulted a doctor |
| ph1042 | A3c: Frequency in the past year respondent has taken cannabis/marihuana | Quest_Partner | no | substance_household | 5yrs1m | 4-5yrs | 4 | 5 | yes | Every day];Often;Sometimes;Not at all | Often_Sometimes_Not at all];Every day |
| ph1050 | A3k: Frequency in the past year respondent has taken amphetamines/other stimulants | Quest_Partner | no | substance_household | 5yrs1m | 4-5yrs | 4 | 5 | yes | Every day;Often;Sometimes];Not at all | Not at all];Every day_Often_Sometimes |
| ph1053 | A3m: Frequency in the past year respondent has taken heroin/methadone/crack/cocaine | Quest_Partner | no | substance_household | 5yrs1m | 4-5yrs | 4 | 5 | yes | Sometimes];Not at all | Not at all];Sometimes |
| pj3053 | C3l: Respondent has taken heroin, methadone, crack or cocaine since child's 5th birthday | Quest_Partner | no | substance_household | 6yrs1m | 5-6yrs | 5 | 6 | special_yes | Every day;Sometimes];Not at all];Once | Not at all];Once_Every day_Sometimes |
| l3022 | C2m: Respondent has had/continued to have alcohol problem since study child's 5th birthday | Quest_Mother | no | substance_household | 6yrs1m | 5-6yrs | 5 | 6 | yes | Yes & consulted doctor];Yes but did not consult doctor;No | Yes but did not consult doctor_No];Yes & consulted doctor |
| l3042 | C3c: Frequency respondent has taken cannabis/marihuana since study child's 5th birthday | Quest_Mother | no | substance_household | 6yrs1m | 5-6yrs | 5 | 6 | yes | Every day];Often;Sometimes;Not at all;Once Only | Often_Sometimes_Not at all_Once Only];Every day |
| l3050 | C3k: Frequency respondent has taken amphetamines/other stimulants since study child's 5th birthday | Quest_Mother | no | substance_household | 6yrs1m | 5-6yrs | 5 | 6 | yes | Every day;Often;Sometimes];Not at all | Not at all];Every day_Often_Sometimes |
| l3053 | C3n: Frequency respondent has taken heroin, methadone, crack, cocaine since study child's 5th birthday | Quest_Mother | no | substance_household | 6yrs1m | 5-6yrs | 5 | 6 | yes | Every day;Often;Sometimes];Not at all | Not at all];Every day_Often_Sometimes |
| l6032 | F3m: Respondent's partner has had a drink (alcohol) problem since study child was 5 years old | Quest_Mother | no | substance_household | 6yrs1m | 5-6yrs | 5 | 6 | yes | Yes & saw a doctor;Yes, but did not see a doctor];No, not at all;Do not know | No, not at all_Do not know];Yes & saw a doctor_Yes, but did not see a doctor |
| pj3022 | C2m: Respondent has suffered from alcohol problems since child's 5th birthday | Quest_Partner | no | substance_household | 6yrs1m | 5-6yrs | 5 | 6 | yes | Yes and consulted a doctor];Yes but did not consult a doctor;No | Yes but did not consult a doctor_No];Yes and consulted a doctor |
| pj3042 | C3c: Respondent has taken cannabis or marihuana since child's 5th birthday | Quest_Partner | no | substance_household | 6yrs1m | 5-6yrs | 5 | 6 | yes | Every day];Often;Sometimes;Not at all | Often_Sometimes_Not at all];Every day |
| pj3050 | C3j: Respondent has taken amphetamines or other stimulants since child's 5th birthday | Quest_Partner | no | substance_household | 6yrs1m | 5-6yrs | 5 | 6 | yes | Often;Sometimes];Not at all | Not at all];Often_Sometimes |
| pj6032 | F3m: Respondent's partner has had alcohol problems since study child was 5 | Quest_Partner | no | substance_household | 6yrs1m | 5-6yrs | 5 | 6 | yes | Yes and saw a doctor];Yes but did not see a doctor;No, not at all;Do not know | Yes but did not see a doctor_No, not at all_Do not know];Yes and saw a doctor |
| k4022 | D23: Mother’s partner was physically cruel to her in past year | Quest_Mother | no | violence_between_parents | 5yrs1m | 4-5yrs | 4 | 5 | yes | Yes, affected a lot;Yes, moderately affected;Yes, mildly affected];Yes, did not affect at all;No, did not happen | No, did not happen];Yes, affected a lot_Yes, moderately affected_Yes, mildly affected_Yes, did not affect at all |
| ph4022 | D23: Respondent's assessment of how much their partner being physically cruel in the last year has affected them | Quest_Partner | no | violence_between_parents | 5yrs1m | 4-5yrs | 4 | 5 | yes | Yes, affected me a lot;Yes, moderately affected;Yes, mildly affected];Yes, but did not affect me at all;No, did not happen | No, did not happen];Yes, affected me a lot_Yes, moderately affected_Yes, mildly affected_Yes, but did not affect me at all |
| l4022 | D23: Respondent's partner was physically cruel to them since study child's 5th birthday | Quest_Mother | no | violence_between_parents | 6yrs1m | 5-6yrs | 5 | 6 | yes | Yes & affected respondent a lot;Yes, moderately affected;Yes, mildly affected];Yes, did not affect respondent at all;No, did not happen | No, did not happen];Yes & affected respondent a lot_Yes, moderately affected_Yes, mildly affected_Yes, did not affect respondent at all |
| pj4022 | D23: Respondent's assessment of how much partner being physically cruel since study child's 5th birthday has affected them | Quest_Partner | no | violence_between_parents | 6yrs1m | 5-6yrs | 5 | 6 | yes | Yes and affected me a lot;Yes and moderately affected;Yes, mildly affected];Yes, but did not affect me at all;No, did not happen | No, did not happen];Yes and affected me a lot_Yes and moderately affected_Yes, mildly affected_Yes, but did not affect me at all |
| p2022 | B23: Mother's husband/partner was physically cruel to her since the study child's 6th birthday | Quest_Mother | yes | violence_between_parents | 9yrs2m | 6-7yrs | 6 | 7 | special_yes | Yes, when the study child was 6 or 7];Yes, since the study child's 8th birthday];Yes, both when the study child was 6/7 and 8+];No, did not happen in past 3 years | No, did not happen in past 3 years_Yes, since the study child's 8th birthday];Yes, when the study child was 6 or 7_Yes, both when the study child was 6/7 and 8+ |
| pm2022 | B23: Father's wife/partner was physically cruel to him since the study child's 6th birthday | Quest_Partner | yes | violence_between_parents | 9yrs2m | 6-7yrs | 6 | 7 | special_yes | Yes, when the study child was 6 or 7];Yes, since the study child's 8th birthday];Yes, both when the study child was 6/7 and 8+];No, did not happen in past 3 years | No, did not happen in past 3 years_Yes, since the study child's 8th birthday];Yes, when the study child was 6 or 7_Yes, both when the study child was 6/7 and 8+ |
| j322 | PTR PHYS Cruel to MUM> CH 30 MTHs | Quest_Mother | no | violence_between_parents | 3yrs11m | 2.5-4yrs | 2.5 | 4 | yes | Yes but Not Affected;Yes Bit affected;Yes MOD Affected;Yes & Affected Lot];No | No];Yes but Not Affected_Yes Bit affected_Yes MOD Affected_Yes & Affected Lot |
| pg3022 | C23: Degree to which physical cruelty from a partner affected partner since child was 2.5 years old | Quest_Partner | no | violence_between_parents | 3yrs11m | 2.5-4yrs | 2.5 | 4 | yes | A lot;Moderately;Mildly];Not at all;Did not happen | Did not happen];A lot_Moderately_Mildly_Not at all |

| **Table S4. ACE variables in late childhood (7-12 yrs).** | | | | | | | | | | | |
| --- | --- | --- | --- | --- | --- | --- | --- | --- | --- | --- | --- |
| **variable name** | **description** | **datasource** | **retrospective** | **ACE** | **time_alspac** | **time_period** | **time_yrs_start** | **time_yrs_end** | **reverse_scale** | **recode_ACE** | **factor_level_after_recode** |
| ccc290 | A20: Frequency child has been bullied | Quest_Child Completed | no | bullying | 97m | 8yrs | 8 | 8 | yes | All the time];Quite a lot;A little bit;Never | Quite a lot_A little bit_Never];All the time |
| f8fp141 | Bullying OR, Personal belongings stolen - Freq: F8 | Clinic_Child | no | bullying | 8.5yrs | 8.5yrs | 8.5 | 8.5 | no | 1-3 times in the past 6 mths;4+ times last 6 mths but <1/wk];At least 1/wk | FALSE_1-3 times in the past 6 mths_4+ times last 6 mths but <1/wk];At least 1/wk |
| f8fp151 | Bullying OR, Threatened/blackmailed - Freq: F8 | Clinic_Child | no | bullying | 8.5yrs | 8.5yrs | 8.5 | 8.5 | no | 1-3 times in the past 6 mths;4+ times last 6 mths but <1/wk];At least 1/wk | FALSE_1-3 times in the past 6 mths_4+ times last 6 mths but <1/wk];At least 1/wk |
| f8fp161 | Bullying OR, Hit/beaten up - Freq: F8 | Clinic_Child | no | bullying | 8.5yrs | 8.5yrs | 8.5 | 8.5 | no | 1-3 times in the past 6 mths;4+ times last 6 mths but <1/wk];At least 1/wk | FALSE_1-3 times in the past 6 mths_4+ times last 6 mths but <1/wk];At least 1/wk |
| f8fp341 | Bullying RR, Got to do something didn't want to - Freq: F8 | Clinic_Child | no | bullying | 8.5yrs | 8.5yrs | 8.5 | 8.5 | no | 1-3 times in the past 6 mths;4+ times last 6 mths but <1/wk];At least 1/wk | FALSE_1-3 times in the past 6 mths_4+ times last 6 mths but <1/wk];At least 1/wk |
| f8fp351 | Bullying RR, Told lies about - Freq: F8 | Clinic_Child | no | bullying | 8.5yrs | 8.5yrs | 8.5 | 8.5 | no | 1-3 times in the past 6 mths;4+ times last 6 mths but <1/wk];At least 1/wk | FALSE_1-3 times in the past 6 mths_4+ times last 6 mths but <1/wk];At least 1/wk |
| fdfp141 | Bullying OR, Personal belongings stolen - Freq: F10 | Clinic_Child | no | bullying | 10+yrs | 10yrs | 10 | 10 | special_no | 1-3 times in the past 6 mths;Freq, 4+ times last 6 mths but <1/wk];Very freq, at least 1/wk];Ch said DK | FALSE_Ch said DK_1-3 times in the past 6 mths_Freq, 4+ times last 6 mths but <1/wk];Very freq, at least 1/wk |
| fdfp151 | Bullying OR, Threatened/blackmailed - Freq: F10 | Clinic_Child | no | bullying | 10+yrs | 10yrs | 10 | 10 | special_no | 1-3 times in the past 6 mths;Freq, 4+ times last 6 mths but <1/wk];Very freq, at least 1/wk];Ch said DK | FALSE_Ch said DK_1-3 times in the past 6 mths_Freq, 4+ times last 6 mths but <1/wk];Very freq, at least 1/wk |
| fdfp161 | Bullying OR, Hit/beaten up - Freq: F10 | Clinic_Child | no | bullying | 10+yrs | 10yrs | 10 | 10 | special_no | 1-3 times in the past 6 mths;Freq, 4+ times last 6 mths but <1/wk];Very freq, at least 1/wk];Ch said DK | FALSE_Ch said DK_1-3 times in the past 6 mths_Freq, 4+ times last 6 mths but <1/wk];Very freq, at least 1/wk |
| fdfp341 | Bullying RR, Got to do something didn't want to - Freq: F10 | Clinic_Child | no | bullying | 10+yrs | 10yrs | 10 | 10 | special_no | 1-3 times in the past 6 mths;Freq, 4+ times last 6 mths but <1/wk];Very freq, at least 1/wk];Ch said DK | FALSE_Ch said DK_1-3 times in the past 6 mths_Freq, 4+ times last 6 mths but <1/wk];Very freq, at least 1/wk |
| fdfp351 | Bullying RR, Told lies about - Freq: F10 | Clinic_Child | no | bullying | 10+yrs | 10yrs | 10 | 10 | special_no | 1-3 times in the past 6 mths;Freq, 4+ times last 6 mths but <1/wk];Very freq, at least 1/wk];Ch said DK | FALSE_Ch said DK_1-3 times in the past 6 mths_Freq, 4+ times last 6 mths but <1/wk];Very freq, at least 1/wk |
| p2037 | B38: Mother's husband/partner was emotionally cruel to her children since the study child's 6th birthday | Quest_Mother | no | emotional_abuse | 9yrs2m | 8-9yrs | 8 | 9 | special_no | Yes, when the study child was 6 or 7];Yes, since the study child's 8th birthday;Yes, both when the study child was 6/7 and 8+];No, did not happen in past 3 years | No, did not happen in past 3 years_Yes, when the study child was 6 or 7];Yes, since the study child's 8th birthday_Yes, both when the study child was 6/7 and 8+ |
| p2038 | B39: Mother was emotionally cruel to her children since the study child's 6th birthday | Quest_Mother | no | emotional_abuse | 9yrs2m | 8-9yrs | 8 | 9 | special_no | Yes, when the study child was 6 or 7];Yes, since the study child's 8th birthday;Yes, both when the study child was 6/7 and 8+];No, did not happen in past 3 years | No, did not happen in past 3 years_Yes, when the study child was 6 or 7];Yes, since the study child's 8th birthday_Yes, both when the study child was 6/7 and 8+ |
| pm2037 | B38: Father's wife/partner was emotionally cruel to his children since the study child's 6th birthday | Quest_Partner | no | emotional_abuse | 9yrs2m | 8-9yrs | 8 | 9 | special_no | Yes, when the study child was 6 or 7];Yes, since the study child's 8th birthday;Yes, both when the study child was 6/7 and 8+];No, did not happen in past 3 years | No, did not happen in past 3 years_Yes, when the study child was 6 or 7];Yes, since the study child's 8th birthday_Yes, both when the study child was 6/7 and 8+ |
| pm2038 | B39: Father was emotionally cruel to his children since the study child's 6th birthday | Quest_Partner | no | emotional_abuse | 9yrs2m | 8-9yrs | 8 | 9 | special_no | Yes, when the study child was 6 or 7];Yes, since the study child's 8th birthday;Yes, both when the study child was 6/7 and 8+];No, did not happen in past 3 years | No, did not happen in past 3 years_Yes, when the study child was 6 or 7];Yes, since the study child's 8th birthday_Yes, both when the study child was 6/7 and 8+ |
| pp5037 | F38: Respondent's wife/partner has been emotionally cruel to their children since the study child's 9th birthday | Quest_Partner | yes | emotional_abuse | 11yrs2m | 9-10yrs | 9 | 10 | special_yes | Yes, when the study child was 9 or 10];Yes, since the child's 11th birthday];Yes, both when the study child was 9/10 and 11+];No, did not happen in this period | No, did not happen in this period_Yes, since the child's 11th birthday];Yes, when the study child was 9 or 10_Yes, both when the study child was 9/10 and 11+ |
| pp5038 | F39: Respondent has been emotionally cruel to their children since the study child's 9th birthday | Quest_Partner | yes | emotional_abuse | 11yrs2m | 9-10yrs | 9 | 10 | special_yes | Yes, when the study child was 9 or 10];Yes, since the child's 11th birthday];Yes, both when the study child was 9/10 and 11+];No, did not happen in this period | No, did not happen in this period_Yes, since the child's 11th birthday];Yes, when the study child was 9 or 10_Yes, both when the study child was 9/10 and 11+ |
| r5037 | F38: Respondent's husband/partner has been emotionally cruel to their children since study child's 9th birthday | Quest_Mother | yes | emotional_abuse | 11yrs2m | 9-10yrs | 9 | 10 | special_yes | Yes, when study child was 9 or 10];Yes, since child's 11th birthday];Yes, both when the study child was 10/11 & 11+];No, did not happen in this period | No, did not happen in this period_Yes, since child's 11th birthday];Yes, when study child was 9 or 10_Yes, both when the study child was 10/11 & 11+ |
| r5038 | F39: Respondent has been emotionally cruel to their children since study child's 9th birthday | Quest_Mother | yes | emotional_abuse | 11yrs2m | 9-10yrs | 9 | 10 | special_yes | Yes, when study child was 9 or 10];Yes, since child's 11th birthday];Yes, both when the study child was 10/11 & 11+];No, did not happen in this period | No, did not happen in this period_Yes, since child's 11th birthday];Yes, when study child was 9 or 10_Yes, both when the study child was 10/11 & 11+ |
| pp5037 | F38: Respondent's wife/partner has been emotionally cruel to their children since the study child's 9th birthday | Quest_Partner | no | emotional_abuse | 11yrs2m | 11yrs | 11 | 11 | special_no | Yes, when the study child was 9 or 10];Yes, since the child's 11th birthday;Yes, both when the study child was 9/10 and 11+];No, did not happen in this period | No, did not happen in this period_Yes, when the study child was 9 or 10];Yes, since the child's 11th birthday_Yes, both when the study child was 9/10 and 11+ |
| pp5038 | F39: Respondent has been emotionally cruel to their children since the study child's 9th birthday | Quest_Partner | no | emotional_abuse | 11yrs2m | 11yrs | 11 | 11 | special_no | Yes, when the study child was 9 or 10];Yes, since the child's 11th birthday;Yes, both when the study child was 9/10 and 11+];No, did not happen in this period | No, did not happen in this period_Yes, when the study child was 9 or 10];Yes, since the child's 11th birthday_Yes, both when the study child was 9/10 and 11+ |
| r5037 | F38: Respondent's husband/partner has been emotionally cruel to their children since study child's 9th birthday | Quest_Mother | no | emotional_abuse | 11yrs2m | 11yrs | 11 | 11 | special_no | Yes, when study child was 9 or 10];Yes, since child's 11th birthday;Yes, both when the study child was 10/11 & 11+];No, did not happen in this period | No, did not happen in this period_Yes, when study child was 9 or 10];Yes, since child's 11th birthday_Yes, both when the study child was 10/11 & 11+ |
| r5038 | F39: Respondent has been emotionally cruel to their children since study child's 9th birthday | Quest_Mother | no | emotional_abuse | 11yrs2m | 11yrs | 11 | 11 | special_no | Yes, when study child was 9 or 10];Yes, since child's 11th birthday;Yes, both when the study child was 10/11 & 11+];No, did not happen in this period | No, did not happen in this period_Yes, when study child was 9 or 10];Yes, both when the study child was 10/11 & 11+_Yes, since child's 11th birthday |
| ccc250 | A16: Frequency child feels left out of things | Quest_Child Completed | no | emotional_neglect | 97m | 8yrs | 8 | 8 | no | Never;Sometimes;Often];Always | Never_Sometimes_Often];Always |
| ccf104 | A5: Study child is understood by parent(s) | Quest_Child Completed | no | emotional_neglect | 116m | 9.5yrs | 9.5 | 9.5 | yes | Not true];Mostly untrue;Partly true;Mostly true;True | Mostly untrue_Partly true_Mostly true_True];Not true |
| p1054 | A3e: Frequency mother has taken pills for depression in last 2 years | Quest_Mother | no | mental_health_problems_or_suicide | 9yrs2m | 7-9yrs | 7 | 9 | yes | Every day;Often;Sometimes];Not at all | Not at all];Every day_Often_Sometimes |
| p3031 | C3l: Husband/partner has had schizophrenia, in last 2 years | Quest_Mother | no | mental_health_problems_or_suicide | 9yrs2m | 7-9yrs | 7 | 9 | yes | Yes, saw doctor;Yes, did not see doctor];No;Do not know | No_Do not know];Yes, saw doctor_Yes, did not see doctor |
| pm1054 | A3e: Frequency father has taken pills for depression in last 2 years | Quest_Partner | no | mental_health_problems_or_suicide | 9yrs2m | 7-9yrs | 7 | 9 | yes | Every day;Often;Sometimes];Not at all | Not at all];Every day_Often_Sometimes |
| pm3031 | C3l: Wife/partner has had schizophrenia, in last 2 years | Quest_Partner | no | mental_health_problems_or_suicide | 9yrs2m | 7-9yrs | 7 | 9 | yes | Yes, saw doctor;Yes, did not see doctor];No;Do not know | No_Do not know];Yes, saw doctor_Yes, did not see doctor |
| n1042 | A3c: Mother has ever had bulimia | Quest_Mother | no | mental_health_problems_or_suicide | 8yrs1m | 8yrs | 8 | 8 | yes | Yes, recently];Yes, in past;No | No_Yes, in past];Yes, recently |
| n1059 | A3t: Mother has ever had schizophrenia | Quest_Mother | no | mental_health_problems_or_suicide | 8yrs1m | 8yrs | 8 | 8 | yes | Yes, recently;Yes, in past];No | No_Yes, in past];Yes, recently |
| n1060 | A3u: Mother has ever had anorexia nervosa | Quest_Mother | no | mental_health_problems_or_suicide | 8yrs1m | 8yrs | 8 | 8 | yes | Yes, recently];Yes, in past;No | No_Yes, in past];Yes, recently |
| pl1042 | A3c: Respondent has ever had bulimia | Quest_Partner | no | mental_health_problems_or_suicide | 8yrs1m | 8yrs | 8 | 8 | yes | Yes, recently];Yes, in past not now;No, never | No, never_Yes, in past not now];Yes, recently |
| p2028 | B29: Mother attempted suicide since the study child's 6th birthday | Quest_Mother | no | mental_health_problems_or_suicide | 9yrs2m | 8-9yrs | 8 | 9 | special_no | Yes, when the study child was 6 or 7];Yes, since the study child's 8th birthday;Yes, both when the study child was 6/7 and 8+];No, did not happen in past 3 years | No, did not happen in past 3 years_Yes, when the study child was 6 or 7];Yes, since the study child's 8th birthday_Yes, both when the study child was 6/7 and 8+ |
| pm2028 | B29: Father attempted suicide since the study child's 6th birthday | Quest_Partner | no | mental_health_problems_or_suicide | 9yrs2m | 8-9yrs | 8 | 9 | special_no | Yes, when the study child was 6 or 7];Yes, since the study child's 8th birthday];No, did not happen in past 3 years | No, did not happen in past 3 years_Yes, when the study child was 6 or 7];Yes, since the study child's 8th birthday |
| pn4100 | B1k: Respondent has taken medicines for depression in the past 12 months | Quest_Partner | no | mental_health_problems_or_suicide | 10yrs2m | 9-10yrs | 9 | 10 | no | Yes | FALSE];Yes |
| pn4110 | B1l: Respondent has taken medicines for anxiety/nerves in the past 12 months | Quest_Partner | no | mental_health_problems_or_suicide | 10yrs2m | 9-10yrs | 9 | 10 | no | Yes | FALSE];Yes |
| q4100 | D1k: Mother used medicine in last 12 months for depression | Quest_Mother | no | mental_health_problems_or_suicide | 10yrs2m | 9-10yrs | 9 | 10 | no | Yes | FALSE];Yes |
| q4110 | D1l: Mother used medicine in last 12 months for anxiety/nerves | Quest_Mother | no | mental_health_problems_or_suicide | 10yrs2m | 9-10yrs | 9 | 10 | no | Yes | FALSE];Yes |
| r5028 | F29: Respondent attempted suicide since the study child's 9th birthday | Quest_Mother | yes | mental_health_problems_or_suicide | 11yrs2m | 9-10yrs | 9 | 10 | special_yes | Yes, when study child was 9 or 10];Yes, since child's 11th birthday];Yes, both when the study child was 10/11 & 11+];No, did not happen in this period | No, did not happen in this period_Yes, since child's 11th birthday];Yes, when study child was 9 or 10_Yes, both when the study child was 10/11 & 11+ |
| pp5028 | F29: Respondent attempted suicide since the study child's 9th birthday | Quest_Partner | yes | mental_health_problems_or_suicide | 11yrs2m | 9-10yrs | 9 | 10 | yes | Yes, when the study child was 9 or 10];Yes, since the child's 11th birthday;No, did not happen in this period | Yes, since the child's 11th birthday_No, did not happen in this period];Yes, when the study child was 9 or 10 |
| pp5028 | F29: Respondent attempted suicide since the study child's 9th birthday | Quest_Partner | no | mental_health_problems_or_suicide | 11yrs2m | 11yrs | 11 | 11 | special_no | Yes, when the study child was 9 or 10];Yes, since the child's 11th birthday];No, did not happen in this period | No, did not happen in this period_Yes, when the study child was 9 or 10];Yes, since the child's 11th birthday_Yes, both when the study child was 9/10 and 11+ |
| r5028 | F29: Respondent attempted suicide since the study child's 9th birthday | Quest_Mother | no | mental_health_problems_or_suicide | 11yrs2m | 11yrs | 11 | 11 | special_no | Yes, when study child was 9 or 10];Yes, since child's 11th birthday;Yes, both when the study child was 10/11 & 11+];No, did not happen in this period | No, did not happen in this period_Yes, when study child was 9 or 10];Yes, since child's 11th birthday_Yes, both when the study child was 10/11 & 11+ |
| pq1020 | A2k: Partner has had schizophrenia in the last two years | Quest_Partner | no | mental_health_problems_or_suicide | 12yrs1m | 10-12yrs | 10 | 12 | yes | Yes and consulted doctor];No | No];Yes and consulted doctor |
| pq3031 | C3l: Partner's partner had schizophrenia in the last 2 years | Quest_Partner | no | mental_health_problems_or_suicide | 12yrs1m | 10-12yrs | 10 | 12 | yes | No, not at all;Do not know | No, not at all_Do not know |
| s1020 | A2k: Mother has had schizophrenia in last 2 years | Quest_Mother | no | mental_health_problems_or_suicide | 12yrs1m | 10-12yrs | 10 | 12 | yes | Yes, consulted doctor;Yes, did not consult doctor];No | No];Yes, consulted doctor_Yes, did not consult doctor |
| s3031 | C3l: Mother's partner has had schizophrenia since study child's 10th birthday | Quest_Mother | no | mental_health_problems_or_suicide | 12yrs1m | 10-12yrs | 10 | 12 | yes | Yes, saw a doctor;Yes, did not see doctor];No;Do not know | No_Do not know];Yes, saw a doctor_Yes, did not see doctor |
| pp2002 | B1c: Respondent has ever had bulimia | Quest_Partner | no | mental_health_problems_or_suicide | 11yrs2m | 11yrs | 11 | 11 | yes | Yes, had it recently];Yes, in past, not recently;No, never | No, never_Yes, in past, not recently];Yes, had it recently |
| pp2019 | B1s: Respondent has ever had schizophrenia | Quest_Partner | no | mental_health_problems_or_suicide | 11yrs2m | 11yrs | 11 | 11 | yes | Yes, had it recently;Yes, in past, not recently];No, never | No, never_Yes, in past, not recently];Yes, had it recently |
| pp2020 | B1t: Respondent has ever had anorexia nervosa | Quest_Partner | no | mental_health_problems_or_suicide | 11yrs2m | 11yrs | 11 | 11 | yes | Yes, had it recently];Yes, in past, not recently;No, never | No, never_Yes, in past, not recently];Yes, had it recently |
| r2002 | B1c: Respondent has ever had bulimia | Quest_Mother | no | mental_health_problems_or_suicide | 11yrs2m | 11yrs | 11 | 11 | yes | Yes, had it recently(in past year)];Yes, in past, not recently;No, never | No, never_Yes, in past, not recently];Yes, had it recently(in past year) |
| r2019 | B1t: Respondent has ever had schizophrenia | Quest_Mother | no | mental_health_problems_or_suicide | 11yrs2m | 11yrs | 11 | 11 | yes | Yes, had it recently(in past year);Yes, in past, not recently];No, never | No, never_Yes, in past, not recently];Yes, had it recently(in past year) |
| r2020 | B1u: Respondent has ever had anorexia nervosa | Quest_Mother | no | mental_health_problems_or_suicide | 11yrs2m | 11yrs | 11 | 11 | yes | Yes, had it recently(in past year)];Yes, in past, not recently;No, never | No, never_Yes, in past, not recently];Yes, had it recently(in past year) |
| pq4100 | D1k: Partner has used medicine for depression in the last 12 months | Quest_Partner | no | mental_health_problems_or_suicide | 12yrs1m | 11-12yrs | 11 | 12 | no | Yes | FALSE];Yes |
| pq4110 | D1l: Partner has used medicine for anxiety or nerves in the last 12 months | Quest_Partner | no | mental_health_problems_or_suicide | 12yrs1m | 11-12yrs | 11 | 12 | no | Yes | FALSE];Yes |
| s4100 | D1k: Mother has taken medication for depression in the past 12 months | Quest_Mother | no | mental_health_problems_or_suicide | 12yrs1m | 11-12yrs | 11 | 12 | no | Yes | FALSE];Yes |
| s4110 | D1l: Mother has taken medication for anxiety or nerves in the past 12 months | Quest_Mother | no | mental_health_problems_or_suicide | 12yrs1m | 11-12yrs | 11 | 12 | no | Yes | FALSE];Yes |
| pm1020 | A2k: Father has had schizophrenia in last 3 years | Quest_Partner | no | mental_health_problems_or_suicide | 9yrs2m | 6-9yrs | 6 | 9 | no | No | No |
| p1020 | A2k: Mother has had schizophrenia in last 3 years | Quest_Mother | no | mental_health_problems_or_suicide | 9yrs2m | 6-9yrs | 6 | 9 | yes | Yes, consulted doctor;Yes, did not consult doctor];No | No];Yes, consulted doctor_Yes, did not consult doctor |
| n8377 | J20h: Mother feels very close to study child | Quest_Mother | no | parent_child_bond | 8yrs1m | 8yrs | 8 | 8 | special_no | Yes];No];Sometimes / occasionally | Sometimes / occasionally_Yes];No |
| n8387 | J21h: Partner is very close to study child | Quest_Mother | no | parent_child_bond | 8yrs1m | 8yrs | 8 | 8 | special_no | Yes];No];Sometimes / occasionally | Sometimes / occasionally_Yes];No |
| p3054 | C4e: Mother feels father/partner seems to feel very close to the study child | Quest_Mother | no | parent_child_bond | 9yrs2m | 9yrs | 9 | 9 | no | Yes, always feel;Yes, sometimes feel];No, never feel | Yes, always feel_Yes, sometimes feel];No, never feel |
| pm3054 | C4e: Father feels wife/partner seems to feel very close to the study child | Quest_Partner | no | parent_child_bond | 9yrs2m | 9yrs | 9 | 9 | no | Yes, always feel;Yes, sometimes feel];No, never feel | Yes, always feel_Yes, sometimes feel];No, never feel |
| pq3054 | C4e: Partner's partner seems to feel very close to study child | Quest_Partner | no | parent_child_bond | 12yrs1m | 12yrs | 12 | 12 | no | Partner always feels this way;Partner sometimes feels this way];Partner never feels this way | Partner always feels this way_Partner sometimes feels this way];Partner never feels this way |
| s3054 | C4e: Mother's partner seems very close to study child | Quest_Mother | no | parent_child_bond | 12yrs1m | 12yrs | 12 | 12 | no | Always feels this;Sometimes feels this];Never feels this | Always feels this_Sometimes feels this];Never feels this |
| p2029 | B30: Mother was convicted of an offence since the study child's 6th birthday | Quest_Mother | no | parent_convicted_offence | 9yrs2m | 8-9yrs | 8 | 9 | special_no | Yes, when the study child was 6 or 7];Yes, since the study child's 8th birthday];No, did not happen in past 3 years | No, did not happen in past 3 years_Yes, when the study child was 6 or 7];Yes, since the study child's 8th birthday |
| pm2029 | B30: Father was convicted of an offence since the study child's 6th birthday | Quest_Partner | no | parent_convicted_offence | 9yrs2m | 8-9yrs | 8 | 9 | special_no | Yes, when the study child was 6 or 7];Yes, since the study child's 8th birthday];No, did not happen in past 3 years | No, did not happen in past 3 years_Yes, when the study child was 6 or 7];Yes, since the study child's 8th birthday |
| pp5029 | F30: Respondent has been convicted of an offence since the study child's 9th birthday | Quest_Partner | yes | parent_convicted_offence | 11yrs2m | 9-10yrs | 9 | 10 | yes | Yes, when the study child was 9 or 10];Yes, since the child's 11th birthday;No, did not happen in this period | No, did not happen in this period_Yes, since the child's 11th birthday];Yes, when the study child was 9 or 10_Yes, both when the study child was 9/10 and 11+ |
| r5029 | F30: Respondent was convicted of an offence since study child's 9th birthday | Quest_Mother | yes | parent_convicted_offence | 11yrs2m | 9-10yrs | 9 | 10 | yes | Yes, when study child was 9 or 10];Yes, since child's 11th birthday;No, did not happen in this period | No, did not happen in this period_Yes, since child's 11th birthday];Yes, when study child was 9 or 10 |
| pp5029 | F30: Respondent has been convicted of an offence since the study child's 9th birthday | Quest_Partner | no | parent_convicted_offence | 11yrs2m | 11yrs | 11 | 11 | special_no | Yes, when the study child was 9 or 10];Yes, since the child's 11th birthday];No, did not happen in this period | No, did not happen in this period_Yes, when the study child was 9 or 10];Yes, since the child's 11th birthday_Yes, both when the study child was 9/10 and 11+ |
| r5029 | F30: Respondent was convicted of an offence since study child's 9th birthday | Quest_Mother | no | parent_convicted_offence | 11yrs2m | 11yrs | 11 | 11 | special_no | Yes, when study child was 9 or 10];Yes, since child's 11th birthday];No, did not happen in this period | No, did not happen in this period_Yes, when study child was 9 or 10];Yes, since child's 11th birthday |
| pq5014 | E2c: Partner has been convicted of an offence other than speeding in the last year | Quest_Partner | no | parent_convicted_offence | 12yrs1m | 11-12yrs | 11 | 12 | yes | Yes];No | No_FALSE];Yes |
| s5014 | E2c: Mother was convicted of an offence in the last year | Quest_Mother | no | parent_convicted_offence | 12yrs1m | 11-12yrs | 11 | 12 | yes | Yes];No | No_FALSE];Yes |
| p2008 | B9: Mother was divorced since the study child's 6th birthday | Quest_Mother | no | parental_separation | 9yrs2m | 8-9yrs | 8 | 9 | special_no | Yes, when the study child was 6 or 7];Yes, since the study child's 8th birthday;Yes, both when the study child was 6/7 and 8+];No, did not happen in past 3 years | No, did not happen in past 3 years_Yes, when the study child was 6 or 7];Yes, since the study child's 8th birthday_Yes, both when the study child was 6/7 and 8+ |
| p2017 | B18: Mother and husband/partner separated since the study child's 6th birthday | Quest_Mother | no | parental_separation | 9yrs2m | 8-9yrs | 8 | 9 | special_no | Yes, when the study child was 6 or 7];Yes, since the study child's 8th birthday;Yes, both when the study child was 6/7 and 8+];No, did not happen in past 3 years | No, did not happen in past 3 years_Yes, when the study child was 6 or 7];Yes, since the study child's 8th birthday_Yes, both when the study child was 6/7 and 8+ |
| pm2008 | B9: Father was divorced since the study child's 6th birthday | Quest_Partner | no | parental_separation | 9yrs2m | 8-9yrs | 8 | 9 | special_no | Yes, when the study child was 6 or 7];Yes, since the study child's 8th birthday;Yes, both when the study child was 6/7 and 8+];No, did not happen in past 3 years | No, did not happen in past 3 years_Yes, when the study child was 6 or 7];Yes, since the study child's 8th birthday_Yes, both when the study child was 6/7 and 8+ |
| pm2017 | B18: Father and wife/partner separated since the study child's 6th birthday | Quest_Partner | no | parental_separation | 9yrs2m | 8-9yrs | 8 | 9 | special_no | Yes, when the study child was 6 or 7];Yes, since the study child's 8th birthday;Yes, both when the study child was 6/7 and 8+];No, did not happen in past 3 years | No, did not happen in past 3 years_Yes, when the study child was 6 or 7];Yes, since the study child's 8th birthday_Yes, both when the study child was 6/7 and 8+ |
| pp5008 | F9: Respondent has divorced since the study child's 9th birthday | Quest_Partner | yes | parental_separation | 11yrs2m | 9-10yrs | 9 | 10 | special_yes | Yes, when the study child was 9 or 10];Yes, since the child's 11th birthday];Yes, both when the study child was 9/10 and 11+];No, did not happen in this period | No, did not happen in this period_Yes, since the child's 11th birthday];Yes, when the study child was 9 or 10_Yes, both when the study child was 9/10 and 11+ |
| pp5015 | F16: Respondent's wife/partner went away since the study child's 9th birthday | Quest_Partner | yes | parental_separation | 11yrs2m | 9-10yrs | 9 | 10 | special_yes | Yes, when the study child was 9 or 10];Yes, since the child's 11th birthday];Yes, both when the study child was 9/10 and 11+];No, did not happen in this period | No, did not happen in this period_Yes, since the child's 11th birthday];Yes, when the study child was 9 or 10_Yes, both when the study child was 9/10 and 11+ |
| pp5017 | F18: Respondent has separated from wife/partner since the study child's 9th birthday | Quest_Partner | yes | parental_separation | 11yrs2m | 9-10yrs | 9 | 10 | special_yes | Yes, when the study child was 9 or 10];Yes, since the child's 11th birthday];Yes, both when the study child was 9/10 and 11+];No, did not happen in this period | No, did not happen in this period_Yes, since the child's 11th birthday];Yes, when the study child was 9 or 10_Yes, both when the study child was 9/10 and 11+ |
| r5008 | F9: Respondent has been divorced since child's 9th birthday | Quest_Mother | yes | parental_separation | 11yrs2m | 9-10yrs | 9 | 10 | special_yes | Yes, when study child was 9 or 10];Yes, since child's 11th birthday];Yes, both when the study child was 10/11 & 11+];No, did not happen in this period | No, did not happen in this period_Yes, since the child's 11th birthday];Yes, when the study child was 9 or 10_Yes, both when the study child was 9/10 and 11+ |
| r5017 | F18: Respondent separated from husband/partner since the study child's 9th birthday | Quest_Mother | yes | parental_separation | 11yrs2m | 9-10yrs | 9 | 10 | special_yes | Yes, when study child was 9 or 10];Yes, since child's 11th birthday];Yes, both when the study child was 10/11 & 11+];No, did not happen in this period | No, did not happen in this period_Yes, since the child's 11th birthday];Yes, when the study child was 9 or 10_Yes, both when the study child was 9/10 and 11+ |
| pq3003 | C1d: Partner's current partner is the same as on the study child's 9th birthday | Quest_Partner | no | parental_separation | 12yrs1m | 9-12yrs | 9 | 12 | no | Yes the same];No, a new partner;Don't remember | Yes the same];No, a new partner_Don't remember |
| s3003 | C1d: Mother's current partner is the same as on study child's 9th birthday | Quest_Mother | no | parental_separation | 12yrs1m | 9-12yrs | 9 | 12 | no | Yes];No;Don't remember | Yes];No_Don't remember |
| pp5008 | F9: Respondent has divorced since the study child's 9th birthday | Quest_Partner | no | parental_separation | 11yrs2m | 11yrs | 11 | 11 | special_no | Yes, when the study child was 9 or 10];Yes, since the child's 11th birthday;Yes, both when the study child was 9/10 and 11+];No, did not happen in this period | No, did not happen in this period_Yes, when the study child was 9 or 10];Yes, since the child's 11th birthday_Yes, both when the study child was 9/10 and 11+ |
| pp5015 | F16: Respondent's wife/partner went away since the study child's 9th birthday | Quest_Partner | no | parental_separation | 11yrs2m | 11yrs | 11 | 11 | special_no | Yes, when the study child was 9 or 10];Yes, since the child's 11th birthday;Yes, both when the study child was 9/10 and 11+];No, did not happen in this period | No, did not happen in this period_Yes, when the study child was 9 or 10];Yes, since the child's 11th birthday_Yes, both when the study child was 9/10 and 11+ |
| pp5017 | F18: Respondent has separated from wife/partner since the study child's 9th birthday | Quest_Partner | no | parental_separation | 11yrs2m | 11yrs | 11 | 11 | special_no | Yes, when the study child was 9 or 10];Yes, since the child's 11th birthday;Yes, both when the study child was 9/10 and 11+];No, did not happen in this period | No, did not happen in this period_Yes, when the study child was 9 or 10];Yes, since the child's 11th birthday_Yes, both when the study child was 9/10 and 11+ |
| r5008 | F9: Respondent has been divorced since child's 9th birthday | Quest_Mother | no | parental_separation | 11yrs2m | 11yrs | 11 | 11 | special_no | Yes, when study child was 9 or 10];Yes, since child's 11th birthday;Yes, both when the study child was 10/11 & 11+];No, did not happen in this period | No, did not happen in this period_Yes, when study child was 9 or 10];Yes, since child's 11th birthday_Yes, both when the study child was 10/11 & 11+ |
| r5017 | F18: Respondent separated from husband/partner since the study child's 9th birthday | Quest_Mother | no | parental_separation | 11yrs2m | 11yrs | 11 | 11 | special_no | Yes, when study child was 9 or 10];Yes, since child's 11th birthday;Yes, both when the study child was 10/11 & 11+];No, did not happen in this period | No, did not happen in this period_Yes, when study child was 9 or 10];Yes, since child's 11th birthday_Yes, both when the study child was 10/11 & 11+ |
| p3003 | C1d: Same partner/husband as mother had when study child had 6th birthday | Quest_Mother | no | parental_separation | 9yrs2m | 6-9yrs | 6 | 9 | no | Yes];No;Don't remember | Yes];No_Don't remember |
| pm3003 | C1d: Same partner/wife as father had when study child had 6th birthday | Quest_Partner | no | parental_separation | 9yrs2m | 6-9yrs | 6 | 9 | no | Yes];No;Don't remember | FALSE_Yes];No_Don't remember |
| p2026 | B27: Mother's husband/partner was physically cruel to her children since the study child's 6th birthday | Quest_Mother | no | physical_abuse | 9yrs2m | 8-9yrs | 8 | 9 | special_no | Yes, when the study child was 6 or 7];Yes, since the study child's 8th birthday;Yes, both when the study child was 6/7 and 8+];No, did not happen in past 3 years | No, did not happen in past 3 years_Yes, when the study child was 6 or 7];Yes, since the study child's 8th birthday_Yes, both when the study child was 6/7 and 8+ |
| p2027 | B28: Mother was physically cruel to her children since the study child's 6th birthday | Quest_Mother | no | physical_abuse | 9yrs2m | 8-9yrs | 8 | 9 | special_no | Yes, when the study child was 6 or 7];Yes, since the study child's 8th birthday;Yes, both when the study child was 6/7 and 8+];No, did not happen in past 3 years | No, did not happen in past 3 years_Yes, when the study child was 6 or 7];Yes, since the study child's 8th birthday_Yes, both when the study child was 6/7 and 8+ |
| pm2026 | B27: Father's wife/partner was physically cruel to his children since the study child's 6th birthday | Quest_Partner | no | physical_abuse | 9yrs2m | 8-9yrs | 8 | 9 | yes | Yes, since the study child's 8th birthday;Yes, both when the study child was 6/7 and 8+];No, did not happen in past 3 years | No, did not happen in past 3 years];Yes, since the study child's 8th birthday_Yes, both when the study child was 6/7 and 8+ |
| pm2027 | B28: Father was physically cruel to his children since the study child's 6th birthday | Quest_Partner | no | physical_abuse | 9yrs2m | 8-9yrs | 8 | 9 | yes | Yes, since the study child's 8th birthday;Yes, both when the study child was 6/7 and 8+];No, did not happen in past 3 years | No, did not happen in past 3 years];Yes, since the study child's 8th birthday_Yes, both when the study child was 6/7 and 8+ |
| pp5026 | F27: Respondent's wife/partner was physically cruel to their children since the study child's 9th birthday | Quest_Partner | yes | physical_abuse | 11yrs2m | 9-10yrs | 9 | 10 | special_yes | Yes, when the study child was 9 or 10];Yes, since the child's 11th birthday];Yes, both when the study child was 9/10 and 11+];No, did not happen in this period | No, did not happen in this period_Yes, since the child's 11th birthday];Yes, when the study child was 9 or 10_Yes, both when the study child was 9/10 and 11+ |
| pp5027 | F28: Respondent was physically cruel to their children since the study child's 9th birthday | Quest_Partner | yes | physical_abuse | 11yrs2m | 9-10yrs | 9 | 10 | special_yes | Yes, when the study child was 9 or 10];Yes, since the child's 11th birthday];Yes, both when the study child was 9/10 and 11+];No, did not happen in this period | No, did not happen in this period_Yes, since the child's 11th birthday];Yes, when the study child was 9 or 10_Yes, both when the study child was 9/10 and 11+ |
| r5026 | F27: Respondent's husband/partner was physically cruel to their children since study child's 9th birthday | Quest_Mother | yes | physical_abuse | 11yrs2m | 9-10yrs | 9 | 10 | special_yes | Yes, when study child was 9 or 10];Yes, since child's 11th birthday];Yes, both when the study child was 10/11 & 11+];No, did not happen in this period | No, did not happen in this period_Yes, since the child's 11th birthday];Yes, when the study child was 9 or 10_Yes, both when the study child was 9/10 and 11+ |
| r5027 | F28: Respondent was physically cruel to their children since the study child's 9th birthday | Quest_Mother | yes | physical_abuse | 11yrs2m | 9-10yrs | 9 | 10 | special_yes | Yes, when study child was 9 or 10];Yes, since child's 11th birthday];Yes, both when the study child was 10/11 & 11+];No, did not happen in this period | No, did not happen in this period_Yes, since the child's 11th birthday];Yes, when the study child was 9 or 10_Yes, both when the study child was 9/10 and 11+ |
| pp5026 | F27: Respondent's wife/partner was physically cruel to their children since the study child's 9th birthday | Quest_Partner | no | physical_abuse | 11yrs2m | 11yrs | 11 | 11 | special_no | Yes, when the study child was 9 or 10];Yes, since the child's 11th birthday;Yes, both when the study child was 9/10 and 11+];No, did not happen in this period | No, did not happen in this period_Yes, when the study child was 9 or 10];Yes, since the child's 11th birthday_Yes, both when the study child was 9/10 and 11+ |
| pp5027 | F28: Respondent was physically cruel to their children since the study child's 9th birthday | Quest_Partner | no | physical_abuse | 11yrs2m | 11yrs | 11 | 11 | special_no | Yes, when the study child was 9 or 10];Yes, since the child's 11th birthday;Yes, both when the study child was 9/10 and 11+];No, did not happen in this period | No, did not happen in this period_Yes, when the study child was 9 or 10];Yes, since the child's 11th birthday_Yes, both when the study child was 9/10 and 11+ |
| r5026 | F27: Respondent's husband/partner was physically cruel to their children since study child's 9th birthday | Quest_Mother | no | physical_abuse | 11yrs2m | 11yrs | 11 | 11 | special_no | Yes, when study child was 9 or 10];Yes, since child's 11th birthday;Yes, both when the study child was 10/11 & 11+];No, did not happen in this period | No, did not happen in this period_Yes, when study child was 9 or 10];Yes, since child's 11th birthday_Yes, both when the study child was 10/11 & 11+ |
| r5027 | F28: Respondent was physically cruel to their children since the study child's 9th birthday | Quest_Mother | no | physical_abuse | 11yrs2m | 11yrs | 11 | 11 | special_no | Yes, when study child was 9 or 10];Yes, since child's 11th birthday;Yes, both when the study child was 10/11 & 11+];No, did not happen in this period | No, did not happen in this period_Yes, when study child was 9 or 10];Yes, since child's 11th birthday_Yes, both when the study child was 10/11 & 11+ |
| kt5005 | E6: Since 7th birthday child has been sexually abused | Quest_Child Based | no | sexual_abuse | 105m | 7-9yrs | 7 | 9 | yes | Yes, very upset;Yes, quite upset;Yes, bit upset];No | No];Yes, very upset_Yes, quite upset_Yes, bit upset |
| p1052 | A3c: Frequency mother has taken cannabis/marihuana in last 2 years | Quest_Mother | no | substance_household | 9yrs2m | 7-9yrs | 7 | 9 | yes | Every day];Often;Sometimes;Not at all;Once | Often_Sometimes_Not at all_Once];Every day |
| p1060 | A3k: Frequency mother has taken amphetamines or other stimulants in last 2 years | Quest_Mother | no | substance_household | 9yrs2m | 7-9yrs | 7 | 9 | yes | Every day;Often;Sometimes];Not at all | Not at all];Every day_Often_Sometimes |
| p1063 | A3n: Frequency mother has taken heroin, methadone, crack, cocaine in last 2 years | Quest_Mother | no | substance_household | 9yrs2m | 7-9yrs | 7 | 9 | yes | Every day;Often;Sometimes];Not at all | Not at all];Every day_Often_Sometimes |
| p3032 | C3m: Husband/partner has had drink (alcohol) problem, in last 2 years | Quest_Mother | no | substance_household | 9yrs2m | 7-9yrs | 7 | 9 | yes | Yes, saw doctor];Yes, did not see doctor;No;Do not know | Yes, did not see doctor_No_Do not know];Yes, saw doctor |
| pm1052 | A3c: Frequency father has taken cannabis/marihuana in last 2 years | Quest_Partner | no | substance_household | 9yrs2m | 7-9yrs | 7 | 9 | yes | Every day];Often;Sometimes;Not at all | Often_Sometimes_Not at all];Every day |
| pm1060 | A3j: Frequency father has taken amphetamines or other stimulants in last 2 years | Quest_Partner | no | substance_household | 9yrs2m | 7-9yrs | 7 | 9 | yes | Often;Sometimes];Not at all | Not at all];Often_Sometimes |
| pm1063 | A3l: Frequency father has taken heroin, methadone, crack, cocaine in last 2 years | Quest_Partner | no | substance_household | 9yrs2m | 7-9yrs | 7 | 9 | yes | Every day;Often;Sometimes];Not at all | Not at all];Every day_Often_Sometimes |
| pm3032 | C3m: Wife/partner has had drink (alcohol) problem, in last 2 years | Quest_Partner | no | substance_household | 9yrs2m | 7-9yrs | 7 | 9 | yes | Yes, saw doctor];Yes, did not see doctor;No;Do not know | Yes, did not see doctor_No_Do not know];Yes, saw doctor |
| n1057 | A3r: Mother has ever had a drug addiction | Quest_Mother | no | substance_household | 8yrs1m | 8yrs | 8 | 8 | yes | Yes, recently];Yes, in past;No | Yes, in past_No];Yes, recently |
| n1058 | A3s: Mother has ever had alcoholism | Quest_Mother | no | substance_household | 8yrs1m | 8yrs | 8 | 8 | yes | Yes, recently];Yes, in past;No | Yes, in past_No];Yes, recently |
| pl1057 | A3q: Respondent has ever had drug addiction | Quest_Partner | no | substance_household | 8yrs1m | 8yrs | 8 | 8 | yes | Yes, recently];Yes, in past not now;No, never | Yes, in past not now_No, never];Yes, recently |
| r2017 | B1r: Respondent has ever had a drug addiction | Quest_Mother | no | substance_household | 11yrs2m | 10-11yrs | 10 | 11 | yes | Yes, had it recently(in past year)];Yes, in past, not recently;No, never | Yes, in past, not recently_No, never];Yes, had it recently(in past year) |
| pq1022 | A2m: Partner has had an alcohol problem in the last two years | Quest_Partner | no | substance_household | 12yrs1m | 10-12yrs | 10 | 12 | yes | Yes and consulted doctor];Yes but did not consult doctor;No | Yes but did not consult doctor_No];Yes and consulted doctor |
| pq3032 | C3m: Partner's partner had a drink problem in the last 2 years | Quest_Partner | no | substance_household | 12yrs1m | 10-12yrs | 10 | 12 | yes | Yes, and saw a doctor];Yes, but did not see a doctor;No, not at all;Do not know | Yes, but did not see a doctor_No, not at all_Do not know];Yes, and saw a doctor |
| s1022 | A2m: Mother has had an alcohol problem in last 2 years | Quest_Mother | no | substance_household | 12yrs1m | 10-12yrs | 10 | 12 | yes | Yes, consulted doctor];Yes, did not consult doctor;No | Yes, did not consult doctor_No];Yes, consulted doctor |
| s3032 | C3m: Mother's partner has had an alcohol problem since study child's 10th birthday | Quest_Mother | no | substance_household | 12yrs1m | 10-12yrs | 10 | 12 | yes | Yes, saw a doctor];Yes, did not see doctor;No;Do not know | Yes, did not see doctor_No_Do not know];Yes, saw a doctor |
| pp2017 | B1q: Respondent has ever had a drug addiction | Quest_Partner | no | substance_household | 11yrs2m | 11yrs | 11 | 11 | yes | Yes, had it recently];Yes, in past, not recently;No, never | Yes, in past, not recently_No, never];Yes, had it recently |
| p1022 | A2m: Mother has had alcohol problem in last 3 years | Quest_Mother | no | substance_household | 9yrs2m | 6-9yrs | 6 | 9 | yes | Yes, consulted doctor];Yes, did not consult doctor;No | Yes, did not consult doctor_No];Yes, consulted doctor |
| pm1022 | A2m: Father has had alcohol problem in last 3 years | Quest_Partner | no | substance_household | 9yrs2m | 6-9yrs | 6 | 9 | yes | Yes, consulted doctor];Yes, did not consult doctor;No | Yes, did not consult doctor_No];Yes, consulted doctor |
| p2022 | B23: Mother's husband/partner was physically cruel to her since the study child's 6th birthday | Quest_Mother | no | violence_between_parents | 9yrs2m | 8-9yrs | 8 | 9 | special_no | Yes, when the study child was 6 or 7];Yes, since the study child's 8th birthday;Yes, both when the study child was 6/7 and 8+];No, did not happen in past 3 years | No, did not happen in past 3 years_Yes, when the study child was 6 or 7];Yes, since the study child's 8th birthday_Yes, both when the study child was 6/7 and 8+ |
| pm2022 | B23: Father's wife/partner was physically cruel to him since the study child's 6th birthday | Quest_Partner | no | violence_between_parents | 9yrs2m | 8-9yrs | 8 | 9 | special_no | Yes, when the study child was 6 or 7];Yes, since the study child's 8th birthday;Yes, both when the study child was 6/7 and 8+];No, did not happen in past 3 years | No, did not happen in past 3 years_Yes, when the study child was 6 or 7];Yes, since the study child's 8th birthday_Yes, both when the study child was 6/7 and 8+ |
| pp5022 | F23: Respondent's wife/partner was physically cruel to them since the study child's 9th birthday | Quest_Partner | yes | violence_between_parents | 11yrs2m | 9-10yrs | 9 | 10 | special_yes | Yes, when the study child was 9 or 10];Yes, since the child's 11th birthday];Yes, both when the study child was 9/10 and 11+];No, did not happen in this period | No, did not happen in this period_Yes, since the child's 11th birthday];Yes, when the study child was 9 or 10_Yes, both when the study child was 9/10 and 11+ |
| r5022 | F23: Respondent's husband/partner was physically cruel to them since study child's 9th birthday | Quest_Mother | yes | violence_between_parents | 11yrs2m | 9-10yrs | 9 | 10 | special_yes | Yes, when study child was 9 or 10];Yes, since child's 11th birthday];Yes, both when the study child was 10/11 & 11+];No, did not happen in this period | No, did not happen in this period_Yes, since the child's 11th birthday];Yes, when the study child was 9 or 10_Yes, both when the study child was 9/10 and 11+ |
| pp5022 | F23: Respondent's wife/partner was physically cruel to them since the study child's 9th birthday | Quest_Partner | no | violence_between_parents | 11yrs2m | 11yrs | 11 | 11 | special_no | Yes, when the study child was 9 or 10];Yes, since the child's 11th birthday;Yes, both when the study child was 9/10 and 11+];No, did not happen in this period | No, did not happen in this period_Yes, when the study child was 9 or 10];Yes, since the child's 11th birthday_Yes, both when the study child was 9/10 and 11+ |
| r5022 | F23: Respondent's husband/partner was physically cruel to them since study child's 9th birthday | Quest_Mother | no | violence_between_parents | 11yrs2m | 11yrs | 11 | 11 | special_no | Yes, when study child was 9 or 10];Yes, since child's 11th birthday;Yes, both when the study child was 10/11 & 11+];No, did not happen in this period | No, did not happen in this period_Yes, since child's 11th birthday];Yes, when study child was 9 or 10_Yes, both when the study child was 10/11 & 11+ |
|  | | |  |  |  |  |  |  |  |  |  |

| **Table S5. ACE variables in adolescence (12-18 yrs).** | | | | | | | | | | | |
| --- | --- | --- | --- | --- | --- | --- | --- | --- | --- | --- | --- |
| **variable name** | **description** | **datasource** | **retrospective** | **ACE** | **time_alspac** | **time_period** | **time_yrs_start** | **time_yrs_end** | **reverse_scale** | **recode_ACE** | **factor_level_after_recode** |
| ff6011 | OR9: Frequency someone threatened/blackmailed teenager | Clinic_Child | no | bullying | 12.5yrs | 12.5yrs | 12.5 | 12.5 | no | Seldom (1-3 times);Frequently (>4 times)];Very frequently (>1/week) | FALSE_Seldom (1-3 times)_Frequently (>4 times)];Very frequently (>1/week) |
| ff6211 | RR9: Frequency friends have tried to get teenager to do things didn’t want to do | Clinic_Child | no | bullying | 12.5yrs | 12.5yrs | 12.5 | 12.5 | no | Seldom (1-3 times);Frequently (>4 times)];Very frequently (>1/week) | FALSE_Seldom (1-3 times)_Frequently (>4 times)];Very frequently (>1/week) |
| ff6221 | RR16: Frequency friends have told lies about teenager | Clinic_Child | no | bullying | 12.5yrs | 12.5yrs | 12.5 | 12.5 | special_no | Seldom (1-3 times);Frequently (>4 times)];Very frequently (>1/week)];Doesnt know | FALSE_Doesnt know_Seldom (1-3 times)_Frequently (>4 times)];Very frequently (>1/week) |
| fh8332 | EY2340: Frequency YPs friends put YP down in front of others : TF3 | Clinic_Child | no | bullying | 15.5yrs | 15.5yrs | 15.5 | 15.5 | no | Hardly ever/never;< Once a week];At least once a week;Most days | Hardly ever/never_< Once a week];At least once a week_Most days |
| fh8334 | EY2360: Frequency YPs friends put pressure on YP to do things they don't want to do : TF3 | Clinic_Child | no | bullying | 15.5yrs | 15.5yrs | 15.5 | 15.5 | no | Hardly ever/never;< Once a week];At least once a week;Most days | Hardly ever/never_< Once a week];At least once a week_Most days |
| ccxa210 | A9: Frequency during the last school year YP has been upset by name calling/exclusion from groups or bullying | Quest_Child Completed | no | bullying | year 11 | 16yrs | 16 | 16 | yes | Most days];Sometimes;Rarely;Never | Sometimes_Rarely_Never];Most days |
| fjpc4000 | PL642: Frequency YP has been directly bullied in the last 6 months [F17] | Clinic_Child | no | bullying | 17.5yrs | 17.5yrs | 17.5 | 17.5 | no | Never;Not much (1-3 times)];Quite a lot (>4 times a week);A lot (least once a week) | Never_Not much (1-3 times)];Quite a lot (>4 times a week)_A lot (least once a week) |
| fjpc4100 | PL645: Frequency YP has been relationally bullied in the last 6 months [F17] | Clinic_Child | no | bullying | 17.5yrs | 17.5yrs | 17.5 | 17.5 | no | Never;Not much (1-3 times)];Quite a lot (>4 times a week);A lot (least once a week) | Never_Not much (1-3 times)];Quite a lot (>4 times a week)_A lot (least once a week) |
| ypb8050 | H6a: Frequency adult in family shouted at respondent between ages of 11 and 17 | Quest_Child Completed | yes | emotional_abuse | 22yrs | 11-17yrs | 11 | 17 | yes | Never];Hardly ever;Sometimes;Most of the time;Always | Hardly ever_Sometimes_Most of the time_Always];Never |
| ypb8051 | H6b: Frequency adult in family said hurtful or insulting things to respondent between ages of 11 and 17 | Quest_Child Completed | yes | emotional_abuse | 22yrs | 11-17yrs | 11 | 17 | yes | Never];Hardly ever;Sometimes;Most of the time;Always | Hardly ever_Sometimes_Most of the time_Always];Never |
| ff5316 | PM70: Frequency carers ask teenager what happened in their free time | Clinic_Child | no | emotional_neglect | 12.5yrs | 12.5yrs | 12.5 | 12.5 | yes | Never];Hardly ever;Sometimes;Most of the time;Always | Hardly ever_Sometimes_Most of the time_Always];Never |
| ff5317 | PM80: Frequency past month carers started conversation about teenager’s spare time | Clinic_Child | no | emotional_neglect | 12.5yrs | 12.5yrs | 12.5 | 12.5 | yes | Never];Hardly ever;Sometimes;Most of the time;Always | Hardly ever_Sometimes_Most of the time_Always];Never |
| ff5318 | PM90: Frequency carers take time to listen, when teenager talks about what happened in their free time | Clinic_Child | no | emotional_neglect | 12.5yrs | 12.5yrs | 12.5 | 12.5 | yes | Never];Hardly ever;Sometimes;Most of the time;Always | Hardly ever_Sometimes_Most of the time_Always];Never |
| ff5331 | PM130: Frequency carers know who teenager’s friends are, outside of school | Clinic_Child | no | emotional_neglect | 12.5yrs | 12.5yrs | 12.5 | 13.5 | yes | Never];Hardly ever;Sometimes;Most of time;Always | Hardly ever_Sometimes_Most of time_Always];Never |
| ff5346 | PM240: Frequency carers ask teenager what has happened at school, on normal school day | Clinic_Child | no | emotional_neglect | 12.5yrs | 12.5yrs | 12.5 | 13.5 | yes | Never];Hardly ever;Sometimes;Most of time;Always | Hardly ever_Sometimes_Most of time_Always];Never |
| fg7116 | PM70: Frequency carers ask teenager what happened in free time : TF2 | Clinic_Child | no | emotional_neglect | 13.5yrs | 13.5yrs | 13.5 | 13.5 | yes | Never];Hardly ever;Sometimes;Most of time;Always | Hardly ever_Sometimes_Most of time_Always];Never |
| fg7118 | PM90: Frequency carers take time to listen, when teenager talks about what happened in free time : TF2 | Clinic_Child | no | emotional_neglect | 13.5yrs | 13.5yrs | 13.5 | 13.5 | yes | Never];Hardly ever;Sometimes;Most of time;Always | Hardly ever_Sometimes_Most of time_Always];Never |
| fg7131 | PM130: Frequency carers know who teenager’s friends are, outside of school : TF2 | Clinic_Child | no | emotional_neglect | 13.5yrs | 13.5yrs | 13.5 | 15.5 | yes | Never];Hardly ever;Sometimes;Most of time;Always;Doesn't apply | Hardly ever_Sometimes_Most of time_Always_Doesn't apply];Never |
| fg7146 | PM240: Frequency carers ask teenager what has happened at school, on normal school day : TF2 | Clinic_Child | no | emotional_neglect | 13.5yrs | 13.5yrs | 13.5 | 15.5 | yes | Never];Hardly ever;Sometimes;Most of time;Always;Dont know;Doesn't apply | Hardly ever_Sometimes_Most of time_Always_Dont know_Doesn't apply];Never |
| fh9819 | PM9: Frequency carer asks YP what they did in their free time : TF3 | Clinic_Child | no | emotional_neglect | 15.5yrs | 15.5yrs | 15.5 | 15.5 | yes | Never];Hardly ever;Sometimes;Most of time;Always;Dont know;Doesn't apply | Hardly ever_Sometimes_Most of time_Always_Dont know_Doesn't apply];Never |
| fh9820 | PM10: Frequency carer started conversation about YPs free time, in last month : TF3 | Clinic_Child | no | emotional_neglect | 15.5yrs | 15.5yrs | 15.5 | 15.5 | yes | Never];Hardly ever;Sometimes;Most of time;Always;Doesn't apply | Hardly ever_Sometimes_Most of time_Always_Doesn't apply];Never |
| fh9821 | PM11: Frequency carer listens to YP, when they talk about what they did in their free time : TF3 | Clinic_Child | no | emotional_neglect | 15.5yrs | 15.5yrs | 15.5 | 16 | no | Most days;Sometimes;Rarely];Never | Most days_Sometimes_Rarely];Never |
| fh9838 | PM28: Frequency carer asks YP about what happened at school on normal school day : TF3 | Clinic_Child | no | emotional_neglect | 15.5yrs | 15.5yrs | 15.5 | 16 | yes | Never];Sometimes;Often;Don't know | Sometimes_Often_Don't know];Never |
| ccxa240 | A12a: Frequency over last term parents have asked YP how they are getting on with different subjects at school | Quest_Child Completed | no | emotional_neglect | year 11 | 16yrs | 16 | 16 | yes | Never];Sometimes;Often;Don't know | Sometimes_Often_Don't know];Never |
| txa220 | A13a: Frequency in recent months parent/carer/other adult in household has talked to study child about study child's experiences at school | Quest_Child Based | no | emotional_neglect | school year 11/16yrs/192m (see txa991a) | 16yrs | 16 | 16 | yes | Never];Sometimes;Often;Don't know | Sometimes_Often_Don't know];Never |
| txa221 | A13b: Frequency in recent months parent/carer/other adult in household has talked to study child about study child's friends | Quest_Child Based | no | emotional_neglect | school year 11/16yrs/192m (see txa991a) | 16yrs | 16 | 17.5 | special_no | Very easy;Quite easy;Neutral;Quite difficult];Very difficlt];Don't know | Don't know_Very easy_Quite easy_Neutral_Quite difficult];Very difficlt |
| txa222 | A13c: Frequency in recent months parent/carer/other adult in household has talked to study child about things that are troubling study child | Quest_Child Based | no | emotional_neglect | school year 11/16yrs/192m (see txa991a) | 16yrs | 16 | 16 | no | No];Yes | No_FALSE];Yes |
| fjpc2100 | PL617: How easy YP finds it to discuss their problems with anyone in their family [F17] | Clinic_Child | no | emotional_neglect | 17.5yrs | 17.5yrs | 17.5 | 16 | no | No];Yes | No_FALSE];Yes |
| ypb8120 | H13: Frequency someone in family made respondent feel important or special, between ages 11 and 17 | Quest_Child Completed | yes | emotional_neglect | 22yrs | 11-17yrs | 11 | 12 | no | Partner always feels this way;Partner sometimes feels this way];Partner never feels this way | Partner always feels this way_Partner sometimes feels this way];Partner never feels this way |
| ccs6501 | L1b1: YP's mum has hurt themselves on purpose | Quest_Child Completed | no | mental_health_problems_or_suicide | 16yrs | 16yrs | 16 | 12 | no | Always feels this;Sometimes feels this];Never feels this | Always feels this_Sometimes feels this];Never feels this |
| ccs6502 | L1b2: YP's dad has hurt themselves on purpose | Quest_Child Completed | no | mental_health_problems_or_suicide | 16yrs | 16yrs | 16 | 12.5 | yes | Many conflicts];Some conflicts;Neutral;Quite agreeable;Very agreeable | Some conflicts_Neutral_Quite agreeable_Very agreeable];Many conflicts |
| pq3054 | C4e: Partner's partner seems to feel very close to study child | Quest_Partner | no | parent_child_bond | 12yrs1m | 12yrs | 12 | 17.5 | special_no | Very close to at least one;Quite close to at least one];Not very close to either;Not close at all to either];No parents;Don't know | No parents_Don't know_Very close to at least one_Quite close to at least one];Not very close to either_Not close at all to either |
| s3054 | C4e: Mother's partner seems very close to study child | Quest_Mother | no | parent_child_bond | 12yrs1m | 12yrs | 12 | 16 | yes | Yes];No | No];Yes |
| ff4530 | EAS20: Adult/child interaction - harmony | Clinic_Child | no | parent_child_bond | 12.5yrs | 12.5yrs | 12.5 | 17.5 | yes | Yes];No | No];Yes |
| fjpc2000 | PL615: How close YP feels to their parents [F17] | Clinic_Child | no | parent_child_bond | 17.5yrs | 17.5yrs | 17.5 | 17.5 | yes | Yes];No | No];Yes |
| ccs2050 | C6i: YP's parents have divorced/separated since the age of 12 | Quest_Child Completed | no | parental_separation | 16yrs | 12-16yrs | 12 | 17.5 | no | 1;2;3;4;5;6;7;8;9;10;11;12;13;14;15;16;17;18;19];20;21;22;23;24;25;26;27;29;31;34;36 | 1_2_3_4_5_6_7_8_9_10_11_12_13_14_15_16_17_18_19];20_21_22_23_24_25_26_27_29_31_34_36 |
| fjle112 | A7A: In the last year YP's parents have divorced [F17] | Clinic_Child | no | parental_separation | 17.5yrs | 16.5-17.5yrs | 16.5 | 13.5 | yes | Yes];No | No];Yes |
| fjle114 | A8A: In the last year YP's parents have separated [F17] | Clinic_Child | no | parental_separation | 17.5yrs | 16.5-17.5yrs | 16.5 | 13.5 | yes | Yes];No | No];Yes |
| ypb8053 | H6d: Frequency adult in family smacked respondent for discipline between ages of 11 and 17 | Quest_Child Completed | yes | physical_abuse | 22yrs | 11-17yrs | 11 | 13.5 | yes | Yes];No | No];Yes |
| ypb8056 | H6g: Frequency adult in family actually kicked, punched, hit respondent with something that could hurt respondent or physically attacked respondent in another way between ages of 11 and 17 | Quest_Child Completed | yes | physical_abuse | 22yrs | 11-17yrs | 11 | 13.5 | yes | Yes];No | No];Yes |
| ypb8057 | H6h: Frequency adult in family hit respondent so hard it left bruises or marks between ages of 11 and 17 | Quest_Child Completed | yes | physical_abuse | 22yrs | 11-17yrs | 11 | 13.5 | yes | Yes];No | No];Yes |
| ypb8052 | H6c: Frequency adult in family pushed, grabbed or shoved respondent between ages of 11 and 17 | Quest_Child Completed | yes | physical_abuse | 22yrs | 11-17yrs | 11 | 13.5 | yes | Yes];No | No];Yes |
| ypb8080 | H9: Respondent was touched in a sexual way by adult or older child, or was forced to touch adult or older child in a sexual way, between ages of 11 and 17 | Quest_Child Completed | yes | sexual_abuse | 22yrs | 11-17yrs | 11 | 17 | no | Never;Rarely;Sometimes;Often];Very Often | Never_Rarely_Sometimes_Often];Very Often |
| ypb8090 | H10: Adult or older child forced, or attempted to force, respondent into any sexual activity by threatening or holding respondent down or hurting respondent in some way, between ages of 11 and 17 | Quest_Child Completed | yes | sexual_abuse | 22yrs | 11-17yrs | 11 | 17 | no | Never;Rarely;Sometimes;Often];Very Often | Never_Rarely_Sometimes_Often];Very Often |
| fjal4000 | DV: Alcohol Use Disorders Identification Test (AUDIT) score [F17] | Clinic_Child | no | substance_household | 17.5yrs | 17.5yrs | 17.5 | 17 | yes | Never];Rarely;Sometimes;Often;Very Often | Rarely_Sometimes_Often_Very Often];Never |
| **Note.** YP = young person. | |  |  |  |  |  |  |  |  |  |  |

**Figure S1. Study design and definitions of the ACEs constructs.**

**Note.** (a) Study design and assessment time points; (b) definitions, respondents, and time periods of the ACEs constructs.

**Figure S2. Prevalence of the individual ACEs items and the cumulative ACEs scores across specific early-life periods.**

**Note.** Sample: ALSPAC (N = 3,931). Imputed data. ACEs = adverse childhood experience.

| **Table S6(a). Prevalence of ACEs (-1 to 18 years) and sample characteristics in the observed and imputed data.** | | |
| --- | --- | --- |
|  | **Observed data**  **(N=3931)** | **Imputed datasets ^a^**  **(N=20)** |
| ***Sociodemographic characteristics*** |  |  |
| **Sex** |  |  |
| Male | 1575 (40.1%) | 40.1% |
| Female | 2356 (59.9%) | 59.9% |
| **Ethnicity** |  |  |
| White | 3521 (96.3%) | 96.0% |
| Other | 137 (3.7%) | 4.0% |
| Missing | 273 (6.9%) | 0.0% |
| **Parental occupational class** |  |  |
| Professional | 397 (13.2%) | 12.6% |
| Managerial and technical | 1121 (37.3%) | 35.1% |
| Skilled non-manual | 409 (13.6%) | 13.4% |
| Skilled manual | 892 (29.7%) | 30.8% |
| Partly skilled | 131 (4.4%) | 5.3% |
| Unskilled | 54 (1.8%) | 2.7% |
| Missing | 927 (23.6%) | 0.0% |
| **Mother's education** |  |  |
| CSE | 354 (9.6%) | 10.0% |
| Vocational | 248 (6.7%) | 6.8% |
| O level | 1240 (33.5%) | 33.6% |
| A level | 1102 (29.8%) | 29.5% |
| Degree | 752 (20.3%) | 20.0% |
| Missing | 235 (6.0%) | 0.0% |
| **Mother's marital status at birth** |  |  |
| Never married | 462 (12.4%) | 12.7% |
| Separated/Divorced/Widowed | 157 (4.2%) | 4.4% |
| 1st marriage | 2863 (77.0%) | 76.4% |
| 2nd/3rd marriage | 237 (6.4%) | 6.4% |
| Missing | 212 (5.4%) | 0.0% |
| **Maternal smoking during pregnancy** |  |  |
| Yes | 646 (17.2%) | 17.9% |
| Missing | 168 (4.3%) | 0.0% |
| ***Adverse childhood experiences (ACEs)*** |  |  |
| **Physical abuse** |  |  |
| Yes | 380 (12.4%) | 12.3% |
| Missing | 869 (22.1%) | 0.0% |
| **Emotional abuse/neglect** |  |  |
| Yes | 1196 (32.7%) | 37.2% |
| Missing | 268 (6.8%) | 0.0% |
| **Sexual abuse** |  |  |
| Yes | 170 (4.8%) | 4.8% |
| Missing | 395 (10.0%) | 0.0% |
| **Bullying** |  |  |
| Yes | 919 (26.0%) | 25.9% |
| Missing | 392 (10.0%) | 0.0% |
| **Household violence** |  |  |
| Yes | 698 (20.6%) | 28.5% |
| Missing | 538 (13.7%) | 0.0% |
| **Parental substance use problems** |  |  |
| Yes | 365 (11.2%) | 12.0% |
| Missing | 674 (17.1%) | 0.0% |
| **Parental mental health problems** |  |  |
| Yes | 1504 (48.4%) | 50.7% |
| Missing | 826 (21.0%) | 0.0% |
| **Parental convictions** |  |  |
| Yes | 241 (7.4%) | 7.6% |
| Missing | 656 (16.7%) | 0.0% |
| **Parental separation** |  |  |
| Yes | 875 (27.9%) | 30.8% |
| Missing | 800 (20.4%) | 0.0% |
| **Low parent-child bonding** |  |  |
| Yes | 738 (23.7%) | 24.8% |
| Missing | 813 (20.7%) | 0.0% |
| **Cumulative ACEs score ^b^** |  |  |
| 0 ACEs | 921 (23.8%) | 13.0% |
| 1 ACEs | 1008 (26.0%) | 22.4% |
| 2 ACEs | 797 (20.6%) | 23.1% |
| 3 ACEs | 536 (13.8%) | 18.0% |
| 4 ACEs or more | 611 (15.8%) | 23.5% |
| Missing | 58 (1.5%) | 0.0% |
| ***Early-life inflammation*** |  |  |
| **CRP, age 9** |  |  |
| Mean (SD) | 0.560 (1.003) | 0.558 (1.004) |
| Range | 0.010 - 9.370 | 0.010 - 9.550 |
| Missing | 975 (24.8%) | 0.0% |
| **CRP, age 15** |  |  |
| Mean (SD) | 0.798 (1.207) | 0.814 (1.224) |
| Range | 0.070 - 10.000 | 0.070 - 10.000 |
| Missing | 1491 (37.9%) | 0.0% |
| **CRP, age 18** |  |  |
| Mean (SD) | 1.093 (1.425) | 1.085 (1.416) |
| Range | 0.020 - 9.470 | 0.020 - 9.470 |
| Missing | 1539 (39.2%) | 0.0% |
| ***Depressive symptoms in young adulthood*** |  |  |
| **Depressive symptoms, age 18** |  |  |
| Mean (SD) | 6.551 (5.708) | 6.667 (5.815) |
| Range | 0.000 - 26.000 | 0.000 - 26.000 |
| Missing | 1513 (38.5%) | 0.0% |
| **Depressive symptoms, age 21** |  |  |
| Mean (SD) | 5.533 (5.437) | 5.497 (4.767) |
| Range | 0.000 - 26.000 | 0.000 - 26.000 |
| Missing | 1457 (37.1%) | 0.0% |
| **Depressive symptoms, age 22** |  |  |
| Mean (SD) | 6.075 (5.433) | 5.994 (5.321) |
| Range | 0.000 - 26.000 | 0.000 - 26.000 |
| Missing | 1198 (30.5%) | 0.0% |
| **Depressive symptoms, age 23** |  |  |
| Mean (SD) | 6.762 (5.949) | 6.745 (5.404) |
| Range | 0.000 - 26.000 | 0.000 - 26.000 |
| Missing | 1106 (28.1%) | 0.0% |
| **Note.** Sample: ALSPAC (N = 3,931); ACEs = adverse childhood experiences; CRP = C-Reactive Protein; CSE = Certificate of Secondary Education; NA = Not Available; SD = standard deviation. ^a^ Only pooled percentages are presented for the imputed data as the specific counts may vary across the 20 imputed datasets; ^b^ The distribution of the ACEs score is presented for demonstration purposes only, as it was actually computed post-imputation given the high proportion of missing data in the individual ACEs items. | | |

| **Table S6(b). Prevalence of ACEs across the early-life periods in the observed and imputed data.** | | | | | | | | | | | | | | | | |
| --- | --- | --- | --- | --- | --- | --- | --- | --- | --- | --- | --- | --- | --- | --- | --- | --- |
|  | | **ACEs: -1 to 0 years** | | | **ACEs: 0 to 3 years** | | **ACEs: 3 to 7 years** | | | **ACEs: 7 to 12 years** | | | **ACEs: 12 to 18 years** | | **ACEs: -1 to 18 years** | |
|  | **Observed data** | | **Imputed data** | **Observed data** | | **Imputed data** | | **Observed data** | **Imputed data** | | **Observed data** | **Imputed data** | **Observed data** | **Imputed data** | **Observed data** | **Imputed data** |
| **Physical abuse** |  | |  |  | |  | |  |  | |  |  |  |  |  |  |
| Yes | 1 (0.0%) | | 30 (0.0%) | 113 (3.3%) | | 2534 (3.2%) | | 132 (4.1%) | 3229 (4.1%) | | 40 (1.2%) | 907 (1.2%) | 241 (7.5%) | 5297 (6.7%) | 380 (12.4%) | 9689 (12.3%) |
| Missing | 1072 (27.3%) | | 0 (0.0%) | 455 (11.6%) | | 0 (0.0%) | | 738 (18.8%) | 0 (0.0%) | | 608 (15.5%) | 0 (0.0%) | 709 (18.0%) | 0 (0.0%) | 869 (22.1%) | 0 (0.0%) |
| **Emotional abuse/neglect** | | |  |  | |  | |  |  | |  |  |  |  |  |  |
| Yes | 31 (0.8%) | | 683 (0.9%) | 252 (8.3%) | | 6796 (8.6%) | | 354 (11.1%) | 8821 (11.2%) | | 266 (7.2%) | 6198 (7.9%) | 727 (20.2%) | 17489 (22.2%) | 1196 (32.7%) | 29239 (37.2%) |
| Missing | 271 (6.9%) | | 0 (0.0%) | 889 (22.6%) | | 0 (0.0%) | | 739 (18.8%) | 0 (0.0%) | | 247 (6.3%) | 0 (0.0%) | 336 (8.5%) | 0 (0.0%) | 268 (6.8%) | 0 (0.0%) |
| **Sexual abuse** | x | | x |  | |  | |  |  | |  |  |  |  |  |  |
| Yes |  | |  | 2 (0.1%) | | 42 (0.1%) | | 18 (0.5%) | 436 (0.6%) | | 2 (0.1%) | 47 (0.1%) | 165 (6.1%) | 3513 (4.5%) | 170 (4.8%) | 3797 (4.8%) |
| Missing |  | |  | 259 (6.6%) | | 0 (0.0%) | | 416 (10.6%) | 0 (0.0%) | | 537 (13.7%) | 0 (0.0%) | 1204 (30.6%) | 0 (0.0%) | 395 (10.0%) | 0 (0.0%) |
| **Bullying** | x | | x | x | | x | | x | x | |  |  |  |  |  |  |
| Yes |  | |  |  | |  | |  |  | | 443 (12.7%) | 10023 (12.7%) | 587 (17.1%) | 12857 (16.4%) | 919 (26.0%) | 20377 (25.9%) |
| Missing |  | |  |  | |  | |  |  | | 444 (11.3%) | 0 (0.0%) | 497 (12.6%) | 0 (0.0%) | 392 (10.0%) | 0 (0.0%) |
| **Household violence** |  | |  |  | |  | |  |  | |  |  |  |  |  |  |
| Yes | 18 (0.6%) | | 607 (0.8%) | 166 (4.8%) | | 3888 (4.9%) | | 198 (6.2%) | 4996 (6.4%) | | 149 (4.5%) | 3581 (4.6%) | x | x | 698 (20.6%) | 22391 (28.5%) |
| Missing | 1072 (27.3%) | | 0 (0.0%) | 455 (11.6%) | | 0 (0.0%) | | 735 (18.7%) | 0 (0.0%) | | 600 (15.3%) | 0 (0.0%) |  |  | 538 (13.7%) | 0 (0.0%) |
| **Parental substance use problems** | | |  |  | |  | |  |  | |  |  |  |  |  |  |
| Yes | 109 (3.0%) | | 2524 (3.2%) | 135 (4.0%) | | 3539 (4.5%) | | 160 (5.3%) | 4501 (5.7%) | | 87 (3.0%) | 2631 (3.3%) | 38 (1.2%) | 899 (1.1%) | 365 (11.2%) | 9460 (12.0%) |
| Missing | 280 (7.1%) | | 0 (0.0%) | 560 (14.2%) | | 0 (0.0%) | | 889 (22.6%) | 0 (0.0%) | | 1052 (26.8%) | 0 (0.0%) | 813 (20.7%) | 0 (0.0%) | 674 (17.1%) | 0 (0.0%) |
| **Parental mental health problems** | | |  |  | |  | |  |  | |  |  |  |  |  |  |
| Yes | 863 (23.3%) | | 18612 (23.7%) | 910 (26.9%) | | 21884 (27.8%) | | 593 (18.9%) | 15877 (20.2%) | | 263 (12.2%) | 11197 (14.2%) | 39 (2.0%) | 1999 (2.5%) | 1504 (48.4%) | 39886 (50.7%) |
| Missing | 227 (5.8%) | | 0 (0.0%) | 553 (14.1%) | | 0 (0.0%) | | 799 (20.3%) | 0 (0.0%) | | 1781 (45.3%) | 0 (0.0%) | 1932 (49.1%) | 0 (0.0%) | 826 (21.0%) | 0 (0.0%) |
| **Parental convictions** |  | |  |  | |  | |  |  | |  |  |  |  |  |  |
| Yes | 47 (1.3%) | | 1114 (1.4%) | 101 (2.9%) | | 2374 (3.0%) | | 86 (2.7%) | 2115 (2.7%) | | 59 (1.9%) | 1518 (1.9%) | 24 (0.7%) | 538 (0.7%) | 241 (7.4%) | 5951 (7.6%) |
| Missing | 284 (7.2%) | | 0 (0.0%) | 471 (12.0%) | | 0 (0.0%) | | 729 (18.5%) | 0 (0.0%) | | 765 (19.5%) | 0 (0.0%) | 656 (16.7%) | 0 (0.0%) | 656 (16.7%) | 0 (0.0%) |
| **Parental separation** |  | |  |  | |  | |  |  | |  |  |  |  |  |  |
| Yes | 156 (4.3%) | | 3757 (4.8%) | 252 (7.3%) | | 6227 (7.9%) | | 351 (11.1%) | 9624 (12.2%) | | 206 (9.1%) | 9683 (12.3%) | 316 (10.5%) | 8324 (10.6%) | 875 (27.9%) | 24196 (30.8%) |
| Missing | 293 (7.5%) | | 0 (0.0%) | 464 (11.8%) | | 0 (0.0%) | | 772 (19.6%) | 0 (0.0%) | | 1657 (42.2%) | 0 (0.0%) | 911 (23.2%) | 0 (0.0%) | 800 (20.4%) | 0 (0.0%) |
| **Low parent-child bonding** | | |  |  | |  | |  |  | |  |  |  |  |  |  |
| Yes | x | | x | 124 (3.5%) | | 2948 (3.7%) | | 383 (12.7%) | 10521 (13.4%) | | 432 (14.4%) | 12006 (15.3%) | 367 (11.0%) | 8589 (10.9%) | 738 (23.7%) | 19529 (24.8%) |
| Missing |  | |  | 389 (9.9%) | | 0 (0.0%) | | 919 (23.4%) | 0 (0.0%) | | 922 (23.5%) | 0 (0.0%) | 581 (14.8%) | 0 (0.0%) | 813 (20.7%) | 0 (0.0%) |
| **Cumulative ACEs score** |  | |  |  | |  | |  |  | |  |  |  |  |  |  |
| 0 ACEs | 2684 (71.6%) | | 55264 (70.3%) | 2297 (62.2%) | | 45318 (57.6%) | | 2155 (60.8%) | 42404 (53.9%) | | 2394 (64.0%) | 41733 (53.1%) | 2072 (53.5%) | 35842 (45.6%) | 921 (23.8%) | 10259 (13.0%) |
| 1 ACEs | 925 (24.7%) | | 19992 (25.4%) | 958 (25.9%) | | 22166 (28.2%) | | 857 (24.2%) | 21666 (27.6%) | | 929 (24.8%) | 23118 (29.4%) | 1144 (29.5%) | 25659 (32.6%) | 1008 (26.0%) | 17616 (22.4%) |
| 2 ACEs | 120 (3.2%) | | 2853 (3.6%) | 276 (7.5%) | | 6945 (8.8%) | | 306 (8.6%) | 8477 (10.8%) | | 283 (7.6%) | 8809 (11.2%) | 446 (11.5%) | 11386 (14.5%) | 797 (20.6%) | 18134 (23.1%) |
| 3 ACEs | 17 (0.5%) | | 437 (0.6%) | 121 (3.3%) | | 2934 (3.7%) | | 143 (4.0%) | 3749 (4.8%) | | 94 (2.5%) | 3407 (4.3%) | 166 (4.3%) | 4232 (5.4%) | 536 (13.8%) | 14174 (18.0%) |
| 4 ACEs or more | 2 (0.1%) | | 74 (0.1%) | 43 (1.2%) | | 1257 (1.6%) | | 85 (2.4%) | 2324 (3.0%) | | 39 (1.0%) | 1553 (2.0%) | 45 (1.2%) | 1501 (1.9%) | 611 (15.8%) | 18437 (23.5%) |
| Missing | 183 (4.7%) | | 0 (0.0%) | 236 (6.0%) | | 0 (0.0%) | | 385 (9.8%) | 0 (0.0%) | | 192 (4.9%) | 0 (0.0%) | 58 (1.5%) | 0 (0.0%) | 58 (1.5%) | 0 (0.0%) |
| Note. Sample: ALSPAC (N=3,931). X = variable not available. ACEs = adverse childhood experiences. | | | | | | | | | | | | | | | | |

| **Table S7. Explorative factor analysis of ACEs (prenatal-18yrs) in the four training sets.** | | | | | | | | |
| --- | --- | --- | --- | --- | --- | --- | --- | --- |
|  | **Training set 1**  (N=2566) | | **Training set 2**  (N=2566) | | **Training set 3**  (N=2566) | | **Training set 4**  (N=2566) | |
| *ACE items* | *Factor 1* | *Factor 2* | *Factor 1* | *Factor 2* | *Factor 1* | *Factor 2* | *Factor 1* | *Factor 2* |
| Physical Abuse | **0.372*** | 0.114* | **0.361*** | 0.112* | **0.405*** | 0.065* | **0.353*** | 0.131* |
| Sexual Abuse | **0.129*** | 0.007 | **0.119*** | 0.020 | **0.133*** | -0.002 | **0.133*** | 0.000 |
| Emotional Abuse/ Neglect | **0.569*** | -0.009* | **0.562*** | -0.003 | **0.551*** | -0.003 | **0.579*** | -0.002* |
| Bullying Victimisation | **0.145*** | 0.005 | **0.166*** | -0.013 | **0.155*** | -0.004 | **0.166*** | -0.014 |
| Household Violence | 0.177* | **0.341*** | 0.123* | **0.374*** | 0.115 | **0.375*** | 0.048* | **0.429*** |
| Parental Substance Use Problems | -0.007* | **0.432*** | -0.037 | **0.435*** | -0.069 | **0.447*** | -0.046* | **0.418*** |
| Parental Mental Health Problems | 0.184* | **0.264*** | 0.163* | **0.282*** | 0.122 | **0.311*** | 0.072* | **0.352*** |
| Parental Convictions | -0.021 | **0.352*** | -0.015 | **0.330*** | -0.037 | **0.340*** | -0.054* | **0.347*** |
| Parental Separation | 0.144* | **0.359*** | 0.106* | **0.388*** | 0.054 | **0.425*** | 0.003 | **0.459*** |
| Low Parent-Child Bonding | **0.316*** | 0.014 | **0.345*** | 0.003 | **0.325*** | 0.002 | **0.272*** | 0.061* |
| **Note.** Sample = ALSPAC (N=12,830). Geomin rotated factor loadings. Bold coefficients indicate the highest factor loading of every item in each training set. * Significant at the 95% confidence level. | | | | | | | | |

**Figure S3. Confirmatory factor analysis (CFA) of the ACEs items (prenatal-18yrs) in the test dataset.**

**Note.** Sample: ALSPAC, test dataset (N = 2,566). Estimator = WLSMV. All coefficients are standardised and statistically significant (P-value < 0.001).

| **Table S8. Model fit indices of the latent class growth analysis.** | | | | | | |
| --- | --- | --- | --- | --- | --- | --- |
|  | **Depressive symptoms** | | | **CRP** | | |
|  | ***2-class model*** | ***3-class model*** | ***4-class model*** | ***2-class model*** | ***3-class model*** | ***4-class model*** |
| *AIC* | -30177.51 | **-29888.63** | -29838.82 | -11119.62 | **-11025.85** | -10918.75 |
| *BIC* | -30208.89 | **-29935.70** | -29901.59 | -11151.00 | **-11072.92** | -10981.51 |
| *N classes ≥ 5%* | Yes | **Yes** | Yes | Yes | **Yes** | No |
| *Entropy* | 0.818 | **0.731** | 0.616 | 0.575 | **0.584** | 0.559 |
| *Good Interpretability* | Yes | **Yes** | No, the trajectories of the first and second classes were almost identical. | Yes | **Yes** | Yes |
| **Note.** Depressive symptoms – the 3-class solution was selected as the best fitting model because its AIC and BIC values are similar to those of the 4-class model, and it has considerably higher entropy and better interpretability than the 4-class model. CRP – the three-class solutions was selected as the best fitting model because its AIC and BIC values are similar to those of the 4-class model, all classes include ≥ 5% of participants, and it has higher entropy. | | | | | | |

| **Table S9. Associations of ACEs in the prenatal period with moderate-high and high-moderate CRP trajectories (vs low-moderate).** | | | | | | | | | | | | | |
| --- | --- | --- | --- | --- | --- | --- | --- | --- | --- | --- | --- | --- | --- |
| **Outcome: CRP trajectories** |  | **Moderate-high Trajectory** | | | | | | **High-moderate Trajectory** | | | | | |
|  | **Model** | **OR** | **SE** | **Lower CI** | **Upper CI** | **p-value** | **p-value_fdr** | **OR** | **SE** | **Lower CI** | **Upper CI** | **p-value** | **p-value_fdr** |
| Total ACEs cumulative score | 1 | 0.83 | 0.08 | 0.67 | 0.99 | **0.020** | 0.090 | 0.94 | 0.10 | 0.74 | 1.14 | 0.536 | 0.794 |
| ***ACEs dimensions*** |  |  |  |  |  |  |  |  |  |  |  |  |  |
| Emotional/ Physical threat | 1 | 0.59 | 0.54 | -0.46 | 1.64 | 0.327 | 0.676 | 0.85 | 0.62 | -0.36 | 2.06 | 0.794 | 0.987 |
| Household Dysfunction | 1 | 0.79 | 0.10 | 0.60 | 0.99 | **0.018** | 0.090 | 0.90 | 0.13 | 0.65 | 1.15 | 0.406 | 0.701 |
| **Note.** Sample: ALSPAC (N = 3,931). Pooled estimates from multinomial logistic regression models across 20 imputed models. Reference outcome: Low-moderate CRP trajectory. CI = confidence interval. SE = standard error. fdr = false discovery rate (p-value). ACEs = adverse childhood experiences. CRP = C-reactive protein. Model 1: adjusted for sex, ethnicity, maternal smoking during pregnancy, mother’s marital status, mother’s education, and household’s social class. P-values highlighted in bold are statistically significant at the 95% confidence level. | | | | | | | | | | | | | |

| **Table S10. Associations of ACEs in the prenatal period with moderate and severe depression trajectories (vs low).** | | | | | | | | | | | | | |
| --- | --- | --- | --- | --- | --- | --- | --- | --- | --- | --- | --- | --- | --- |
| **Outcome: Depression trajectories** |  | **Moderate Trajectory** | | | | | | **Severe Trajectory** | | | | | |
|  | **Model** | **OR** | **SE** | **Lower CI** | **Upper CI** | **p-value** | **p-value_fdr** | **OR** | **SE** | **Lower CI** | **Upper CI** | **p-value** | **p-value_fdr** |
| Total ACEs cumulative score | 1 | 1.31 | 0.07 | 1.17 | 1.45 | **0.000** | **0.001** | 1.53 | 0.10 | 1.33 | 1.73 | **0.000** | **0.001** |
| ***ACEs dimensions*** |  |  |  |  |  |  |  |  |  |  |  |  |  |
| Emotional/ Physical threat | 1 | 0.43 | 0.61 | -0.77 | 1.62 | 0.164 | 0.436 | 2.53 | 0.45 | 1.64 | 3.42 | **0.041** | 0.137 |
| Household Dysfunction | 1 | 1.40 | 0.09 | 1.22 | 1.57 | **0.000** | **0.001** | 1.69 | 0.14 | 1.43 | 1.96 | **0.000** | **0.001** |
| **Note.** Sample: ALSPAC (N = 3,931). Pooled estimates from multinomial logistic regression models across 20 imputed models. Reference outcome: Low depression trajectory. CI = confidence interval. SE = standard error. fdr = false discovery rate (p-value). ACEs = adverse childhood experiences. Model 1: adjusted for sex, ethnicity, maternal smoking during pregnancy, mother’s marital status, mother’s education, and household’s social class. P-values highlighted in bold are statistically significant at the 95% confidence level. | | | | | | | | | | | | | |

| **Table S11. Associations of ACEs in early childhood (0-3 yrs) with moderate-high and high-moderate CRP trajectories (vs low-moderate).** | | | | | | | | | | | | | |
| --- | --- | --- | --- | --- | --- | --- | --- | --- | --- | --- | --- | --- | --- |
| **Outcome: CRP trajectories** | **Moderate-high Trajectory** | | | | | | | **High-moderate Trajectory** | | | | | |
|  | **Model** | **OR** | **SE** | **Lower CI** | **Upper CI** | **p-value** | **p-value_fdr** | **OR** | **SE** | **Lower CI** | **Upper CI** | **p-value** | **p-value_fdr** |
|  |  |  |  |  |  |  |  |  |  |  |  |  |  |
| Total ACEs cumulative score | 1 | 0.96 | 0.05 | 0.87 | 1.06 | 0.464 | 0.670 | 0.99 | 0.07 | 0.86 | 1.11 | 0.824 | 0.970 |
|  | 2 | 1.01 | 0.06 | 0.91 | 1.12 | 0.809 | 0.970 | 1.00 | 0.07 | 0.86 | 1.14 | 0.974 | 0.988 |
| ***ACEs dimensions and single adversities*** |  |  |  |  |  |  |  |  |  |  |  |  |  |
| Emotional/ Physical threat | 1 | 0.88 | 0.14 | 0.62 | 1.15 | 0.363 | 0.603 | 0.79 | 0.19 | 0.42 | 1.15 | 0.195 | 0.439 |
|  | 2 | 0.93 | 0.14 | 0.66 | 1.20 | 0.619 | 0.825 | 0.79 | 0.19 | 0.42 | 1.17 | 0.225 | 0.450 |
| - Physical abuse | 1 | 0.90 | 0.25 | 0.40 | 1.40 | 0.676 | 0.888 | 1.22 | 0.29 | 0.64 | 1.80 | 0.496 | 0.704 |
|  | 2 | 0.94 | 0.25 | 0.44 | 1.44 | 0.812 | 0.970 | 1.24 | 0.30 | 0.66 | 1.83 | 0.461 | 0.670 |
| - Emotional abuse/neglect | 1 | 0.81 | 0.16 | 0.50 | 1.13 | 0.201 | 0.442 | 0.75 | 0.22 | 0.31 | 1.19 | 0.195 | 0.439 |
|  | 2 | 0.87 | 0.16 | 0.54 | 1.19 | 0.384 | 0.619 | 0.76 | 0.23 | 0.31 | 1.21 | 0.225 | 0.450 |
| - Low Parent-Child Bonding | 1 | 1.01 | 0.23 | 0.55 | 1.47 | 0.980 | 0.988 | 1.00 | 0.31 | 0.39 | 1.60 | 0.988 | 0.988 |
|  | 2 | 1.05 | 0.24 | 0.59 | 1.51 | 0.827 | 0.970 | 1.01 | 0.31 | 0.41 | 1.61 | 0.971 | 0.988 |
| Household Dysfunction | 1 | 0.99 | 0.09 | 0.80 | 1.17 | 0.884 | 0.985 | 0.99 | 0.12 | 0.75 | 1.23 | 0.944 | 0.988 |
|  | 2 | 1.09 | 0.10 | 0.88 | 1.29 | 0.418 | 0.634 | 1.03 | 0.13 | 0.77 | 1.28 | 0.849 | 0.970 |
| - Household violence | 1 | 0.96 | 0.20 | 0.56 | 1.35 | 0.824 | 0.970 | 1.15 | 0.27 | 0.63 | 1.67 | 0.601 | 0.813 |
|  | 2 | 1.01 | 0.20 | 0.61 | 1.41 | 0.964 | 0.988 | 1.17 | 0.27 | 0.65 | 1.70 | 0.545 | 0.750 |
| - Parental Substance Use Problems | 1 | 0.68 | 0.26 | 0.18 | 1.18 | 0.133 | 0.354 | 1.52 | 0.24 | 1.05 | 1.99 | 0.080 | 0.244 |
|  | 2 | 0.74 | 0.26 | 0.23 | 1.25 | 0.241 | 0.462 | 1.59 | 0.24 | 1.11 | 2.06 | 0.057 | 0.192 |
| - Parental Mental Health Problems | 1 | 0.94 | 0.10 | 0.74 | 1.13 | 0.521 | 0.727 | 0.81 | 0.13 | 0.55 | 1.07 | 0.115 | 0.326 |
|  | 2 | 1.03 | 0.11 | 0.81 | 1.25 | 0.776 | 0.970 | 0.81 | 0.14 | 0.53 | 1.09 | 0.144 | 0.362 |
| - Parental Convictions | 1 | 1.27 | 0.24 | 0.81 | 1.74 | 0.312 | 0.528 | 1.03 | 0.32 | 0.40 | 1.67 | 0.917 | 0.988 |
|  | 2 | 1.33 | 0.24 | 0.86 | 1.80 | 0.232 | 0.454 | 1.05 | 0.32 | 0.42 | 1.69 | 0.875 | 0.985 |
| - Parental Separation | 1 | 1.15 | 0.17 | 0.82 | 1.48 | 0.412 | 0.634 | 1.25 | 0.22 | 0.82 | 1.68 | 0.308 | 0.528 |
|  | 2 | 1.24 | 0.17 | 0.90 | 1.57 | 0.220 | 0.450 | 1.29 | 0.22 | 0.85 | 1.73 | 0.253 | 0.466 |
| **Note.** Sample: ALSPAC (N = 3,931). Pooled estimates from multinomial logistic regression models across 20 imputed models. Reference outcome: Low-moderate CRP trajectory. CI = confidence interval. SE = standard error. fdr = false discovery rate (p-value). ACEs = adverse childhood experiences. CRP = C-reactive protein. Model 1: adjusted for sex, ethnicity, maternal smoking during pregnancy, mother’s marital status, mother’s education, and household’s social class; Model 2: Model 1 + earlier ACEs. P-values highlighted in bold are statistically significant at the 95% confidence level. | | | | | | | | | | | | | |

| **Table S12. Associations of ACEs in early childhood (0-3 yrs) with moderate and severe depression trajectories (vs low).** | | | | | | | | | | | | | |
| --- | --- | --- | --- | --- | --- | --- | --- | --- | --- | --- | --- | --- | --- |
| **Outcome: Depression trajectories** |  | **Moderate Trajectory** | | | | | | **Severe Trajectory** | | | | | |
|  | **Model** | **OR** | **SE** | **Lower CI** | **Upper CI** | **p-value** | **p-value_fdr** | **OR** | **SE** | **Lower CI** | **Upper CI** | **p-value** | **p-value_fdr** |
|  |  |  |  |  |  |  |  |  |  |  |  |  |  |
| Total ACEs cumulative score | 1 | 1.19 | 0.05 | 1.10 | 1.28 | **0.000** | **0.001** | 1.49 | 0.06 | 1.37 | 1.61 | **0.000** | **0.000** |
|  | 2 | 1.13 | 0.05 | 1.03 | 1.23 | **0.015** | 0.073 | 1.42 | 0.07 | 1.29 | 1.56 | **0.000** | **0.000** |
| ***ACEs dimensions and single adversities*** |  |  |  |  |  |  |  |  |  |  |  |  |  |
| Emotional/ Physical threat | 1 | 1.34 | 0.12 | 1.10 | 1.58 | **0.018** | 0.084 | 1.91 | 0.18 | 1.57 | 2.25 | **0.000** | **0.002** |
|  | 2 | 1.24 | 0.13 | 0.99 | 1.49 | 0.087 | 0.256 | 1.69 | 0.18 | 1.34 | 2.05 | **0.004** | **0.024** |
| - Physical abuse | 1 | 1.29 | 0.22 | 0.85 | 1.73 | 0.254 | 0.466 | 2.37 | 0.29 | 1.81 | 2.93 | **0.003** | **0.018** |
|  | 2 | 1.21 | 0.22 | 0.77 | 1.65 | 0.394 | 0.619 | 2.14 | 0.29 | 1.57 | 2.71 | **0.009** | **0.045** |
| - Emotional abuse/neglect | 1 | 1.35 | 0.15 | 1.06 | 1.64 | **0.040** | 0.146 | 1.89 | 0.21 | 1.48 | 2.30 | **0.002** | **0.017** |
|  | 2 | 1.24 | 0.15 | 0.94 | 1.53 | 0.155 | 0.378 | 1.63 | 0.22 | 1.20 | 2.06 | **0.026** | 0.109 |
| - Low Parent-Child Bonding | 1 | 1.34 | 0.21 | 0.93 | 1.76 | 0.162 | 0.386 | 1.85 | 0.28 | 1.30 | 2.40 | **0.028** | 0.111 |
|  | 2 | 1.26 | 0.21 | 0.84 | 1.68 | 0.280 | 0.492 | 1.67 | 0.28 | 1.11 | 2.22 | 0.070 | 0.227 |
| Household Dysfunction | 1 | 1.42 | 0.08 | 1.25 | 1.58 | **0.000** | **0.000** | 2.15 | 0.14 | 1.88 | 2.42 | **0.000** | **0.000** |
|  | 2 | 1.30 | 0.09 | 1.12 | 1.48 | **0.004** | **0.024** | 1.93 | 0.15 | 1.63 | 2.23 | **0.000** | **0.000** |
| - Household violence | 1 | 1.02 | 0.20 | 0.63 | 1.41 | 0.916 | 0.988 | 1.79 | 0.25 | 1.30 | 2.28 | **0.021** | 0.092 |
|  | 2 | 0.94 | 0.20 | 0.55 | 1.34 | 0.770 | 0.970 | 1.58 | 0.26 | 1.08 | 2.09 | 0.074 | 0.231 |
| - Parental Substance Use Problems | 1 | 1.34 | 0.20 | 0.95 | 1.73 | 0.143 | 0.362 | 1.70 | 0.28 | 1.15 | 2.24 | 0.056 | 0.192 |
|  | 2 | 1.19 | 0.20 | 0.79 | 1.59 | 0.394 | 0.619 | 1.38 | 0.29 | 0.81 | 1.95 | 0.265 | 0.475 |
| - Parental Mental Health Problems | 1 | 1.52 | 0.09 | 1.34 | 1.69 | **0.000** | **0.000** | 2.05 | 0.14 | 1.78 | 2.33 | **0.000** | **0.000** |
|  | 2 | 1.40 | 0.10 | 1.20 | 1.59 | **0.001** | **0.005** | 1.81 | 0.16 | 1.51 | 2.12 | **0.000** | **0.001** |
| - Parental Convictions | 1 | 0.72 | 0.26 | 0.21 | 1.24 | 0.213 | 0.450 | 1.13 | 0.33 | 0.48 | 1.79 | 0.708 | 0.916 |
|  | 2 | 0.67 | 0.26 | 0.15 | 1.19 | 0.132 | 0.354 | 1.01 | 0.34 | 0.34 | 1.67 | 0.982 | 0.988 |
| - Parental Separation | 1 | 1.13 | 0.16 | 0.82 | 1.45 | 0.435 | 0.649 | 1.87 | 0.22 | 1.44 | 2.31 | **0.005** | **0.025** |
|  | 2 | 1.03 | 0.16 | 0.71 | 1.35 | 0.848 | 0.970 | 1.61 | 0.23 | 1.16 | 2.06 | **0.039** | 0.146 |
| **Note.** Sample: ALSPAC (N = 3,931). Pooled estimates from multinomial logistic regression models across 20 imputed models. Reference outcome: Low depression trajectory. CI = confidence interval. SE = standard error. fdr = false discovery rate (p-value). ACEs = adverse childhood experiences. Model 1: adjusted for sex, ethnicity, maternal smoking during pregnancy, mother’s marital status, mother’s education, and household’s social class; Model 2: Model 1 + earlier ACEs. P-values highlighted in bold are statistically significant at the 95% confidence level. | | | | | | | | | | | | | |

| **Table S13. Associations of ACEs in middle childhood (3-7 yrs) with moderate-high and high-moderate CRP trajectories (vs low-moderate).** | | | | | | | | | | | | | |
| --- | --- | --- | --- | --- | --- | --- | --- | --- | --- | --- | --- | --- | --- |
| **Outcome: CRP trajectories** |  | **Moderate-high Trajectory** | | | | | | **High-moderate Trajectory** | | | | | |
|  | **Model** | **OR** | **SE** | **Lower CI** | **Upper CI** | **p-value** | **p-value_fdr** | **OR** | **SE** | **Lower CI** | **Upper CI** | **p-value** | **p-value_fdr** |
|  |  |  |  |  |  |  |  |  |  |  |  |  |  |
| Total ACEs cumulative score | 1 | 0.99 | 0.04 | 0.90 | 1.07 | 0.728 | 0.987 | 1.03 | 0.05 | 0.93 | 1.14 | 0.550 | 0.987 |
|  | 2 | 1.00 | 0.05 | 0.91 | 1.10 | 0.926 | 0.987 | 1.05 | 0.06 | 0.93 | 1.18 | 0.430 | 0.984 |
| ***ACEs dimensions and single adversities*** |  |  |  |  |  |  |  |  |  |  |  |  |  |
| Emotional/ Physical threat | 1 | 1.00 | 0.11 | 0.79 | 1.21 | 0.993 | 0.999 | 0.97 | 0.15 | 0.68 | 1.26 | 0.829 | 0.987 |
|  | 2 | 1.03 | 0.11 | 0.81 | 1.24 | 0.814 | 0.987 | 0.98 | 0.16 | 0.66 | 1.29 | 0.877 | 0.987 |
| - Physical abuse | 1 | 0.79 | 0.25 | 0.30 | 1.28 | 0.336 | 0.946 | 1.03 | 0.28 | 0.48 | 1.59 | 0.905 | 0.987 |
|  | 2 | 0.80 | 0.26 | 0.30 | 1.30 | 0.379 | 0.946 | 1.05 | 0.29 | 0.48 | 1.61 | 0.877 | 0.987 |
| - Emotional abuse/neglect | 1 | 0.98 | 0.14 | 0.70 | 1.26 | 0.915 | 0.987 | 0.93 | 0.19 | 0.56 | 1.31 | 0.715 | 0.987 |
|  | 2 | 1.02 | 0.15 | 0.72 | 1.31 | 0.917 | 0.987 | 0.94 | 0.20 | 0.55 | 1.33 | 0.759 | 0.987 |
| - Low Parent-Child Bonding | 1 | 1.03 | 0.13 | 0.77 | 1.29 | 0.821 | 0.987 | 1.07 | 0.17 | 0.72 | 1.41 | 0.711 | 0.987 |
|  | 2 | 1.05 | 0.13 | 0.79 | 1.31 | 0.705 | 0.987 | 1.08 | 0.18 | 0.72 | 1.43 | 0.678 | 0.987 |
| Household Dysfunction | 1 | 1.00 | 0.10 | 0.81 | 1.19 | 0.999 | 0.999 | 1.06 | 0.13 | 0.81 | 1.31 | 0.668 | 0.987 |
|  | 2 | 1.04 | 0.11 | 0.83 | 1.25 | 0.694 | 0.987 | 1.08 | 0.14 | 0.81 | 1.36 | 0.566 | 0.987 |
| - Household violence | 1 | 1.13 | 0.17 | 0.79 | 1.47 | 0.479 | 0.987 | 1.06 | 0.25 | 0.57 | 1.55 | 0.821 | 0.987 |
|  | 2 | 1.18 | 0.18 | 0.83 | 1.52 | 0.357 | 0.946 | 1.08 | 0.26 | 0.57 | 1.59 | 0.777 | 0.987 |
| - Parental Substance Use Problems | 1 | 0.84 | 0.20 | 0.45 | 1.24 | 0.405 | 0.971 | 1.37 | 0.23 | 0.92 | 1.82 | 0.170 | 0.573 |
|  | 2 | 0.88 | 0.21 | 0.48 | 1.29 | 0.546 | 0.987 | 1.41 | 0.24 | 0.95 | 1.88 | 0.144 | 0.512 |
| - Parental Mental Health Problems | 1 | 0.91 | 0.12 | 0.67 | 1.14 | 0.425 | 0.984 | 1.06 | 0.14 | 0.79 | 1.34 | 0.663 | 0.987 |
|  | 2 | 0.94 | 0.13 | 0.69 | 1.20 | 0.640 | 0.987 | 1.10 | 0.16 | 0.79 | 1.40 | 0.559 | 0.987 |
| - Parental Convictions | 1 | 0.98 | 0.28 | 0.44 | 1.53 | 0.953 | 0.990 | 0.98 | 0.35 | 0.28 | 1.67 | 0.943 | 0.990 |
|  | 2 | 1.00 | 0.28 | 0.46 | 1.55 | 0.991 | 0.999 | 0.98 | 0.35 | 0.29 | 1.67 | 0.959 | 0.990 |
| - Parental Separation | 1 | 1.07 | 0.14 | 0.80 | 1.35 | 0.609 | 0.987 | 1.03 | 0.19 | 0.66 | 1.40 | 0.864 | 0.987 |
|  | 2 | 1.10 | 0.14 | 0.81 | 1.38 | 0.528 | 0.987 | 1.04 | 0.19 | 0.66 | 1.42 | 0.836 | 0.987 |
| **Note.** Sample: ALSPAC (N = 3,931). Pooled estimates from multinomial logistic regression models across 20 imputed models. Reference outcome: Low-moderate CRP trajectory. CI = confidence interval. SE = standard error. fdr = false discovery rate (p-value). ACEs = adverse childhood experiences. CRP = C-reactive protein. Model 1: adjusted for sex, ethnicity, maternal smoking during pregnancy, mother’s marital status, mother’s education, and household’s social class; Model 2: Model 1 + earlier ACEs. P-values highlighted in bold are statistically significant at the 95% confidence level. | | | | | | | | | | | | | |

| **Table S14. Associations of ACEs in middle childhood (3-7 yrs) with moderate and severe depression trajectories (vs low).** | | | | | | | | | | | | | |
| --- | --- | --- | --- | --- | --- | --- | --- | --- | --- | --- | --- | --- | --- |
| **Outcome: Depression trajectories** | | **Moderate Trajectory** | | | | | | **Severe Trajectory** | | | | | |
|  | **Model** | **OR** | **SE** | **Lower CI** | **Upper CI** | **p-value** | **p-value_fdr** | **OR** | **SE** | **Lower CI** | **Upper CI** | **p-value** | **p-value_fdr** |
|  |  |  |  |  |  |  |  |  |  |  |  |  |  |
| Total ACEs cumulative score | 1 | 1.15 | 0.04 | 1.08 | 1.23 | **0.000** | **0.005** | 1.35 | 0.05 | 1.24 | 1.45 | **0.000** | **0.000** |
|  | 2 | 1.10 | 0.04 | 1.01 | 1.18 | **0.040** | 0.225 | 1.18 | 0.06 | 1.05 | 1.31 | **0.010** | 0.075 |
| ***ACEs dimensions and single adversities*** | |  |  |  |  |  |  |  |  |  |  |  |  |
| Emotional/ Physical threat | 1 | 1.28 | 0.10 | 1.08 | 1.48 | **0.015** | 0.096 | 1.87 | 0.15 | 1.58 | 2.17 | **0.000** | **0.001** |
|  | 2 | 1.17 | 0.11 | 0.97 | 1.38 | 0.130 | 0.498 | 1.50 | 0.16 | 1.18 | 1.81 | **0.012** | 0.081 |
| - Physical abuse | 1 | 1.35 | 0.20 | 0.95 | 1.74 | 0.140 | 0.512 | 1.32 | 0.32 | 0.70 | 1.94 | 0.378 | 0.946 |
|  | 2 | 1.17 | 0.21 | 0.77 | 1.58 | 0.442 | 0.987 | 0.90 | 0.33 | 0.25 | 1.56 | 0.762 | 0.987 |
| - Emotional abuse/neglect | 1 | 1.48 | 0.13 | 1.23 | 1.73 | **0.002** | **0.019** | 1.81 | 0.18 | 1.46 | 2.17 | **0.001** | **0.013** |
|  | 2 | 1.33 | 0.13 | 1.07 | 1.59 | **0.031** | 0.184 | 1.36 | 0.19 | 0.99 | 1.73 | 0.104 | 0.417 |
| - Low Parent-Child Bonding | 1 | 1.15 | 0.12 | 0.91 | 1.39 | 0.260 | 0.805 | 1.78 | 0.18 | 1.42 | 2.14 | **0.002** | **0.014** |
|  | 2 | 1.06 | 0.13 | 0.82 | 1.31 | 0.630 | 0.987 | 1.45 | 0.19 | 1.08 | 1.82 | **0.048** | 0.254 |
| Household Dysfunction | 1 | 1.32 | 0.09 | 1.15 | 1.49 | **0.001** | **0.014** | 1.74 | 0.14 | 1.47 | 2.02 | **0.000** | **0.002** |
|  | 2 | 1.18 | 0.09 | 1.00 | 1.36 | 0.077 | 0.350 | 1.30 | 0.16 | 1.00 | 1.61 | 0.091 | 0.393 |
| - Household violence | 1 | 1.09 | 0.17 | 0.76 | 1.42 | 0.617 | 0.987 | 1.53 | 0.24 | 1.07 | 2.00 | 0.072 | 0.346 |
|  | 2 | 0.94 | 0.17 | 0.60 | 1.28 | 0.717 | 0.987 | 1.07 | 0.25 | 0.58 | 1.56 | 0.783 | 0.987 |
| - Parental Substance Use Problems | 1 | 1.34 | 0.17 | 1.00 | 1.67 | 0.094 | 0.393 | 1.26 | 0.27 | 0.74 | 1.79 | 0.384 | 0.946 |
|  | 2 | 1.17 | 0.17 | 0.83 | 1.51 | 0.366 | 0.946 | 0.92 | 0.28 | 0.37 | 1.48 | 0.782 | 0.987 |
| - Parental Mental Health Problems | 1 | 1.58 | 0.10 | 1.38 | 1.77 | **0.000** | **0.000** | 1.88 | 0.15 | 1.58 | 2.18 | **0.000** | **0.001** |
|  | 2 | 1.42 | 0.11 | 1.21 | 1.63 | **0.001** | **0.013** | 1.38 | 0.17 | 1.05 | 1.71 | 0.057 | 0.289 |
| - Parental Convictions | 1 | 0.90 | 0.27 | 0.37 | 1.42 | 0.680 | 0.987 | 1.22 | 0.38 | 0.48 | 1.97 | 0.598 | 0.987 |
|  | 2 | 0.84 | 0.27 | 0.31 | 1.36 | 0.510 | 0.987 | 1.04 | 0.38 | 0.30 | 1.79 | 0.909 | 0.987 |
| - Parental Separation | 1 | 0.95 | 0.14 | 0.68 | 1.22 | 0.715 | 0.987 | 1.60 | 0.18 | 1.25 | 1.96 | **0.009** | 0.075 |
|  | 2 | 0.87 | 0.14 | 0.60 | 1.14 | 0.305 | 0.886 | 1.29 | 0.19 | 0.92 | 1.66 | 0.177 | 0.573 |
| **Note.** Sample: ALSPAC (N = 3,931). Pooled estimates from multinomial logistic regression models across 20 imputed models. Reference outcome: Low depression trajectory. CI = confidence interval. SE = standard error. fdr = false discovery rate (p-value). ACEs = adverse childhood experiences. Model 1: adjusted for sex, ethnicity, maternal smoking during pregnancy, mother’s marital status, mother’s education, and household’s social class; Model 2: Model 1 + earlier ACEs. P-values highlighted in bold are statistically significant at the 95% confidence level. | | | | | | | | | | | | | |

| **Table S15. Associations of ACEs in late childhood (7-12 yrs) with moderate-high and high-moderate CRP trajectories (vs low-moderate).** | | | | | | | | | | | | | |
| --- | --- | --- | --- | --- | --- | --- | --- | --- | --- | --- | --- | --- | --- |
| **Outcome: CRP trajectories** |  | **Moderate-high Trajectory** | | | | | | **High-moderate Trajectory** | | | | | |
|  | **Model** | **OR** | **SE** | **Lower CI** | **Upper CI** | **p-value** | **p-value_fdr** | **OR** | **SE** | **Lower CI** | **Upper CI** | **p-value** | **p-value_fdr** |
|  |  |  |  |  |  |  |  |  |  |  |  |  |  |
| Total ACEs cumulative score | 1 | 1.01 | 0.04 | 0.93 | 1.10 | 0.757 | 0.995 | 1.04 | 0.06 | 0.93 | 1.15 | 0.510 | 0.995 |
|  | 2 | 1.03 | 0.05 | 0.93 | 1.13 | 0.529 | 0.995 | 1.03 | 0.07 | 0.90 | 1.16 | 0.648 | 0.995 |
| ***ACEs dimensions and single adversities*** |  |  |  |  |  |  |  |  |  |  |  |  |  |
| Emotional/ Physical threat | 1 | 0.99 | 0.11 | 0.78 | 1.20 | 0.911 | 0.995 | 0.98 | 0.14 | 0.70 | 1.26 | 0.890 | 0.995 |
|  | 2 | 1.00 | 0.12 | 0.78 | 1.23 | 0.973 | 0.995 | 0.95 | 0.15 | 0.64 | 1.25 | 0.717 | 0.995 |
| - Physical abuse | 1 | 0.60 | 0.51 | -0.40 | 1.60 | 0.317 | 0.801 | 1.02 | 0.49 | 0.05 | 1.98 | 0.976 | 0.995 |
|  | 2 | 0.62 | 0.52 | -0.39 | 1.63 | 0.353 | 0.848 | 0.98 | 0.50 | 0.00 | 1.96 | 0.968 | 0.995 |
| - Emotional abuse/neglect | 1 | 0.98 | 0.16 | 0.67 | 1.30 | 0.916 | 0.995 | 0.93 | 0.22 | 0.50 | 1.35 | 0.724 | 0.995 |
|  | 2 | 1.01 | 0.17 | 0.68 | 1.33 | 0.976 | 0.995 | 0.90 | 0.22 | 0.47 | 1.33 | 0.630 | 0.995 |
| - Low Parent-Child Bonding | 1 | 1.03 | 0.12 | 0.79 | 1.26 | 0.835 | 0.995 | 1.04 | 0.16 | 0.72 | 1.35 | 0.822 | 0.995 |
|  | 2 | 1.04 | 0.13 | 0.79 | 1.29 | 0.746 | 0.995 | 1.00 | 0.17 | 0.67 | 1.34 | 0.980 | 0.995 |
| Household Dysfunction | 1 | 1.04 | 0.10 | 0.85 | 1.24 | 0.666 | 0.995 | 0.97 | 0.13 | 0.71 | 1.24 | 0.831 | 0.995 |
|  | 2 | 1.07 | 0.11 | 0.87 | 1.28 | 0.500 | 0.995 | 0.95 | 0.15 | 0.66 | 1.24 | 0.718 | 0.995 |
| - Household violence | 1 | 1.00 | 0.21 | 0.58 | 1.42 | 0.993 | 0.995 | 1.06 | 0.27 | 0.53 | 1.59 | 0.833 | 0.995 |
|  | 2 | 1.02 | 0.22 | 0.59 | 1.46 | 0.913 | 0.995 | 1.02 | 0.28 | 0.46 | 1.58 | 0.938 | 0.995 |
| - Parental Substance Use Problems | 1 | 0.57 | 0.31 | -0.05 | 1.18 | 0.070 | 0.281 | 1.10 | 0.30 | 0.52 | 1.69 | 0.745 | 0.995 |
|  | 2 | 0.60 | 0.32 | -0.03 | 1.22 | 0.102 | 0.362 | 1.10 | 0.30 | 0.51 | 1.70 | 0.747 | 0.995 |
| - Parental Mental Health Problems | 1 | 1.12 | 0.14 | 0.84 | 1.40 | 0.436 | 0.951 | 1.10 | 0.19 | 0.74 | 1.47 | 0.596 | 0.995 |
|  | 2 | 1.16 | 0.15 | 0.86 | 1.45 | 0.337 | 0.830 | 1.10 | 0.19 | 0.72 | 1.48 | 0.624 | 0.995 |
| - Parental Convictions | 1 | 1.00 | 0.31 | 0.39 | 1.62 | 0.995 | 0.995 | 0.21 | 1.00 | -1.75 | 2.17 | 0.122 | 0.391 |
|  | 2 | 1.01 | 0.31 | 0.40 | 1.63 | 0.963 | 0.995 | 0.21 | 1.00 | -1.75 | 2.17 | 0.121 | 0.391 |
| - Parental Separation | 1 | 1.17 | 0.15 | 0.88 | 1.46 | 0.294 | 0.762 | 0.93 | 0.19 | 0.55 | 1.31 | 0.717 | 0.995 |
|  | 2 | 1.18 | 0.16 | 0.88 | 1.49 | 0.285 | 0.760 | 0.89 | 0.20 | 0.50 | 1.29 | 0.580 | 0.995 |
| Bullying | 1 | 1.00 | 0.14 | 0.74 | 1.27 | 0.981 | 0.995 | 1.43 | 0.15 | 1.13 | 1.73 | **0.019** | 0.111 |
|  | 2 | 1.02 | 0.14 | 0.75 | 1.28 | 0.908 | 0.995 | 1.43 | 0.15 | 1.13 | 1.73 | **0.020** | 0.111 |
| **Note.** Sample: ALSPAC (N = 3,931). Pooled estimates from multinomial logistic regression models across 20 imputed models. Reference outcome: Low-moderate CRP trajectory. CI = confidence interval. SE = standard error. fdr = false discovery rate (p-value). ACEs = adverse childhood experiences. CRP = C-reactive protein. Model 1: adjusted for sex, ethnicity, maternal smoking during pregnancy, mother’s marital status, mother’s education, and household’s social class; Model 2: Model 1 + earlier ACEs. P-values highlighted in bold are statistically significant at the 95% confidence level. | | | | | | | | | | | | | |

| **Table S16. Associations of ACEs in late childhood (7-12 yrs) with moderate and severe depression trajectories (vs low).** | | | | | | | | | | | | | |
| --- | --- | --- | --- | --- | --- | --- | --- | --- | --- | --- | --- | --- | --- |
| **Outcome: Depression trajectories** | | **Moderate Trajectory** | | | | | | **Severe Trajectory** | | | | | |
|  | **Model** | **OR** | **SE** | **Lower CI** | **Upper CI** | **p-value** | **p-value_fdr** | **OR** | **SE** | **Lower CI** | **Upper CI** | **p-value** | **p-value_fdr** |
|  |  |  |  |  |  |  |  |  |  |  |  |  |  |
| Total ACEs cumulative score | 1 | 1.19 | 0.04 | 1.11 | 1.27 | **0.000** | **0.001** | 1.35 | 0.06 | 1.23 | 1.47 | **0.000** | **0.000** |
|  | 2 | 1.13 | 0.05 | 1.04 | 1.22 | **0.006** | 0.056 | 1.20 | 0.07 | 1.06 | 1.34 | **0.009** | 0.072 |
| ***ACEs dimensions and single adversities*** |  |  |  |  |  |  |  |  |  |  |  |  |  |
| Emotional/ Physical threat | 1 | 1.17 | 0.10 | 0.98 | 1.37 | 0.107 | 0.367 | 1.82 | 0.15 | 1.53 | 2.11 | **0.000** | **0.002** |
|  | 2 | 1.04 | 0.11 | 0.83 | 1.25 | 0.722 | 0.995 | 1.45 | 0.16 | 1.14 | 1.76 | **0.019** | 0.111 |
| - Physical abuse | 1 | 1.10 | 0.38 | 0.36 | 1.84 | 0.806 | 0.995 | 1.34 | 0.57 | 0.23 | 2.45 | 0.602 | 0.995 |
|  | 2 | 0.85 | 0.39 | 0.09 | 1.60 | 0.671 | 0.995 | 0.81 | 0.58 | -0.33 | 1.95 | 0.718 | 0.995 |
| - Emotional abuse/neglect | 1 | 1.54 | 0.15 | 1.26 | 1.83 | **0.003** | **0.032** | 1.56 | 0.22 | 1.14 | 1.98 | **0.040** | 0.181 |
|  | 2 | 1.37 | 0.15 | 1.07 | 1.66 | **0.039** | 0.181 | 1.14 | 0.23 | 0.70 | 1.59 | 0.559 | 0.995 |
| - Low Parent-Child Bonding | 1 | 1.06 | 0.12 | 0.83 | 1.29 | 0.617 | 0.995 | 1.80 | 0.17 | 1.47 | 2.13 | **0.000** | **0.009** |
|  | 2 | 0.94 | 0.13 | 0.69 | 1.19 | 0.606 | 0.995 | 1.44 | 0.18 | 1.09 | 1.79 | **0.042** | 0.181 |
| Household Dysfunction | 1 | 1.28 | 0.10 | 1.09 | 1.47 | **0.011** | 0.080 | 1.47 | 0.16 | 1.15 | 1.79 | **0.018** | 0.111 |
|  | 2 | 1.15 | 0.10 | 0.95 | 1.36 | 0.163 | 0.489 | 1.16 | 0.18 | 0.80 | 1.52 | 0.419 | 0.938 |
| - Household violence | 1 | 1.32 | 0.20 | 0.94 | 1.70 | 0.155 | 0.481 | 1.61 | 0.27 | 1.08 | 2.14 | 0.078 | 0.298 |
|  | 2 | 1.10 | 0.20 | 0.71 | 1.50 | 0.624 | 0.995 | 1.11 | 0.28 | 0.55 | 1.67 | 0.715 | 0.995 |
| - Parental Substance Use Problems | 1 | 1.58 | 0.23 | 1.13 | 2.02 | **0.045** | 0.187 | 1.10 | 0.39 | 0.33 | 1.88 | 0.805 | 0.995 |
|  | 2 | 1.33 | 0.23 | 0.87 | 1.78 | 0.225 | 0.653 | 0.74 | 0.41 | -0.06 | 1.55 | 0.470 | 0.981 |
| - Parental Mental Health Problems | 1 | 1.47 | 0.13 | 1.21 | 1.73 | **0.004** | **0.038** | 1.57 | 0.20 | 1.17 | 1.97 | **0.028** | 0.147 |
|  | 2 | 1.33 | 0.14 | 1.07 | 1.60 | **0.035** | 0.176 | 1.26 | 0.21 | 0.85 | 1.67 | 0.272 | 0.745 |
| - Parental Convictions | 1 | 1.36 | 0.28 | 0.81 | 1.90 | 0.270 | 0.745 | 1.11 | 0.47 | 0.19 | 2.04 | 0.822 | 0.995 |
|  | 2 | 1.29 | 0.28 | 0.74 | 1.84 | 0.364 | 0.853 | 0.99 | 0.48 | 0.05 | 1.93 | 0.982 | 0.995 |
| - Parental Separation | 1 | 0.98 | 0.13 | 0.72 | 1.24 | 0.884 | 0.995 | 1.39 | 0.20 | 1.01 | 1.77 | 0.093 | 0.344 |
|  | 2 | 0.89 | 0.14 | 0.62 | 1.17 | 0.420 | 0.938 | 1.17 | 0.21 | 0.75 | 1.58 | 0.461 | 0.981 |
| Bullying | 1 | 1.49 | 0.12 | 1.26 | 1.72 | **0.001** | **0.010** | 1.83 | 0.17 | 1.50 | 2.15 | **0.000** | **0.007** |
|  | 2 | 1.44 | 0.12 | 1.21 | 1.67 | **0.002** | **0.024** | 1.69 | 0.17 | 1.36 | 2.02 | **0.002** | **0.024** |
| **Note.** Sample: ALSPAC (N = 3,931). Pooled estimates from multinomial logistic regression models across 20 imputed models. Reference outcome: Low depression trajectory. CI = confidence interval. SE = standard error. fdr = false discovery rate (p-value). ACEs = adverse childhood experiences. Model 1: adjusted for sex, ethnicity, maternal smoking during pregnancy, mother’s marital status, mother’s education, and household’s social class; Model 2: Model 1 + earlier ACEs. P-values highlighted in bold are statistically significant at the 95% confidence level. | | | | | | | | | | | | | |

| **Table S17. Associations of ACEs in adolescence (12-18 yrs) with moderate-high and high-moderate CRP trajectories (vs low-moderate).** | | | | | | | | | | | | | |
| --- | --- | --- | --- | --- | --- | --- | --- | --- | --- | --- | --- | --- | --- |
| **Outcome: CRP trajectories** | | **Moderate-high Trajectory** | | | | |  | **High-moderate Trajectory** | | | | | |
|  | **Model** | **OR** | **SE** | **Lower CI** | **Upper CI** | **p-value** | **p-value_fdr** | **OR** | **SE** | **Lower CI** | **Upper CI** | **p-value** | **p-value_fdr** |
|  |  |  |  |  |  |  |  |  |  |  |  |  |  |
| Total ACEs cumulative score | 1 | 1.07 | 0.04 | 0.99 | 1.15 | 0.104 | 0.246 | 1.07 | 0.06 | 0.96 | 1.19 | 0.209 | 0.454 |
|  | 2 | 1.08 | 0.04 | 0.99 | 1.16 | 0.097 | 0.235 | 1.07 | 0.06 | 0.95 | 1.19 | 0.256 | 0.502 |
| ***ACEs dimensions and single adversities*** |  |  |  |  |  |  |  |  |  |  |  |  |  |
| Emotional/ Physical threat | 1 | 0.96 | 0.09 | 0.78 | 1.13 | 0.617 | 0.804 | 1.03 | 0.13 | 0.78 | 1.28 | 0.810 | 0.887 |
|  | 2 | 0.95 | 0.09 | 0.76 | 1.13 | 0.559 | 0.775 | 1.01 | 0.13 | 0.76 | 1.27 | 0.916 | 0.972 |
| - Physical abuse | 1 | 1.15 | 0.16 | 0.83 | 1.47 | 0.391 | 0.603 | 1.22 | 0.21 | 0.81 | 1.64 | 0.337 | 0.599 |
|  | 2 | 1.17 | 0.17 | 0.85 | 1.50 | 0.340 | 0.599 | 1.21 | 0.21 | 0.78 | 1.63 | 0.383 | 0.603 |
| - Emotional abuse/neglect | 1 | 0.91 | 0.10 | 0.71 | 1.12 | 0.394 | 0.603 | 1.07 | 0.14 | 0.81 | 1.34 | 0.610 | 0.804 |
|  | 2 | 0.91 | 0.11 | 0.70 | 1.12 | 0.374 | 0.599 | 1.06 | 0.14 | 0.79 | 1.33 | 0.667 | 0.814 |
| - Low Parent-Child Bonding | 1 | 1.03 | 0.13 | 0.77 | 1.29 | 0.804 | 0.887 | 1.01 | 0.18 | 0.65 | 1.37 | 0.970 | 0.994 |
|  | 2 | 1.02 | 0.14 | 0.75 | 1.29 | 0.878 | 0.951 | 0.98 | 0.19 | 0.62 | 1.35 | 0.930 | 0.977 |
| Household Dysfunction | 1 | 1.27 | 0.11 | 1.05 | 1.49 | **0.030** | 0.086 | 1.14 | 0.17 | 0.80 | 1.48 | 0.440 | 0.654 |
|  | 2 | 1.28 | 0.11 | 1.06 | 1.51 | **0.029** | 0.084 | 1.13 | 0.18 | 0.79 | 1.48 | 0.480 | 0.693 |
| - Parental Substance Use Problems | 1 | 1.47 | 0.38 | 0.73 | 2.21 | 0.304 | 0.577 | 0.75 | 0.61 | -0.45 | 1.94 | 0.634 | 0.814 |
|  | 2 | 1.47 | 0.38 | 0.73 | 2.22 | 0.305 | 0.577 | 0.74 | 0.61 | -0.46 | 1.94 | 0.618 | 0.804 |
| - Parental Mental Health Problems | 1 | 1.31 | 0.29 | 0.74 | 1.89 | 0.352 | 0.599 | 1.21 | 0.46 | 0.32 | 2.10 | 0.673 | 0.814 |
|  | 2 | 1.34 | 0.31 | 0.74 | 1.94 | 0.341 | 0.599 | 1.18 | 0.47 | 0.26 | 2.11 | 0.721 | 0.824 |
| - Parental Separation | 1 | 1.27 | 0.14 | 1.00 | 1.54 | 0.082 | 0.208 | 1.11 | 0.19 | 0.73 | 1.48 | 0.597 | 0.804 |
|  | 2 | 1.27 | 0.14 | 0.99 | 1.54 | 0.090 | 0.222 | 1.09 | 0.19 | 0.71 | 1.46 | 0.667 | 0.814 |
| Sexual abuse | 1 | 1.09 | 0.19 | 0.71 | 1.47 | 0.653 | 0.814 | 0.91 | 0.26 | 0.39 | 1.42 | 0.707 | 0.824 |
|  | 2 | 1.09 | 0.19 | 0.72 | 1.47 | 0.644 | 0.814 | 0.90 | 0.26 | 0.38 | 1.42 | 0.681 | 0.814 |
| Bullying | 1 | 1.11 | 0.11 | 0.88 | 1.33 | 0.372 | 0.599 | 1.15 | 0.14 | 0.87 | 1.43 | 0.340 | 0.599 |
|  | 2 | 1.11 | 0.11 | 0.88 | 1.33 | 0.371 | 0.599 | 1.14 | 0.14 | 0.86 | 1.43 | 0.350 | 0.599 |
| **Note.** Sample: ALSPAC (N = 3,931). Pooled estimates from multinomial logistic regression models across 20 imputed models. Reference outcome: Low-moderate CRP trajectory. CI = confidence interval. SE = standard error. fdr = false discovery rate (p-value). ACEs = adverse childhood experiences. CRP = C-reactive protein. Model 1: adjusted for sex, ethnicity, maternal smoking during pregnancy, mother’s marital status, mother’s education, and household’s social class; Model 2: Model 1 + earlier ACEs. P-values highlighted in bold are statistically significant at the 95% confidence level. | | | | | | | | | | | | | |
|  | | | | | | | | | | | | | |

| **Table S18. Associations of ACEs in adolescence (12-18 yrs) with moderate and severe depression trajectories (vs low).** | | | | | | | | | | | | | |
| --- | --- | --- | --- | --- | --- | --- | --- | --- | --- | --- | --- | --- | --- |
| **Outcome: Depression trajectories** | | **Moderate Trajectory** | | | | | | **Severe Trajectory** | | | | | |
|  | **Model** | **OR** | **SE** | **Lower CI** | **Upper CI** | **p-value** | **p-value_fdr** | **OR** | **SE** | **Lower CI** | **Upper CI** | **p-value** | **p-value_fdr** |
|  |  |  |  |  |  |  |  |  |  |  |  |  |  |
| Total ACEs cumulative score | 1 | 1.47 | 0.04 | 1.38 | 1.55 | **0.000** | **0.000** | 1.87 | 0.06 | 1.76 | 1.99 | **0.000** | **0.000** |
|  | 2 | 1.43 | 0.05 | 1.34 | 1.52 | **0.000** | **0.000** | 1.79 | 0.07 | 1.67 | 1.92 | **0.000** | **0.000** |
| ***ACEs dimensions and single adversities*** |  |  |  |  |  |  |  |  |  |  |  |  |  |
| Emotional/ Physical threat | 1 | 1.47 | 0.09 | 1.29 | 1.64 | **0.000** | **0.000** | 2.46 | 0.13 | 2.21 | 2.72 | **0.000** | **0.000** |
|  | 2 | 1.39 | 0.09 | 1.21 | 1.56 | **0.000** | **0.001** | 2.23 | 0.13 | 1.96 | 2.49 | **0.000** | **0.000** |
| - Physical abuse | 1 | 1.98 | 0.16 | 1.67 | 2.29 | **0.000** | **0.000** | 5.54 | 0.18 | 5.18 | 5.90 | **0.000** | **0.000** |
|  | 2 | 1.81 | 0.16 | 1.50 | 2.13 | **0.000** | **0.001** | 4.83 | 0.19 | 4.46 | 5.20 | **0.000** | **0.000** |
| - Emotional abuse/neglect | 1 | 1.38 | 0.10 | 1.19 | 1.57 | **0.001** | **0.004** | 2.14 | 0.14 | 1.86 | 2.41 | **0.000** | **0.000** |
|  | 2 | 1.32 | 0.10 | 1.12 | 1.51 | **0.006** | **0.019** | 1.96 | 0.14 | 1.68 | 2.24 | **0.000** | **0.000** |
| - Low Parent-Child Bonding | 1 | 1.45 | 0.12 | 1.20 | 1.69 | **0.003** | **0.011** | 2.04 | 0.18 | 1.70 | 2.39 | **0.000** | **0.000** |
|  | 2 | 1.33 | 0.13 | 1.08 | 1.58 | **0.026** | 0.077 | 1.75 | 0.19 | 1.39 | 2.11 | **0.003** | **0.009** |
| Household Dysfunction | 1 | 1.38 | 0.12 | 1.14 | 1.62 | **0.008** | **0.025** | 1.82 | 0.17 | 1.48 | 2.15 | **0.001** | **0.002** |
|  | 2 | 1.27 | 0.13 | 1.02 | 1.52 | 0.057 | 0.151 | 1.57 | 0.19 | 1.21 | 1.93 | **0.015** | **0.045** |
| - Parental Substance Use Problems | 1 | 1.28 | 0.39 | 0.53 | 2.04 | 0.521 | 0.732 | 2.53 | 0.45 | 1.65 | 3.42 | **0.040** | 0.109 |
|  | 2 | 1.23 | 0.39 | 0.47 | 1.99 | 0.595 | 0.804 | 2.39 | 0.46 | 1.48 | 3.30 | 0.061 | 0.158 |
| - Parental Mental Health Problems | 1 | 1.44 | 0.29 | 0.87 | 2.01 | 0.207 | 0.454 | 1.70 | 0.41 | 0.90 | 2.49 | 0.194 | 0.445 |
|  | 2 | 1.10 | 0.29 | 0.52 | 1.67 | 0.748 | 0.845 | 1.00 | 0.43 | 0.15 | 1.85 | 0.997 | 0.997 |
| - Parental Separation | 1 | 1.11 | 0.14 | 0.83 | 1.39 | 0.458 | 0.671 | 1.18 | 0.20 | 0.78 | 1.58 | 0.417 | 0.628 |
|  | 2 | 1.02 | 0.15 | 0.72 | 1.31 | 0.912 | 0.972 | 1.01 | 0.21 | 0.60 | 1.43 | 0.955 | 0.994 |
| Sexual abuse | 1 | 3.16 | 0.18 | 2.80 | 3.52 | **0.000** | **0.000** | 4.43 | 0.22 | 3.99 | 4.87 | **0.000** | **0.000** |
|  | 2 | 3.09 | 0.18 | 2.73 | 3.45 | **0.000** | **0.000** | 4.29 | 0.23 | 3.85 | 4.74 | **0.000** | **0.000** |
| Bullying | 1 | 2.16 | 0.10 | 1.97 | 2.36 | **0.000** | **0.000** | 2.14 | 0.16 | 1.83 | 2.45 | **0.000** | **0.000** |
|  | 2 | 2.12 | 0.10 | 1.92 | 2.32 | **0.000** | **0.000** | 2.01 | 0.16 | 1.69 | 2.33 | **0.000** | **0.000** |
| **Note.** Sample: ALSPAC (N = 3,931). Pooled estimates from multinomial logistic regression models across 20 imputed models. Reference outcome: Low depression trajectory. CI = confidence interval. SE = standard error. fdr = false discovery rate (p-value). ACEs = adverse childhood experiences. Model 1: adjusted for sex, ethnicity, maternal smoking during pregnancy, mother’s marital status, mother’s education, and household’s social class; Model 2: Model 1 + earlier ACEs. P-values highlighted in bold are statistically significant at the 95% confidence level. | | | | | | | | | | | | | |

| **Table S19. Associations of any ACEs exposure throughout childhood (prenatal-18yrs) with moderate-high and high-moderate CRP trajectories (vs low-moderate).** | | | | | | | | | | | | | |
| --- | --- | --- | --- | --- | --- | --- | --- | --- | --- | --- | --- | --- | --- |
| **Outcome: CRP trajectories** |  | **Moderate-high Trajectory** | | | | | | **High-moderate Trajectory** | | | | | |
|  | **Model** | **OR** | **SE** | **Lower CI** | **Upper CI** | **p-value** | **p-value_fdr** | **OR** | **SE** | **Lower CI** | **Upper CI** | **p-value** | **p-value_fdr** |
|  |  |  |  |  |  |  |  |  |  |  |  |  |  |
| Total ACEs cumulative score | 1 | 1.01 | 0.03 | 0.96 | 1.06 | 0.720 | 0.782 | 1.02 | 0.03 | 0.96 | 1.09 | 0.480 | 0.713 |
| ***ACEs dimensions and single adversities*** |  |  |  |  |  |  |  |  |  |  |  |  |  |
| Emotional/ Physical threat | 1 | 0.95 | 0.09 | 0.78 | 1.12 | 0.560 | 0.777 | 0.96 | 0.11 | 0.74 | 1.18 | 0.700 | 0.782 |
| - Physical abuse | 1 | 0.93 | 0.14 | 0.67 | 1.20 | 0.605 | 0.777 | 1.16 | 0.16 | 0.84 | 1.48 | 0.373 | 0.646 |
| - Emotional abuse/neglect | 1 | 0.92 | 0.09 | 0.74 | 1.10 | 0.386 | 0.648 | 0.94 | 0.12 | 0.70 | 1.18 | 0.597 | 0.777 |
| - Low Parent-Child Bonding | 1 | 1.03 | 0.10 | 0.83 | 1.23 | 0.778 | 0.826 | 0.95 | 0.14 | 0.69 | 1.22 | 0.722 | 0.782 |
| Household Dysfunction | 1 | 0.96 | 0.10 | 0.76 | 1.15 | 0.658 | 0.778 | 1.07 | 0.13 | 0.82 | 1.32 | 0.585 | 0.777 |
| - Household violence | 1 | 1.15 | 0.10 | 0.94 | 1.35 | 0.188 | 0.350 | 1.05 | 0.13 | 0.79 | 1.31 | 0.706 | 0.782 |
| - Parental Substance Use Problems | 1 | 0.81 | 0.15 | 0.51 | 1.10 | 0.155 | 0.298 | 1.28 | 0.17 | 0.95 | 1.61 | 0.138 | 0.275 |
| - Parental Mental Health Problems | 1 | 0.93 | 0.09 | 0.75 | 1.11 | 0.431 | 0.659 | 0.91 | 0.11 | 0.69 | 1.13 | 0.417 | 0.659 |
| - Parental Convictions | 1 | 1.04 | 0.16 | 0.72 | 1.36 | 0.815 | 0.831 | 0.87 | 0.22 | 0.44 | 1.30 | 0.530 | 0.765 |
| - Parental Separation | 1 | 1.17 | 0.10 | 0.97 | 1.37 | 0.115 | 0.240 | 1.06 | 0.13 | 0.81 | 1.31 | 0.641 | 0.778 |
| Sexual abuse | 1 | 1.09 | 0.19 | 0.72 | 1.46 | 0.644 | 0.778 | 0.88 | 0.25 | 0.38 | 1.38 | 0.613 | 0.777 |
| Bullying | 1 | 1.09 | 0.10 | 0.90 | 1.28 | 0.359 | 0.644 | 1.35 | 0.12 | 1.12 | 1.58 | **0.012** | **0.030** |
| **Note.** Sample: ALSPAC (N = 3,931). Pooled estimates from multinomial logistic regression models across 20 imputed models. Reference outcome: Low-moderate CRP trajectory. CI = confidence interval. SE = standard error. fdr = false discovery rate (p-value). ACEs = adverse childhood experiences. CRP = C-reactive protein. Model 1: adjusted for sex, ethnicity, maternal smoking during pregnancy, mother’s marital status, mother’s education, and household’s social class. P-values highlighted in bold are statistically significant at the 95% confidence level. | | | | | | | | | | | | | |

| **Table S20. Associations of any ACEs exposure throughout childhood (prenatal-18yrs) with moderate and severe depression trajectories (vs low).** | | | | | | | | | | | | | |
| --- | --- | --- | --- | --- | --- | --- | --- | --- | --- | --- | --- | --- | --- |
| **Outcome: Depression trajectories** | | **Moderate Trajectory** | | | | | | **Severe Trajectory** | | | | | |
|  | **Model** | **OR** | **SE** | **Lower CI** | **Upper CI** | **p-value** | **p-value_fdr** | **OR** | **SE** | **Lower CI** | **Upper CI** | **p-value** | **p-value_fdr** |
|  |  |  |  |  |  |  |  |  |  |  |  |  |  |
| Total ACEs cumulative score | 1 | 1.24 | 0.03 | 1.19 | 1.29 | **0.000** | **0.000** | 1.51 | 0.04 | 1.43 | 1.58 | **0.000** | **0.000** |
| ***ACEs dimensions and single adversities*** | |  |  |  |  |  |  |  |  |  |  |  |  |
| Emotional/ Physical threat | 1 | 1.48 | 0.09 | 1.32 | 1.65 | **0.000** | **0.000** | 2.56 | 0.16 | 2.26 | 2.87 | **0.000** | **0.000** |
| - Physical abuse | 1 | 1.59 | 0.12 | 1.35 | 1.83 | **0.000** | **0.000** | 3.56 | 0.16 | 3.26 | 3.87 | **0.000** | **0.000** |
| - Emotional abuse/neglect | 1 | 1.54 | 0.09 | 1.38 | 1.71 | **0.000** | **0.000** | 2.33 | 0.14 | 2.06 | 2.60 | **0.000** | **0.000** |
| - Low Parent-Child Bonding | 1 | 1.28 | 0.09 | 1.10 | 1.47 | **0.007** | **0.019** | 1.82 | 0.14 | 1.54 | 2.10 | **0.000** | **0.000** |
| Household Dysfunction | 1 | 1.52 | 0.11 | 1.31 | 1.73 | **0.000** | **0.000** | 2.06 | 0.19 | 1.70 | 2.42 | **0.000** | **0.000** |
| - Household violence | 1 | 1.26 | 0.10 | 1.06 | 1.45 | **0.020** | **0.046** | 1.75 | 0.15 | 1.45 | 2.05 | **0.000** | **0.001** |
| - Parental Substance Use Problems | 1 | 1.31 | 0.12 | 1.07 | 1.54 | **0.027** | 0.061 | 1.48 | 0.19 | 1.11 | 1.86 | **0.038** | 0.083 |
| - Parental Mental Health Problems | 1 | 1.60 | 0.08 | 1.43 | 1.76 | **0.000** | **0.000** | 2.01 | 0.14 | 1.74 | 2.28 | **0.000** | **0.000** |
| - Parental Convictions | 1 | 0.98 | 0.16 | 0.67 | 1.29 | 0.892 | 0.892 | 1.20 | 0.22 | 0.76 | 1.63 | 0.421 | 0.659 |
| - Parental Separation | 1 | 0.98 | 0.10 | 0.79 | 1.16 | 0.796 | 0.828 | 1.55 | 0.15 | 1.26 | 1.84 | **0.003** | **0.009** |
| Sexual abuse | 1 | 2.92 | 0.18 | 2.57 | 3.27 | **0.000** | **0.000** | 4.43 | 0.21 | 4.01 | 4.84 | **0.000** | **0.000** |
| Bullying | 1 | 1.89 | 0.09 | 1.72 | 2.06 | **0.000** | **0.000** | 1.95 | 0.14 | 1.68 | 2.22 | **0.000** | **0.000** |
| **Note.** Sample: ALSPAC (N = 3,931). Pooled estimates from multinomial logistic regression models across 20 imputed models. Reference outcome: Low depression trajectory. CI = confidence interval. SE = standard error. fdr = false discovery rate (p-value). ACEs = adverse childhood experiences. Model 1: adjusted for sex, ethnicity, maternal smoking during pregnancy, mother’s marital status, mother’s education, and household’s social class. P-values highlighted in bold are statistically significant at the 95% confidence level. | | | | | | | | | | | | | |

| **Table S21. Associations of single-exposure and multiple-exposure to ACEs throughout childhood (prenatal-18yrs) with moderate-high and high-moderate CRP trajectories (vs low-moderate).** | | | | | | | | | | | | | |
| --- | --- | --- | --- | --- | --- | --- | --- | --- | --- | --- | --- | --- | --- |
| **Outcome: CRP trajectories** |  | **Moderate-high Trajectory** | | | | | | **High-moderate Trajectory** | | | | | |
|  |  | **OR** | **SE** | **Lower CI** | **Upper CI** | **p-value** | **p-value_fdr** | **OR** | **SE** | **Lower CI** | **Upper CI** | **p-value** | **p-value_fdr** |
| Total ACEs cumulative score | Single-exposure | 1.035 | 0.173 | 0.696 | 1.375 | 0.842 | 0.915 | 1.213 | 0.212 | 0.798 | 1.628 | 0.362 | 0.673 |
|  | Multiple-exposure | 1.028 | 0.127 | 0.778 | 1.277 | 0.829 | 0.915 | 1.291 | 0.168 | 0.962 | 1.621 | 0.128 | 0.272 |
| ***ACEs dimensions and single adversities*** |  |  |  |  |  |  |  |  |  |  |  |  |  |
| Emotional/ Physical threat | Single-exposure | 0.952 | 0.107 | 0.741 | 1.162 | 0.645 | 0.839 | 0.981 | 0.139 | 0.709 | 1.254 | 0.892 | 0.919 |
|  | Multiple-exposure | 0.940 | 0.109 | 0.726 | 1.154 | 0.571 | 0.803 | 0.955 | 0.139 | 0.684 | 1.227 | 0.742 | 0.888 |
| - Physical abuse | Single-exposure | 0.979 | 0.152 | 0.680 | 1.278 | 0.890 | 0.919 | 1.109 | 0.185 | 0.747 | 1.471 | 0.575 | 0.803 |
|  | Multiple-exposure | 0.838 | 0.319 | 0.214 | 1.463 | 0.580 | 0.803 | 1.404 | 0.321 | 0.776 | 2.033 | 0.289 | 0.579 |
| - Emotional abuse/neglect | Single-exposure | 0.922 | 0.102 | 0.722 | 1.123 | 0.430 | 0.721 | 0.964 | 0.139 | 0.693 | 1.236 | 0.794 | 0.915 |
|  | Multiple-exposure | 0.886 | 0.149 | 0.594 | 1.178 | 0.417 | 0.718 | 0.887 | 0.194 | 0.506 | 1.268 | 0.536 | 0.802 |
| - Low Parent-Child Bonding | Single-exposure | 1.032 | 0.152 | 0.735 | 1.329 | 0.834 | 0.915 | 0.840 | 0.206 | 0.436 | 1.245 | 0.399 | 0.715 |
|  | Multiple-exposure | 1.025 | 0.123 | 0.783 | 1.266 | 0.843 | 0.915 | 1.102 | 0.166 | 0.777 | 1.427 | 0.557 | 0.803 |
| Household Dysfunction | Single-exposure | 0.974 | 0.133 | 0.714 | 1.235 | 0.844 | 0.915 | 1.232 | 0.161 | 0.916 | 1.547 | 0.196 | 0.407 |
|  | Multiple-exposure | 1.039 | 0.106 | 0.832 | 1.247 | 0.716 | 0.886 | 1.070 | 0.137 | 0.801 | 1.340 | 0.622 | 0.831 |
| - Household violence | Single-exposure | 1.283 | 0.130 | 1.027 | 1.538 | 0.056 | 0.149 | 1.205 | 0.189 | 0.834 | 1.575 | 0.324 | 0.624 |
|  | Multiple-exposure | 0.936 | 0.207 | 0.531 | 1.342 | 0.751 | 0.888 | 1.088 | 0.253 | 0.592 | 1.584 | 0.739 | 0.888 |
| - Parental Substance Use Problems | Single-exposure | 0.909 | 0.169 | 0.578 | 1.241 | 0.574 | 0.803 | 1.224 | 0.212 | 0.809 | 1.639 | 0.339 | 0.642 |
|  | Multiple-exposure | 0.650 | 0.268 | 0.126 | 1.174 | 0.107 | 0.253 | 1.536 | 0.263 | 1.020 | 2.052 | 0.103 | 0.253 |
| - Parental Mental Health Problems | Single-exposure | 0.912 | 0.137 | 0.645 | 1.180 | 0.502 | 0.783 | 1.014 | 0.160 | 0.701 | 1.327 | 0.930 | 0.949 |
|  | Multiple-exposure | 0.918 | 0.107 | 0.709 | 1.127 | 0.421 | 0.718 | 0.905 | 0.136 | 0.639 | 1.172 | 0.466 | 0.767 |
| - Parental Convictions | Single-exposure | 1.027 | 0.194 | 0.647 | 1.406 | 0.892 | 0.919 | 1.059 | 0.249 | 0.570 | 1.547 | 0.819 | 0.915 |
|  | Multiple-exposure | 1.015 | 0.287 | 0.452 | 1.578 | 0.959 | 0.968 | 0.324 | 0.644 | -0.939 | 1.587 | 0.080 | 0.203 |
| - Parental Separation | Single-exposure | 1.117 | 0.136 | 0.851 | 1.383 | 0.414 | 0.718 | 1.100 | 0.176 | 0.756 | 1.444 | 0.587 | 0.803 |
|  | Multiple-exposure | 1.225 | 0.131 | 0.969 | 1.482 | 0.121 | 0.268 | 1.038 | 0.176 | 0.692 | 1.383 | 0.834 | 0.915 |
| Sexual abuse * | Single-exposure | 1.076 | 0.186 | 0.713 | 1.440 | 0.692 | 0.882 | 0.910 | 0.254 | 0.412 | 1.408 | 0.711 | 0.886 |
| Bullying | Single-exposure | 1.077 | 0.103 | 0.876 | 1.277 | 0.472 | 0.767 | 1.324 | 0.126 | 1.077 | 1.571 | **0.026** | **0.049** |
|  | Multiple-exposure | 1.048 | 0.255 | 0.548 | 1.548 | 0.853 | 0.915 | 1.317 | 0.309 | 0.713 | 1.922 | 0.372 | 0.678 |
| **Note.** Sample: ALSPAC (N = 3,931). Pooled estimates from multinomial logistic regression models across 20 imputed models. Reference outcome: Low-moderate CRP trajectory. CI = confidence interval. SE = standard error. fdr = false discovery rate (p-value). ACEs = adverse childhood experiences. CRP = C-reactive protein. Model 4: adjusted for sex, ethnicity, maternal smoking during pregnancy, mother’s marital status, mother’s education, and household’s social class. P-values highlighted in bold are statistically significant at the 95% confidence level.  * The Multiple-exposure group was not included due to limited sample size. | | | | | | | | | | | | | |

| **Table S22. Associations of single-exposure and multi-exposure to ACEs throughout childhood (prenatal-18yrs) with moderate and severe depression trajectories (vs low).** | | | | | | | | | | | | | |
| --- | --- | --- | --- | --- | --- | --- | --- | --- | --- | --- | --- | --- | --- |
| **Outcome: Depression trajectories** | | **Moderate Trajectory** | | | | | | **Severe Trajectory** | | | | | |
|  | **Model** | **OR** | **SE** | **Lower CI** | **Upper CI** | **p-value** | **p-value_fdr** | **OR** | **SE** | **Lower CI** | **Upper CI** | **p-value** | **p-value_fdr** |
| Total ACEs cumulative score | Single-exposure | 1.316 | 0.179 | 0.966 | 1.667 | 0.124 | 0.269 | 1.846 | 0.334 | 1.191 | 2.500 | 0.066 | 0.173 |
|  | Multiple-exposure | 2.103 | 0.134 | 1.840 | 2.365 | **0.000** | **0.000** | 4.278 | 0.278 | 3.732 | 4.823 | **0.000** | **0.000** |
| ***ACEs dimensions and single adversities*** |  |  |  |  |  |  |  |  |  |  |  |  |  |
| Emotional/ Physical threat | Single-exposure | 1.507 | 0.103 | 1.305 | 1.709 | **0.000** | **0.000** | 1.985 | 0.178 | 1.636 | 2.335 | **0.000** | **0.001** |
|  | Multiple-exposure | 1.502 | 0.099 | 1.307 | 1.697 | **0.000** | **0.000** | 2.982 | 0.162 | 2.665 | 3.299 | **0.000** | **0.000** |
| - Physical abuse | Single-exposure | 1.563 | 0.133 | 1.303 | 1.823 | **0.001** | **0.003** | 3.664 | 0.165 | 3.339 | 3.988 | **0.000** | **0.000** |
|  | Multiple-exposure | 1.634 | 0.254 | 1.137 | 2.131 | 0.053 | 0.145 | 2.787 | 0.343 | 2.115 | 3.460 | **0.003** | **0.011** |
| - Emotional abuse/neglect | Single-exposure | 1.475 | 0.096 | 1.286 | 1.663 | **0.000** | **0.000** | 2.071 | 0.157 | 1.763 | 2.378 | **0.000** | **0.000** |
|  | Multiple-exposure | 1.646 | 0.132 | 1.388 | 1.904 | **0.000** | **0.001** | 2.767 | 0.189 | 2.397 | 3.138 | **0.000** | **0.000** |
| - Low Parent-Child Bonding | Single-exposure | 1.466 | 0.129 | 1.214 | 1.719 | **0.003** | **0.012** | 1.556 | 0.212 | 1.140 | 1.971 | **0.037** | 0.110 |
|  | Multiple-exposure | 1.207 | 0.119 | 0.974 | 1.440 | 0.113 | 0.261 | 2.059 | 0.166 | 1.734 | 2.384 | **0.000** | **0.000** |
| Household Dysfunction | Single-exposure | 1.280 | 0.122 | 1.040 | 1.519 | **0.043** | 0.125 | 1.589 | 0.221 | 1.156 | 2.022 | **0.036** | 0.110 |
|  | Multiple-exposure | 1.619 | 0.102 | 1.419 | 1.819 | **0.000** | **0.000** | 2.644 | 0.178 | 2.295 | 2.994 | **0.000** | **0.000** |
| - Household violence | Single-exposure | 1.349 | 0.151 | 1.052 | 1.645 | **0.048** | 0.135 | 2.042 | 0.195 | 1.660 | 2.423 | **0.000** | **0.001** |
|  | Multiple-exposure | 1.332 | 0.177 | 0.985 | 1.678 | 0.105 | 0.253 | 2.192 | 0.263 | 1.676 | 2.708 | **0.003** | **0.011** |
| - Parental Substance Use Problems | Single-exposure | 1.114 | 0.162 | 0.796 | 1.431 | 0.507 | 0.783 | 1.180 | 0.270 | 0.651 | 1.710 | 0.539 | 0.802 |
|  | Multiple-exposure | 1.735 | 0.197 | 1.350 | 2.121 | **0.005** | **0.019** | 1.938 | 0.299 | 1.352 | 2.524 | **0.027** | 0.087 |
| - Parental Mental Health Problems | Single-exposure | 1.320 | 0.119 | 1.086 | 1.553 | **0.020** | 0.068 | 1.392 | 0.212 | 0.976 | 1.807 | 0.119 | 0.268 |
|  | Multiple-exposure | 1.721 | 0.094 | 1.536 | 1.906 | **0.000** | **0.000** | 2.232 | 0.151 | 1.936 | 2.529 | **0.000** | **0.000** |
| - Parental Convictions | Single-exposure | 0.928 | 0.190 | 0.557 | 1.300 | 0.695 | 0.882 | 1.096 | 0.281 | 0.545 | 1.647 | 0.744 | 0.888 |
|  | Multiple-exposure | 1.209 | 0.271 | 0.677 | 1.741 | 0.484 | 0.775 | 1.226 | 0.432 | 0.380 | 2.072 | 0.637 | 0.838 |
| - Parental Separation | Single-exposure | 0.916 | 0.133 | 0.655 | 1.178 | 0.512 | 0.783 | 1.518 | 0.191 | 1.144 | 1.891 | **0.029** | 0.090 |
|  | Multiple-exposure | 1.065 | 0.129 | 0.813 | 1.318 | 0.623 | 0.831 | 1.665 | 0.185 | 1.302 | 2.029 | **0.006** | **0.021** |
| Sexual abuse * | Single-exposure | 2.802 | 0.177 | 2.454 | 3.149 | **0.000** | **0.000** | 4.097 | 0.215 | 3.676 | 4.518 | **0.000** | **0.000** |
| Bullying | Single-exposure | 1.789 | 0.092 | 1.609 | 1.969 | **0.000** | **0.000** | 1.757 | 0.149 | 1.466 | 2.048 | **0.000** | **0.001** |
|  | Multiple-exposure | 2.937 | 0.226 | 2.495 | 3.380 | **0.000** | **0.000** | 3.981 | 0.288 | 3.417 | 4.545 | **0.000** | **0.000** |
| **Note.** Sample: ALSPAC (N = 3,931). Pooled estimates from multinomial logistic regression models across 20 imputed models. Reference outcome: Low depression trajectory. CI = confidence interval. SE = standard error. fdr = false discovery rate (p-value). ACEs = adverse childhood experiences. CRP = C-reactive protein. Model 4: adjusted for sex, ethnicity, maternal smoking during pregnancy, mother’s marital status, mother’s education, and household’s social class. P-values highlighted in bold are statistically significant at the 95% confidence level.  * The Multiple-exposure group was not included due to limited sample size. | | | | | | | | | | | | | |

| **Table S23. Mutually adjusted associations of the ACEs dimensions with moderate-high and high-moderate CRP trajectories (vs low-moderate).** | | | | | | | | |
| --- | --- | --- | --- | --- | --- | --- | --- | --- |
| **Outcome: CRP trajectories** | **Moderate-High Trajectory** | | |  | **High-moderate Trajectory** | | |  |
|  | **OR** | **Lower CI** | **Upper CI** | **p-value** | **OR** | **Lower CI** | **Upper CI** | **p-value** |
| Household Dysfunction prenatal | 0.80 | 0.66 | 0.97 | **0.02** | 0.90 | 0.70 | 1.16 | 0.41 |
| Emotional/ Physical threat prenatal | 0.63 | 0.22 | 1.79 | 0.38 | 0.87 | 0.26 | 2.93 | 0.83 |
| Household Dysfunction 0-3yrs | 1.10 | 0.89 | 1.35 | 0.37 | 1.05 | 0.81 | 1.36 | 0.71 |
| Emotional/ Physical threat 0-3yrs | 0.92 | 0.70 | 1.20 | 0.53 | 0.79 | 0.54 | 1.14 | 0.21 |
| Household Dysfunction 3-7yrs | 1.04 | 0.84 | 1.29 | 0.71 | 1.09 | 0.83 | 1.43 | 0.55 |
| Emotional/ Physical threat 3-7yrs | 1.02 | 0.82 | 1.27 | 0.85 | 0.97 | 0.71 | 1.32 | 0.83 |
| Household Dysfunction 7-12yrs | 1.07 | 0.87 | 1.32 | 0.50 | 0.95 | 0.71 | 1.27 | 0.73 |
| Emotional/ Physical threat 7-12yrs | 1.00 | 0.79 | 1.25 | 0.98 | 0.95 | 0.70 | 1.28 | 0.73 |
| Household Dysfunction 12-18yrs | 1.29 | 1.03 | 1.61 | **0.03** | 1.13 | 0.80 | 1.60 | 0.48 |
| Emotional/ Physical threat 12-18yrs | 0.94 | 0.78 | 1.13 | 0.53 | 1.01 | 0.78 | 1.31 | 0.93 |
| Household Dysfunction prenatal-18yrs | 0.96 | 0.79 | 1.18 | 0.72 | 1.08 | 0.84 | 1.39 | 0.54 |
| Emotional/ Physical threat prenatal-18yrs | 0.96 | 0.81 | 1.13 | 0.61 | 0.95 | 0.76 | 1.18 | 0.63 |
| **Note.** Sample: ALSPAC (N=3,931). Pooled estimates from multinomial logistic regression models across 20 imputed models. Reference outcome: Low-moderate CRP trajectory. CI = confidence interval. ACEs = adverse childhood experiences. CRP = C-reactive protein. Model 5 - mutually adjusted effects of the ACEs dimensions, adjusted for all covariates and previous ACEs. | | | | | | | | |

| **Table S24. Mutually adjusted associations of the ACEs dimensions with moderate and severe depression trajectories (vs low).** | | | | | | | | | | |
| --- | --- | --- | --- | --- | --- | --- | --- | --- | --- | --- |
| **Outcome: Depression trajectories** | **Moderate Trajectory** | | | |  | **Severe Trajectory** | | | | |
|  | **OR** | **Lower CI** | **Upper CI** | **p-value** | **p-value difference*** | **OR** | **Lower CI** | **Upper CI** | **p-value** | **p-value difference*** |
| Household Dysfunction prenatal | 1.41 | 1.18 | 1.68 | **0.00** | 0.064 | 1.67 | 1.28 | 2.18 | **0.00** | 0.237 |
| Emotional/ Physical threat prenatal | 0.39 | 0.12 | 1.31 | 0.13 |  | 2.25 | 0.92 | 5.52 | 0.08 |  |
| Household Dysfunction 0-3yrs | 1.28 | 1.07 | 1.54 | **0.01** | 0.412 | 1.84 | 1.36 | 2.49 | **0.00** | 0.274 |
| Emotional/ Physical threat 0-3yrs | 1.18 | 0.92 | 1.52 | 0.18 |  | 1.51 | 1.05 | 2.16 | **0.02** |  |
| Household Dysfunction 3-7yrs | 1.16 | 0.97 | 1.40 | 0.11 | 0.403 | 1.25 | 0.92 | 1.70 | 0.16 | 0.145 |
| Emotional/ Physical threat 3-7yrs | 1.15 | 0.94 | 1.42 | 0.18 |  | 1.46 | 1.06 | 2.00 | **0.02** |  |
| Household Dysfunction 7-12yrs | 1.15 | 0.94 | 1.41 | 0.17 | 0.950 | 1.13 | 0.79 | 1.62 | 0.51 | 0.169 |
| Emotional/ Physical threat 7-12yrs | 1.03 | 0.83 | 1.27 | 0.81 |  | 1.43 | 1.05 | 1.96 | **0.02** |  |
| Household Dysfunction 12-18yrs | 1.27 | 0.99 | 1.63 | 0.06 | 0.074 | 1.54 | 1.07 | 2.24 | **0.02** | **0.003** |
| Emotional/ Physical threat 12-18yrs | 1.38 | 1.16 | 1.65 | **0.00** |  | 2.21 | 1.70 | 2.88 | **0.00** |  |
| Household Dysfunction prenatal-18yrs | 1.43 | 1.17 | 1.76 | **0.00** | 0.943 | 1.77 | 1.23 | 2.56 | **0.00** | **0.003** |
| Emotional/ Physical threat prenatal-18yrs | 1.42 | 1.20 | 1.68 | **0.00** |  | 2.39 | 1.76 | 3.24 | **0.00** |  |
| **Note.** Sample: ALSPAC (N=3,931). Pooled estimates from multinomial logistic regression models across 20 imputed models. Reference outcome: Low depression trajectory. CI = confidence interval. ACEs = adverse childhood experiences. Model 5 - mutually adjusted effects of the ACEs dimensions, adjusted for all covariates and previous ACEs.  * P-values of the differences in the magnitude of the associations of the ACEs dimensions with depression. | | | | | | | | | | |

| **Table S25. Interaction effects between ACEs and sex on CRP trajectories.** | | | | | | | | |
| --- | --- | --- | --- | --- | --- | --- | --- | --- |
| **Outcome: Moderate-High CRP Trajectory** |  | |  | |  | |  | |
|  | **OR** | **SE** | | **Lower CI** | **Upper CI** | **p-value** | | **p-value_fdr** |
| **ACEs prenatal** |  |  | |  |  |  | |  |
| ACEs cumulative score | 0.63 | 0.16 | | 0.33 | 0.94 | **0.003** | | **0.009** |
| Child sex: Female | 1.47 | 0.10 | | 1.28 | 1.67 | **0.000** | | **0.000** |
| ACEs cumulative score*Female | 1.45 | 0.18 | | 1.11 | 1.80 | **0.033** | | 0.084 |
| Household Dysfunction | 0.54 | 0.18 | | 0.18 | 0.89 | **0.001** | | **0.002** |
| Child sex: Female | 1.42 | 0.10 | | 1.23 | 1.62 | **0.000** | | **0.001** |
| Household Dysfunction*Female | 1.76 | 0.21 | | 1.34 | 2.17 | **0.008** | | **0.021** |
| **ACEs 0-3 yrs** |  |  | |  |  |  | |  |
| Household Dysfunction | 0.80 | 0.17 | | 0.48 | 1.13 | 0.188 | | 0.344 |
| Child sex: Female | 1.39 | 0.11 | | 1.18 | 1.60 | **0.003** | | **0.007** |
| Household Dysfunction*Female | 1.58 | 0.19 | | 1.21 | 1.96 | **0.015** | | **0.039** |
| **ACEs 3-7 yrs** |  |  | |  |  |  | |  |
| Household Dysfunction | 0.80 | 0.17 | | 0.47 | 1.13 | 0.190 | | 0.412 |
| Child sex:Female | 1.43 | 0.11 | | 1.22 | 1.64 | **0.001** | | **0.002** |
| Household Dysfunction*Female | 1.49 | 0.20 | | 1.11 | 1.88 | **0.043** | | 0.120 |
| Parental Mental Health Problems | 0.64 | 0.30 | | 0.04 | 1.24 | 0.140 | | 0.337 |
| Child sex: Female | 1.59 | 0.13 | | 1.34 | 1.84 | **0.000** | | **0.001** |
| Parental Mental Health Problems*Female | 2.12 | 0.34 | | 1.45 | 2.79 | **0.028** | | 0.080 |
| **ACEs 12-18 yrs** |  |  | |  |  |  | |  |
| Sexual abuse | 6.52 | 0.57 | | 5.39 | 7.64 | **0.001** | | **0.003** |
| Child sex:Female | 1.71 | 0.09 | | 1.54 | 1.89 | **0.000** | | **0.000** |
| Sexual abuse*Female | 0.13 | 0.62 | | 0.04 | 0.44 | **0.001** | | **0.003** |
| Parental Separation | 1.82 | 0.22 | | 1.40 | 2.25 | **0.006** | | **0.015** |
| Child sex:Female | 1.75 | 0.09 | | 1.57 | 1.93 | **0.000** | | **0.000** |
| Parental Separation*Female | 0.57 | 0.27 | | 0.04 | 1.11 | **0.041** | | 0.093 |
| **ACEs prenatal-18yrs** |  |  | |  |  |  | |  |
| Sexual abuse | 6.69 | 0.52 | | 5.67 | 7.71 | **0.000** | | **0.001** |
| Child sex | 1.70 | 0.09 | | 1.53 | 1.87 | **0.000** | | **0.000** |
| Sexual abuse*Child sex | 0.13 | 0.55 | | -0.96 | 1.21 | **0.000** | | **0.001** |
| Parental Mental Health Problems | 0.70 | 0.15 | | 0.41 | 0.99 | **0.016** | | **0.038** |
| Child sex | 1.31 | 0.12 | | 1.07 | 1.55 | **0.027** | | 0.060 |
| Parental Mental Health Problems*Child sex | 1.56 | 0.18 | | 1.20 | 1.92 | **0.015** | | **0.037** |
| **Note.** Sample: ALSPAC (N = 3,931). Pooled estimates from multinomial logistic regression models across 20 imputed models. Reference outcome: Low-moderate CRP trajectory. CI = confidence interval. SE = standard error. fdr = false discovery rate (p-value). ACEs = adverse childhood experiences. CRP = C-reactive protein. Model 3: adjusted for sex, ethnicity, maternal smoking during pregnancy, mother’s marital status, mother’s education, household’s social class, and earlier ACEs. P-values highlighted in bold are statistically significant at the 95% confidence level. Only statistically significant interaction effects (p < 0.05) are presented in the table. | | | | | | | | |

**
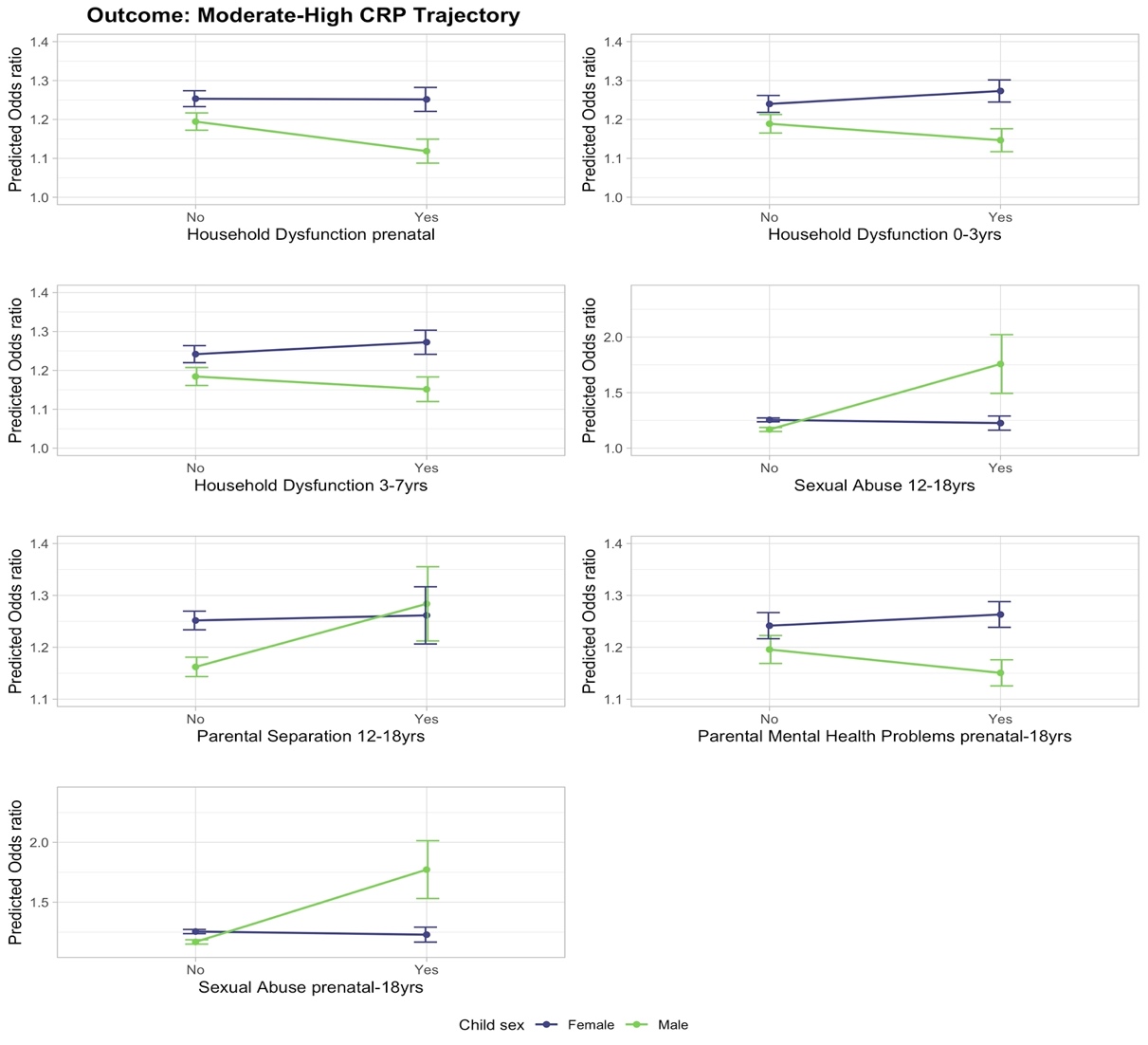
**

**Figure S4. Interaction effects between ACEs and sex on CRP trajectories.**

**Note.** Sample: ALSPAC (N = 3,931). Predicted odds ratios derived from multinomial logistic regression models adjusted for sex, ethnicity, maternal smoking during pregnancy, mother’s marital status, mother’s education, household’s social class, and earlier ACEs. Pooled estimates across 20 imputed datasets. Reference outcome: low-moderate CRP trajectory. Only statistically significant interaction effects (p < 0.05) are presented in the figure.

| **Table S26. Interaction effects between ACEs and sex on depression trajectories.** | | | | | | |
| --- | --- | --- | --- | --- | --- | --- |
| **Outcome: Moderate depression trajectory** |  | | | | | |
|  | **OR** | **SE** | **Lower CI** | **Upper CI** | **p-value** | **p-value_fdr** |
| **ACEs 0-3 yrs** |  |  |  |  |  |  |
| Low Parent-Child Bonding | 0.55 | 0.43 | -0.29 | 1.40 | 0.168 | 0.331 |
| Child sex: Female | 1.44 | 0.08 | 1.28 | 1.59 | **0.000** | **0.000** |
| Low Parent-Child Bonding*Female | 3.52 | 0.50 | 2.55 | 4.49 | **0.011** | **0.029** |
| **ACEs prenatal-18yrs** |  |  |  |  |  |  |
| Physical abuse | 1.12 | 0.20 | 0.72 | 1.52 | 0.589 | 0.733 |
| Child sex | 1.41 | 0.09 | 1.25 | 1.58 | **0.000** | **0.000** |
| Physical abuse*Child sex | 1.76 | 0.25 | 1.26 | 2.25 | **0.025** | 0.056 |
| **Note.** Sample: ALSPAC (N = 3,931). Pooled estimates from multinomial logistic regression models across 20 imputed models. Reference outcome: Low depression trajectory. CI = confidence interval. SE = standard error. fdr = false discovery rate (p-value). ACEs = adverse childhood experiences. Model 3: adjusted for sex, ethnicity, maternal smoking during pregnancy, mother’s marital status, mother’s education, household’s social class, and earlier ACEs. P-values highlighted in bold are statistically significant at the 95% confidence level. Only statistically significant interaction effects (p < 0.05) are presented in the table. | | | | | | |

**
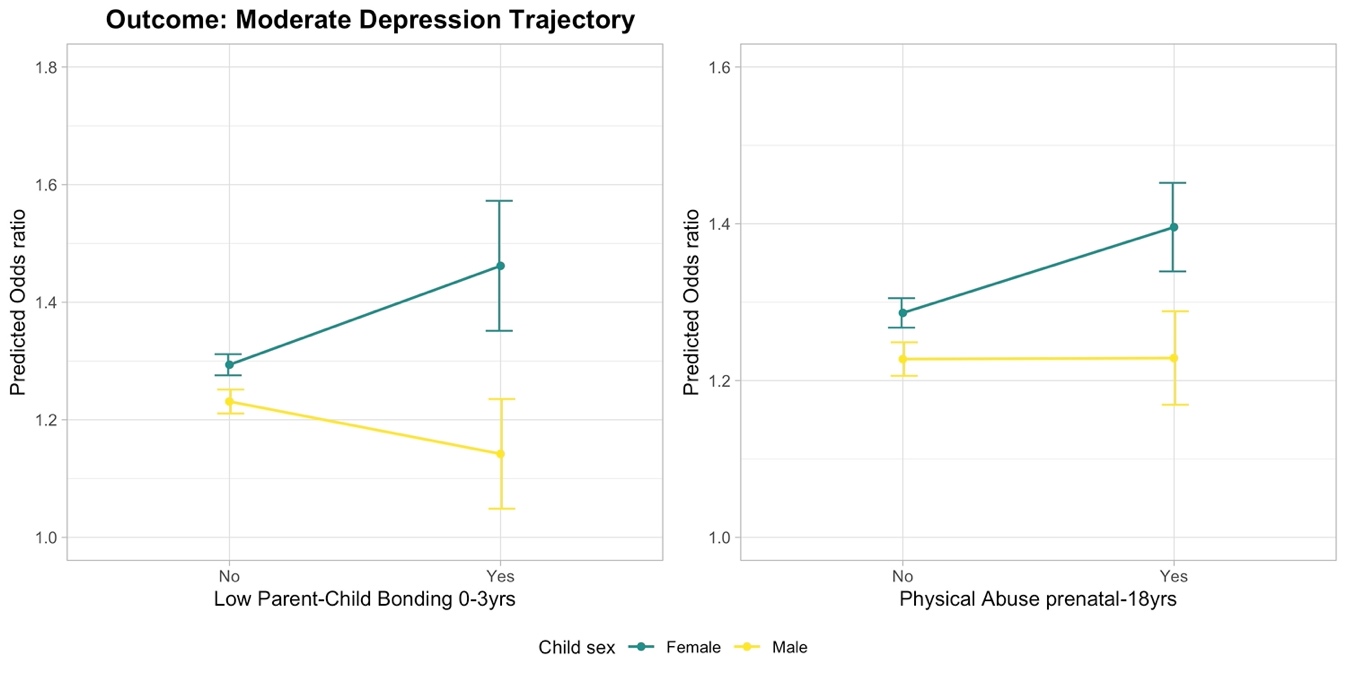
**

**Figure S5. Interaction effects between ACEs and sex on depression trajectories.**

**Note.** Sample: ALSPAC (N = 3,931). Predicted odds ratios derived from multinomial logistic regression models adjusted for sex, ethnicity, maternal smoking during pregnancy, mother’s marital status, mother’s education, household’s social class, and earlier ACEs. Pooled estimates across 20 imputed datasets. Reference outcome: low depression trajectory. Only statistically significant interaction effects (p < 0.05) are presented in the figure.

| **Table S27. Associations of the individual CRP measures and CRP trajectories with moderate and severe depression trajectories (vs low).** | | | | | | | | | | |
| --- | --- | --- | --- | --- | --- | --- | --- | --- | --- | --- |
| **Outcome: Depression trajectories** | **Moderate Trajectory** | | | | | **Severe Trajectory** | | | | |
|  | **OR** | **SE** | **Lower CI** | **Upper CI** | **p-value** | **OR** | **SE** | **Lower CI** | **Upper CI** | **p-value** |
| ***Single CRP measures*** |  |  |  |  |  |  |  |  |  |  |
| CRP 18 yrs (log) | 0.94 | 0.04 | 0.86 | 1.03 | 0.20 | 0.96 | 0.07 | 0.83 | 1.10 | 0.58 |
| CRP 15 yrs (log) | 0.99 | 0.05 | 0.90 | 1.08 | 0.85 | 1.00 | 0.07 | 0.86 | 1.15 | 0.98 |
| CRP 9 yrs (log) | 0.94 | 0.04 | 0.86 | 1.02 | 0.13 | 1.00 | 0.06 | 0.88 | 1.12 | 0.99 |
| Average CRP 9-18 yrs (log) | 0.90 | 0.06 | 0.79 | 1.02 | 0.10 | 0.97 | 0.10 | 0.80 | 1.19 | 0.79 |
| ***CRP Trajectories*** |  |  |  |  |  |  |  |  |  |  |
| Low CRP Trajectory | ref |  |  |  |  | ref |  |  |  |  |
| Moderate-High CRP Trajectory | 0.96 | 0.10 | 0.77 | 1.15 | 0.64 | 1.01 | 0.15 | 0.70 | 1.31 | 0.97 |
| High-Moderate CRP Trajectory | 0.85 | 0.13 | 0.59 | 1.10 | 0.21 | 1.19 | 0.19 | 0.83 | 1.56 | 0.34 |
| **Note.** Sample: ALSPAC (N = 3,931). Pooled estimates from multinomial logistic regression models across 20 imputed models. Reference outcome: Low depression trajectory. CI = confidence interval. SE = standard error. CRP = C-reactive protein. Model 1: adjusted for sex, ethnicity, maternal smoking during pregnancy, mother’s marital status, mother’s education, and household’s social class. P-values highlighted in bold are statistically significant at the 95% confidence level. | | | | | | | | | | |

| **Table S28. Associations of the individual CRP measures and CRP trajectories with the risk of depression (SMFQ total score ≥ 10) at 18, 21, and 23 years.** | | | | | | | | | | | | | | | |
| --- | --- | --- | --- | --- | --- | --- | --- | --- | --- | --- | --- | --- | --- | --- | --- |
|  | **Outcome: Depression, age 18 years** | | | | | **Outcome: Depression, age 21 years** | | | | | **Outcome: Depression, age 23 years** | | | | |
|  | **OR** | **SE** | **Lower CI** | **Upper CI** | **p-value** | **OR** | **SE** | **Lower CI** | **Upper CI** | **p-value** | **OR** | **SE** | **Lower CI** | **Upper CI** | **p-value** |
| ***Single CRP measures*** |  |  |  |  |  |  |  |  |  |  |  |  |  |  |  |
| CRP 18 yrs (log) | 0.97 | 0.05 | 0.88 | 1.07 | 0.57 | 1.01 | 0.05 | 0.92 | 1.10 | 0.90 | 0.99 | 0.05 | 0.89 | 1.09 | 0.81 |
| CRP 15 yrs (log) | 0.98 | 0.05 | 0.87 | 1.08 | 0.69 | 1.00 | 0.05 | 0.91 | 1.10 | 0.93 | 1.04 | 0.05 | 0.95 | 1.13 | 0.40 |
| CRP 9 yrs (log) | 1.00 | 0.05 | 0.91 | 1.09 | 0.95 | 1.04 | 0.04 | 0.95 | 1.13 | 0.41 | 1.00 | 0.04 | 0.91 | 1.09 | 1.00 |
| Average CRP 9-18 yrs (log) | 0.96 | 0.07 | 0.83 | 1.11 | 0.60 | 1.04 | 0.07 | 0.91 | 1.19 | 0.56 | 1.02 | 0.07 | 0.89 | 1.17 | 0.79 |
| ***CRP Trajectories*** |  |  |  |  |  |  |  |  |  |  |  |  |  |  |  |
| Low CRP Trajectory | ref |  |  |  |  |  |  |  |  |  | ref |  |  |  |  |
| Moderate-High CRP Trajectory | 0.95 | 0.11 | 0.74 | 1.16 | 0.63 | 1.19 | 0.12 | 0.95 | 1.49 | 0.13 | 0.97 | 0.11 | 0.76 | 1.18 | 0.78 |
| High-Moderate CRP Trajectory | 1.03 | 0.15 | 0.75 | 1.32 | 0.83 | 1.04 | 0.16 | 0.77 | 1.42 | 0.78 | 1.00 | 0.14 | 0.73 | 1.27 | 0.99 |
| **Note.** Sample: ALSPAC (N = 3,931). Pooled estimates from logistic regression models across 20 imputed models. CI = confidence interval. SE = standard error. ACEs = adverse childhood experiences. CRP = C-reactive protein. Model 1: adjusted for sex, ethnicity, maternal smoking during pregnancy, mother’s marital status, mother’s education, and household’s social class. P-values highlighted in bold are statistically significant at the 95% confidence level. | | | | | | | | | | | | | | | |

| **Table S29. Longitudinal mediation effects of CRP trajectories (9-18 yrs) in the association between ACEs (prenatal-18yrs) and depression trajectories (18-23 yrs).** | | | | | | | | | | | | | | | | |
| --- | --- | --- | --- | --- | --- | --- | --- | --- | --- | --- | --- | --- | --- | --- | --- | --- |
|  |  | **Mediation Effect (unexposed)** | | | | | **Mediation Effect (exposed)** | | | | | **Mediation Effect (average)** | | | | |
| **Exposure** | **Mediator** | **Estimate**  **(ACME)** | **Lower CI** | **Upper CI** | **p-value** | **Proportion mediated effect** | **Estimate**  **(ACME)** | **Lower CI** | **Upper CI** | **p-value** | **Proportion mediated effect** | **Estimate**  **(ACME)** | **Lower CI** | **Upper CI** | **p-value** | **Proportion mediated effect** |
| **Outcome: Moderate depression trajectory (vs low)** | | |  |  |  |  |  |  |  |  |  |  |  |  |  |  |
| ACEs multiple-exposure (2+ ACEs) | Moderate-High CRP Traj | 0.000 | -0.002 | 0.001 | 0.828 | -0.002 | 0.000 | -0.001 | 0.001 | 0.918 | 0.000 | 0.000 | -0.001 | 0.001 | 0.885 | -0.001 |
|  | High-moderate CRP Traj | 0.000 | -0.002 | 0.002 | 0.901 | -0.001 | 0.000 | -0.001 | 0.001 | 0.947 | 0.000 | 0.000 | -0.001 | 0.001 | 0.900 | -0.001 |
| Emotional/ Physical threat | Moderate-High CRP Traj | 0.000 | -0.001 | 0.001 | 0.515 | 0.004 | 0.000 | -0.001 | 0.001 | 0.705 | -0.002 | 0.000 | -0.000 | 0.001 | 0.793 | 0.001 |
|  | High-moderate CRP Traj | 0.001 | 0.000 | 0.001 | 0.244 | 0.008 | 0.001 | -0.001 | 0.002 | 0.272 | 0.010 | 0.001 | -0.000 | 0.002 | 0.179 | 0.009 |
| Household Dysfunction | Moderate-High CRP Traj | 0.000 | -0.001 | 0.001 | 0.849 | 0.002 | 0.000 | -0.001 | 0.001 | 0.973 | 0.000 | 0.000 | -0.001 | 0.001 | 0.861 | 0.001 |
|  | High-moderate CRP Traj | 0.000 | -0.001 | 0.001 | 0.991 | 0.000 | 0.000 | -0.001 | 0.001 | 0.986 | 0.000 | 0.000 | -0.001 | 0.001 | 0.985 | 0.000 |
| Physical abuse | Moderate-High CRP Traj | 0.000 | -0.001 | 0.001 | 0.658 | 0.003 | -0.001 | -0.004 | 0.003 | 0.693 | -0.009 | 0.000 | -0.002 | 0.001 | 0.773 | -0.003 |
|  | High-moderate CRP Traj | -0.001 | -0.003 | 0.001 | 0.346 | -0.011 | 0.003 | -0.003 | 0.009 | 0.349 | 0.029 | 0.001 | -0.001 | 0.003 | 0.441 | 0.009 |
| Emotional abuse/neglect | Moderate-High CRP Traj | 0.000 | -0.001 | 0.001 | 0.933 | 0.000 | 0.000 | -0.001 | 0.001 | 0.979 | 0.000 | 0.000 | -0.001 | 0.001 | 0.976 | 0.000 |
|  | High-moderate CRP Traj | 0.000 | -0.001 | 0.001 | 0.987 | 0.000 | 0.000 | -0.002 | 0.002 | 0.974 | 0.000 | 0.000 | -0.001 | 0.001 | 0.972 | 0.000 |
| Sexual abuse | Moderate-High CRP Traj | 0.000 | -0.002 | 0.001 | 0.660 | -0.002 | 0.002 | -0.007 | 0.012 | 0.653 | 0.010 | 0.001 | -0.004 | 0.006 | 0.714 | 0.004 |
|  | High-moderate CRP Traj | 0.000 | -0.002 | 0.003 | 0.707 | 0.002 | -0.002 | -0.014 | 0.009 | 0.712 | -0.010 | -0.001 | -0.006 | 0.004 | 0.731 | -0.004 |
| Bullying | Moderate-High CRP Traj | 0.000 | -0.001 | 0.001 | 0.928 | 0.000 | 0.000 | -0.001 | 0.001 | 0.963 | 0.000 | 0.000 | -0.001 | 0.001 | 0.932 | 0.000 |
|  | High-moderate CRP Traj | -0.001 | -0.003 | 0.001 | 0.215 | -0.010 | 0.000 | -0.003 | 0.003 | 0.782 | -0.003 | -0.001 | -0.003 | 0.001 | 0.339 | -0.007 |
| Household violence | Moderate-High CRP Traj | 0.000 | -0.001 | 0.001 | 0.945 | 0.001 | 0.000 | -0.002 | 0.002 | 0.976 | 0.001 | 0.000 | -0.001 | 0.001 | 0.954 | 0.001 |
|  | High-moderate CRP Traj | 0.000 | -0.001 | 0.001 | 0.779 | 0.003 | 0.001 | -0.002 | 0.003 | 0.666 | 0.012 | 0.000 | -0.001 | 0.002 | 0.646 | 0.007 |
| Parental Substance Use Problems | Moderate-High CRP Traj | 0.000 | -0.001 | 0.001 | 0.850 | 0.002 | 0.001 | -0.002 | 0.004 | 0.682 | 0.012 | 0.000 | -0.001 | 0.002 | 0.658 | 0.007 |
|  | High-moderate CRP Traj | -0.002 | -0.003 | 0.000 | 0.126 | -0.024 | 0.000 | -0.005 | 0.005 | 0.926 | 0.004 | -0.001 | -0.003 | 0.002 | 0.611 | -0.010 |
| Parental Mental Health Problems | Moderate-High CRP Traj | 0.000 | -0.001 | 0.001 | 0.693 | 0.002 | 0.000 | -0.001 | 0.001 | 0.882 | -0.001 | 0.000 | -0.001 | 0.001 | 0.865 | 0.001 |
|  | High-moderate CRP Traj | **0.001** | **0.000** | **0.002** | **0.045** | 0.012 | 0.000 | -0.001 | 0.001 | 0.664 | 0.003 | 0.001 | -0.000 | 0.002 | 0.137 | 0.007 |
| Parental Convictions | Moderate-High CRP Traj | 0.000 | -0.001 | 0.001 | 0.686 | -0.026 | 0.001 | -0.003 | 0.004 | 0.743 | 0.078 | 0.000 | -0.001 | 0.002 | 0.833 | 0.026 |
|  | High-moderate CRP Traj | 0.000 | -0.001 | 0.002 | 0.571 | -0.032 | 0.002 | -0.004 | 0.009 | 0.486 | -0.159 | 0.001 | -0.002 | 0.005 | 0.468 | -0.096 |
| Parental Separation | Moderate-High CRP Traj | 0.000 | -0.002 | 0.001 | 0.722 | 0.538 | 0.000 | -0.002 | 0.002 | 0.776 | 0.515 | 0.000 | -0.001 | 0.001 | 0.651 | 0.526 |
|  | High-moderate CRP Traj | 0.000 | -0.002 | 0.001 | 0.448 | -0.178 | 0.000 | -0.002 | 0.001 | 0.570 | -0.142 | 0.000 | -0.001 | 0.001 | 0.403 | -0.160 |
| Low Parent-Child Bonding | Moderate-High CRP Traj | 0.000 | -0.001 | 0.000 | 0.516 | -0.004 | 0.000 | -0.001 | 0.002 | 0.726 | 0.004 | 0.000 | -0.001 | 0.001 | 0.986 | 0.000 |
|  | High-moderate CRP Traj | 0.000 | -0.001 | 0.001 | 0.822 | 0.003 | 0.000 | -0.002 | 0.002 | 0.926 | 0.002 | 0.000 | -0.001 | 0.001 | 0.863 | 0.002 |
| **Outcome: Severe depression trajectory (vs low)** | | |  |  |  |  |  |  |  |  |  |  |  |  |  |  |
| ACEs multiple-exposure (2+ ACEs) | Moderate-High CRP Traj |  |  |  |  |  |  |  |  |  |  |  |  |  |  |  |
|  | High-moderate CRP Traj | -0.001 | -0.002 | 0.000 | 0.067 | -0.017 | 0.000 | -0.000 | 0.001 | 0.259 | 0.006 | 0.000 | -0.001 | 0.000 | 0.244 | -0.005 |
| Emotional/ Physical threat | Moderate-High CRP Traj | 0.000 | -0.001 | 0.001 | 0.616 | -0.003 | 0.000 | -0.001 | 0.002 | 0.654 | 0.003 | 0.000 | -0.001 | 0.001 | 0.965 | 0.000 |
|  | High-moderate CRP Traj | 0.000 | -0.001 | 0.001 | 0.866 | -0.001 | 0.000 | -0.001 | 0.001 | 0.608 | -0.002 | 0.000 | -0.001 | 0.000 | 0.569 | -0.001 |
| Household Dysfunction | Moderate-High CRP Traj | 0.000 | -0.001 | 0.001 | 0.624 | -0.003 | 0.000 | -0.001 | 0.001 | 0.703 | -0.002 | 0.000 | -0.001 | 0.001 | 0.587 | -0.003 |
|  | High-moderate CRP Traj | 0.001 | -0.001 | 0.002 | 0.413 | 0.014 | 0.000 | -0.001 | 0.001 | 0.654 | -0.004 | 0.000 | -0.001 | 0.001 | 0.544 | 0.005 |
| Physical abuse | Moderate-High CRP Traj | 0.000 | -0.001 | 0.001 | 0.930 | -0.001 | 0.000 | -0.001 | 0.001 | 0.389 | 0.007 | 0.000 | -0.001 | 0.001 | 0.616 | 0.003 |
|  | High-moderate CRP Traj | 0.000 | -0.001 | 0.001 | 0.818 | -0.001 | 0.000 | -0.004 | 0.005 | 0.961 | 0.001 | 0.000 | -0.002 | 0.002 | 0.996 | 0.000 |
| Emotional abuse/neglect | Moderate-High CRP Traj | 0.000 | -0.001 | 0.001 | 0.935 | 0.000 | 0.000 | -0.004 | 0.003 | 0.879 | -0.002 | 0.000 | -0.002 | 0.002 | 0.878 | -0.001 |
|  | High-moderate CRP Traj | 0.000 | -0.001 | 0.000 | 0.415 | -0.004 | 0.000 | -0.001 | 0.002 | 0.629 | 0.004 | 0.000 | -0.001 | 0.001 | 0.976 | 0.000 |
| Sexual abuse | Moderate-High CRP Traj | 0.000 | -0.001 | 0.001 | 0.836 | 0.001 | 0.000 | -0.001 | 0.002 | 0.890 | 0.001 | 0.000 | -0.001 | 0.001 | 0.839 | 0.001 |
|  | High-moderate CRP Traj | 0.000 | -0.001 | 0.001 | 0.562 | -0.002 | 0.003 | -0.005 | 0.011 | 0.489 | 0.014 | 0.001 | -0.003 | 0.005 | 0.521 | 0.006 |
| Bullying | Moderate-High CRP Traj | -0.001 | -0.003 | 0.001 | 0.180 | -0.006 | 0.003 | -0.017 | 0.023 | 0.799 | 0.012 | 0.001 | -0.009 | 0.011 | 0.906 | 0.003 |
|  | High-moderate CRP Traj | 0.000 | -0.001 | 0.001 | 0.934 | 0.000 | 0.000 | -0.002 | 0.002 | 0.848 | 0.002 | 0.000 | -0.001 | 0.001 | 0.878 | 0.001 |
| Household violence | Moderate-High CRP Traj | 0.001 | -0.001 | 0.002 | 0.408 | 0.008 | 0.000 | -0.002 | 0.002 | 0.936 | 0.001 | 0.000 | -0.001 | 0.002 | 0.601 | 0.005 |
|  | High-moderate CRP Traj | 0.000 | -0.001 | 0.001 | 0.695 | -0.003 | 0.000 | -0.001 | 0.002 | 0.713 | 0.006 | 0.000 | -0.001 | 0.001 | 0.883 | 0.001 |
| Parental Substance Use Problems | Moderate-High CRP Traj | 0.000 | -0.001 | 0.001 | 0.918 | -0.001 | 0.000 | -0.001 | 0.001 | 0.958 | -0.001 | 0.000 | -0.001 | 0.001 | 0.920 | -0.001 |
|  | High-moderate CRP Traj | -0.001 | -0.002 | 0.001 | 0.458 | -0.013 | 0.003 | -0.002 | 0.009 | 0.221 | 0.079 | 0.001 | -0.001 | 0.004 | 0.253 | 0.033 |
| Parental Mental Health Problems | Moderate-High CRP Traj | 0.000 | -0.001 | 0.001 | 0.816 | 0.002 | 0.000 | -0.003 | 0.004 | 0.877 | 0.005 | 0.000 | -0.002 | 0.002 | 0.833 | 0.003 |
|  | High-moderate CRP Traj | 0.000 | -0.001 | 0.001 | 0.692 | -0.004 | 0.000 | -0.001 | 0.001 | 0.747 | 0.003 | 0.000 | -0.001 | 0.001 | 0.934 | 0.000 |
| Parental Convictions | Moderate-High CRP Traj | 0.000 | -0.001 | 0.001 | 0.772 | -0.001 | 0.000 | -0.002 | 0.001 | 0.557 | -0.006 | 0.000 | -0.001 | 0.000 | 0.481 | -0.004 |
|  | High-moderate CRP Traj | 0.000 | -0.001 | 0.001 | 0.925 | -0.005 | 0.001 | -0.003 | 0.004 | 0.664 | 0.098 | 0.000 | -0.002 | 0.002 | 0.689 | 0.047 |
| Parental Separation | Moderate-High CRP Traj | 0.000 | -0.001 | 0.001 | 0.842 | -0.029 | -0.001 | -0.006 | 0.004 | 0.602 | -0.350 | -0.001 | -0.004 | 0.002 | 0.613 | -0.189 |
|  | High-moderate CRP Traj | 0.000 | -0.001 | 0.002 | 0.625 | 0.008 | -0.001 | -0.002 | 0.001 | 0.551 | -0.014 | 0.000 | -0.001 | 0.001 | 0.783 | -0.003 |
| Low Parent-Child Bonding | Moderate-High CRP Traj | 0.000 | -0.001 | 0.001 | 0.975 | 0.000 | 0.000 | -0.002 | 0.002 | 0.880 | 0.003 | 0.000 | -0.001 | 0.001 | 0.898 | 0.001 |
|  | High-moderate CRP Traj | 0.000 | -0.001 | 0.001 | 0.724 | -0.002 | 0.000 | -0.001 | 0.002 | 0.592 | 0.009 | 0.000 | -0.001 | 0.001 | 0.701 | 0.003 |
| **Note.** Sample: ALSPAC (N = 3,931). Pooled estimates from causal mediation analysis with 20 imputed datasets. The outcome and mediator models were estimated using logistic regression analysis. The estimation of the mediation effects in the unexposed and exposed groups allows the investigation of possible interactions between the exposure and mediator variables. Values in bold represent statistically significant coefficients at the 5% significance level (p<0.05). ACME = average causal mediation effect. | | | | | | | | | | | | | | | | |

| **Table S30. Associations of ACEs in the prenatal period with moderate-high and high-moderate CRP trajectories (vs low-moderate) ­– Complete data analysis.** | | | | | | | | | | | | | |
| --- | --- | --- | --- | --- | --- | --- | --- | --- | --- | --- | --- | --- | --- |
| **Outcome: CRP trajectories** |  | **Moderate-High Trajectory** | | | | | | **High-moderate Trajectory** | | | | | |
|  | **Model** | **OR** | **SE** | **Lower CI** | **Upper CI** | **p-value** | **p-value_fdr** | **OR** | **SE** | **Lower CI** | **Upper CI** | **p-value** | **p-value_fdr** |
| Total ACEs cumulative score | 1 | 0.83 | 0.15 | 0.55 | 1.11 | 0.20 | 0.65 | 1.00 | 0.19 | 0.62 | 1.38 | 0.99 | 0.99 |
| ***ACEs dimensions*** |  |  |  |  |  |  |  |  |  |  |  |  |  |
| Emotional/ Physical threat | 1 | 0.00 | 541.90 | -1062.13 | 1062.13 | 0.98 | 0.99 | 1.46 | 1.13 | -0.76 | 3.68 | 0.74 | 0.99 |
| Household Dysfunction | 1 | 0.75 | 0.17 | 0.42 | 1.09 | 0.10 | 0.35 | 0.93 | 0.23 | 0.49 | 1.38 | 0.76 | 0.99 |
| **Note.** Sample: ALSPAC (N = 1,242). Estimates from multinomial logistic regression models in the complete data sample. Reference outcome: Low-moderate CRP trajectory. CI = confidence interval. SE = standard error. fdr = false discovery rate (p-value). ACEs = adverse childhood experiences. CRP = C-reactive protein. Model 1: adjusted for sex, ethnicity, maternal smoking during pregnancy, mother’s marital status, mother’s education, and household’s social class; Model 2: Model 1 + earlier ACEs. P-values highlighted in bold are statistically significant at the 95% confidence level. | | | | | | | | | | | | | |

| **Table S31. Associations of ACEs in the prenatal period with moderate and severe depression trajectories (vs low) ­– Complete data analysis.** | | | | | | | | | | | | | |
| --- | --- | --- | --- | --- | --- | --- | --- | --- | --- | --- | --- | --- | --- |
| **Outcome: Depression trajectories** | | **Moderate Trajectory** | | | | | | **Severe Trajectory** | | | | | |
|  | **Model** | **OR** | **SE** | **Lower CI** | **Upper CI** | **p-value** | **p-value_fdr** | **OR** | **SE** | **Lower CI** | **Upper CI** | **p-value** | **p-value_fdr** |
|  |  |  |  |  |  |  |  |  |  |  |  |  |  |
| Total ACEs cumulative score | 1 | 1.64 | 0.13 | 1.38 | 1.89 | **0.00** | **0.00** | 1.82 | 0.19 | 1.44 | 2.20 | **0.00** | **0.01** |
| ***ACEs dimensions*** |  |  |  |  |  |  |  |  |  |  |  |  |  |
| Emotional/ Physical threat | 1 | 1.92 | 0.94 | 0.09 | 3.76 | 0.48 | 0.99 | 1.79 | 1.24 | -0.64 | 4.23 | 0.64 | 0.99 |
| Household Dysfunction | 1 | 1.75 | 0.15 | 1.45 | 2.05 | **0.00** | **0.00** | 2.34 | 0.24 | 1.88 | 2.81 | **0.00** | **0.00** |
| **Note.** Sample: ALSPAC (N = 1,242). Estimates from multinomial logistic regression models in the complete data sample. Reference outcome: Low depression trajectory. CI = confidence interval. SE = standard error. fdr = false discovery rate (p-value). ACEs = adverse childhood experiences. Model 1: adjusted for sex, ethnicity, maternal smoking during pregnancy, mother’s marital status, mother’s education, and household’s social class; Model 2: Model 1 + earlier ACEs. P-values highlighted in bold are statistically significant at the 95% confidence level. | | | | | | | | | | | | | |

| **Table S32. Associations of ACEs in early childhood (0-3 yrs) with moderate-high and high-moderate CRP trajectories (vs low-moderate) ­– Complete data analysis.** | | | | | | | | | | | | | |
| --- | --- | --- | --- | --- | --- | --- | --- | --- | --- | --- | --- | --- | --- |
| **Outcome: CRP trajectories** |  | **Moderate-High Trajectory** | | | | | | **High-moderate Trajectory** | | | | | |
|  | **Model** | **OR** | **SE** | **Lower CI** | **Upper CI** | **p-value** | **p-value_fdr** | **OR** | **SE** | **Lower CI** | **Upper CI** | **p-value** | **p-value_fdr** |
|  |  |  |  |  |  |  |  |  |  |  |  |  |  |
| Total ACEs cumulative score | 1 | 0.94 | 0.09 | 0.77 | 1.11 | 0.49 | 0.68 | 0.82 | 0.13 | 0.56 | 1.08 | 0.13 | 0.36 |
|  | 2 | 0.98 | 0.09 | 0.80 | 1.17 | 0.84 | 0.88 | 0.79 | 0.14 | 0.51 | 1.07 | 0.10 | 0.31 |
| ***ACEs dimensions*** |  |  |  |  |  |  |  |  |  |  |  |  |  |
| Emotional/ Physical threat | 1 | 0.71 | 0.24 | 0.23 | 1.18 | 0.15 | 0.39 | 0.78 | 0.33 | 0.14 | 1.42 | 0.44 | 0.63 |
|  | 2 | 0.73 | 0.24 | 0.26 | 1.21 | 0.20 | 0.46 | 0.77 | 0.33 | 0.12 | 1.42 | 0.43 | 0.63 |
| Household Dysfunction | 1 | 1.08 | 0.16 | 0.78 | 1.39 | 0.61 | 0.70 | 0.67 | 0.23 | 0.22 | 1.12 | 0.08 | 0.28 |
|  | 2 | 1.21 | 0.17 | 0.89 | 1.54 | 0.25 | 0.52 | 0.63 | 0.25 | 0.14 | 1.11 | 0.06 | 0.23 |
| ***Single adversities*** |  |  |  |  |  |  |  |  |  |  |  |  |  |
| Physical abuse | 1 | 0.56 | 0.55 | -0.52 | 1.63 | 0.29 | 0.52 | 0.56 | 0.75 | -0.91 | 2.03 | 0.44 | 0.63 |
|  | 2 | 0.58 | 0.55 | -0.50 | 1.65 | 0.32 | 0.56 | 0.56 | 0.75 | -0.92 | 2.03 | 0.44 | 0.63 |
| Emotional abuse/neglect | 1 | 0.75 | 0.29 | 0.18 | 1.31 | 0.31 | 0.56 | 0.81 | 0.40 | 0.03 | 1.59 | 0.59 | 0.70 |
|  | 2 | 0.78 | 0.29 | 0.21 | 1.35 | 0.39 | 0.61 | 0.79 | 0.40 | 0.00 | 1.58 | 0.57 | 0.69 |
| Household violence | 1 | 0.68 | 0.40 | -0.11 | 1.47 | 0.34 | 0.58 | 0.73 | 0.54 | -0.34 | 1.80 | 0.56 | 0.69 |
|  | 2 | 0.70 | 0.40 | -0.10 | 1.49 | 0.37 | 0.61 | 0.73 | 0.55 | -0.34 | 1.80 | 0.56 | 0.69 |
| Parental Substance Use Problems | 1 | 0.70 | 0.51 | -0.29 | 1.70 | 0.49 | 0.68 | 2.60 | 0.44 | 1.73 | 3.47 | **0.03** | 0.14 |
|  | 2 | 0.72 | 0.51 | -0.27 | 1.72 | 0.53 | 0.68 | 2.61 | 0.45 | 1.73 | 3.48 | **0.03** | 0.14 |
| Parental Mental Health Problems | 1 | 0.99 | 0.17 | 0.67 | 1.32 | 0.97 | 0.98 | 0.46 | 0.27 | -0.07 | 1.00 | **0.01** | **0.04** |
|  | 2 | 1.10 | 0.18 | 0.74 | 1.46 | 0.60 | 0.70 | 0.40 | 0.30 | -0.18 | 0.98 | **0.00** | **0.02** |
| Parental Convictions | 1 | 0.90 | 0.44 | 0.03 | 1.76 | 0.80 | 0.85 | 0.83 | 0.63 | -0.41 | 2.07 | 0.77 | 0.83 |
|  | 2 | 0.95 | 0.44 | 0.08 | 1.82 | 0.91 | 0.93 | 0.83 | 0.64 | -0.41 | 2.08 | 0.77 | 0.83 |
| Parental Separation | 1 | 1.48 | 0.29 | 0.90 | 2.05 | 0.18 | 0.43 | 1.22 | 0.42 | 0.40 | 2.05 | 0.63 | 0.70 |
|  | 2 | 1.59 | 0.30 | 1.00 | 2.17 | 0.12 | 0.34 | 1.23 | 0.43 | 0.40 | 2.06 | 0.63 | 0.70 |
| Low Parent-Child Bonding | 1 | 0.50 | 0.49 | -0.46 | 1.47 | 0.16 | 0.39 | 0.68 | 0.62 | -0.54 | 1.89 | 0.53 | 0.68 |
|  | 2 | 0.52 | 0.49 | -0.45 | 1.48 | 0.18 | 0.43 | 0.68 | 0.62 | -0.54 | 1.90 | 0.54 | 0.68 |
| **Note.** Sample: ALSPAC (N = 1,221). Estimates from multinomial logistic regression models in the complete data sample. Reference outcome: Low-moderate CRP trajectory. CI = confidence interval. SE = standard error. fdr = false discovery rate (p-value). ACEs = adverse childhood experiences. CRP = C-reactive protein. Model 1: adjusted for sex, ethnicity, maternal smoking during pregnancy, mother’s marital status, mother’s education, and household’s social class; Model 2: Model 1 + earlier ACEs. P-values highlighted in bold are statistically significant at the 95% confidence level. | | | | | | | | | | | | | |

| **Table S33. Associations of ACEs in early childhood (0-3 yrs) with moderate and severe depression trajectories (vs low) ­– Complete data analysis.** | | | | | | | | | | | | | |
| --- | --- | --- | --- | --- | --- | --- | --- | --- | --- | --- | --- | --- | --- |
| **Outcome: Depression trajectories** |  | **Moderate Trajectory** | | | | | | **Severe Trajectory** | | | | | |
|  | **Model** | **OR** | **SE** | **Lower CI** | **Upper CI** | **p-value** | **p-value_fdr** | **OR** | **SE** | **Lower CI** | **Upper CI** | **p-value** | **p-value_fdr** |
|  |  |  |  |  |  |  |  |  |  |  |  |  |  |
| Total ACEs cumulative score | 1 | 1.28 | 0.08 | 1.11 | 1.44 | **0.00** | **0.03** | 1.76 | 0.11 | 1.54 | 1.99 | **0.00** | **0.00** |
|  | 2 | 1.16 | 0.09 | 0.99 | 1.34 | 0.09 | 0.31 | 1.67 | 0.12 | 1.42 | 1.91 | **0.00** | **0.00** |
| ***ACEs dimensions*** |  |  |  |  |  |  |  |  |  |  |  |  |  |
| Emotional/ Physical threat | 1 | 1.23 | 0.22 | 0.81 | 1.65 | 0.34 | 0.58 | 2.34 | 0.29 | 1.77 | 2.91 | **0.00** | **0.03** |
|  | 2 | 1.11 | 0.22 | 0.68 | 1.54 | 0.62 | 0.70 | 2.10 | 0.30 | 1.51 | 2.68 | **0.01** | 0.09 |
| Household Dysfunction | 1 | 1.67 | 0.15 | 1.38 | 1.96 | **0.00** | **0.01** | 2.75 | 0.24 | 2.27 | 3.22 | **0.00** | **0.00** |
|  | 2 | 1.46 | 0.16 | 1.14 | 1.77 | **0.02** | 0.10 | 2.42 | 0.26 | 1.92 | 2.93 | **0.00** | **0.01** |
| ***Single adversities*** |  |  |  |  |  |  |  |  |  |  |  |  |  |
| Physical abuse | 1 | 0.77 | 0.52 | -0.24 | 1.78 | 0.61 | 0.70 | 3.66 | 0.52 | 2.64 | 4.68 | **0.01** | 0.09 |
|  | 2 | 0.71 | 0.52 | -0.31 | 1.73 | 0.51 | 0.68 | 3.28 | 0.53 | 2.24 | 4.32 | **0.03** | 0.12 |
| Emotional abuse/neglect | 1 | 1.32 | 0.26 | 0.82 | 1.83 | 0.28 | 0.52 | 2.08 | 0.35 | 1.39 | 2.78 | **0.04** | 0.15 |
|  | 2 | 1.19 | 0.26 | 0.67 | 1.70 | 0.52 | 0.68 | 1.74 | 0.36 | 1.02 | 2.45 | 0.13 | 0.36 |
| Household violence | 1 | 1.07 | 0.37 | 0.35 | 1.78 | 0.86 | 0.89 | 2.19 | 0.46 | 1.29 | 3.09 | 0.09 | 0.31 |
|  | 2 | 1.00 | 0.37 | 0.27 | 1.72 | 0.99 | 0.99 | 2.08 | 0.46 | 1.19 | 2.98 | 0.11 | 0.32 |
| Parental Substance Use Problems | 1 | 1.34 | 0.42 | 0.52 | 2.16 | 0.48 | 0.68 | 3.29 | 0.49 | 2.33 | 4.25 | **0.01** | 0.09 |
|  | 2 | 1.26 | 0.42 | 0.43 | 2.08 | 0.59 | 0.70 | 2.90 | 0.50 | 1.92 | 3.89 | **0.03** | 0.14 |
| Parental Mental Health Problems | 1 | 1.76 | 0.16 | 1.45 | 2.07 | **0.00** | **0.01** | 2.35 | 0.25 | 1.87 | 2.83 | **0.00** | **0.01** |
|  | 2 | 1.50 | 0.17 | 1.16 | 1.84 | **0.02** | 0.10 | 1.93 | 0.27 | 1.40 | 2.46 | **0.01** | 0.09 |
| Parental Convictions | 1 | 0.69 | 0.47 | -0.23 | 1.61 | 0.43 | 0.63 | 1.84 | 0.53 | 0.80 | 2.88 | 0.25 | 0.52 |
|  | 2 | 0.59 | 0.48 | -0.35 | 1.52 | 0.26 | 0.52 | 1.59 | 0.53 | 0.54 | 2.63 | 0.39 | 0.61 |
| Parental Separation | 1 | 1.60 | 0.29 | 1.04 | 2.17 | 0.10 | 0.31 | 1.97 | 0.41 | 1.17 | 2.77 | 0.10 | 0.31 |
|  | 2 | 1.41 | 0.29 | 0.83 | 1.98 | 0.25 | 0.52 | 1.60 | 0.43 | 0.76 | 2.43 | 0.27 | 0.52 |
| Low Parent-Child Bonding | 1 | 1.50 | 0.37 | 0.77 | 2.23 | 0.28 | 0.52 | 2.14 | 0.53 | 1.09 | 3.18 | 0.15 | 0.39 |
|  | 2 | 1.38 | 0.38 | 0.64 | 2.13 | 0.39 | 0.61 | 1.92 | 0.54 | 0.86 | 2.97 | 0.23 | 0.50 |
| **Note.** Sample: ALSPAC (N = 1,221). Estimates from multinomial logistic regression models in the complete data sample. Reference outcome: Low depression trajectory. CI = confidence interval. SE = standard error. fdr = false discovery rate (p-value). ACEs = adverse childhood experiences. Model 1: adjusted for sex, ethnicity, maternal smoking during pregnancy, mother’s marital status, mother’s education, and household’s social class; Model 2: Model 1 + earlier ACEs. P-values highlighted in bold are statistically significant at the 95% confidence level. | | | | | | | | | | | | | |

| **Table S34. Associations of ACEs in middle childhood (3-7 yrs) with moderate-high and high-moderate CRP trajectories (vs low-moderate) ­– Complete data analysis.** | | | | | | | | | | | | | |
| --- | --- | --- | --- | --- | --- | --- | --- | --- | --- | --- | --- | --- | --- |
| **Outcome: CRP trajectories** |  | **Moderate-High Trajectory** | | | | | | **High-moderate Trajectory** | | | | | |
|  | **Model** | **OR** | **SE** | **Lower CI** | **Upper CI** | **p-value** | **p-value_fdr** | **OR** | **SE** | **Lower CI** | **Upper CI** | **p-value** | **p-value_fdr** |
|  |  |  |  |  |  |  |  |  |  |  |  |  |  |
| Total ACEs cumulative score | 1 | 0.97 | 0.07 | 0.82 | 1.12 | 0.69 | 0.99 | 0.99 | 0.10 | 0.79 | 1.19 | 0.93 | 1.00 |
|  | 2 | 1.01 | 0.09 | 0.84 | 1.18 | 0.91 | 1.00 | 1.09 | 0.11 | 0.87 | 1.31 | 0.44 | 0.96 |
| ***ACEs dimensions*** |  |  |  |  |  |  |  |  |  |  |  |  |  |
| Emotional/ Physical threat | 1 | 0.93 | 0.19 | 0.56 | 1.31 | 0.72 | 1.00 | 1.23 | 0.25 | 0.73 | 1.73 | 0.41 | 0.96 |
|  | 2 | 0.98 | 0.20 | 0.58 | 1.38 | 0.93 | 1.00 | 1.55 | 0.27 | 1.02 | 2.08 | 0.10 | 0.63 |
| Household Dysfunction | 1 | 1.18 | 0.16 | 0.86 | 1.50 | 0.32 | 0.93 | 0.87 | 0.24 | 0.41 | 1.34 | 0.57 | 0.99 |
|  | 2 | 1.34 | 0.18 | 0.99 | 1.69 | 0.11 | 0.63 | 1.06 | 0.26 | 0.56 | 1.56 | 0.82 | 1.00 |
| ***Single adversities*** |  |  |  |  |  |  |  |  |  |  |  |  |  |
| Physical abuse | 1 | 0.27 | 0.61 | -0.92 | 1.46 | **0.03** | 0.42 | 1.23 | 0.46 | 0.33 | 2.14 | 0.65 | 0.99 |
|  | 2 | 0.29 | 0.62 | -0.92 | 1.50 | **0.04** | 0.42 | 1.57 | 0.48 | 0.63 | 2.51 | 0.35 | 0.96 |
| Emotional abuse/neglect | 1 | 0.80 | 0.26 | 0.29 | 1.31 | 0.38 | 0.96 | 1.01 | 0.35 | 0.33 | 1.69 | 0.97 | 1.00 |
|  | 2 | 0.88 | 0.28 | 0.33 | 1.42 | 0.64 | 0.99 | 1.32 | 0.37 | 0.60 | 2.04 | 0.45 | 0.96 |
| Household violence | 1 | 0.85 | 0.37 | 0.14 | 1.57 | 0.67 | 0.99 | 0.77 | 0.54 | -0.29 | 1.84 | 0.64 | 0.99 |
|  | 2 | 0.94 | 0.38 | 0.20 | 1.68 | 0.87 | 1.00 | 0.96 | 0.55 | -0.13 | 2.05 | 0.94 | 1.00 |
| Parental Substance Use Problems | 1 | 0.72 | 0.41 | -0.09 | 1.53 | 0.42 | 0.96 | 1.02 | 0.51 | 0.02 | 2.03 | 0.97 | 1.00 |
|  | 2 | 0.79 | 0.42 | -0.02 | 1.61 | 0.58 | 0.99 | 1.13 | 0.52 | 0.11 | 2.14 | 0.82 | 1.00 |
| Parental Mental Health Problems | 1 | 0.97 | 0.20 | 0.59 | 1.36 | 0.89 | 1.00 | 0.96 | 0.28 | 0.41 | 1.50 | 0.88 | 1.00 |
|  | 2 | 1.12 | 0.22 | 0.69 | 1.55 | 0.60 | 0.99 | 1.32 | 0.30 | 0.72 | 1.91 | 0.37 | 0.96 |
| Parental Convictions | 1 | 1.21 | 0.42 | 0.38 | 2.05 | 0.65 | 0.99 | 0.32 | 1.03 | -1.70 | 2.35 | 0.27 | 0.91 |
|  | 2 | 1.24 | 0.43 | 0.41 | 2.07 | 0.61 | 0.99 | 0.34 | 1.04 | -1.69 | 2.37 | 0.29 | 0.91 |
| Parental Separation | 1 | 1.62 | 0.24 | 1.16 | 2.08 | **0.04** | 0.42 | 1.27 | 0.34 | 0.61 | 1.94 | 0.48 | 0.96 |
|  | 2 | 1.76 | 0.24 | 1.28 | 2.23 | **0.02** | 0.42 | 1.43 | 0.35 | 0.75 | 2.11 | 0.30 | 0.91 |
| Low Parent-Child Bonding | 1 | 0.97 | 0.24 | 0.50 | 1.43 | 0.88 | 1.00 | 1.38 | 0.31 | 0.78 | 1.99 | 0.29 | 0.91 |
|  | 2 | 1.05 | 0.25 | 0.56 | 1.54 | 0.85 | 1.00 | 1.71 | 0.32 | 1.08 | 2.34 | 0.09 | 0.63 |
| **Note.** Sample: ALSPAC (N = 1,172). Estimates from multinomial logistic regression models in the complete data sample. Reference outcome: Low-moderate CRP trajectory. CI = confidence interval. SE = standard error. fdr = false discovery rate (p-value). ACEs = adverse childhood experiences. CRP = C-reactive protein. Model 1: adjusted for sex, ethnicity, maternal smoking during pregnancy, mother’s marital status, mother’s education, and household’s social class; Model 2: Model 1 + earlier ACEs. P-values highlighted in bold are statistically significant at the 95% confidence level. | | | | | | | | | | | | | |

| **Table S35. Associations of ACEs in middle childhood (3-7 yrs) with moderate and severe depression trajectories (vs low) ­– Complete data analysis.** | | | | | | | | | | | | | |
| --- | --- | --- | --- | --- | --- | --- | --- | --- | --- | --- | --- | --- | --- |
| **Outcome: Depression trajectories** | | **Moderate Trajectory** | | | | | | **Severe Trajectory** | | | | | |
|  | **Model** | **OR** | **SE** | **Lower CI** | **Upper CI** | **p-value** | **p-value_fdr** | **OR** | **SE** | **Lower CI** | **Upper CI** | **p-value** | **p-value_fdr** |
|  |  |  |  |  |  |  |  |  |  |  |  |  |  |
| Total ACEs cumulative score | 1 | 1.13 | 0.07 | 0.99 | 1.26 | 0.08 | 0.63 | 1.32 | 0.10 | 1.12 | 1.51 | **0.01** | 0.28 |
|  | 2 | 1.03 | 0.08 | 0.87 | 1.18 | 0.74 | 1.00 | 1.05 | 0.12 | 0.82 | 1.29 | 0.66 | 0.99 |
| ***ACEs dimensions*** |  |  |  |  |  |  |  |  |  |  |  |  |  |
| Emotional/ Physical threat | 1 | 1.35 | 0.18 | 1.01 | 1.70 | 0.09 | 0.63 | 2.19 | 0.27 | 1.66 | 2.72 | **0.00** | 0.28 |
|  | 2 | 1.18 | 0.19 | 0.82 | 1.55 | 0.37 | 0.96 | 1.59 | 0.30 | 1.01 | 2.17 | 0.12 | 0.67 |
| Household Dysfunction | 1 | 1.13 | 0.16 | 0.82 | 1.44 | 0.43 | 0.96 | 1.70 | 0.26 | 1.19 | 2.20 | **0.04** | 0.42 |
|  | 2 | 0.91 | 0.18 | 0.56 | 1.25 | 0.57 | 0.99 | 1.08 | 0.29 | 0.52 | 1.65 | 0.78 | 1.00 |
| ***Single adversities*** |  |  |  |  |  |  |  |  |  |  |  |  |  |
| Physical abuse | 1 | 0.99 | 0.38 | 0.25 | 1.74 | 0.99 | 1.00 | 1.75 | 0.52 | 0.73 | 2.77 | 0.28 | 0.91 |
|  | 2 | 0.77 | 0.39 | 0.00 | 1.54 | 0.51 | 0.97 | 1.00 | 0.55 | -0.08 | 2.08 | 1.00 | 1.00 |
| Emotional abuse/neglect | 1 | 1.31 | 0.23 | 0.85 | 1.76 | 0.25 | 0.91 | 2.09 | 0.35 | 1.41 | 2.77 | **0.03** | 0.42 |
|  | 2 | 1.04 | 0.25 | 0.54 | 1.53 | 0.88 | 1.00 | 1.20 | 0.39 | 0.43 | 1.97 | 0.64 | 0.99 |
| Household violence | 1 | 1.44 | 0.33 | 0.79 | 2.08 | 0.27 | 0.91 | 2.07 | 0.48 | 1.13 | 3.01 | 0.13 | 0.69 |
|  | 2 | 1.16 | 0.34 | 0.49 | 1.82 | 0.67 | 0.99 | 1.25 | 0.51 | 0.25 | 2.24 | 0.67 | 0.99 |
| Parental Substance Use Problems | 1 | 0.95 | 0.38 | 0.20 | 1.71 | 0.90 | 1.00 | 1.63 | 0.51 | 0.64 | 2.63 | 0.33 | 0.94 |
|  | 2 | 0.86 | 0.39 | 0.09 | 1.62 | 0.69 | 0.99 | 1.09 | 0.54 | 0.04 | 2.14 | 0.88 | 1.00 |
| Parental Mental Health Problems | 1 | 1.55 | 0.18 | 1.20 | 1.91 | **0.01** | 0.42 | 1.63 | 0.29 | 1.06 | 2.20 | 0.09 | 0.63 |
|  | 2 | 1.28 | 0.20 | 0.89 | 1.67 | 0.22 | 0.91 | 0.92 | 0.33 | 0.27 | 1.58 | 0.81 | 1.00 |
| Parental Convictions | 1 | 0.71 | 0.47 | -0.22 | 1.64 | 0.47 | 0.96 | 1.11 | 0.66 | -0.18 | 2.40 | 0.87 | 1.00 |
|  | 2 | 0.70 | 0.48 | -0.24 | 1.63 | 0.45 | 0.96 | 0.98 | 0.68 | -0.35 | 2.32 | 0.98 | 1.00 |
| Parental Separation | 1 | 0.89 | 0.25 | 0.40 | 1.37 | 0.64 | 0.99 | 1.28 | 0.36 | 0.56 | 1.99 | 0.50 | 0.97 |
|  | 2 | 0.77 | 0.26 | 0.27 | 1.27 | 0.30 | 0.91 | 0.95 | 0.38 | 0.21 | 1.70 | 0.90 | 1.00 |
| Low Parent-Child Bonding | 1 | 1.36 | 0.22 | 0.94 | 1.79 | 0.15 | 0.73 | 2.04 | 0.33 | 1.38 | 2.69 | **0.03** | 0.42 |
|  | 2 | 1.26 | 0.22 | 0.82 | 1.70 | 0.30 | 0.91 | 1.46 | 0.35 | 0.77 | 2.15 | 0.28 | 0.91 |
| **Note.** Sample: ALSPAC (N = 1,172). Estimates from multinomial logistic regression models in the complete data sample. Reference outcome: Low depression trajectory. CI = confidence interval. SE = standard error. fdr = false discovery rate (p-value). ACEs = adverse childhood experiences. Model 1: adjusted for sex, ethnicity, maternal smoking during pregnancy, mother’s marital status, mother’s education, and household’s social class; Model 2: Model 1 + earlier ACEs. P-values highlighted in bold are statistically significant at the 95% confidence level. | | | | | | | | | | | | | |

| **Table S36. Associations of ACEs in late childhood (7-12 yrs) with moderate-high and high-moderate CRP trajectories (vs low-moderate) ­– Complete data analysis.** | | | | | | | | | | | | | |
| --- | --- | --- | --- | --- | --- | --- | --- | --- | --- | --- | --- | --- | --- |
| **Outcome: CRP trajectories** |  | **Moderate-High Trajectory** | | | | | | **High-moderate Trajectory** | | | | | |
|  | **Model** | **OR** | **SE** | **Lower CI** | **Upper CI** | **p-value** | **p-value_fdr** | **OR** | **SE** | **Lower CI** | **Upper CI** | **p-value** | **p-value_fdr** |
|  |  |  |  |  |  |  |  |  |  |  |  |  |  |
| Total ACEs cumulative score | 1 | 1.03 | 0.08 | 0.87 | 1.19 | 0.74 | 0.96 | 1.14 | 0.10 | 0.94 | 1.34 | 0.20 | 0.69 |
|  | 2 | 1.05 | 0.09 | 0.87 | 1.23 | 0.58 | 0.96 | 1.19 | 0.12 | 0.97 | 1.42 | 0.13 | 0.60 |
| ***ACEs dimensions*** |  |  |  |  |  |  |  |  |  |  |  |  |  |
| Emotional/ Physical threat | 1 | 1.06 | 0.19 | 0.69 | 1.43 | 0.77 | 0.96 | 1.35 | 0.24 | 0.87 | 1.83 | 0.22 | 0.69 |
|  | 2 | 1.10 | 0.21 | 0.69 | 1.50 | 0.66 | 0.96 | 1.43 | 0.27 | 0.91 | 1.96 | 0.18 | 0.69 |
| Household Dysfunction | 1 | 1.13 | 0.19 | 0.76 | 1.50 | 0.52 | 0.96 | 0.69 | 0.29 | 0.13 | 1.26 | 0.20 | 0.69 |
|  | 2 | 1.25 | 0.20 | 0.86 | 1.63 | 0.26 | 0.81 | 0.74 | 0.30 | 0.16 | 1.33 | 0.31 | 0.92 |
| ***Single adversities*** |  |  |  |  |  |  |  |  |  |  |  |  |  |
| Physical abuse | 1 | 0.00 | 753.18 | -1476.23 | 1476.23 | 0.98 | 0.99 | 1.31 | 0.80 | -0.26 | 2.87 | 0.74 | 0.96 |
|  | 2 | 0.00 | 524.53 | -1028.08 | 1028.08 | 0.98 | 0.99 | 1.24 | 0.85 | -0.43 | 2.91 | 0.80 | 0.96 |
| Emotional abuse/neglect | 1 | 0.92 | 0.29 | 0.35 | 1.49 | 0.77 | 0.96 | 1.18 | 0.36 | 0.46 | 1.89 | 0.66 | 0.96 |
|  | 2 | 1.01 | 0.31 | 0.41 | 1.61 | 0.97 | 0.99 | 1.25 | 0.39 | 0.50 | 2.01 | 0.56 | 0.96 |
| Bullying | 1 | 1.06 | 0.22 | 0.63 | 1.49 | 0.80 | 0.96 | 1.69 | 0.26 | 1.18 | 2.21 | **0.04** | 0.35 |
|  | 2 | 1.08 | 0.23 | 0.63 | 1.52 | 0.75 | 0.96 | 1.85 | 0.27 | 1.32 | 2.37 | **0.02** | 0.30 |
| Household violence | 1 | 0.98 | 0.36 | 0.27 | 1.69 | 0.96 | 0.99 | 0.70 | 0.55 | -0.37 | 1.77 | 0.51 | 0.96 |
|  | 2 | 1.11 | 0.38 | 0.36 | 1.85 | 0.79 | 0.96 | 0.74 | 0.57 | -0.39 | 1.86 | 0.60 | 0.96 |
| Parental Substance Use Problems | 1 | 0.12 | 1.03 | -1.90 | 2.14 | **0.04** | 0.35 | 0.51 | 0.77 | -1.00 | 2.02 | 0.38 | 0.95 |
|  | 2 | 0.14 | 1.04 | -1.89 | 2.17 | 0.06 | 0.41 | 0.77 | 0.80 | -0.79 | 2.32 | 0.74 | 0.96 |
| Parental Mental Health Problems | 1 | 1.22 | 0.26 | 0.71 | 1.74 | 0.44 | 0.96 | 1.03 | 0.39 | 0.26 | 1.81 | 0.93 | 0.99 |
|  | 2 | 1.42 | 0.27 | 0.88 | 1.96 | 0.20 | 0.69 | 1.21 | 0.41 | 0.41 | 2.01 | 0.64 | 0.96 |
| Parental Convictions | 1 | 0.95 | 0.53 | -0.09 | 1.98 | 0.92 | 0.99 | 0.00 | 455.97 | -893.71 | 893.71 | 0.98 | 0.99 |
|  | 2 | 0.99 | 0.53 | -0.06 | 2.03 | 0.98 | 0.99 | 0.00 | 573.58 | -1124.23 | 1124.23 | 0.98 | 0.99 |
| Parental Separation | 1 | 1.50 | 0.28 | 0.95 | 2.05 | 0.15 | 0.67 | 1.20 | 0.41 | 0.40 | 2.01 | 0.65 | 0.96 |
|  | 2 | 1.65 | 0.29 | 1.10 | 2.21 | 0.08 | 0.41 | 1.26 | 0.42 | 0.43 | 2.08 | 0.59 | 0.96 |
| Low Parent-Child Bonding | 1 | 1.10 | 0.22 | 0.67 | 1.53 | 0.67 | 0.96 | 1.66 | 0.27 | 1.13 | 2.20 | 0.06 | 0.41 |
|  | 2 | 1.19 | 0.24 | 0.72 | 1.66 | 0.47 | 0.96 | 1.90 | 0.30 | 1.31 | 2.49 | **0.03** | 0.35 |
| **Note.** Sample: ALSPAC (N = 1,188). Estimates from multinomial logistic regression models in the complete data sample. Reference outcome: Low-moderate CRP trajectory. CI = confidence interval. SE = standard error. fdr = false discovery rate (p-value). ACEs = adverse childhood experiences. CRP = C-reactive protein. Model 1: adjusted for sex, ethnicity, maternal smoking during pregnancy, mother’s marital status, mother’s education, and household’s social class; Model 2: Model 1 + earlier ACEs. P-values highlighted in bold are statistically significant at the 95% confidence level. | | | | | | | | | | | | | |
|  | | | | | | | | | | | | | |

| **Table S37. Associations of ACEs in late childhood (7-12 yrs) with moderate and severe depression trajectories (vs low) ­– Complete data analysis.** | | | | | | | | | | | | | | | |
| --- | --- | --- | --- | --- | --- | --- | --- | --- | --- | --- | --- | --- | --- | --- | --- |
| **Outcome: Depression trajectories** | | | | **Moderate Trajectory** | | | | | | **Severe Trajectory** | | | | | |
|  | **Model** | **OR** | **SE** | | **Lower CI** | **Upper CI** | **p-value** | **p-value_fdr** | **OR** | | **SE** | **Lower CI** | **Upper CI** | **p-value** | **p-value_fdr** |
|  |  |  |  | |  |  |  |  |  | |  |  |  |  |  |
| Total ACEs cumulative score | 1 | 1.17 | 0.08 | | 1.03 | 1.32 | **0.03** | 0.35 | 1.31 | | 0.11 | 1.09 | 1.54 | **0.02** | 0.28 |
|  | 2 | 1.12 | 0.08 | | 0.95 | 1.28 | 0.19 | 0.69 | 1.14 | | 0.13 | 0.88 | 1.40 | 0.33 | 0.92 |
| ***ACEs dimensions*** |  |  |  | |  |  |  |  |  | |  |  |  |  |  |
| Emotional/ Physical threat | 1 | 1.11 | 0.18 | | 0.75 | 1.46 | 0.58 | 0.96 | 2.17 | | 0.26 | 1.65 | 2.69 | **0.00** | 0.09 |
|  | 2 | 1.00 | 0.20 | | 0.61 | 1.39 | 0.99 | 0.99 | 1.83 | | 0.30 | 1.25 | 2.42 | **0.04** | 0.35 |
| Household Dysfunction | 1 | 1.38 | 0.18 | | 1.03 | 1.73 | 0.07 | 0.41 | 1.22 | | 0.29 | 0.65 | 1.79 | 0.50 | 0.96 |
|  | 2 | 1.20 | 0.19 | | 0.83 | 1.57 | 0.34 | 0.94 | 0.90 | | 0.32 | 0.27 | 1.52 | 0.73 | 0.96 |
| ***Single adversities*** |  |  |  | |  |  |  |  |  | |  |  |  |  |  |
| Physical abuse | 1 | 1.61 | 0.65 | | 0.33 | 2.88 | 0.47 | 0.96 | 1.41 | | 1.14 | -0.82 | 3.63 | 0.76 | 0.96 |
|  | 2 | 1.19 | 0.67 | | -0.13 | 2.50 | 0.80 | 0.96 | 0.76 | | 1.19 | -1.57 | 3.09 | 0.82 | 0.96 |
| Emotional abuse/neglect | 1 | 1.08 | 0.27 | | 0.55 | 1.60 | 0.78 | 0.96 | 1.22 | | 0.41 | 0.42 | 2.02 | 0.63 | 0.96 |
|  | 2 | 0.93 | 0.29 | | 0.36 | 1.49 | 0.79 | 0.96 | 0.77 | | 0.46 | -0.14 | 1.68 | 0.57 | 0.96 |
| Bullying | 1 | 1.44 | 0.20 | | 1.04 | 1.83 | 0.07 | 0.41 | 1.72 | | 0.30 | 1.13 | 2.31 | 0.07 | 0.41 |
|  | 2 | 1.38 | 0.21 | | 0.97 | 1.78 | 0.12 | 0.60 | 1.56 | | 0.32 | 0.94 | 2.18 | 0.16 | 0.67 |
| Household violence | 1 | 1.60 | 0.33 | | 0.95 | 2.25 | 0.15 | 0.67 | 1.04 | | 0.57 | -0.08 | 2.16 | 0.95 | 0.99 |
|  | 2 | 1.28 | 0.35 | | 0.59 | 1.97 | 0.48 | 0.96 | 0.57 | | 0.63 | -0.67 | 1.81 | 0.38 | 0.95 |
| Parental Substance Use Problems | 1 | 1.09 | 0.49 | | 0.13 | 2.05 | 0.86 | 0.98 | 2.14 | | 0.61 | 0.94 | 3.35 | 0.21 | 0.69 |
|  | 2 | 0.70 | 0.54 | | -0.35 | 1.76 | 0.51 | 0.96 | 1.13 | | 0.66 | -0.16 | 2.43 | 0.85 | 0.98 |
| Parental Mental Health Problems | 1 | 2.05 | 0.25 | | 1.57 | 2.53 | **0.00** | 0.09 | 1.33 | | 0.43 | 0.49 | 2.17 | 0.51 | 0.96 |
|  | 2 | 2.09 | 0.26 | | 1.59 | 2.60 | **0.00** | 0.09 | 0.93 | | 0.47 | 0.00 | 1.85 | 0.87 | 0.99 |
| Parental Convictions | 1 | 1.57 | 0.49 | | 0.60 | 2.54 | 0.36 | 0.94 | 1.49 | | 0.80 | -0.08 | 3.06 | 0.62 | 0.96 |
|  | 2 | 1.49 | 0.50 | | 0.51 | 2.47 | 0.42 | 0.96 | 1.21 | | 0.81 | -0.38 | 2.81 | 0.81 | 0.96 |
| Parental Separation | 1 | 0.72 | 0.31 | | 0.11 | 1.33 | 0.29 | 0.86 | 0.64 | | 0.55 | -0.43 | 1.72 | 0.42 | 0.96 |
|  | 2 | 0.75 | 0.32 | | 0.13 | 1.37 | 0.36 | 0.94 | 0.64 | | 0.57 | -0.48 | 1.75 | 0.42 | 0.96 |
| Low Parent-Child Bonding | 1 | 1.10 | 0.21 | | 0.69 | 1.52 | 0.64 | 0.96 | 2.89 | | 0.30 | 2.30 | 3.48 | **0.00** | **0.04** |
|  | 2 | 1.08 | 0.23 | | 0.63 | 1.53 | 0.73 | 0.96 | 2.60 | | 0.34 | 1.94 | 3.26 | **0.00** | 0.09 |
| **Note.** Sample: ALSPAC (N = 1,188). Estimates from multinomial logistic regression models in the complete data sample. Reference outcome: Low depression trajectory. CI = confidence interval. SE = standard error. fdr = false discovery rate (p-value). ACEs = adverse childhood experiences. Model 1: adjusted for sex, ethnicity, maternal smoking during pregnancy, mother’s marital status, mother’s education, and household’s social class; Model 2: Model 1 + earlier ACEs. P-values highlighted in bold are statistically significant at the 95% confidence level. | | | | | | | | | | | | | | | |

| **Table S38. Associations of ACEs in adolescence (12-18 yrs) with moderate-high and high-moderate CRP trajectories (vs low-moderate) ­– Complete data analysis.** | | | | | | | | | | | | | |
| --- | --- | --- | --- | --- | --- | --- | --- | --- | --- | --- | --- | --- | --- |
| **Outcome: CRP trajectories** | | **Moderate-High Trajectory** | | | | |  | **High-moderate Trajectory** | | | | | |
|  | **Model** | **OR** | **SE** | **Lower CI** | **Upper CI** | **p-value** | **p-value_fdr** | **OR** | **SE** | **Lower CI** | **Upper CI** | **p-value** | **p-value_fdr** |
|  |  |  |  |  |  |  |  |  |  |  |  |  |  |
| Total ACEs cumulative score | 1 | 1.05 | 0.08 | 0.90 | 1.21 | 0.50 | 0.76 | 1.32 | 0.10 | 1.13 | 1.51 | **0.00** | **0.03** |
|  | 2 | 1.04 | 0.08 | 0.88 | 1.21 | 0.61 | 0.84 | 1.29 | 0.10 | 1.09 | 1.50 | **0.01** | 0.05 |
| ***ACEs dimensions*** |  |  |  |  |  |  |  |  |  |  |  |  |  |
| Emotional/ Physical threat | 1 | 0.92 | 0.16 | 0.60 | 1.23 | 0.59 | 0.84 | 1.28 | 0.21 | 0.87 | 1.69 | 0.24 | 0.50 |
|  | 2 | 0.93 | 0.17 | 0.60 | 1.26 | 0.65 | 0.84 | 1.23 | 0.22 | 0.80 | 1.66 | 0.35 | 0.64 |
| Household Dysfunction | 1 | 1.20 | 0.19 | 0.82 | 1.58 | 0.34 | 0.63 | 1.11 | 0.27 | 0.59 | 1.64 | 0.68 | 0.84 |
|  | 2 | 1.23 | 0.20 | 0.83 | 1.63 | 0.30 | 0.60 | 0.97 | 0.28 | 0.41 | 1.52 | 0.90 | 0.99 |
| ***Single adversities*** |  |  |  |  |  |  |  |  |  |  |  |  |  |
| Physical abuse | 1 | 1.10 | 0.29 | 0.52 | 1.67 | 0.76 | 0.90 | 2.35 | 0.32 | 1.71 | 2.98 | **0.01** | **0.04** |
|  | 2 | 1.14 | 0.31 | 0.53 | 1.76 | 0.67 | 0.84 | 2.29 | 0.35 | 1.60 | 2.98 | **0.02** | 0.06 |
| Emotional abuse/neglect | 1 | 0.88 | 0.19 | 0.51 | 1.25 | 0.49 | 0.76 | 1.26 | 0.23 | 0.81 | 1.72 | 0.32 | 0.61 |
|  | 2 | 0.87 | 0.19 | 0.49 | 1.25 | 0.48 | 0.76 | 1.24 | 0.24 | 0.77 | 1.71 | 0.37 | 0.67 |
| Sexual abuse | 1 | 0.77 | 0.38 | 0.02 | 1.51 | 0.48 | 0.76 | 1.59 | 0.43 | 0.75 | 2.43 | 0.28 | 0.57 |
|  | 2 | 0.74 | 0.40 | -0.05 | 1.53 | 0.45 | 0.76 | 1.84 | 0.44 | 0.97 | 2.70 | 0.17 | 0.38 |
| Bullying | 1 | 1.17 | 0.20 | 0.77 | 1.57 | 0.44 | 0.76 | 1.91 | 0.25 | 1.42 | 2.39 | **0.01** | **0.04** |
|  | 2 | 1.10 | 0.22 | 0.68 | 1.52 | 0.66 | 0.84 | 2.07 | 0.25 | 1.57 | 2.56 | **0.00** | **0.03** |
| Parental Substance Use Problems | 1 | 1.38 | 0.61 | 0.17 | 2.58 | 0.60 | 0.84 | 1.71 | 0.80 | 0.14 | 3.28 | 0.50 | 0.76 |
|  | 2 | 1.37 | 0.62 | 0.16 | 2.58 | 0.61 | 0.84 | 1.55 | 0.81 | -0.03 | 3.13 | 0.59 | 0.84 |
| Parental Mental Health Problems | 1 | 1.53 | 0.63 | 0.29 | 2.76 | 0.50 | 0.76 | 0.83 | 1.10 | -1.33 | 3.00 | 0.87 | 0.98 |
|  | 2 | 1.63 | 0.67 | 0.32 | 2.94 | 0.47 | 0.76 | 0.81 | 1.13 | -1.40 | 3.02 | 0.85 | 0.98 |
| Parental Separation | 1 | 1.32 | 0.25 | 0.83 | 1.80 | 0.26 | 0.55 | 0.88 | 0.39 | 0.12 | 1.65 | 0.75 | 0.90 |
|  | 2 | 1.42 | 0.26 | 0.92 | 1.92 | 0.17 | 0.38 | 0.85 | 0.40 | 0.06 | 1.63 | 0.68 | 0.84 |
| Low Parent-Child Bonding | 1 | 0.97 | 0.25 | 0.47 | 1.47 | 0.91 | 0.99 | 1.19 | 0.33 | 0.55 | 1.82 | 0.60 | 0.84 |
|  | 2 | 1.02 | 0.26 | 0.50 | 1.53 | 0.95 | 0.99 | 1.14 | 0.34 | 0.48 | 1.80 | 0.69 | 0.84 |
| **Note.** Sample: ALSPAC (N = 1,220). Estimates from multinomial logistic regression models in the complete data sample. Reference outcome: Low-moderate CRP trajectory. CI = confidence interval. SE = standard error. fdr = false discovery rate (p-value). ACEs = adverse childhood experiences. CRP = C-reactive protein. Model 1: adjusted for sex, ethnicity, maternal smoking during pregnancy, mother’s marital status, mother’s education, and household’s social class; Model 2: Model 1 + earlier ACEs. P-values highlighted in bold are statistically significant at the 95% confidence level. | | | | | | | | | | | | | |

| **Table S39. Associations of ACEs in adolescence (12-18 yrs) with moderate and severe depression trajectories (vs low) ­– Complete data analysis.** | | | | | | | | | | | | | |
| --- | --- | --- | --- | --- | --- | --- | --- | --- | --- | --- | --- | --- | --- |
| **Outcome: Depression trajectories** | | **Moderate Trajectory** | | | | | | **Severe Trajectory** | | | | | |
|  | **Model** | **OR** | **SE** | **Lower CI** | **Upper CI** | **p-value** | **p-value_fdr** | **OR** | **SE** | **Lower CI** | **Upper CI** | **p-value** | **p-value_fdr** |
|  |  |  |  |  |  |  |  |  |  |  |  |  |  |
| Total ACEs cumulative score | 1 | 1.49 | 0.07 | 1.35 | 1.64 | **0.00** | **0.00** | 2.08 | 0.11 | 1.87 | 2.29 | **0.00** | **0.00** |
|  | 2 | 1.44 | 0.08 | 1.28 | 1.60 | **0.00** | **0.00** | 2.11 | 0.12 | 1.87 | 2.34 | **0.00** | **0.00** |
| ***ACEs dimensions*** |  |  |  |  |  |  |  |  |  |  |  |  |  |
| Emotional/ Physical threat | 1 | 1.51 | 0.15 | 1.22 | 1.80 | **0.01** | **0.03** | 2.71 | 0.24 | 2.25 | 3.17 | **0.00** | **0.00** |
|  | 2 | 1.49 | 0.16 | 1.18 | 1.79 | **0.01** | 0.05 | 2.78 | 0.25 | 2.28 | 3.28 | **0.00** | **0.00** |
| Household Dysfunction | 1 | 1.35 | 0.18 | 0.99 | 1.72 | 0.10 | 0.26 | 1.93 | 0.27 | 1.40 | 2.46 | **0.02** | 0.06 |
|  | 2 | 1.16 | 0.20 | 0.77 | 1.55 | 0.45 | 0.76 | 1.57 | 0.30 | 0.99 | 2.15 | 0.13 | 0.31 |
| ***Single adversities*** |  |  |  |  |  |  |  |  |  |  |  |  |  |
| Physical abuse | 1 | 2.04 | 0.27 | 1.51 | 2.57 | **0.01** | **0.04** | 6.06 | 0.34 | 5.40 | 6.73 | **0.00** | **0.00** |
|  | 2 | 1.86 | 0.29 | 1.29 | 2.44 | **0.03** | 0.11 | 6.99 | 0.37 | 6.26 | 7.71 | **0.00** | **0.00** |
| Emotional abuse/neglect | 1 | 1.49 | 0.17 | 1.16 | 1.82 | **0.02** | 0.06 | 1.97 | 0.26 | 1.46 | 2.47 | **0.01** | **0.04** |
|  | 2 | 1.35 | 0.18 | 1.01 | 1.70 | 0.09 | 0.24 | 1.93 | 0.27 | 1.40 | 2.47 | **0.02** | 0.06 |
| Sexual abuse | 1 | 4.41 | 0.38 | 3.66 | 5.16 | **0.00** | **0.00** | 14.48 | 0.43 | 13.64 | 15.32 | **0.00** | **0.00** |
|  | 2 | 4.22 | 0.41 | 3.42 | 5.02 | **0.00** | **0.00** | 15.26 | 0.45 | 14.37 | 16.15 | **0.00** | **0.00** |
| Bullying | 1 | 1.90 | 0.18 | 1.54 | 2.26 | **0.00** | **0.00** | 1.86 | 0.31 | 1.26 | 2.46 | **0.04** | 0.13 |
|  | 2 | 1.81 | 0.19 | 1.44 | 2.19 | **0.00** | **0.01** | 1.67 | 0.33 | 1.03 | 2.31 | 0.12 | 0.29 |
| Parental Substance Use Problems | 1 | 2.86 | 0.59 | 1.69 | 4.02 | 0.08 | 0.22 | 4.07 | 0.76 | 2.58 | 5.56 | 0.06 | 0.19 |
|  | 2 | 2.86 | 0.60 | 1.68 | 4.04 | 0.08 | 0.23 | 4.29 | 0.77 | 2.77 | 5.81 | 0.06 | 0.18 |
| Parental Mental Health Problems | 1 | 0.83 | 0.68 | -0.51 | 2.17 | 0.79 | 0.93 | 0.00 | 543.49 | -1065.24 | 1065.24 | 0.98 | 0.99 |
|  | 2 | 0.70 | 0.73 | -0.73 | 2.13 | 0.63 | 0.84 | 0.00 | 787.05 | -1542.63 | 1542.63 | 0.99 | 0.99 |
| Parental Separation | 1 | 1.07 | 0.25 | 0.57 | 1.56 | 0.80 | 0.93 | 1.42 | 0.36 | 0.72 | 2.12 | 0.33 | 0.61 |
|  | 2 | 0.90 | 0.27 | 0.37 | 1.42 | 0.68 | 0.84 | 1.26 | 0.37 | 0.53 | 1.99 | 0.54 | 0.80 |
| Low Parent-Child Bonding | 1 | 1.36 | 0.24 | 0.90 | 1.82 | 0.19 | 0.41 | 2.66 | 0.32 | 2.03 | 3.29 | **0.00** | **0.02** |
|  | 2 | 1.20 | 0.25 | 0.70 | 1.69 | 0.48 | 0.76 | 2.63 | 0.34 | 1.96 | 3.30 | **0.00** | **0.03** |
| **Note.** Sample: ALSPAC (N = 1,220). Estimates from multinomial logistic regression models in the complete data sample. Reference outcome: Low depression trajectory. CI = confidence interval. SE = standard error. fdr = false discovery rate (p-value). ACEs = adverse childhood experiences. Model 1: adjusted for sex, ethnicity, maternal smoking during pregnancy, mother’s marital status, mother’s education, and household’s social class; Model 2: Model 1 + earlier ACEs. P-values highlighted in bold are statistically significant at the 95% confidence level. | | | | | | | | | | | | | |

| **Table S40. Associations of any ACEs exposure throughout childhood (prenatal-18yrs) with moderate-high and high-moderate CRP trajectories (vs low-moderate) ­– Complete data analysis.** | | | | | | | | | | | | | | |
| --- | --- | --- | --- | --- | --- | --- | --- | --- | --- | --- | --- | --- | --- | --- |
| **Outcome: CRP trajectories** |  | **Moderate-High Trajectory** | | | | | | | **High-moderate Trajectory** | | | | | |
|  | **Model** | | **OR** | **SE** | **Lower CI** | **Upper CI** | **p-value** | **p-value_fdr** | **OR** | **SE** | **Lower CI** | **Upper CI** | **p-value** | **p-value_fdr** |
|  |  | |  |  |  |  |  |  |  |  |  |  |  |  |
| Total ACEs cumulative score | 1 | | 1.03 | 0.04 | 0.95 | 1.12 | 0.45 | 0.61 | 1.09 | 0.06 | 0.98 | 1.21 | 0.14 | 0.29 |
| ***ACEs dimensions*** |  | |  |  |  |  |  |  |  |  |  |  |  |  |
| Emotional/ Physical threat | 1 | | 1.02 | 0.15 | 0.73 | 1.31 | 0.90 | 0.96 | 1.34 | 0.20 | 0.95 | 1.74 | 0.14 | 0.29 |
| Household Dysfunction | 1 | | 1.11 | 0.16 | 0.80 | 1.41 | 0.52 | 0.65 | 0.90 | 0.21 | 0.48 | 1.31 | 0.60 | 0.71 |
| ***Single adversities*** |  | |  |  |  |  |  |  |  |  |  |  |  |  |
| Physical abuse | 1 | | 0.79 | 0.25 | 0.30 | 1.29 | 0.35 | 0.54 | 1.79 | 0.27 | 1.25 | 2.33 | **0.03** | 0.09 |
| Emotional abuse/neglect | 1 | | 0.91 | 0.16 | 0.60 | 1.21 | 0.53 | 0.65 | 1.16 | 0.21 | 0.76 | 1.57 | 0.46 | 0.62 |
| Sexual abuse | 1 | | 0.63 | 0.37 | -0.09 | 1.35 | 0.21 | 0.38 | 1.17 | 0.41 | 0.37 | 1.98 | 0.70 | 0.77 |
| Bullying | 1 | | 1.15 | 0.17 | 0.82 | 1.48 | 0.41 | 0.59 | 1.90 | 0.21 | 1.48 | 2.31 | **0.00** | **0.01** |
| Household violence | 1 | | 1.09 | 0.19 | 0.73 | 1.46 | 0.63 | 0.73 | 1.22 | 0.25 | 0.73 | 1.72 | 0.42 | 0.59 |
| Parental Substance Use Problems | 1 | | 0.79 | 0.27 | 0.26 | 1.32 | 0.39 | 0.58 | 1.19 | 0.33 | 0.54 | 1.84 | 0.59 | 0.71 |
| Parental Mental Health Problems | 1 | | 1.19 | 0.16 | 0.89 | 1.50 | 0.26 | 0.44 | 0.81 | 0.21 | 0.40 | 1.23 | 0.33 | 0.52 |
| Parental Convictions | 1 | | 1.01 | 0.28 | 0.47 | 1.56 | 0.97 | 0.97 | 0.55 | 0.48 | -0.40 | 1.49 | 0.21 | 0.38 |
| Parental Separation | 1 | | 1.32 | 0.17 | 0.99 | 1.65 | 0.10 | 0.23 | 0.99 | 0.24 | 0.52 | 1.46 | 0.97 | 0.97 |
| Low Parent-Child Bonding | 1 | | 1.06 | 0.18 | 0.70 | 1.42 | 0.75 | 0.81 | 1.11 | 0.25 | 0.62 | 1.60 | 0.69 | 0.77 |
| **Note.** Sample: ALSPAC (N = 1,224). Estimates from multinomial logistic regression models in the complete data sample. Reference outcome: Low-moderate CRP trajectory. CI = confidence interval. SE = standard error. fdr = false discovery rate (p-value). ACEs = adverse childhood experiences. CRP = C-reactive protein. Model 1: adjusted for sex, ethnicity, maternal smoking during pregnancy, mother’s marital status, mother’s education, and household’s social class; Model 2: Model 1 + earlier ACEs. P-values highlighted in bold are statistically significant at the 95% confidence level. | | | | | | | | | | | | | | |

| **Table S41. Associations of any ACEs exposure throughout childhood (prenatal-18yrs) with moderate and severe depression trajectories (vs low) ­– Complete data analysis.** | | | | | | | | | | | | | |
| --- | --- | --- | --- | --- | --- | --- | --- | --- | --- | --- | --- | --- | --- |
| **Outcome: Depression trajectories** | | **Moderate Trajectory** | | | | | | **Severe Trajectory** | | | | | |
|  | **Model** | **OR** | **SE** | **Lower CI** | **Upper CI** | **p-value** | **p-value_fdr** | **OR** | **SE** | **Lower CI** | **Upper CI** | **p-value** | **p-value_fdr** |
|  |  |  |  |  |  |  |  |  |  |  |  |  |  |
| Total ACEs cumulative score | 1 | 1.23 | 0.04 | 1.14 | 1.31 | **0.00** | **0.00** | 1.57 | 0.06 | 1.45 | 1.70 | **0.00** | **0.00** |
| ***ACEs dimensions*** |  |  |  |  |  |  |  |  |  |  |  |  |  |
| Emotional/ Physical threat | 1 | 1.67 | 0.14 | 1.39 | 1.95 | **0.00** | **0.00** | 3.19 | 0.25 | 2.69 | 3.68 | **0.00** | **0.00** |
| Household Dysfunction | 1 | 1.76 | 0.15 | 1.46 | 2.06 | **0.00** | **0.00** | 3.45 | 0.31 | 2.84 | 4.05 | **0.00** | **0.00** |
| ***Single adversities*** |  |  |  |  |  |  |  |  |  |  |  |  |  |
| Physical abuse | 1 | 1.60 | 0.22 | 1.17 | 2.04 | **0.03** | 0.09 | 5.41 | 0.28 | 4.85 | 5.96 | **0.00** | **0.00** |
| Emotional abuse/neglect | 1 | 1.61 | 0.15 | 1.33 | 1.90 | **0.00** | **0.00** | 2.61 | 0.24 | 2.14 | 3.07 | **0.00** | **0.00** |
| Sexual abuse | 1 | 3.57 | 0.34 | 2.90 | 4.23 | **0.00** | **0.00** | 10.76 | 0.37 | 10.03 | 11.49 | **0.00** | **0.00** |
| Bullying | 1 | 1.67 | 0.16 | 1.37 | 1.98 | **0.00** | **0.00** | 1.41 | 0.26 | 0.91 | 1.91 | 0.18 | 0.35 |
| Household violence | 1 | 1.45 | 0.18 | 1.10 | 1.79 | **0.04** | 0.09 | 2.04 | 0.26 | 1.52 | 2.56 | **0.01** | **0.02** |
| Parental Substance Use Problems | 1 | 1.31 | 0.24 | 0.83 | 1.79 | 0.27 | 0.44 | 2.66 | 0.33 | 2.02 | 3.30 | **0.00** | **0.01** |
| Parental Mental Health Problems | 1 | 1.91 | 0.15 | 1.62 | 2.21 | **0.00** | **0.00** | 3.06 | 0.27 | 2.54 | 3.58 | **0.00** | **0.00** |
| Parental Convictions | 1 | 0.98 | 0.28 | 0.44 | 1.53 | 0.95 | 0.97 | 1.53 | 0.38 | 0.78 | 2.28 | 0.27 | 0.44 |
| Parental Separation | 1 | 0.89 | 0.17 | 0.56 | 1.23 | 0.51 | 0.65 | 1.49 | 0.25 | 0.99 | 1.99 | 0.12 | 0.26 |
| Low Parent-Child Bonding | 1 | 1.37 | 0.17 | 1.03 | 1.71 | 0.07 | 0.16 | 2.41 | 0.26 | 1.90 | 2.92 | **0.00** | **0.00** |
| **Note.** Sample: ALSPAC (N = 1,224). Estimates from multinomial logistic regression models in the complete data sample. Reference outcome: Low depression trajectory. CI = confidence interval. SE = standard error. fdr = false discovery rate (p-value). ACEs = adverse childhood experiences. Model 1: adjusted for sex, ethnicity, maternal smoking during pregnancy, mother’s marital status, mother’s education, and household’s social class; Model 2: Model 1 + earlier ACEs. P-values highlighted in bold are statistically significant at the 95% confidence level. | | | | | | | | | | | | | |

| **Table S42. Associations of the individual CRP measures and CRP trajectories with moderate and severe depression trajectories (vs low) – Complete data analysis.** | | | | | | | | | | |
| --- | --- | --- | --- | --- | --- | --- | --- | --- | --- | --- |
| **Outcome: Depression trajectories** | **Moderate Trajectory** | | | |  | **Severe Trajectory** | | | | |
|  | **OR** | **SE** | **Lower CI** | **Upper CI** | **p-value** | **OR** | **SE** | **Lower CI** | **Upper CI** | **p-value** |
| ***Single CRP measures*** |  |  |  |  |  |  |  |  |  |  |
| CRP 18 yrs (log) | 0.96 | 0.09 | 0.79 | 1.13 | 0.62 | 0.97 | 0.15 | 0.68 | 1.27 | 0.86 |
| CRP 15 yrs (log) | 0.92 | 0.09 | 0.75 | 1.10 | 0.38 | 1.01 | 0.15 | 0.72 | 1.30 | 0.95 |
| CRP 9 yrs (log) | 0.86 | 0.08 | 0.71 | 1.01 | 0.05 | 1.19 | 0.12 | 0.95 | 1.44 | 0.15 |
| ***CRP Trajectories*** |  |  |  |  |  |  |  |  |  |  |
| Low CRP Trajectory | ref |  |  |  |  |  |  |  |  |  |
| Moderate-High CRP Trajectory | 0.92 | 0.17 | 0.59 | 1.25 | 0.62 | 0.78 | 0.30 | 0.20 | 1.36 | 0.40 |
| High-Moderate CRP Trajectory | 0.84 | 0.24 | 0.37 | 1.32 | 0.49 | 1.80 | 0.32 | 1.18 | 2.43 | 0.07 |
| **Note.** Sample: ALSPAC (N = 1,242). Estimates from multinomial logistic regression models in the complete data sample. Reference outcome: Low depression trajectory. CI = confidence interval. SE = standard error. Model adjusted for sex, ethnicity, maternal smoking during pregnancy, mother’s marital status, mother’s education, and household’s social class. P-values highlighted in bold are statistically significant at the 95% confidence level. | | | | | | | | | | |

| **Table S43. Associations of the individual CRP measures and CRP trajectories with the risk of depression at age 18 years – Complete data analysis.** | | | | | |
| --- | --- | --- | --- | --- | --- |
| **Outcome: Depression, age 18 years** | | | | | |
|  | **OR** | **SE** | **Lower CI** | **Upper CI** | **p-value** |
| ***Single CRP measures*** | | | | | |
| CRP 18 yrs (log) | 0.97 | 0.11 | 0.75 | 1.19 | 0.78 |
| CRP 15 yrs (log) | 0.97 | 0.12 | 0.74 | 1.20 | 0.80 |
| CRP 9 yrs (log) | 0.91 | 0.10 | 0.70 | 1.11 | 0.36 |
| ***CRP Trajectories*** | | | | | |
| Low CRP Trajectory | ref |  |  |  |  |
| Moderate-High CRP Trajectory | 0.94 | 0.23 | 0.48 | 1.39 | 0.77 |
| High-Moderate CRP Trajectory | 1.29 | 0.30 | 0.70 | 1.89 | 0.40 |
| **Note.** Sample: ALSPAC (N = 1,242). Estimates from logistic regression models in the complete data sample. CI = confidence interval. SE = standard error. Model adjusted for sex, ethnicity, maternal smoking during pregnancy, mother’s marital status, mother’s education, and household’s social class. P-values highlighted in bold are statistically significant at the 95% confidence level. | | | | | |

| **Table S44. Longitudinal mediation effects of CRP trajectories (9-18 yrs) in the association between ACEs (prenatal-18yrs) and depression trajectories (18-23 yrs) – Complete data analysis.** | | | | | | | | | | |
| --- | --- | --- | --- | --- | --- | --- | --- | --- | --- | --- |
|  |  | **Mediation Effect (unexposed)** | | | **Mediation Effect (exposed)** | | | **Mediation Effect (average)** | | |
| **Exposure** | **Mediator** | **Estimate (ACME)** | **Lower CI** | **Upper CI** | **Estimate (ACME)** | **Lower CI** | **Upper CI** | **Estimate (ACME)** | **Lower CI** | **Upper CI** |
| **Outcome: Moderate depression trajectory** | |  |  |  |  |  |  |  |  |  |
| ACEs multiple-exposure (2+ ACEs) | Moderate-High CRP Traj | -0.003 | -0.016 | 0.001 | 0.000 | -0.004 | 0.002 | -0.001 | -0.010 | 0.001 |
|  | High-moderate CRP Traj | -0.001 | -0.003 | 0.008 | -0.001 | -0.004 | 0.002 | -0.001 | -0.002 | 0.002 |
| Emotional/ Physical threat | Moderate-High CRP Traj | 0.000 | -0.001 | 0.000 | 0.000 | -0.001 | 0.010 | 0.000 | -0.001 | 0.004 |
|  | High-moderate CRP Traj | 0.000 | -0.004 | 0.001 | 0.000 | -0.006 | 0.002 | 0.000 | -0.004 | 0.001 |
| Household Dysfunction | Moderate-High CRP Traj | 0.000 | -0.004 | 0.003 | -0.001 | -0.003 | 0.003 | 0.000 | -0.002 | 0.002 |
|  | High-moderate CRP Traj | 0.000 | -0.007 | 0.001 | 0.000 | -0.000 | 0.008 | 0.000 | -0.002 | 0.003 |
| Physical abuse | Moderate-High CRP Traj | 0.004 | -0.001 | 0.009 | -0.019 | -0.036 | 0.014 | -0.008 | -0.014 | 0.007 |
|  | High-moderate CRP Traj | -0.003 | -0.008 | 0.000 | 0.007 | -0.013 | 0.017 | 0.002 | -0.010 | 0.008 |
| Emotional abuse/neglect | Moderate-High CRP Traj | 0.000 | -0.005 | 0.001 | 0.000 | -0.002 | 0.003 | 0.000 | -0.003 | 0.001 |
|  | High-moderate CRP Traj | 0.000 | -0.003 | 0.001 | 0.000 | -0.006 | 0.004 | 0.000 | -0.004 | 0.002 |
| Sexual abuse | Moderate-High CRP Traj | 0.000 | -0.002 | 0.007 | 0.001 | -0.016 | 0.007 | 0.001 | -0.005 | 0.002 |
|  | High-moderate CRP Traj | 0.000 | -0.009 | 0.003 | -0.002 | -0.042 | 0.043 | -0.001 | -0.020 | 0.017 |
| Bullying | Moderate-High CRP Traj | 0.000 | -0.003 | 0.001 | 0.000 | -0.003 | 0.008 | 0.000 | -0.002 | 0.003 |
|  | High-moderate CRP Traj | -0.002 | -0.014 | 0.003 | -0.003 | -0.020 | 0.004 | -0.002 | -0.013 | 0.003 |
| Household violence | Moderate-High CRP Traj | -0.001 | -0.010 | 0.005 | 0.001 | -0.009 | 0.016 | 0.000 | -0.004 | 0.008 |
|  | High-moderate CRP Traj | -0.001 | -0.003 | 0.002 | -0.001 | -0.006 | 0.003 | -0.001 | -0.005 | 0.002 |
| Parental Substance Use Problems | Moderate-High CRP Traj | 0.000 | -0.001 | 0.007 | 0.001 | -0.004 | 0.010 | 0.001 | -0.002 | 0.006 |
|  | High-moderate CRP Traj | -0.001 | -0.002 | 0.005 | -0.002 | -0.007 | 0.010 | -0.001 | -0.004 | 0.007 |
| Parental Mental Health Problems | Moderate-High CRP Traj | -0.001 | -0.003 | 0.003 | -0.002 | -0.004 | 0.003 | -0.002 | -0.002 | 0.001 |
|  | High-moderate CRP Traj | 0.000 | -0.002 | 0.002 | 0.003 | -0.003 | 0.013 | 0.002 | -0.002 | 0.007 |
| Parental Convictions | Moderate-High CRP Traj | 0.000 | -0.003 | 0.006 | -0.001 | -0.019 | 0.030 | 0.000 | -0.007 | 0.014 |
|  | High-moderate CRP Traj | 0.001 | -0.004 | 0.008 | 0.010 | -0.003 | 0.035 | 0.005 | -0.002 | 0.020 |
| Parental Separation | Moderate-High CRP Traj | 0.000 | -0.003 | 0.007 | -0.002 | -0.006 | 0.001 | -0.001 | -0.002 | 0.001 |
|  | High-moderate CRP Traj | 0.000 | -0.002 | 0.003 | -0.001 | -0.007 | 0.014 | 0.000 | -0.002 | 0.008 |
| Low Parent-Child Bonding | Moderate-High CRP Traj | -0.001 | -0.006 | 0.001 | -0.003 | -0.009 | 0.001 | -0.002 | -0.005 | 0.000 |
|  | High-moderate CRP Traj | 0.000 | -0.001 | 0.003 | 0.000 | -0.013 | 0.003 | 0.000 | -0.007 | 0.002 |
| **Outcome: Severe depression trajectory** | | |  |  |  |  |  |  |  |  |
| ACEs multiple-exposure (2+ ACEs) | Moderate-High CRP Traj | 0.000 | -0.001 | 0.001 | -0.001 | -0.003 | 0.003 | 0.000 | -0.001 | 0.001 |
|  | High-moderate CRP Traj | -0.002 | -0.002 | 0.000 | 0.004 | -0.001 | 0.009 | 0.001 | -0.000 | 0.004 |
| Emotional/ Physical threat | Moderate-High CRP Traj | 0.001 | -0.003 | 0.002 | -0.002 | -0.005 | 0.005 | 0.000 | -0.001 | 0.002 |
|  | High-moderate CRP Traj | 0.001 | -0.005 | 0.003 | 0.006 | -0.003 | 0.007 | 0.004 | -0.003 | 0.005 |
| Household Dysfunction | Moderate-High CRP Traj | 0.000 | -0.002 | 0.002 | -0.001 | -0.004 | 0.000 | 0.000 | -0.002 | 0.000 |
|  | High-moderate CRP Traj | 0.001 | -0.004 | 0.005 | 0.002 | -0.008 | 0.004 | 0.001 | -0.006 | 0.004 |
| Physical abuse | Moderate-High CRP Traj | 0.002 | -0.001 | 0.009 | 0.001 | -0.021 | 0.017 | 0.002 | -0.008 | 0.009 |
|  | High-moderate CRP Traj | **0.002** | **0.001** | **0.008** | 0.003 | -0.006 | 0.011 | 0.003 | -0.003 | 0.010 |
| Emotional abuse/neglect | Moderate-High CRP Traj | 0.000 | -0.003 | 0.001 | 0.002 | -0.003 | 0.010 | 0.001 | -0.001 | 0.004 |
|  | High-moderate CRP Traj | 0.001 | -0.001 | 0.003 | 0.003 | -0.001 | 0.012 | 0.002 | -0.001 | 0.007 |
| Sexual abuse | Moderate-High CRP Traj | 0.000 | -0.003 | 0.003 | 0.028 | -0.054 | 0.067 | 0.014 | -0.028 | 0.034 |
|  | High-moderate CRP Traj | **-0.005** | **-0.014** | **-0.002** | -0.035 | -0.081 | 0.000 | -0.020 | -0.040 | 0.000 |
| Bullying | Moderate-High CRP Traj | -0.001 | -0.001 | 0.001 | -0.001 | -0.002 | 0.002 | -0.001 | -0.001 | 0.002 |
|  | High-moderate CRP Traj | **0.006** | **0.000** | **0.013** | 0.000 | -0.004 | 0.018 | 0.003 | -0.001 | 0.010 |
| Household violence | Moderate-High CRP Traj | 0.000 | -0.001 | 0.003 | 0.002 | -0.003 | 0.013 | 0.001 | -0.002 | 0.008 |
|  | High-moderate CRP Traj | 0.001 | -0.003 | 0.006 | 0.002 | -0.006 | 0.009 | 0.002 | -0.005 | 0.006 |
| Parental Substance Use Problems | Moderate-High CRP Traj | 0.001 | -0.000 | 0.005 | 0.015 | -0.004 | 0.021 | 0.008 | -0.002 | 0.012 |
|  | High-moderate CRP Traj | 0.003 | -0.004 | 0.008 | 0.012 | -0.035 | 0.020 | 0.007 | -0.019 | 0.011 |
| Parental Mental Health Problems | Moderate-High CRP Traj | 0.000 | -0.003 | 0.003 | 0.001 | -0.008 | 0.001 | 0.000 | -0.005 | 0.001 |
|  | High-moderate CRP Traj | 0.000 | -0.002 | 0.005 | 0.002 | -0.009 | 0.005 | 0.001 | -0.004 | 0.005 |
| Parental Convictions | Moderate-High CRP Traj | 0.001 | -0.002 | 0.005 | 0.000 | -0.010 | 0.007 | 0.000 | -0.003 | 0.004 |
|  | High-moderate CRP Traj | -0.004 | -0.005 | 0.002 | -0.003 | -0.012 | 0.014 | -0.004 | -0.008 | 0.008 |
| Parental Separation | Moderate-High CRP Traj | **-0.002** | **-0.006** | **-0.001** | -0.002 | -0.008 | 0.000 | -0.002 | -0.004 | 0.000 |
|  | High-moderate CRP Traj | 0.002 | -0.001 | 0.005 | 0.003 | -0.005 | 0.014 | 0.002 | -0.003 | 0.009 |
| Low Parent-Child Bonding | Moderate-High CRP Traj | 0.000 | -0.001 | 0.001 | -0.001 | -0.006 | 0.001 | -0.001 | -0.003 | 0.001 |
|  | High-moderate CRP Traj | -0.001 | -0.001 | 0.010 | -0.001 | -0.000 | 0.010 | -0.001 | -0.000 | 0.010 |
| **Note.** Sample: ALSPAC (N = 1,224). Estimates from causal mediation analysis in the complete data sample. The outcome and mediator models were estimated using logistic regression analysis. The estimation of the mediation effects in the unexposed and exposed groups allows the investigation of possible interactions between the exposure and mediator variables. Values in bold represent statistically significant coefficients at the 5% significance level (p<0.05). ACME = average causal mediation effect. | | | | | | | | | | |

| **Table S45. Comparison of sample characteristics between the analytical sample and non-included ALSPAC participants.** | | | | |
| --- | --- | --- | --- | --- |
|  | **Non-included (N=8439)** | **Included (N=3931)** | **P-value comparison** | **Correlation (r)** |
| **Sex** |  |  | < 0.001 | 0.155 |
| Male | 56.6% | 40.1% |  |  |
| Female | 43.4% | 59.9% |  |  |
| **Ethnicity** |  |  | 0.005 | -0.029 |
| White | 95.1% | 96.3% |  |  |
| Other | 4.9% | 3.7% |  |  |
| Missing | 10.9% | 6.9% |  |  |
| **Parental occupational class** |  |  | < 0.001 | -0.133 |
| Professional | 7.8% | 13.2% |  |  |
| Managerial and technical | 29.7% | 37.3% |  |  |
| Skilled non-manual | 12.3% | 13.6% |  |  |
| Skilled manual | 40.3% | 29.7% |  |  |
| Partly skilled | 6.7% | 4.4% |  |  |
| Unskilled | 3.2% | 1.8% |  |  |
| Missing | 32.1% | 23.6% |  |  |
| **Mother's education** |  |  | < 0.001 | 0.216 |
| CSE | 22.6% | 9.6% |  |  |
| Vocational | 11.1% | 6.7% |  |  |
| O level | 35.9% | 33.6% |  |  |
| A level | 20.1% | 29.8% |  |  |
| Degree | 10.2% | 20.3% |  |  |
| Missing | 8.3% | 6.0% |  |  |
| **Mother's marital status at birth** |  |  | < 0.001 | 0.090 |
| Never married | 19.5% | 12.5% |  |  |
| Widowed | 0.2% | 0.1% |  |  |
| Divorced/Separated | 6.1% | 4.2% |  |  |
| 1st marriage | 67.8% | 77.0% |  |  |
| 2nd/3rd marriage | 6.5% | 6.3% |  |  |
| Missing | 6.2% | 5.4% |  |  |
| **Maternal smoking during pregnancy** |  |  | < 0.001 | -0.123 |
| No | 72.1% | 83.5% |  |  |
| Yes | 27.9% | 16.5% |  |  |
| **Note.** P-values from chi-square tests. r coefficients obtained from Spearman’s Rho correlation tests. The analytical sample includes participants with at least 10% of ACEs data across all early-life periods, one measure of CRP, and one measure of depressive symptoms. | | | | |

| **Table S46. Associations of ACEs with individual CRP measures.** | | | | | | | | | | | | | | | |
| --- | --- | --- | --- | --- | --- | --- | --- | --- | --- | --- | --- | --- | --- | --- | --- |
|  | **Outcome: CRP 9 yrs (log)** | | | | | **Outcome: CRP 15 yrs (log)** | | | | | **Outcome: CRP 18 yrs (log)** | | | | |
| **Exposure: ACEs** | **B** | **SE** | **p-value** | **CI lower** | **CI upper** | **B** | **SE** | **p-value** | **CI lower** | **CI upper** | **B** | **SE** | **p-value** | **CI lower** | **CI upper** |
| **Prenatal** |  |  |  |  |  |  |  |  |  |  |  |  |  |  |  |
| Total ACEs cumulative score, prenatal | -0.014 | 0.034 | 0.685 | -0.081 | 0.054 | -0.029 | 0.036 | 0.416 | -0.100 | 0.042 | -0.037 | 0.034 | 0.268 | -0.103 | 0.029 |
| Emotional/ Physical threat, prenatal | 0.093 | 0.214 | 0.664 | -0.326 | 0.512 | -0.003 | 0.213 | 0.990 | -0.421 | 0.415 | 0.042 | 0.192 | 0.828 | -0.334 | 0.417 |
| Household Dysfunction, prenatal | -0.028 | 0.044 | 0.529 | -0.115 | 0.059 | -0.053 | 0.043 | 0.216 | -0.137 | 0.031 | -0.046 | 0.044 | 0.290 | -0.133 | 0.040 |
| Household violence, prenatal | -0.015 | 0.253 | 0.952 | -0.511 | 0.481 | -0.089 | 0.240 | 0.710 | -0.559 | 0.381 | -0.090 | 0.225 | 0.691 | -0.531 | 0.352 |
| Parental Substance Use Problems, prenatal | 0.047 | 0.139 | 0.735 | -0.225 | 0.319 | -0.110 | 0.110 | 0.320 | -0.326 | 0.107 | -0.012 | 0.110 | 0.915 | -0.227 | 0.204 |
| Parental Mental Health Problems, prenatal | -0.027 | 0.048 | 0.583 | -0.122 | 0.068 | -0.021 | 0.046 | 0.654 | -0.112 | 0.070 | -0.040 | 0.046 | 0.380 | -0.130 | 0.050 |
| Parental Convictions, prenatal | -0.073 | 0.182 | 0.689 | -0.430 | 0.284 | -0.197 | 0.166 | 0.236 | -0.523 | 0.129 | -0.181 | 0.168 | 0.281 | -0.510 | 0.148 |
| Parental Separation, prenatal | -0.021 | 0.101 | 0.836 | -0.219 | 0.177 | 0.030 | 0.097 | 0.760 | -0.161 | 0.221 | -0.035 | 0.096 | 0.719 | -0.224 | 0.154 |
| **0-3 years** |  |  |  |  |  |  |  |  |  |  |  |  |  |  |  |
| Total ACEs cumulative score, 0-3yrs | -0.013 | 0.025 | 0.616 | -0.062 | 0.037 | 0.005 | 0.022 | 0.819 | -0.038 | 0.048 | -0.018 | 0.020 | 0.350 | -0.057 | 0.020 |
| Emotional/ Physical threat, 0-3yrs | -0.069 | 0.069 | 0.321 | -0.204 | 0.067 | -0.005 | 0.062 | 0.939 | -0.127 | 0.117 | -0.065 | 0.060 | 0.280 | -0.184 | 0.053 |
| Household Dysfunction, 0-3yrs | -0.032 | 0.046 | 0.487 | -0.122 | 0.058 | 0.023 | 0.042 | 0.575 | -0.058 | 0.105 | -0.021 | 0.040 | 0.602 | -0.099 | 0.057 |
| Physical abuse, 0-3yrs | 0.072 | 0.113 | 0.525 | -0.150 | 0.294 | -0.139 | 0.109 | 0.201 | -0.352 | 0.074 | -0.033 | 0.104 | 0.748 | -0.237 | 0.171 |
| Emotional abuse/neglect, 0-3yrs | -0.095 | 0.079 | 0.233 | -0.250 | 0.061 | -0.023 | 0.073 | 0.756 | -0.166 | 0.120 | -0.041 | 0.071 | 0.565 | -0.180 | 0.098 |
| Household violence, 0-3yrs | 0.032 | 0.094 | 0.733 | -0.152 | 0.216 | -0.034 | 0.090 | 0.707 | -0.210 | 0.142 | 0.016 | 0.088 | 0.852 | -0.156 | 0.189 |
| Parental Substance Use Problems, 0-3yrs | 0.059 | 0.102 | 0.560 | -0.140 | 0.259 | 0.012 | 0.096 | 0.903 | -0.176 | 0.200 | 0.012 | 0.102 | 0.909 | -0.189 | 0.212 |
| Parental Mental Health Problems, 0-3yrs | -0.071 | 0.050 | 0.156 | -0.170 | 0.027 | 0.023 | 0.045 | 0.611 | -0.065 | 0.111 | -0.024 | 0.044 | 0.584 | -0.110 | 0.062 |
| Parental Convictions, 0-3yrs | 0.012 | 0.118 | 0.921 | -0.219 | 0.243 | -0.048 | 0.105 | 0.651 | -0.253 | 0.158 | 0.024 | 0.119 | 0.840 | -0.209 | 0.256 |
| Parental Separation, 0-3yrs | 0.066 | 0.084 | 0.432 | -0.099 | 0.230 | 0.032 | 0.070 | 0.645 | -0.105 | 0.169 | -0.059 | 0.078 | 0.451 | -0.212 | 0.094 |
| Low Parent-Child Bonding, 0-3yrs | 0.008 | 0.118 | 0.946 | -0.223 | 0.239 | 0.160 | 0.112 | 0.153 | -0.059 | 0.380 | -0.105 | 0.107 | 0.330 | -0.315 | 0.106 |
| **3-7 years** |  |  |  |  |  |  |  |  |  |  |  |  |  |  |  |
| Total ACEs cumulative score, 3-7yrs | 0.007 | 0.021 | 0.727 | -0.034 | 0.049 | -0.001 | 0.019 | 0.968 | -0.038 | 0.037 | -0.009 | 0.019 | 0.615 | -0.046 | 0.027 |
| Emotional/ Physical threat, 3-7yrs | -0.041 | 0.055 | 0.456 | -0.148 | 0.066 | 0.006 | 0.049 | 0.900 | -0.090 | 0.102 | 0.003 | 0.049 | 0.955 | -0.093 | 0.099 |
| Household Dysfunction, 3-7yrs | 0.022 | 0.045 | 0.633 | -0.067 | 0.110 | -0.002 | 0.037 | 0.967 | -0.074 | 0.071 | -0.005 | 0.044 | 0.914 | -0.091 | 0.082 |
| Physical abuse, 3-7yrs | 0.059 | 0.103 | 0.568 | -0.144 | 0.262 | -0.027 | 0.104 | 0.797 | -0.232 | 0.178 | -0.104 | 0.101 | 0.303 | -0.302 | 0.094 |
| Emotional abuse/neglect, 3-7yrs | -0.037 | 0.070 | 0.591 | -0.174 | 0.099 | 0.012 | 0.067 | 0.860 | -0.120 | 0.143 | -0.011 | 0.061 | 0.856 | -0.130 | 0.108 |
| Sexual abuse, 3-7yrs | 0.085 | 0.295 | 0.774 | -0.494 | 0.664 | -0.007 | 0.247 | 0.976 | -0.491 | 0.477 | -0.133 | 0.282 | 0.638 | -0.686 | 0.420 |
| Household violence, 3-7yrs | 0.066 | 0.086 | 0.442 | -0.103 | 0.236 | 0.030 | 0.091 | 0.742 | -0.148 | 0.208 | -0.003 | 0.087 | 0.974 | -0.174 | 0.169 |
| Parental Substance Use Problems, 3-7yrs | 0.048 | 0.091 | 0.596 | -0.129 | 0.226 | -0.020 | 0.093 | 0.830 | -0.202 | 0.162 | -0.018 | 0.090 | 0.843 | -0.195 | 0.159 |
| Parental Mental Health Problems, 3-7yrs | -0.010 | 0.054 | 0.850 | -0.116 | 0.095 | 0.005 | 0.046 | 0.906 | -0.085 | 0.096 | -0.023 | 0.058 | 0.696 | -0.135 | 0.090 |
| Parental Convictions, 3-7yrs | 0.032 | 0.128 | 0.802 | -0.219 | 0.283 | 0.028 | 0.136 | 0.837 | -0.239 | 0.295 | 0.040 | 0.125 | 0.752 | -0.206 | 0.286 |
| Parental Separation, 3-7yrs | 0.054 | 0.067 | 0.420 | -0.078 | 0.186 | -0.030 | 0.060 | 0.616 | -0.148 | 0.088 | 0.007 | 0.061 | 0.913 | -0.112 | 0.126 |
| Low Parent-Child Bonding, 3-7yrs | -0.019 | 0.064 | 0.767 | -0.145 | 0.107 | -0.001 | 0.065 | 0.985 | -0.129 | 0.127 | -0.013 | 0.058 | 0.830 | -0.127 | 0.102 |
| **7-12 years** |  |  |  |  |  |  |  |  |  |  |  |  |  |  |  |
| Total ACEs cumulative score, 7-12yrs | 0.022 | 0.021 | 0.292 | -0.019 | 0.063 | 0.004 | 0.018 | 0.829 | -0.032 | 0.040 | 0.002 | 0.019 | 0.913 | -0.036 | 0.040 |
| Emotional/ Physical threat, 7-12yrs | 0.030 | 0.048 | 0.535 | -0.064 | 0.124 | -0.001 | 0.049 | 0.981 | -0.096 | 0.094 | -0.015 | 0.049 | 0.760 | -0.111 | 0.081 |
| Household Dysfunction, 7-12yrs | 0.009 | 0.054 | 0.871 | -0.098 | 0.115 | -0.005 | 0.040 | 0.902 | -0.083 | 0.073 | 0.012 | 0.049 | 0.801 | -0.083 | 0.108 |
| Physical abuse, 7-12yrs | 0.121 | 0.193 | 0.530 | -0.257 | 0.500 | -0.201 | 0.172 | 0.242 | -0.539 | 0.136 | 0.004 | 0.184 | 0.984 | -0.357 | 0.365 |
| Emotional abuse/neglect, 7-12yrs | 0.050 | 0.075 | 0.504 | -0.097 | 0.197 | -0.017 | 0.072 | 0.817 | -0.157 | 0.124 | -0.051 | 0.070 | 0.461 | -0.189 | 0.086 |
| Bullying, 7-12yrs | 0.085 | 0.067 | 0.203 | -0.046 | 0.217 | 0.038 | 0.056 | 0.501 | -0.073 | 0.149 | 0.016 | 0.063 | 0.805 | -0.109 | 0.140 |
| Household violence, 7-12yrs | -0.013 | 0.104 | 0.899 | -0.217 | 0.190 | -0.046 | 0.096 | 0.631 | -0.234 | 0.142 | 0.025 | 0.094 | 0.793 | -0.160 | 0.210 |
| Parental Substance Use Problems, 7-12yrs | -0.028 | 0.118 | 0.816 | -0.260 | 0.204 | -0.073 | 0.101 | 0.471 | -0.271 | 0.125 | -0.135 | 0.119 | 0.257 | -0.369 | 0.099 |
| Parental Mental Health Problems, 7-12yrs | 0.031 | 0.072 | 0.670 | -0.110 | 0.172 | 0.012 | 0.058 | 0.838 | -0.102 | 0.125 | 0.072 | 0.061 | 0.241 | -0.048 | 0.191 |
| Parental Convictions, 7-12yrs | -0.066 | 0.149 | 0.659 | -0.358 | 0.227 | 0.081 | 0.139 | 0.559 | -0.192 | 0.354 | -0.013 | 0.160 | 0.937 | -0.327 | 0.302 |
| Parental Separation, 7-12yrs | 0.044 | 0.072 | 0.541 | -0.097 | 0.185 | 0.027 | 0.054 | 0.619 | -0.079 | 0.133 | -0.011 | 0.067 | 0.865 | -0.142 | 0.119 |
| Low Parent-Child Bonding, 7-12yrs | 0.012 | 0.056 | 0.836 | -0.098 | 0.121 | 0.012 | 0.059 | 0.832 | -0.102 | 0.127 | 0.002 | 0.059 | 0.974 | -0.113 | 0.117 |
| **12-18 years** |  |  |  |  |  |  |  |  |  |  |  |  |  |  |  |
| Total ACEs cumulative score, 12-18yrs | 0.027 | 0.021 | 0.196 | -0.014 | 0.067 | 0.028 | 0.018 | 0.115 | -0.007 | 0.064 | 0.019 | 0.019 | 0.318 | -0.019 | 0.057 |
| Emotional/ Physical threat, 12-18yrs | 0.000 | 0.046 | 0.997 | -0.089 | 0.090 | -0.005 | 0.039 | 0.889 | -0.083 | 0.072 | -0.011 | 0.040 | 0.784 | -0.090 | 0.068 |
| Household Dysfunction, 12-18yrs | 0.096 | 0.056 | 0.085 | -0.013 | 0.205 | 0.081 | 0.050 | 0.106 | -0.017 | 0.180 | 0.050 | 0.046 | 0.274 | -0.039 | 0.139 |
| Physical abuse, 12-18yrs | 0.028 | 0.079 | 0.719 | -0.126 | 0.183 | 0.017 | 0.077 | 0.828 | -0.134 | 0.168 | 0.089 | 0.077 | 0.248 | -0.062 | 0.241 |
| Emotional abuse/neglect, 12-18yrs | 0.011 | 0.050 | 0.819 | -0.086 | 0.109 | -0.007 | 0.043 | 0.871 | -0.091 | 0.077 | -0.011 | 0.047 | 0.813 | -0.104 | 0.081 |
| Sexual abuse, 12-18yrs | -0.007 | 0.107 | 0.949 | -0.217 | 0.203 | 0.104 | 0.096 | 0.281 | -0.085 | 0.292 | 0.013 | 0.100 | 0.894 | -0.183 | 0.210 |
| Bullying, 12-18yrs | 0.027 | 0.054 | 0.615 | -0.078 | 0.132 | 0.049 | 0.047 | 0.299 | -0.043 | 0.140 | 0.022 | 0.054 | 0.680 | -0.083 | 0.127 |
| Household violence, 12-18yrs | 0.099 | 0.087 | 0.254 | -0.071 | 0.268 | 0.039 | 0.075 | 0.599 | -0.107 | 0.186 | 0.026 | 0.067 | 0.700 | -0.105 | 0.157 |
| Parental Substance Use Problems, 12-18yrs | 0.052 | 0.239 | 0.829 | -0.417 | 0.520 | 0.243 | 0.169 | 0.150 | -0.088 | 0.574 | 0.321 | 0.174 | 0.065 | -0.020 | 0.661 |
| Parental Mental Health Problems, 12-18yrs | 0.119 | 0.168 | 0.476 | -0.209 | 0.448 | 0.135 | 0.126 | 0.283 | -0.112 | 0.382 | 0.063 | 0.152 | 0.679 | -0.235 | 0.361 |
| Parental Convictions, 12-18yrs | -0.069 | 0.238 | 0.771 | -0.537 | 0.398 | 0.290 | 0.248 | 0.243 | -0.197 | 0.777 | -0.085 | 0.240 | 0.722 | -0.556 | 0.385 |
| Parental Separation, 12-18yrs | 0.084 | 0.068 | 0.218 | -0.049 | 0.217 | 0.071 | 0.065 | 0.278 | -0.057 | 0.199 | 0.065 | 0.066 | 0.324 | -0.064 | 0.194 |
| Low Parent-Child Bonding, 12-18yrs | -0.008 | 0.071 | 0.906 | -0.148 | 0.131 | -0.012 | 0.060 | 0.838 | -0.131 | 0.106 | -0.013 | 0.061 | 0.830 | -0.133 | 0.106 |
| **Prenatal-18 years** |  |  |  |  |  |  |  |  |  |  |  |  |  |  |  |
| Total ACEs cumulative score, prenatal-18yrs | 0.008 | 0.013 | 0.525 | -0.017 | 0.033 | 0.008 | 0.012 | 0.519 | -0.015 | 0.030 | 0.002 | 0.012 | 0.830 | -0.020 | 0.025 |
| Emotional/ Physical threat, prenatal-18yrs | -0.019 | 0.041 | 0.649 | -0.099 | 0.062 | 0.009 | 0.037 | 0.815 | -0.065 | 0.082 | -0.017 | 0.040 | 0.678 | -0.096 | 0.062 |
| Household Dysfunction, prenatal-18yrs | 0.014 | 0.048 | 0.769 | -0.080 | 0.108 | 0.017 | 0.043 | 0.698 | -0.068 | 0.101 | 0.011 | 0.046 | 0.803 | -0.079 | 0.102 |
| Physical abuse, prenatal-18yrs | 0.037 | 0.062 | 0.551 | -0.084 | 0.158 | -0.026 | 0.057 | 0.653 | -0.138 | 0.086 | 0.010 | 0.057 | 0.863 | -0.102 | 0.122 |
| Emotional abuse/neglect, prenatal-18yrs | -0.019 | 0.046 | 0.671 | -0.109 | 0.070 | 0.001 | 0.038 | 0.978 | -0.073 | 0.075 | -0.016 | 0.041 | 0.689 | -0.097 | 0.064 |
| Sexual abuse, prenatal-18yrs | 0.003 | 0.106 | 0.976 | -0.204 | 0.211 | 0.102 | 0.093 | 0.272 | -0.080 | 0.284 | -0.009 | 0.096 | 0.924 | -0.198 | 0.180 |
| Bullying, prenatal-18yrs | 0.061 | 0.048 | 0.205 | -0.033 | 0.154 | 0.058 | 0.040 | 0.143 | -0.020 | 0.136 | 0.024 | 0.047 | 0.609 | -0.068 | 0.115 |
| Household violence, prenatal-18yrs | 0.033 | 0.053 | 0.532 | -0.071 | 0.137 | 0.017 | 0.040 | 0.682 | -0.063 | 0.096 | 0.036 | 0.047 | 0.440 | -0.056 | 0.128 |
| Parental Substance Use Problems, prenatal-18yrs | 0.034 | 0.075 | 0.652 | -0.114 | 0.182 | -0.029 | 0.064 | 0.656 | -0.155 | 0.098 | 0.012 | 0.063 | 0.848 | -0.112 | 0.137 |
| Parental Mental Health Problems, prenatal-18yrs | -0.033 | 0.044 | 0.451 | -0.119 | 0.053 | 0.012 | 0.043 | 0.774 | -0.071 | 0.096 | -0.021 | 0.042 | 0.620 | -0.103 | 0.061 |
| Parental Convictions, prenatal-18yrs | 0.004 | 0.076 | 0.959 | -0.145 | 0.153 | -0.016 | 0.077 | 0.831 | -0.167 | 0.135 | 0.001 | 0.073 | 0.988 | -0.142 | 0.144 |
| Parental Separation, prenatal-18yrs | 0.066 | 0.045 | 0.144 | -0.023 | 0.155 | 0.014 | 0.043 | 0.739 | -0.070 | 0.099 | 0.026 | 0.045 | 0.572 | -0.063 | 0.115 |
| Low Parent-Child Bonding, prenatal-18yrs | -0.027 | 0.049 | 0.575 | -0.123 | 0.068 | 0.010 | 0.044 | 0.826 | -0.077 | 0.097 | -0.018 | 0.047 | 0.704 | -0.109 | 0.074 |
| **Note.** Sample: ALSPAC (N = 3,931). Pooled estimates from linear regression models across 20 imputed datasets. CI = confidence interval. SE = standard error. Models adjusted for sex, ethnicity, maternal smoking during pregnancy, mother’s marital status, mother’s education, and household’s social class. P-values highlighted in bold are statistically significant at the 95% confidence level. | | | | | | | | | | | | | | | |

| **Table S47. Growth curve mixed-effects models: Associations of ACEs with baseline CRP levels (log) and the rate of change in CRP levels with time (i.e. age).** | | | | | |
| --- | --- | --- | --- | --- | --- |
| **Exposure: ACEs** | **B** | **SE** | **p-value** | **CI lower** | **CI upper** |
| **ACEs prenatal period** |  |  |  |  |  |
| Age | 0.094 | 0.003 | **<0.001** | 0.088 | 0.100 |
| Total ACEs cumulative score, prenatal | 0.006 | 0.072 | 0.931 | -0.135 | 0.147 |
| Age*Total ACEs cumulative score, prenatal | -0.002 | 0.005 | 0.617 | -0.012 | 0.007 |
| Age | 0.094 | 0.003 | **<0.001** | 0.088 | 0.099 |
| Emotional/ Physical threat, prenatal | 0.115 | 0.462 | 0.804 | -0.791 | 1.021 |
| Age*Emotional/ Physical threat, prenatal | -0.005 | 0.031 | 0.868 | -0.065 | 0.055 |
| Age | 0.094 | 0.003 | **<0.001** | 0.088 | 0.100 |
| Household Dysfunction, prenatal | -0.013 | 0.091 | 0.887 | -0.192 | 0.166 |
| Age*Household Dysfunction, prenatal | -0.002 | 0.006 | 0.725 | -0.014 | 0.010 |
| Age | 0.094 | 0.003 | **<0.001** | 0.088 | 0.099 |
| Household violence, prenatal | 0.092 | 0.507 | 0.856 | -0.902 | 1.086 |
| Age*Household violence, prenatal | -0.011 | 0.033 | 0.737 | -0.076 | 0.054 |
| Age | 0.094 | 0.003 | **<0.001** | 0.088 | 0.099 |
| Parental Substance Use Problems, prenatal | 0.131 | 0.268 | 0.624 | -0.394 | 0.656 |
| Age*Parental Substance Use Problems, prenatal | -0.011 | 0.017 | 0.518 | -0.045 | 0.023 |
| Age | 0.094 | 0.003 | **<0.001** | 0.088 | 0.100 |
| Parental Mental Health Problems, prenatal | -0.014 | 0.100 | 0.885 | -0.210 | 0.181 |
| Age*Parental Mental Health Problems, prenatal | -0.001 | 0.007 | 0.870 | -0.014 | 0.012 |
| Age | 0.094 | 0.003 | **<0.001** | 0.088 | 0.099 |
| Parental Convictions, prenatal | 0.032 | 0.390 | 0.935 | -0.733 | 0.796 |
| Age*Parental Convictions, prenatal | -0.013 | 0.026 | 0.618 | -0.064 | 0.038 |
| Age | 0.094 | 0.003 | <0.001 | 0.088 | 0.099 |
| Parental Separation, prenatal | -0.019 | 0.202 | 0.927 | -0.415 | 0.378 |
| Age*Parental Separation, prenatal | 0.001 | 0.013 | 0.959 | -0.026 | 0.027 |
| **ACEs 0-3 years** |  |  |  |  |  |
| Age | 0.094 | 0.003 | **<0.001** | 0.087 | 0.100 |
| Total ACEs cumulative score, 0-3yrs | -0.009 | 0.051 | 0.862 | -0.109 | 0.091 |
| Age*Total ACEs cumulative score, 0-3yrs | 0.000 | 0.003 | 0.997 | -0.006 | 0.006 |
| Age | 0.093 | 0.003 | **<0.001** | 0.087 | 0.099 |
| Emotional/ Physical threat, 0-3yrs | -0.081 | 0.150 | 0.587 | -0.374 | 0.212 |
| Age*Emotional/ Physical threat, 0-3yrs | 0.002 | 0.010 | 0.805 | -0.017 | 0.022 |
| Age | 0.093 | 0.004 | **<0.001** | 0.085 | 0.100 |
| Household Dysfunction, 0-3yrs | -0.047 | 0.093 | 0.614 | -0.229 | 0.135 |
| Age*Household Dysfunction, 0-3yrs | 0.003 | 0.006 | 0.668 | -0.009 | 0.015 |
| Age | 0.094 | 0.003 | **<0.001** | 0.088 | 0.100 |
| Physical abuse, 0-3yrs | 0.169 | 0.245 | 0.491 | -0.311 | 0.649 |
| Age*Physical abuse, 0-3yrs | -0.014 | 0.016 | 0.378 | -0.047 | 0.018 |
| Age | 0.093 | 0.003 | **<0.001** | 0.087 | 0.099 |
| Emotional abuse/neglect, 0-3yrs | -0.152 | 0.165 | 0.355 | -0.475 | 0.170 |
| Age*Emotional abuse/neglect, 0-3yrs | 0.007 | 0.011 | 0.513 | -0.014 | 0.028 |
| Age | 0.094 | 0.003 | **<0.001** | 0.088 | 0.099 |
| Household violence, 0-3yrs | 0.036 | 0.197 | 0.855 | -0.350 | 0.423 |
| Age*Household violence, 0-3yrs | -0.002 | 0.013 | 0.864 | -0.028 | 0.023 |
| Age | 0.094 | 0.003 | **<0.001** | 0.088 | 0.099 |
| Parental Substance Use Problems, 0-3yrs | 0.106 | 0.201 | 0.598 | -0.287 | 0.499 |
| Age*Parental Substance Use Problems, 0-3yrs | -0.006 | 0.013 | 0.678 | -0.032 | 0.021 |
| Age | 0.092 | 0.003 | **<0.001** | 0.085 | 0.098 |
| Parental Mental Health Problems, 0-3yrs | -0.116 | 0.105 | 0.267 | -0.321 | 0.089 |
| Age*Parental Mental Health Problems, 0-3yrs | 0.007 | 0.007 | 0.343 | -0.007 | 0.020 |
| Age | 0.094 | 0.003 | **<0.001** | 0.088 | 0.099 |
| Parental Convictions, 0-3yrs | 0.006 | 0.248 | 0.982 | -0.480 | 0.492 |
| Age*Parental Convictions, 0-3yrs | -0.001 | 0.017 | 0.968 | -0.034 | 0.033 |
| Age | 0.094 | 0.003 | **<0.001** | 0.089 | 0.100 |
| Parental Separation, 0-3yrs | 0.172 | 0.162 | 0.288 | -0.145 | 0.490 |
| Age*Parental Separation, 0-3yrs | -0.011 | 0.010 | 0.275 | -0.032 | 0.009 |
| Age | 0.094 | 0.003 | **<0.001** | 0.088 | 0.099 |
| Low Parent-Child Bonding, 0-3yrs | 0.098 | 0.253 | 0.698 | -0.398 | 0.595 |
| Age*Low Parent-Child Bonding, 0-3yrs | -0.006 | 0.017 | 0.747 | -0.039 | 0.028 |
| **ACEs 3-7 years** |  |  |  |  |  |
| Age | 0.095 | 0.004 | **<0.001** | 0.088 | 0.102 |
| Total ACEs cumulative score, 3-7yrs | 0.024 | 0.046 | 0.594 | -0.065 | 0.114 |
| Age*Total ACEs cumulative score, 3-7yrs | -0.002 | 0.003 | 0.555 | -0.008 | 0.004 |
| Age | 0.092 | 0.003 | **<0.001** | 0.086 | 0.099 |
| Emotional/ Physical threat, 3-7yrs | -0.089 | 0.124 | 0.471 | -0.331 | 0.153 |
| Age*Emotional/ Physical threat, 3-7yrs | 0.006 | 0.008 | 0.503 | -0.011 | 0.022 |
| Age | 0.095 | 0.004 | **<0.001** | 0.087 | 0.102 |
| Household Dysfunction, 3-7yrs | 0.045 | 0.097 | 0.643 | -0.146 | 0.236 |
| Age*Household Dysfunction, 3-7yrs | -0.003 | 0.007 | 0.665 | -0.016 | 0.010 |
| Age | 0.094 | 0.003 | **<0.001** | 0.089 | 0.100 |
| Physical abuse, 3-7yrs | 0.227 | 0.225 | 0.312 | -0.213 | 0.667 |
| Age*Physical abuse, 3-7yrs | -0.018 | 0.016 | 0.255 | -0.049 | 0.013 |
| Age | 0.093 | 0.003 | **<0.001** | 0.087 | 0.099 |
| Emotional abuse/neglect, 3-7yrs | -0.064 | 0.145 | 0.661 | -0.348 | 0.220 |
| Age*Emotional abuse/neglect, 3-7yrs | 0.004 | 0.010 | 0.701 | -0.015 | 0.022 |
| Age | 0.094 | 0.003 | **<0.001** | 0.088 | 0.099 |
| Sexual abuse, 3-7yrs | 0.418 | 0.633 | 0.509 | -0.822 | 1.659 |
| Age*Sexual abuse, 3-7yrs | -0.031 | 0.043 | 0.471 | -0.115 | 0.053 |
| Age | 0.094 | 0.003 | **<0.001** | 0.088 | 0.100 |
| Household violence, 3-7yrs | 0.121 | 0.185 | 0.513 | -0.242 | 0.485 |
| Age*Household violence, 3-7yrs | -0.006 | 0.013 | 0.621 | -0.032 | 0.019 |
| Age | 0.094 | 0.003 | **<0.001** | 0.088 | 0.100 |
| Parental Substance Use Problems, 3-7yrs | 0.118 | 0.188 | 0.530 | -0.251 | 0.488 |
| Age*Parental Substance Use Problems, 3-7yrs | -0.008 | 0.012 | 0.511 | -0.033 | 0.016 |
| Age | 0.094 | 0.003 | **<0.001** | 0.087 | 0.100 |
| Parental Mental Health Problems, 3-7yrs | 0.001 | 0.125 | 0.996 | -0.244 | 0.245 |
| Age*Parental Mental Health Problems, 3-7yrs | -0.001 | 0.009 | 0.937 | -0.018 | 0.016 |
| Age | 0.094 | 0.003 | **<0.001** | 0.088 | 0.099 |
| Parental Convictions, 3-7yrs | 0.033 | 0.258 | 0.898 | -0.473 | 0.540 |
| Age*Parental Convictions, 3-7yrs | 0.000 | 0.017 | 0.999 | -0.034 | 0.034 |
| Age | 0.094 | 0.003 | **<0.001** | 0.088 | 0.100 |
| Parental Separation, 3-7yrs | 0.115 | 0.135 | 0.398 | -0.151 | 0.380 |
| Age*Parental Separation, 3-7yrs | -0.007 | 0.009 | 0.404 | -0.025 | 0.010 |
| Age | 0.093 | 0.003 | **<0.001** | 0.087 | 0.099 |
| Low Parent-Child Bonding, 3-7yrs | -0.035 | 0.139 | 0.802 | -0.307 | 0.237 |
| Age*Low Parent-Child Bonding, 3-7yrs | 0.002 | 0.009 | 0.856 | -0.017 | 0.020 |
| **ACEs 7-12 years** |  |  |  |  |  |
| Age | 0.095 | 0.004 | **<0.001** | 0.088 | 0.102 |
| Total ACEs cumulative score, 7-12yrs | 0.040 | 0.043 | 0.353 | -0.044 | 0.123 |
| Age*Total ACEs cumulative score, 7-12yrs | -0.002 | 0.003 | 0.445 | -0.008 | 0.003 |
| Age | 0.095 | 0.003 | **<0.001** | 0.088 | 0.101 |
| Emotional/ Physical threat, 7-12yrs | 0.075 | 0.102 | 0.464 | -0.125 | 0.275 |
| Age*Emotional/ Physical threat, 7-12yrs | -0.005 | 0.007 | 0.474 | -0.019 | 0.009 |
| Age | 0.094 | 0.003 | **<0.001** | 0.087 | 0.100 |
| Household Dysfunction, 7-12yrs | 0.004 | 0.105 | 0.973 | -0.201 | 0.208 |
| Age*Household Dysfunction, 7-12yrs | 0.000 | 0.007 | 0.984 | -0.013 | 0.013 |
| Age | 0.094 | 0.003 | **<0.001** | 0.088 | 0.099 |
| Physical abuse, 7-12yrs | 0.239 | 0.412 | 0.562 | -0.569 | 1.047 |
| Age*Physical abuse, 7-12yrs | -0.019 | 0.027 | 0.492 | -0.073 | 0.035 |
| Age | 0.094 | 0.003 | **<0.001** | 0.089 | 0.100 |
| Emotional abuse/neglect, 7-12yrs | 0.158 | 0.156 | 0.309 | -0.147 | 0.464 |
| Age*Emotional abuse/neglect, 7-12yrs | -0.012 | 0.011 | 0.269 | -0.033 | 0.009 |
| Age | 0.094 | 0.003 | **<0.001** | 0.089 | 0.100 |
| Bullying, 7-12yrs | 0.142 | 0.142 | 0.318 | -0.136 | 0.420 |
| Age*Bullying, 7-12yrs | -0.007 | 0.009 | 0.466 | -0.025 | 0.012 |
| Age | 0.093 | 0.003 | **<0.001** | 0.088 | 0.099 |
| Household violence, 7-12yrs | -0.052 | 0.236 | 0.827 | -0.514 | 0.410 |
| Age*Household violence, 7-12yrs | 0.003 | 0.016 | 0.857 | -0.028 | 0.034 |
| Age | 0.094 | 0.003 | **<0.001** | 0.088 | 0.099 |
| Parental Substance Use Problems, 7-12yrs | 0.083 | 0.254 | 0.742 | -0.414 | 0.581 |
| Age*Parental Substance Use Problems, 7-12yrs | -0.012 | 0.018 | 0.514 | -0.046 | 0.023 |
| Age | 0.093 | 0.003 | **<0.001** | 0.087 | 0.099 |
| Parental Mental Health Problems, 7-12yrs | -0.017 | 0.151 | 0.909 | -0.313 | 0.278 |
| Age*Parental Mental Health Problems, 7-12yrs | 0.004 | 0.010 | 0.690 | -0.015 | 0.023 |
| Age | 0.093 | 0.003 | **<0.001** | 0.088 | 0.099 |
| Parental Convictions, 7-12yrs | -0.097 | 0.332 | 0.771 | -0.748 | 0.555 |
| Age*Parental Convictions, 7-12yrs | 0.007 | 0.023 | 0.760 | -0.038 | 0.052 |
| Age | 0.094 | 0.003 | **<0.001** | 0.088 | 0.100 |
| Parental Separation, 7-12yrs | 0.096 | 0.142 | 0.500 | -0.183 | 0.375 |
| Age*Parental Separation, 7-12yrs | -0.005 | 0.009 | 0.563 | -0.024 | 0.013 |
| Age | 0.094 | 0.003 | **<0.001** | 0.088 | 0.100 |
| Low Parent-Child Bonding, 7-12yrs | 0.016 | 0.121 | 0.892 | -0.220 | 0.253 |
| Age*Low Parent-Child Bonding, 7-12yrs | -0.001 | 0.008 | 0.946 | -0.017 | 0.016 |
| **ACEs 12-18 years** |  |  |  |  |  |
| Age | 0.094 | 0.004 | **<0.001** | 0.086 | 0.101 |
| Total ACEs cumulative score, 12-18yrs | 0.028 | 0.043 | 0.521 | -0.057 | 0.113 |
| Age*Total ACEs cumulative score, 12-18yrs | 0.000 | 0.003 | 0.937 | -0.006 | 0.006 |
| Age | 0.094 | 0.003 | **<0.001** | 0.087 | 0.100 |
| Emotional/ Physical threat, 12-18yrs | -0.005 | 0.094 | 0.957 | -0.190 | 0.180 |
| Age*Emotional/ Physical threat, 12-18yrs | 0.000 | 0.006 | 0.993 | -0.012 | 0.012 |
| Age | 0.094 | 0.003 |  | 0.088 | 0.101 |
| Household Dysfunction, 12-18yrs | 0.125 | 0.112 | 0.264 | -0.094 | 0.344 |
| Age*Household Dysfunction, 12-18yrs | -0.004 | 0.007 | 0.621 | -0.018 | 0.011 |
| Age | 0.093 | 0.003 | **<0.001** | 0.087 | 0.099 |
| Physical abuse, 12-18yrs | -0.025 | 0.171 | 0.883 | -0.359 | 0.309 |
| Age*Physical abuse, 12-18yrs | 0.005 | 0.012 | 0.668 | -0.018 | 0.028 |
| Age | 0.094 | 0.003 | **<0.001** | 0.088 | 0.100 |
| Emotional abuse/neglect, 12-18yrs | 0.020 | 0.105 | 0.851 | -0.187 | 0.226 |
| Age*Emotional abuse/neglect, 12-18yrs | -0.002 | 0.007 | 0.822 | -0.016 | 0.012 |
| Age | 0.094 | 0.003 | **<0.001** | 0.088 | 0.099 |
| Sexual abuse, 12-18yrs | 0.071 | 0.222 | 0.750 | -0.365 | 0.506 |
| Age*Sexual abuse, 12-18yrs | -0.002 | 0.015 | 0.881 | -0.032 | 0.027 |
| Age | 0.093 | 0.003 | **<0.001** | 0.087 | 0.099 |
| Bullying, 12-18yrs | 0.003 | 0.114 | 0.982 | -0.220 | 0.225 |
| Age*Bullying, 12-18yrs | 0.002 | 0.008 | 0.786 | -0.013 | 0.017 |
| Age | 0.094 | 0.003 | **<0.001** | 0.088 | 0.100 |
| Household violence, 12-18yrs | 0.141 | 0.171 | 0.411 | -0.195 | 0.476 |
| Age*Household violence, 12-18yrs | -0.006 | 0.011 | 0.561 | -0.027 | 0.015 |
| Age | 0.093 | 0.003 | **<0.001** | 0.088 | 0.099 |
| Parental Substance Use Problems, 12-18yrs | -0.201 | 0.477 | 0.674 | -1.137 | 0.735 |
| Age*Parental Substance Use Problems, 12-18yrs | 0.029 | 0.031 | 0.348 | -0.032 | 0.090 |
| Age | 0.094 | 0.003 | **<0.001** | 0.088 | 0.099 |
| Parental Mental Health Problems, 12-18yrs | 0.164 | 0.349 | 0.637 | -0.519 | 0.848 |
| Age*Parental Mental Health Problems, 12-18yrs | -0.004 | 0.023 | 0.858 | -0.050 | 0.042 |
| Age | 0.094 | 0.003 | **<0.001** | 0.088 | 0.099 |
| Parental Convictions, 12-18yrs | -0.026 | 0.519 | 0.960 | -1.044 | 0.992 |
| Age*Parental Convictions, 12-18yrs | 0.005 | 0.036 | 0.888 | -0.066 | 0.076 |
| Age | 0.094 | 0.003 | **<0.001** | 0.088 | 0.100 |
| Parental Separation, 12-18yrs | 0.093 | 0.144 | 0.520 | -0.189 | 0.374 |
| Age*Parental Separation, 12-18yrs | -0.001 | 0.010 | 0.884 | -0.020 | 0.017 |
| Age | 0.094 | 0.003 | **<0.001** | 0.088 | 0.099 |
| Low Parent-Child Bonding, 12-18yrs | -0.008 | 0.148 | 0.957 | -0.297 | 0.281 |
| Age*Low Parent-Child Bonding, 12-18yrs | 0.000 | 0.010 | 0.980 | -0.020 | 0.019 |
| **ACEs prenatal-18 years** |  |  |  |  |  |
| Age | 0.094 | 0.005 | **<0.001** | 0.085 | 0.104 |
| Total ACEs cumulative score, prenatal-18yrs | 0.011 | 0.026 | 0.669 | -0.040 | 0.062 |
| Age*Total ACEs cumulative score, prenatal-18yrs | 0.000 | 0.002 | 0.836 | -0.004 | 0.003 |
| Age | 0.093 | 0.004 | **<0.001** | 0.085 | 0.101 |
| Emotional/ Physical threat, prenatal-18yrs | -0.035 | 0.086 | 0.685 | -0.203 | 0.133 |
| Age*Emotional/ Physical threat, prenatal-18yrs | 0.002 | 0.006 | 0.750 | -0.009 | 0.013 |
| Age | 0.093 | 0.006 | **<0.001** | 0.082 | 0.105 |
| Household Dysfunction, prenatal-18yrs | 0.009 | 0.099 | 0.925 | -0.184 | 0.203 |
| Age*Household Dysfunction, prenatal-18yrs | 0.000 | 0.007 | 0.960 | -0.013 | 0.013 |
| Age | 0.094 | 0.003 | **<0.001** | 0.088 | 0.100 |
| Physical abuse, prenatal-18yrs | 0.066 | 0.133 | 0.619 | -0.194 | 0.326 |
| Age*Physical abuse, prenatal-18yrs | -0.004 | 0.009 | 0.643 | -0.022 | 0.014 |
| Age | 0.093 | 0.004 | **<0.001** | 0.086 | 0.100 |
| Emotional abuse/neglect, prenatal-18yrs | -0.030 | 0.095 | 0.750 | -0.217 | 0.156 |
| Age*Emotional abuse/neglect, prenatal-18yrs | 0.001 | 0.006 | 0.832 | -0.011 | 0.014 |
| Age | 0.094 | 0.003 | **<0.001** | 0.088 | 0.100 |
| Sexual abuse, prenatal-18yrs | 0.117 | 0.220 | 0.595 | -0.314 | 0.549 |
| Age*Sexual abuse, prenatal-18yrs | -0.006 | 0.015 | 0.692 | -0.035 | 0.023 |
| Age | 0.094 | 0.003 | **<0.001** | 0.088 | 0.100 |
| Bullying, prenatal-18yrs | 0.072 | 0.100 | 0.468 | -0.123 | 0.268 |
| Age*Bullying, prenatal-18yrs | -0.002 | 0.007 | 0.786 | -0.015 | 0.011 |
| Age | 0.093 | 0.003 | **<0.001** | 0.087 | 0.100 |
| Household violence, prenatal-18yrs | 0.025 | 0.108 | 0.818 | -0.186 | 0.236 |
| Age*Household violence, prenatal-18yrs | 0.000 | 0.007 | 0.969 | -0.013 | 0.014 |
| Age | 0.094 | 0.003 | **<0.001** | 0.088 | 0.100 |
| Parental Substance Use Problems, prenatal-18yrs | 0.052 | 0.144 | 0.719 | -0.231 | 0.335 |
| Age*Parental Substance Use Problems, prenatal-18yrs | -0.003 | 0.009 | 0.716 | -0.021 | 0.015 |
| Age | 0.092 | 0.004 | **<0.001** | 0.084 | 0.101 |
| Parental Mental Health Problems, prenatal-18yrs | -0.048 | 0.092 | 0.605 | -0.228 | 0.133 |
| Age*Parental Mental Health Problems, prenatal-18yrs | 0.002 | 0.006 | 0.699 | -0.010 | 0.015 |
| Age | 0.094 | 0.003 | **<0.001** | 0.088 | 0.099 |
| Parental Convictions, prenatal-18yrs | 0.014 | 0.155 | 0.930 | -0.291 | 0.318 |
| Age*Parental Convictions, prenatal-18yrs | -0.001 | 0.010 | 0.905 | -0.021 | 0.019 |
| Age | 0.095 | 0.003 | **<0.001** | 0.088 | 0.101 |
| Parental Separation, prenatal-18yrs | 0.098 | 0.089 | 0.275 | -0.077 | 0.273 |
| Age*Parental Separation, prenatal-18yrs | -0.004 | 0.006 | 0.451 | -0.016 | 0.007 |
| Age | 0.093 | 0.003 | **<0.001** | 0.086 | 0.100 |
| Low Parent-Child Bonding, prenatal-18yrs | -0.047 | 0.103 | 0.649 | -0.248 | 0.154 |
| Age*Low Parent-Child Bonding, prenatal-18yrs | 0.002 | 0.007 | 0.724 | -0.011 | 0.016 |
| **Note.** Sample: ALSPAC (N = 3,931). Pooled estimates from growth curve mixed-effects models across 20 imputed datasets. CI = confidence interval. SE = standard error. Models adjusted for sex, ethnicity, maternal smoking during pregnancy, mother’s marital status, mother’s education, and household’s social class. P-values highlighted in bold are statistically significant at the 95% confidence level. | | | | | |

| **Table S48. Associations of ACEs throughout childhood (prenatal-18 yrs) with moderate-high and high-moderate CRP trajectories (vs low-moderate), with further adjustment for BMI (15 yrs).** | | | | | | | | |
| --- | --- | --- | --- | --- | --- | --- | --- | --- |
|  | **Moderate-high CRP Trajectory** | | | | **High-moderate CRP Trajectory** | | | |
| **Exposure: ACEs** | **OR** | **p-value** | **CI lower** | **CI upper** | **OR** | **p-value** | **CI lower** | **CI upper** |
| Total ACEs cumulative score, prenatal-18yrs | 1.000 | 0.996 | 0.949 | 1.054 | 1.013 | 0.695 | 0.948 | 1.083 |
| Emotional/ Physical threat, prenatal-18yrs | 0.943 | 0.504 | 0.794 | 1.120 | 0.949 | 0.650 | 0.758 | 1.189 |
| Household Dysfunction, prenatal-18yrs | 0.933 | 0.486 | 0.768 | 1.134 | 1.041 | 0.757 | 0.809 | 1.338 |
| Physical abuse, prenatal-18yrs | 0.900 | 0.450 | 0.686 | 1.182 | 1.112 | 0.521 | 0.804 | 1.536 |
| Emotional abuse/neglect, prenatal-18yrs | 0.902 | 0.275 | 0.750 | 1.085 | 0.917 | 0.480 | 0.722 | 1.166 |
| Sexual abuse, prenatal-18yrs | 1.020 | 0.916 | 0.704 | 1.480 | 0.818 | 0.432 | 0.496 | 1.350 |
| Bullying, prenatal-18yrs | 1.085 | 0.411 | 0.893 | 1.318 | 1.338 | **0.016** | 1.056 | 1.694 |
| Household violence, prenatal-18yrs | 1.118 | 0.296 | 0.907 | 1.379 | 1.022 | 0.871 | 0.789 | 1.323 |
| Parental Substance Use Problems, prenatal-18yrs | 0.804 | 0.139 | 0.602 | 1.073 | 1.285 | 0.141 | 0.920 | 1.795 |
| Parental Mental Health Problems, prenatal-18yrs | 0.902 | 0.250 | 0.756 | 1.076 | 0.878 | 0.263 | 0.700 | 1.102 |
| Parental Convictions, prenatal-18yrs | 1.054 | 0.753 | 0.760 | 1.462 | 0.887 | 0.591 | 0.574 | 1.372 |
| Parental Separation, prenatal-18yrs | 1.163 | 0.147 | 0.948 | 1.427 | 1.051 | 0.701 | 0.816 | 1.354 |
| Low Parent-Child Bonding, prenatal-18yrs | 1.037 | 0.726 | 0.847 | 1.269 | 0.963 | 0.785 | 0.736 | 1.261 |
| **Note.** Sample: ALSPAC (N = 3,931). Pooled estimates from multinomial logistic regression models across 20 imputed models. Reference outcome: Low-moderate CRP trajectory. CI = confidence interval. SE = standard error. ACEs = adverse childhood experiences. BMI = body mass index. Models adjusted for sex, ethnicity, maternal smoking during pregnancy, mother’s marital status, mother’s education, household’s social class, and BMI 15 yrs. P-values highlighted in bold are statistically significant at the 95% confidence level. | | | | | | | | |

| **Table S49. Associations of the individual CRP measures and CRP trajectories with moderate and severe depression trajectories (vs low), with further adjustment for BMI (15 yrs).** | | | | | | | | |
| --- | --- | --- | --- | --- | --- | --- | --- | --- |
|  | **Moderate Trajectory** | | | | **Severe Trajectory** | | | |
| **Exposure: CRP** | **OR** | **p-value** | **CI lower** | **CI upper** | **OR** | **p-value** | **CI lower** | **CI upper** |
| *Single CRP measures* |  |  |  |  |  |  |  |  |
| CRP 18 yrs (log) | 0.945 | 0.214 | 0.865 | 1.033 | 0.945 | 0.415 | 0.825 | 1.083 |
| CRP 15 yrs (log) | 0.995 | 0.908 | 0.906 | 1.092 | 0.972 | 0.719 | 0.835 | 1.133 |
| CRP 9 yrs (log) | 0.942 | 0.151 | 0.868 | 1.022 | 0.977 | 0.716 | 0.859 | 1.110 |
| *CRP Trajectories* |  |  |  |  |  |  |  |  |
| Low-Moderate CRP Trajectory | ref |  |  |  | ref |  |  |  |
| Moderate-High CRP Trajectory | 0.962 | 0.695 | 0.793 | 1.167 | 0.854 | 0.230 | 0.660 | 1.105 |
| High-Moderate CRP Trajectory | 0.938 | 0.683 | 0.690 | 1.275 | 1.109 | 0.585 | 0.765 | 1.608 |
| **Note.** Sample: ALSPAC (N = 3,931). Pooled estimates from multinomial logistic regression models across 20 imputed models. Reference outcome: Low depression trajectory. CI = confidence interval. SE = standard error. ACEs = adverse childhood experiences. BMI = body mass index. Models adjusted for sex, ethnicity, maternal smoking during pregnancy, mother’s marital status, mother’s education, household’s social class, and BMI 15 yrs. P-values highlighted in bold are statistically significant at the 95% confidence level. | | | | | | | | |

| **Table S50. Associations of child-reported versus parent-reported emotional neglect (16 yrs) with depression (18 yrs).** | | | | | |
| --- | --- | --- | --- | --- | --- |
| **Exposure: Emotional neglect** | **OR** | **SE** | **P-value** | **Lower CI** | **Upper CI** |
| Child-reported emotional neglect | 1.400 | 0.213 | **0.027** | 1.039 | 1.886 |
| Parent-reported emotional neglect | 1.105 | 0.095 | 0.247 | 0.933 | 1.308 |
| **Note.** Sample: ALSPAC (N = 3,931). Pooled estimates from logistic regression models across 20 imputed models. Outcome: depression at 18 years (SMFQ total score ≥ 10). CI = confidence interval. SE = standard error. ACEs = adverse childhood experiences. Models adjusted for sex, ethnicity, maternal smoking during pregnancy, mother’s marital status, mother’s education, and household’s social class. P-values highlighted in bold are statistically significant at the 95% confidence level. | | | | | |
